# Inter-domain dynamics in the chaperone SurA and multi-site binding to its outer membrane protein clients

**DOI:** 10.1038/s41467-020-15702-1

**Published:** 2020-05-01

**Authors:** Antonio N. Calabrese, Bob Schiffrin, Matthew Watson, Theodoros K. Karamanos, Martin Walko, Julia R. Humes, Jim E. Horne, Paul White, Andrew J. Wilson, Antreas C. Kalli, Roman Tuma, Alison E. Ashcroft, David J. Brockwell, Sheena E. Radford

**Affiliations:** 1 Astbury Centre for Structural Molecular Biology, School of Molecular and Cellular Biology, Faculty of Biological Sciences, University of Leeds, Leeds, LS2 9JT UK; 2 Astbury Centre for Structural Molecular Biology, School of Chemistry, University of Leeds, Leeds, LS2 9JT UK; 3 Astbury Centre for Structural Molecular Biology and School of Medicine, University of Leeds, Leeds, LS2 9JT UK; 4 Faculty of Science, University of South Bohemia, Ceske Budejovice, Czech Republic; 5 Present Address: National Institute of Diabetes and Digestive and Kidney Diseases, National Institutes of Health, Bethesda, MD 20892 USA

**Keywords:** Chaperones, Mass spectrometry, Bacterial structural biology, Structural biology

## Abstract

The periplasmic chaperone SurA plays a key role in outer membrane protein (OMP) biogenesis. *E. coli* SurA comprises a core domain and two peptidylprolyl isomerase domains (P1 and P2), but its mechanisms of client binding and chaperone function have remained unclear. Here, we use chemical cross-linking, hydrogen-deuterium exchange mass spectrometry, single-molecule FRET and molecular dynamics simulations to map the client binding site(s) on SurA and interrogate the role of conformational dynamics in OMP recognition. We demonstrate that SurA samples an array of conformations in solution in which P2 primarily lies closer to the core/P1 domains than suggested in the SurA crystal structure. OMP binding sites are located primarily in the core domain, and OMP binding results in conformational changes between the core/P1 domains. Together, the results suggest that unfolded OMP substrates bind in a cradle formed between the SurA domains, with structural flexibility between domains assisting OMP recognition, binding and release.

## Abstract (web summary)

The chaperone SurA is involved in outer membrane protein (OMP) biogenesis in Gram-negative bacteria, but its mechanism of action is not fully understood. Combining mass spectrometric, biophysical and computational approaches, the authors here show how the conformational dynamics of SurA facilitate OMP binding.

## Introduction

Chaperones play vital roles in multicomponent proteostasis networks, ensuring that proteins fold and avoid aggregation in the crowded cellular milieu, and that misfolded proteins which cannot be rescued by chaperones are targeted for degradation^1,2^. It is now established that many chaperones are in rapid dynamic exchange between co-populated conformations, and that this conformational plasticity is key to their functional mechanisms^3^. In the case of ATP-dependent chaperones, e.g. the Hsp60 chaperonins GroEL and TRiC, and the Hsp90 and Hsp70 families, ATP binding and/or hydrolysis promotes conformational changes that facilitate the folding and/or release of their clients^1,2,4–8^. However, some chaperones are not dependent on energy from nucleotide binding/hydrolysis, and instead their intrinsic structural flexibility is proposed to be key to their function^3,9–12^. The functional mechanisms of these ATP-independent chaperones, including how they bind and release their substrates in a controlled manner, are generally not well understood.

**Fig. 1:**
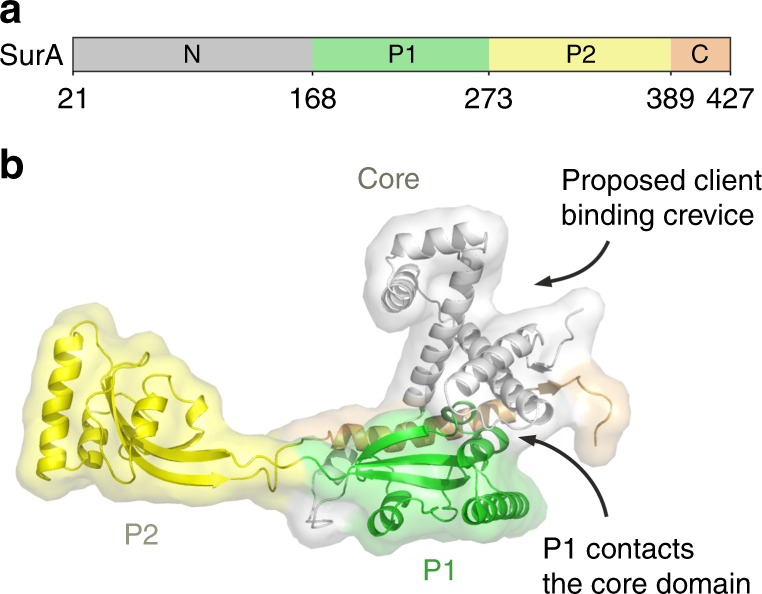
SurA structure and domain architecture. **a** Domain architecture of *E. coli* SurA. Regions are coloured grey (N-terminal region of the core domain), green (P1), yellow (P2) and orange (C-terminal region of the core domain). The signal sequence is not shown, and was not present in any of the constructs used in this study, but the numbering used throughout reflects the gene numbering (including the signal peptide). **b** Crystal structure of *E. coli* SurA WT (PDB 1M5Y^27^), with missing residues added using MODELLER^106^. A client binding crevice in the core domain was proposed based on crystal contacts with a neighbouring SurA molecule, as indicated^27^. P1 contacts the core domain in this crystal structure (Supplementary Fig. 1a-c). Regions are coloured as in (**a**).

SurA is an ATP-independent chaperone involved in the biogenesis of outer membrane proteins (OMPs) in the periplasm of Gram-negative bacteria^13–18^. This protein is thought to be the major chaperone responsible for protecting OMPs from aggregation in the periplasm^13–18^ and facilitating OMP delivery to the β-barrel assembly machinery (BAM) for folding and insertion into the outer membrane (OM)^13,14,19–21^. Deletion of SurA leads to OMP assembly defects, the induction of stress responses, and increased sensitivity to antibiotics and detergents^15,17,22–24^. Further, *ΔsurA* strains show reduced assembly of virulence factors, such as pili and adhesins, and exhibit reduced pathogenicity in a number of species^22,25,26^. *E. coli* SurA has a three domain architecture, consisting of a core domain which is composed of its N- and C-terminal regions, and two parvulin-like peptidylprolyl isomerase (PPIase) domains (P1 and P2) (Fig. 1a)^27^. However, despite the availability of its crystal structure^27^, how SurA binds its unfolded OMP clients and the molecular mechanism(s) of SurA function remain unknown. A substrate binding crevice was proposed based on examination of molecular packing interactions in crystals of SurA (Fig. 1b), but the location of OMP binding regions and the roles of the PPIase domains (which are not essential for in vivo or in vitro function, at least for some clients^24,28,29^) in folding and binding its varied OMP clients remained unknown.

In the crystal structure of full-length SurA^27^, an extended conformation is observed in which the core and P1 domains are in contact (Fig. 1b, Supplementary Fig. 1a-c), while P2 is separated from this globular region via a linker (Fig. 1b, Supplementary Fig. 1d). Examination of the molecular packing interactions in the crystal lattice revealed multiple contacts between all three domains and neighbouring molecules, which may stabilise the elongated architecture observed (Supplementary Fig. 2). SurA homologues and domain deletion variants have been crystallised in conformations with a variety of domain orientations (Supplementary Fig. 3) suggesting that SurA may have a dynamic structure. Further, tethering of the P1 and core domains via a disulfide bond resulted in impaired OMP assembly in vivo^29^. However, the precise nature of these conformational dynamics and how they are linked to OMP binding have remained elusive.

Here, we sought to determine the conformational properties of full-length *E. coli* SurA in solution in an effort to better understand its conformational dynamics and how inter-domain motions may be exploited or modified by client binding. Combining mass spectrometric (MS) methods (chemical cross-linking (XL) and hydrogen-deuterium exchange (HDX)), with single-molecule FRET (smFRET) and molecular dynamics (MD) simulations we show that SurA adopts conformations in solution that differ substantially from its crystal structure^27^. Specifically, the P1 domain samples open and closed states relative to the core domain, and P2 is primarily located closer to the core/P1 domains than observed in the crystal structure. We also show that multiple sites on the SurA surface, predominantly located in the core domain, are involved in client binding. OMPs bind to these specific sites in different orientations, consistent with a dynamically bound state, and the conformations adopted by the chaperone alter in response to OMP binding. Combined, our results portray a model in which the three domains of SurA form a cradle around its OMP clients, protecting them from misfolding and aggregation on their journey through the periplasm, with the conformational dynamics of the domains presumably facilitating their delivery to BAM for folding into the outer membrane.

## Results

### Inter-domain conformational flexibility in SurA

**Fig. 2:**
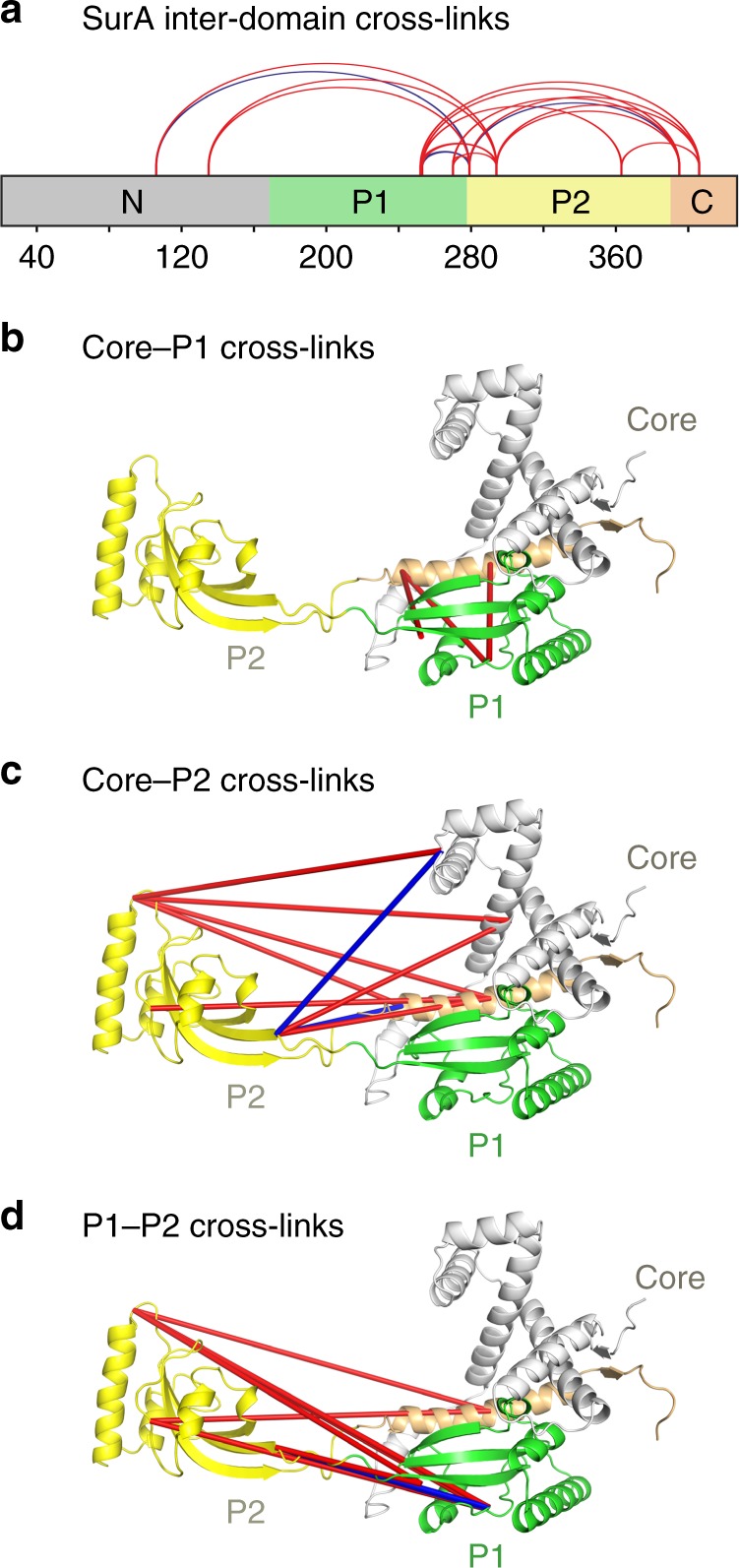
XL-MS suggests the P2 domain is closer to the core/P1 than implied by the crystal structure. **a** Locations of the 19 identified SurA inter-domain cross-links (red and blue lines). **b**–**d** Crystal structure of SurA showing the identified inter-domain cross-links between **b** core-P1 (3 in total), **c** core-P2 (9 in total), and **d** P1-P2 (7 in total). Only four of the inter-domain cross-links identified (coloured in blue) are consistent with the crystal structure of full-length SurA (PDB 1M5Y^27^), based on a maximum SASD of 35 Å^35^. Other cross-links are inconsistent with this distance cut-off (red lines). Details of cross-linked residues are given in Supplementary Table 1. A representative mass spectrum for each cross-link can be found in Supplementary Data 1. Reactions contained 5 µM SurA, 50 µM DSBU, in 10 mM potassium phosphate buffer, pH 8.0, for 45 min, 25 °C. Note that for clarity cross-links are shown as straight lines between residues (rather than as SASDs which provide a more reliable measurement for comparison with protein structures (see text)). The N-terminal region of the core domain, P1, P2 and the C-terminal region of the core domain are shown in grey, green, yellow and orange, respectively.

We first investigated the structure and dynamics of apo-SurA in solution using XL-MS, which provides distance information in the form of spatial restraints, and enables comparison of the solution conformation(s) of the protein with structural data^30^. For this purpose, we used the bifunctional reagent disuccinimidyl dibutyric urea (DSBU), which primarily cross-links Lys residues^31^, and a SurA concentration at which the protein is monomeric in solution (5 μM)^32^. The concentration of SurA in the periplasm has been estimated to be ~7 μM suggesting it is a functional monomer^33^. DSBU has been shown to cross-link residues within a straight line distance (SLD) between their Cα atoms of ca. 27–30 Å^34^. More recently it has been shown that considering the solvent accessible surface distance (SASD) between residues may more reliably predict structural models^35^ (a Cα–Cα distance between cross-linked residues of up to ca. 35 Å is considered feasible for DSBU). For monomeric SurA, a total of 13 intra-domain (core-core, P1-P1 and P2-P2) (Supplementary Fig. 4, Supplementary Table 1) and 19 inter-domain (core-P1, core-P2 and P1-P2) cross-links were detected (Fig. 2a, Supplementary Table 1). Most of the intra-domain cross-links identified (8 of 13, based on the SASD) are consistent with the domain structures observed in the crystal structure of full-length *E. coli* SurA (Supplementary Fig. 4, Supplementary Table 1), but there is some suggestion of local fluctuations within the domain structures, with five unsatisfied cross-links (K90-K134, K134-K394 and K134-K405 in the core domain, and two, K278-K293 and K362-K388, in the P2 domain). Interestingly, two of the five unsatisfied intra-domain cross-links (K134-K394 in the core domain, and K362-K388 in the P2 domain) involve a residue that was not present/disordered in the crystal structure (and was built in using MODELLER), while the remaining three cross-links (K90-K134 and K134-K405 in the core domain, and K278-K293 in the P2 domain) involve Lys residues in regions of defined secondary structure (Supplementary Fig. 1d). By contrast, only four (K105-K278, K278-K394, K251-K278, K252-278) of the 19 inter-domain cross-links (core-P1, core-P2 or P1-P2) are compatible with SASD values derived from the SurA crystal structure (Fig. 2b–d, Supplementary Table 1). Interestingly, the three core-P1 cross-links detected (K251-K405, K252-K394 and K269-K394) involve residues that have SLDs of ~26 Å^36^. However, the vectors of the SLDs for these cross-links pass directly through the P1 and core domains, highlighting the importance of considering SASDs in judging cross-link violation^35,37^. Taken together, the data show that SurA populates structures in solution in which P2 is closer to both the core and P1 domains than portrayed by its crystal structure, as well as conformations in which the orientation and/or distance of P1 relative to the core is distinct from that observed in the crystal structure of the protein^27^.

**Fig. 3:**
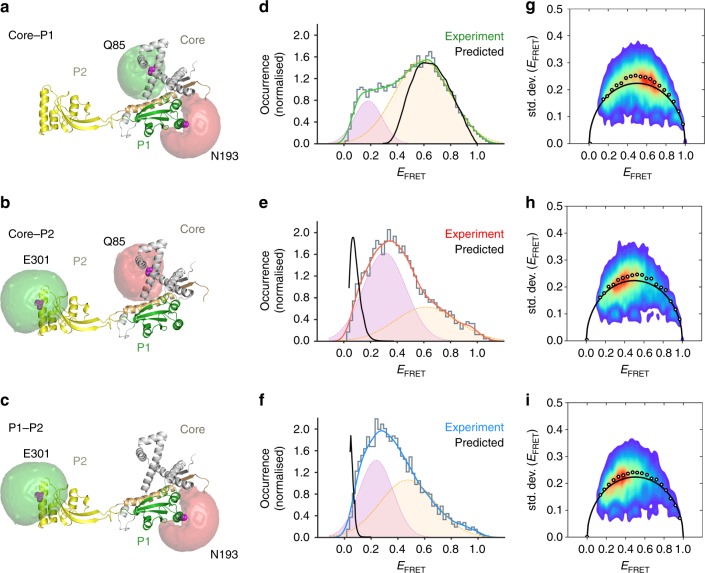
Comparison of smFRET data to values predicted from crystal structure of SurA. The crystal structure of SurA indicating the available volumes calculated for each dye label at residues **a** 85 and 193 (core-P1 distance probes); **b** 85 and 301 (core-P2 distance probes) and **c** 193 and 301 (P1-P2 distance probes). **d**–**f** Predicted E_FRET_ between each pair of dyes for the crystal structure of SurA (black line) and the observed E_FRET_ distributions (green, red or blue lines) independently fitted to two Gaussians (pink/yellow). **g**–**i** BVA reveals high variance for all three dye pairs. The average values of the measured variance for the E_FRET_ values (white filled circles) lie above the expected shot-noise limited standard deviation (black arc) indicating dynamics on a timescale faster than the duration of the bursts (here sub-ms). Predicted E_FRET_ distributions **d**–**f** were calculated from the SurA crystal structure (PDB 1M5Y^27^) using distance distributions generated by the MtsslWizard plugin for PyMOL^103^, which takes into account both the location of the dyes and the flexibility of the dye linkers^39^. Note that all dyes can be considered as freely rotating, as manifested by their low anisotropy (Supplementary Table 2), hence changes in E_FRET_ can be translated to distance variations. Samples contained ~50 pM labelled SurA variant in 50 mM Tris-HCl, pH 8.0, 25 °C.

As an independent validation of the cross-linking results, we investigated the inter-domain distances in monomeric SurA by smFRET^38^. We selected non-conserved residues in the core, P1 and P2 domains (Q85, N193 and E301, respectively) to substitute with Cys, and constructed three variants containing Cys substitutions at two positions (core-P1, core-P2 and P1-P2) (Fig. 3a–c, Supplementary Table 2). Each SurA variant was stochastically labelled with Alexa 488 and Alexa 594 dyes (*R*_0_ = 60 Å), enabling inter-domain distance distributions to be monitored in a pairwise fashion. Samples containing ~50 pM of labelled monomeric SurA were interrogated using confocal fluorescence detection and alternating laser excitation (ALEX) (see Methods). Fluorescence intensities in the donor and acceptor channels yielded FRET efficiencies (E_FRET_) for the passage of each single molecule through the confocal volume (fluorescence burst). These were collated into FRET efficiency histograms, fitted to two Gaussian components and compared with distributions predicted for each labelled SurA double Cys variant calculated from the SurA crystal structure (see Methods) (Fig. 3d–f)^39,40^.

The predicted E_FRET_ distribution for the core-P1 SurA variant calculated from the crystal structure of *E. coli* SurA^27^ has a single maximum at ~0.6 (Fig. 3d). While the experimentally observed distribution recapitulates this predicted peak maximum, it also shows a second, smaller, population centred on E_FRET_ ~ 0.2 (Fig. 3d). This suggests that SurA populates at least two distinct conformational ensembles in solution, one in which P1 is located close to the core domain with an inter-domain distance similar to that in the crystal structure (core-P1_closed_ ensemble), and one in which the P1 and core domains are further apart (core-P1_open_ ensemble). The observed E_FRET_ distributions for the labelled core-P2 and P1-P2 variants were also obtained and fitted to two Gaussians with maxima of the most intense peak at ~0.3 and ~0.2, respectively, and smaller peaks at ~0.6 and ~0.5, respectively (Fig. 3e, f), both in marked contrast with the very low predicted values for the crystal structure (~0.1 and ~0.02, a spatial separation of ~85 and ~115 Å, for core-P2 and P1-P2, respectively). This indicates that, in the vast majority of molecules, P2 is located closer to the core and P1 domains than suggested by the SurA crystal structure^27^, consistent with the XL-MS data (Fig. 2b–d), and that there are at least two discernible conformational ensembles. Note that the concentrations of SurA used here are lower than those found in vivo (50 pM vs 7 μM in the periplasm^33^). At higher concentrations, and indeed in the crowded, dynamic periplasm, the conformational ensembles of SurA may be influenced by a number of factors, including excluded volume effects, higher viscosity, perturbed diffusion, and protein–protein interactions. For example, excluded volume effects may favour more compact conformations while higher viscosity generally slows down segmental motions.

Burst variance analysis (BVA)^41^ was used to determine if dynamic interconversion between states was occurring on a sub-millisecond timescale. BVA compares the FRET efficiency variance derived from the experimental data within each burst with the theoretical shot-noise limited variance expected for static FRET values. Dynamic interconversion between FRET states on a timescale faster than the duration of the burst (i.e. the average time it takes a molecule to diffuse through the confocal volume, in this case less than a millisecond) will add to the experimental variance which in turn will be larger than the shot-noise limited value. For each FRET pair (Fig. 3a–c), the experimental FRET variance was larger than that predicted for a static molecule, demonstrating that inter-domain motions involving each pair of domains occurs on the sub-millisecond timescale (Fig. 3g–i). Together, these data indicate a dynamic chaperone in which sub-ms motions involving all three domains are occurring, in particular at the core-P1 interface which interconverts between core-P1_closed_ and core-P1_open_ states. In addition, the data show that P2 is also mobile, consistent with dynamic interconversion between conformational states on the sub-ms timescale as suggested by BVA analysis, but spends most of its time closer to the core and P1 domains than suggested by the SurA crystal structure.

To help visualise the possible conformational excursions of the different domains of apo-SurA in solution we performed unrestrained all-atom molecular dynamics (MD) simulations. Initially three 1-μs simulations were performed starting from the crystal structure of full-length SurA (PDB 1M5Y^27^). However, in these simulations the individual domains of SurA were unstable, with both P1 and P2 unfolding. Therefore, we built an alternative starting model of full-length SurA in which the core and P1 domains are spatially separated, consistent with the smFRET data (SurA^core-P1-open^) (Supplementary Fig. 5a, see Methods). In the three 1-μs simulations performed using this model as a starting structure, each domain remained folded and a wide variety of conformations was observed in which the distances between the domains differed markedly (Supplementary Fig. 5b-g & Supplementary Movies 1–3). While the three endpoint structures of these simulations satisfy more of the detected inter-domain cross-links than the crystal structure (an additional 5, Supplementary Table 3), they do not satisfy all of the cross-links observed. However, 18 of the 19 inter-domain cross-links are compatible with conformations of SurA that were sampled during the three 1-μs simulations (Supplementary Table 4), consistent with SurA adopting a broad array of conformations in solution that are in rapid exchange.

We also performed simulated annealing MD simulations of SurA using the detected inter-domain cross-links as distance restraints (see Methods) in order to visualise possible conformations of the chaperone in which P1 and/or P2 are docked onto the core domain (each domain was treated as a rigid body). All 19 inter-domain cross-links were used as restraints in these simulations. In the lowest energy structure obtained by this approach (structure 1 in Supplementary Fig. 6) all of the 19 inter-domain cross-links were satisfied (Supplementary Fig. 6, Supplementary Table 5). However, this does not suggest that SurA adopts a unique structure in solution, and indeed other structures obtained in the simulated annealing calculations with different domain orientations explain the observed cross-links almost equally well (the 10 lowest energy structures are shown in Supplementary Fig. 6, at least 15/19 inter-domain cross-links were satisfied in each structure, Supplementary Table 5). In these structures a range of SurA domain orientations are observed (Supplementary Fig. 6b), with P2 docking against the core in all 10 structures, whereas P1 adopts a range of conformations (Supplementary Fig. 6).

Given that the simulated annealing approach will drive SurA to adopt compact states that satisfy the maximum number of cross-link restraints within a single structure, more extended states of SurA that are significantly populated in solution, as shown by the smFRET data (Fig. 3), will not be captured by this approach. Indeed, as shown by the smFRET and unrestrained MD simulations, the dynamic nature of SurA makes it challenging to define its precise conformational landscape, wherein a repertoire of conformations in dynamic exchange on a sub-millisecond timescale are formed. Consistent with this, no single structure can satisfy the broad distributions observed by smFRET (Supplementary Table 6), providing further evidence that the cross-links observed cannot all result from a single SurA conformation, but result from different rapidly interconverting states. Together, the unrestrained all-atom MD and simulated annealing simulations demonstrate that the three domains of SurA are able to move independently of each other as rigid bodies, facilitated by the flexible linker regions between them (Supplementary Fig. 1d). This results in chaperone structures with a broad range of inter-domain distances and orientations in rapid exchange as confirmed by smFRET.

### SurA binds its OMP substrates at multiple interaction sites

**Fig. 4:**
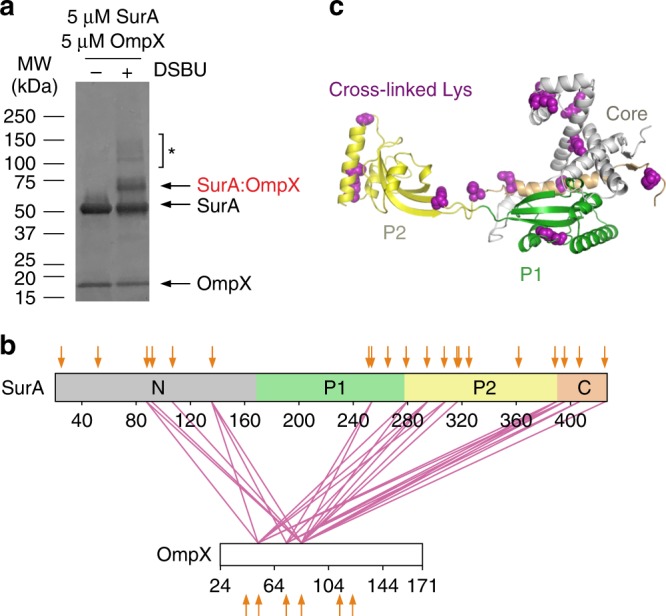
Multi-site binding of OmpX to SurA. **a** SDS-PAGE analysis of DSBU cross-linked SurA-OmpX. Note that the species indicated with an asterisk (*) are higher order cross-linked species of mass corresponding to multiple SurA molecules bound to OmpX, consistent with multivalent binding observed previously^46^. These were not analysed further here. Source data are provided as a Source Data file. **b** Inter-molecular cross-links detected in the SurA-OmpX complex. The location of all Lys residues are indicated with orange arrows. **c** Crystal structure of SurA (PDB 1M5Y^27^). Purple spheres indicate identified cross-link sites (Supplementary Table 7). Samples contained 5 µM SurA, 5 µM OmpX, 0.24 M urea, 50 µM–2 mM DSBU, in 10 mM potassium phosphate buffer, pH 8.0, 25 °C. A representative mass spectrum for each cross-link can be found in Supplementary Data 1.

We next investigated how SurA binds its OMP clients, and how this affects conformations adopted by the chaperone. While NMR studies have shown that OMP substrates bound to SurA remain in a dynamic, unfolded state^42–44^, their binding site(s) on SurA remained unexplored. To map the OMP interaction surface on SurA we used *E. coli* OmpX (16 kDa) as a model substrate (OmpX forms an 8-stranded β-barrel in its native state). SurA binds unfolded OmpX with a *K*_d,app_ of ~800 nM, as measured by microscale thermophoresis (MST) (Supplementary Fig. 7), similar to the affinity of SurA for other OMPs^28,45,46^. SurA-OmpX complexes were assembled by rapid dilution of urea-denatured OmpX into a solution of SurA (final concentrations: 5 μM OmpX, 5 μM SurA, 0.24 M urea) (see Methods) immediately prior to cross-linking with DSBU. A band corresponding to cross-linked SurA-OmpX complexes could be observed by SDS-PAGE (Fig. 4a), and following in-gel digestion a total of 26 unique inter-molecular cross-linked peptides were detected (Fig. 4b, Supplementary Table 7, Supplementary Data 1). Sixteen of the 26 unique crosslinks between SurA and OmpX are in the core domain (Fig. 4c, Supplementary Table 7). Four Lys residues in P2, one in P1, and 7 in the core cross-linked to OmpX, but no cross-links were detected for the remaining 11 Lys residues in P1 and P2 indicating either that these regions are not involved in the interaction or that the orientations of residue side-chains hampered the XL reaction at these sites (Fig. 4b, c). Importantly, several cross-links were detected from the same residue in OmpX to several residues on SurA (e.g. residue 82 of OmpX cross-links to 13 different residues in SurA spanning all four regions of the chain, Supplementary Table 7). Similarly, the same site on SurA cross-linked to multiple sites on OmpX (e.g. residues 135, 294, 389 and 395 in SurA each cross-link to three residues (50, 71 and 82) in OmpX, Supplementary Table 7) consistent with a flexible and dynamic OmpX in the bound state (Fig. 4b).

**Fig. 5:**
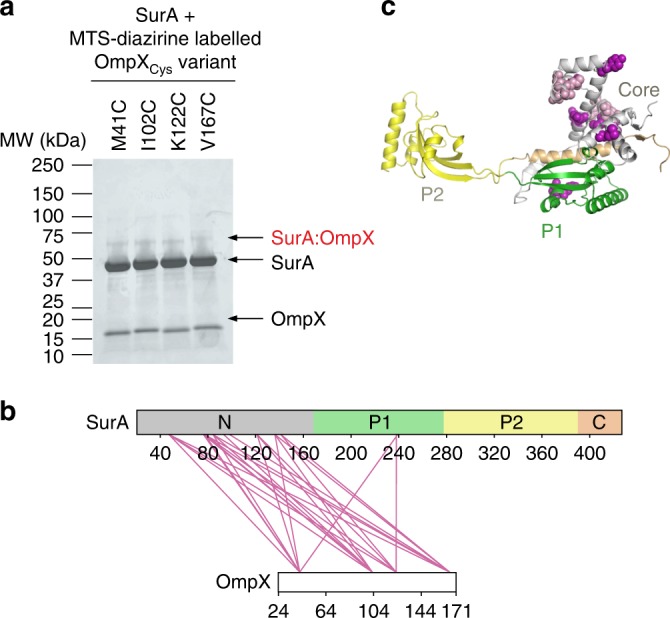
Multiple locations across the OmpX sequence interact with similar sites on SurA. **a** Tag-transfer photo-cross-linking^48^ of SurA-OmpX complexes using OmpX Cys variants labelled with MTS-diazirine analysed by SDS-PAGE. A band corresponding to the SurA-OmpX complex is observed for all OmpX variants following UV irradiation. These bands were not observed when analysed using reducing SDS-PAGE (Supplementary Fig. 8b). Source data are provided as a Source Data file. **b** Inter-molecular cross-links detected in the SurA-OmpX complex (Supplementary Table 8). **c** Structure of SurA with residues which were photo-cross-linked to labelled OmpX Cys variants shown in purple. Where the data quality did not permit residue level assignment, the cross-linked peptide is shown in light purple. Samples contained 10 μM SurA, 5 µM MTS-diazirine-labelled OmpX, 0.24 M urea, in 10 mM potassium phosphate buffer, pH 8.0, 25 °C and cross-linking was initiated by UV LED irradiation of the sample for 30 s (see Methods). A representative mass spectrum for each cross-link can be found in Supplementary Data 1.

The extended spacer arm length of DSBU and its limited reactivity (primarily with Lys residues), makes it challenging to precisely define the interaction interface for OmpX on SurA. To probe the organisation of the SurA-OmpX complex in more detail we thus exploited the ability of the photoactivatable cross-linker MTS-diazirine (Supplementary Fig. 8a) to react rapidly (within ns^47^), and non-specifically with any residue within ~15 Å of the diazirine moiety (Cα-Cα Euclidean distance)^48^. This “tag transfer” method was developed specifically to enable detection of weak and transient protein–protein interactions^48^. We created four MTS-diazirine-labelled single-Cys variants of OmpX (M41C, I102C, K122C, V167C), formed complexes of each with SurA, and following rapid UV irradiation (for only 30 s)^48^, identified the cross-linked products by LC-MS/MS (Fig. 5a–c, Supplementary Fig. 8a-c and Supplementary Table 8, Supplementary Data 1)^48^. In this experimental design (Supplementary Fig. 8a), all amino acid substitutions were performed on the OMP, which remains dynamically unfolded upon binding SurA^44^. Despite the location of the labelled Cys residues in distant regions of the OmpX sequence and the lack of specificity of the diazirine cross-linker^48^, all four Cys-OmpX variants cross-linked to the N-terminal domain of SurA (11 cross-linked sites were identified, Supplementary Table 8) and P1 (two cross-linked sites), indicating that these regions form the heart of the binding epitope (Fig. 5b, c, Supplementary Fig. 8c). Notably, no cross-links were detected between OmpX and the SurA P2 domain or C-terminal region, despite the highly promiscuous photoactivatable cross-linker employed. This differs from the SurA-OmpX cross-links detected with DSBU, probably because (1) the much longer cross-linking time (45 min) required for cross-linking with DSBU, compared with 30 s for the diazirine, permits conformational excursions during the cross-linking reaction; and/or (2) the increased spacer arm length and cross-linking distance (ca. 27–30 Å) of DSBU, compared with the tag-transfer XL (ca. 15 Å), enables DSBU to capture longer-range interactions. Overall, therefore, the results suggest that OmpX adopts a range of likely interconverting conformations upon binding SurA, in which multiple specific interactions are formed predominantly with the N-terminal region of the chaperone core domain.

### Conformational changes in SurA upon OMP binding

**Fig. 6:**
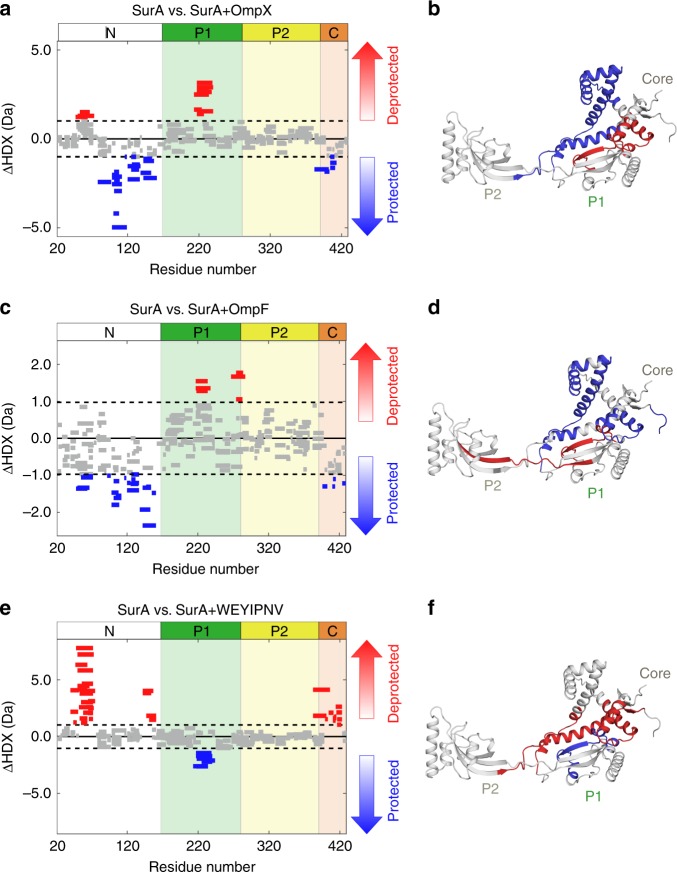
SurA binding to different substrates leads to varying patterns of protection and deprotection by differential HDX-MS analysis. Wood’s plots showing the summed differences in deuterium uptake in SurA over all four HDX timepoints, comparing SurA alone with SurA in the presence of **a** OmpX, **c** OmpF or **e** WEYIPNV. Wood’s plots were generated using Deuteros^115^. Peptides coloured in blue or red, respectively, are protected or deprotected from exchange in the presence of OmpX/OmpF/WEYIPNV. Peptides with no significant difference between conditions, determined using a 99% confidence interval (dotted line), are shown in grey. Regions of SurA protected or deprotected in the presence of **b** OmpX, **d** OmpF and **f** WEYIPNV coloured in blue or red, respectively. Example deuterium uptake curves are shown in Supplementary Fig. 9. See Methods for experimental details.

Next, we examined the conformational changes induced by OMP binding to SurA using differential HDX-MS analysis (Fig. 6a–f and Supplementary Fig. 9). We first compared the uptake of deuterium by different regions of SurA in the presence or absence of OmpX under conditions which minimise OMP aggregation (10 mM potassium phosphate, pH 8.0, 0.24 M urea, 4 ˚C^28,49,50^) (Fig. 6a, b, Supplementary Fig. 9a). In the presence of OmpX, regions in SurA that are protected from deuterium uptake upon substrate binding all cluster to the core domain. No change in protection in P2 was detected in the presence of OmpX, consistent with the tag transfer XL-MS results and with previous results which have shown that P2 is not required to prevent the aggregation of the small (8-stranded) tOmpA^28^. Intriguingly, two regions of SurA (residues 46–72 in the N-terminal region and 212–239 in P1) (Fig. 6a, b, Supplementary Fig. 9a), that are located at the core-P1 interface (Supplementary Fig. 1d), were deprotected upon OmpX binding, demonstrating a structural reorganisation of this interface in response to substrate binding.

To determine whether deprotection at the core-P1 interface occurs in the presence of other OMPs, the effects of binding the larger substrate, OmpF (16-stranded), on the HDX properties of SurA was examined. In the presence of OmpF, residues in the N- and C-terminal regions of the core domain were also protected from exchange, consistent with shared OmpX and OmpF binding sites. However, in marked contrast with the results for OmpX in which residues 46–72 of SurA were deprotected from exchange upon substrate binding, these residues were instead protected from exchange in the presence of OmpF, suggesting that the larger OMP binds to, or occludes, a greater surface area on the core (Fig. 6c, d, Supplementary Fig. 9b). Importantly, as for OmpX, deprotection was observed in the P1 domain at the core-P1 interface, suggesting that structural reorganisation of this interface also occurs upon OmpF binding. Notably, the hinge region between P1 and P2 (residues 266–286) was also deprotected in the presence of OmpF, suggesting that binding of the larger substrate may also alter the conformational dynamics at locations more distal to the core.

To decouple the phenomena of protection arising as a result of OmpX/OmpF binding and deprotection as a result of conformational changes in SurA, we also compared the levels of deuterium uptake of SurA in the presence of a 7-residue peptide known to bind to the P1 domain (WEYIPNV, *K*_d_ 1–14 μM)^51,52^ (Fig. 6e, f, Supplementary Fig. 9c,d). Interestingly, extensive deprotection at the core-P1 interface (residues 39–74, 142–160 and 381–422) was observed in the presence of WEYIPNV, while protection was only observed in P1 at the known peptide binding site (residues 212–243^52^) (Supplementary Fig. 9c,d). Combined, these results demonstrate that the OMP substrate binding surface is more extensive in OmpX/OmpF compared with WEYIPNV, but in all three cases binding triggers a structural reorganisation between the core and P1 domains.

**Fig. 7:**
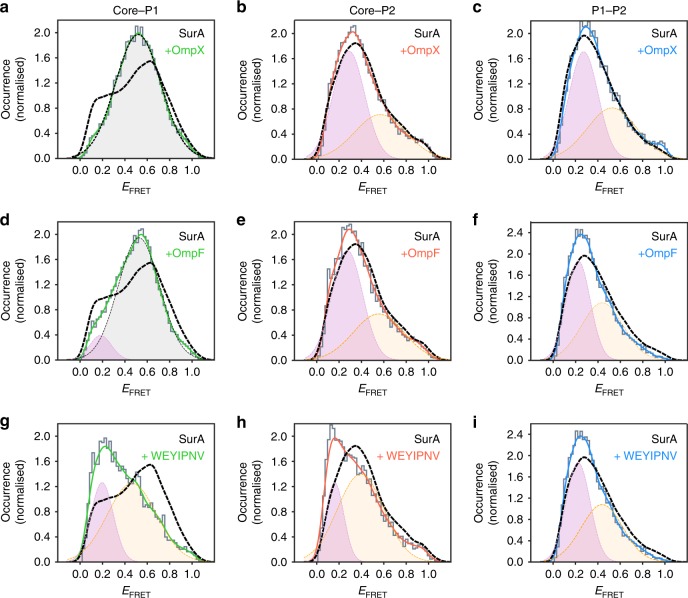
Response of SurA inter-domain distances to substrate binding measured by smFRET. Experimentally measured E_FRET_ distributions (grey) at equilibrium for the three pairwise combinations of fluorescently-labelled SurA double mutants (core-P1, core-P2, and P1-P2) in the presence of **a**–**c** OmpX, **d**–**f** OmpF, or **g**–**i** WEYIPNV. Kernel density estimations (KDEs) of the probability density function of the measured E_FRET_ values are shown as green, red and blue solid lines for **a**, **d**, **g** SurA core-P1, **b**, **e**, **h** core-P2 and **c**, **f**, **i** P1-P2 pairwise measurements in the presence of OmpX, OmpF or peptide WEYIPNV, respectively. Each were fitted to a maximum of two Gaussians to appraise the ensemble heterogeneity (note that these do not necessarily represent true discrete states). Only a single Gaussian was used in (**a**) as this distribution is approximately unimodal. The corresponding apo-SurA distributions (black dashed lines, taken from Fig. 3d–f) are shown for reference to allow comparison between apo and holo SurA. Samples contained ~50 pM labelled SurA variant, 1.5 μM OmpX/OmpF/WEYIPNV, in 50 mM Tris-HCl, pH 8.0, 25 °C, with a final urea concentration of 0.24 M in the OMP-containing samples.

To further study the effects of substrate binding on the conformations of SurA adopted in solution we used smFRET to examine the inter-domain distances of SurA-bound to OmpX, OmpF or WEYIPNV (Fig. 7). Consistent with the HDX data (Fig. 6), binding of OmpX to SurA resulted in changes at the core-P1 interface. Instead of the ca. bimodal E_FRET_ distribution observed for the apo-SurA (E_FRET_ centred on ~0.2 and ~0.6, Fig. 3d), a single broad E_FRET_ distribution with a maximum at an E_FRET_ value (~0.5) between that of the open and closed states was observed for the SurA-OmpX complex (Fig. 7a). By contrast, the E_FRET_ distributions for the core-P2 (Fig. 7b) and P1-P2 (Fig. 7c) SurA variants bound to OmpX were similar to those of apo-SurA. Control experiments in which E_FRET_ was determined for apo-SurA in the presence of 0.24 M urea (used to aid solubilisation of the OMP^50^) showed broadening of the core-P1 distribution (relative to apo-SurA in the absence of urea) (Supplementary Fig. 10a), and little change in the core-P2 and P1-P2 distributions (Supplementary Fig. 10b-c). BVA showed increased dynamics on a sub-ms timescale for all three FRET pairs, suggesting more frequent opening/closing transitions in the presence of denaturant (Supplementary Fig. 10d–f). Effects similar to OmpX binding on the E_FRET_ distributions were observed when OmpF was added to SurA (Fig. 7d–f). In marked contrast with the effects on the E_FRET_ distributions on OmpX/OmpF binding, the addition of the P1-binding peptide WEYIPNV had a more profound effect, changing both the core-P1 and core-P2 inter-domain E_FRET_ distributions, inverting the populations of the core-P1_open_ and core-P1_closed_ distributions to favour core-P1_open_ (Fig. 7g), and shifting the maximum of the core-P2 E_FRET_ distribution to a lower E_FRET_ value (Fig. 7h), with only a subtle change to the P1-P2 E_FRET_ distribution (Fig. 7i). Consistent with the HDX data, these results suggest that binding of WEYIPNV promotes the release of the P1 domain from the core. BVA on the SurA-substrate complexes indicated that all complexes remained dynamic on the sub-millisecond timescale (Supplementary Fig. 11a-i), although the dynamics of the larger OmpF-SurA complex were dampened relative to those of SurA-OmpX (Supplementary Fig. 11d–f).

## Discussion

**Fig. 8:**
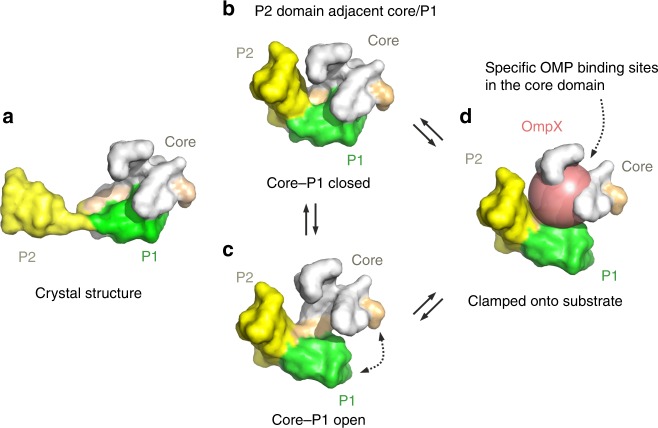
Summary of SurA conformational dynamics and a proposed mechanism of substrate binding. **a** The crystal structure of SurA (PDB 1M5Y^27^). Note that this conformation is not significantly populated in solution, as demonstrated here. Instead, in solution the P2 domain is mostly found close to the core/P1 domains (**b**, **c**). In these conformations the P1 domain can adopt **b** core-P1_closed_ and **c** core-P1_open_ states. **d** Substrate binding results in the P1 domain adopting a structure intermediate between core-P1_open_ and core-P1_closed_. Our data are consistent with the OMP client being captured as a dynamically unfolded state^43^ within a cradle formed by the three domains of SurA. Whether SurA-bound OMP is in a collapsed globule (represented here as a sphere) or a more extended state remains unclear. However, the XL data are consistent with the presence of multiple, specific OMP recognition sites on SurA, suggesting a dynamic ensemble of bound structures. Note that the images presented are schematic and aim to portray the dynamics of the P1, P2 and core domains relative to each other, rather than atomic-level detail.

Despite its key role in OMP biogenesis and bacterial virulence^53,54^, how SurA binds its OMP substrates specifically, but weakly^28,46,51^, and how it is able to protect its clients from aggregation and deliver them to BAM for folding into the OM, remain poorly understood in molecular detail. Previous NMR studies have shown that OmpX, tOmpA and FhuA are dynamically disordered when bound to SurA^42–44^. However, precisely how SurA binds its OMP clients and how OMP binding alters the conformation(s) adopted by SurA in solution have remained unknown. Here, we have exploited XL, HDX-MS, MD and smFRET, to analyse the conformational dynamics of apo-SurA and to investigate how this is modulated by substrate binding. Further, we have identified the regions of SurA involved in substrate binding for both small (OmpX) and larger (OmpF) clients. The combined data presented are consistent with a model in which specific, yet multi-site, binding by a dynamically disordered substrate is accomplished within a cradle-like conformation of SurA that is very different to that observed in its crystal structure (Fig. 8).

Flexibility and/or inter-domain dynamics are key features of the mechanisms of several ATP-independent chaperones, such as Tim9/10^55^, Trigger Factor (TF)^5,56^, Spy^9^, SecB^10^, and the periplasmic OMP chaperones Skp^57–59^ and FkpA^60^. Like SurA, TF and FkpA are multi-domain proteins containing PPIase domains that exhibit inter-domain dynamics^60,61^. The ATP-dependent chaperones GroEL/TRiC, Hsp70 and Hsp90 also utilise inter-domain dynamics for their functional cycles^7,8,62^. For the ATP-independent chaperones HdeA^63^ and Hsp33^64^, which are activated by acidic and oxidative stress, respectively, conformational switching has been shown to trigger their chaperone function. The results presented here suggest that SurA populates a broad ensemble of structures in solution involving different inter-domain distances and orientations. Interconversion between these conformations occurs on a sub-ms timescale, as demonstrated here using smFRET BVA (Fig. 3g–i). We propose that such rapid conformational changes are likely to be important for SurA to be able to bind its clients in the periplasm and to release substrates to BAM for folding into the OM. Notably, a similarly broad range of inter-domain motions on comparable timescales to those observed here for SurA was observed previously in MD simulations for the homologous chaperone TF^5^.

Our combined XL-MS, HDX-MS and smFRET data show that the P1 domain of SurA is not statically bound to the core domain in solution. Instead, these domains are in a dynamic equilibrium between core-P1_open_ and core-P1_closed_ ensembles, suggesting a role for core-P1 dynamics in regulating access of the client OMP to the core chaperone domain and perhaps for access to P1 itself, providing client specificity and enhancing binding affinity^52^. Previous studies revealed that tethering the core and P1 domains by creation of a disulfide bond impairs OMP assembly in vivo, and that destabilising SurA can rescue OMP assembly defects in BAM-compromised strains^29,65^. These results can now be explained by the opening and closing motions between the core and P1 domains revealed here by smFRET and HDX-MS. We also show here that the P2 domain commonly populates conformations in close proximity to the core and P1 domains in solution (Figs. 1, 2) in marked contrast with the orientation of P2 in the SurA crystal structure in which it is distal to P1/core, most likely as a consequence of crystal packing (Supplementary Fig. 2)^27^. This positions the P2 domain such that it could readily provide additional binding/chaperoning capacity for specific substrates^28^. Thus, our data are consistent with SurA frequently adopting a compact, possibly cradle-like, structure (Fig. 8) that may act as the acceptor state for client binding. However, an alternative model in which more extended SurA structures initially capture the unfolded OMP, followed by compaction of the complex, is also possible. In either scenario, the result of binding is a compact SurA in which the client is bound to the core domain, protecting the OMP from aggregation within the dynamic complex. Such a dynamic structure could provide a mechanism for release of bound OMPs to BAM for folding into the OM without the requirement for ATP binding/hydrolysis to drive client release.

Previous reports based on crystal contacts proposed that SurA may bind its substrates via a binding crevice in the N-terminal domain (Fig. 1)^27^. By contrast, the results presented here show that SurA binds its OMP clients at multiple sites^32,66^, predominantly involving the core domain as indicated by tag-transfer XL and HDX. The additional binding capacity in the P1 and P2 domains may be employed in a substrate-specific manner, as suggested by the finding that P2 is required to suppress the aggregation of the 10-stranded OmpT, but not the 8-strand tOmpA^28^. Previous results have also shown that deletion of P2 can perturb SurA function in vivo, as measured by a decrease in the amount of assembled LamB in a BAM-compromised strain^29^. These assembly defects are rescued by further deletion of the P1 domain, suggesting that P2 may also play a role in regulating interactions between the P1 and core domains that inhibit SurA chaperone function^29^. Here we have shown that the three domains of SurA can come into close proximity in solution, consistent with the view that inter-domain communication between all three domains may a play a role in SurA activity. P2 has also been suggested to interact with the BAM complex^67^, to either localise OMPs to this folding catalyst, prime BAM for OMP insertion, promote substrate release, or create supercomplexes linking SecYEG and BAM across the periplasm^68^. Differential proteomics experiments have identified reduced levels of eight different OMPs upon SurA deletion^17^, including OmpX and OmpF which were studied here. Further work will be needed to understand the relay of interactions between SurA, its clients, other chaperones, and BAM, and to discern whether/how the mechanism of OMP delivery to the OM is dependent on the identity of the OMP client.

The XL-MS data presented show that OmpX is able to adopt multiple conformations and orientations when bound to SurA^42^. The cross-link locations identified on SurA by tag-transfer experiments show no clear correlation with areas with a particular electrostatic potential, hydrophobic patches, or regions of high sequence conservation (Supplementary Fig. 12). Instead, they cluster to regions within the cradle which forms by docking of the three domains of SurA and in which the OMP is sequestered and binds predominantly to the core domain (Fig. 8). Such a model is consistent with in vivo data showing that the SurA core domain alone can largely (but not wholly) complement deletion of wild-type SurA^24,29,69^. Specific client interaction sites have also been identified in TF using the substrate PhoA, which are also located in a cradle formed between its domains^70^. In the presence of substrate, the E_FRET_ distribution observed by smFRET shifts to an intermediate state between core-P1_open_ and core-P1_closed_, suggesting that substrate binding either alters the conformations of SurA, or that the interconversion between the open and closed states occurs more rapidly than in the unbound state, leading to signal averaging. In either case, this is reminiscent of the lid motions in Hsp70 that entrap its substrates^71^, the sequestration of OMPs within the cavity of the chaperone Skp^57,58^, and the conformational flexibility that has been suggested to be important for substrate capture/release by Spy^9^. The conformational properties of OmpX when bound to SurA cannot be resolved in detail using the data presented here. Interestingly, however, analysis of the locations of cross-linking sites on OmpX in the OmpX-SurA complex using DSBU does not show any obvious correlation with regions that form β-strands, loops or turns in the folded OMP, consistent with the tag-transfer results which showed cross-linking to SurA when the diazirine was positioned in regions that either form β-strands or loops in native OmpX (Fig. 5a, Supplementary Fig. 13). Combined, these results suggest that while specific SurA recognition motifs in OMP sequences may be present^19,28,45,51^, the interaction interface on SurA may be less well-defined, consistent with recent reports from Marx et al. who also used XL as well as neutron diffraction to examine SurA-client interactions^32^.

A number of sequence motifs in OMPs have been implicated in facilitating OMP biogenesis. In the case of OmpX, such motifs have been shown to nucleate assembly^72^, mediate hydrophobic collapse and membrane interactions^73^, and stabilise the folded state^74^. Aromatic-containing motifs in OMPs (Ar-Ar and Ar-X-Ar, where Ar is an aromatic residue and X is any amino acid) have also been implicated in OMP-SurA interactions^19,32,45,51^. Many of the cross-links we detect are close to these motifs (Supplementary Fig. 13). Available evidence suggests that when bound to SurA, OMPs adopt extended structures^32,33^, and that binding of multiple SurA molecules to different sites in the OMP may be essential to prevent aggregation from these extended states^28,32,46^.

The in vitro folding pathways of several OMPs have been explored^75,76^, however, how OMP folding is modulated by chaperone binding^28,46,50,58,75^ and interactions with BAM remain unresolved^77,78^. Once translocated into the periplasm by the SecYEG complex, OMP folding, comprising delivery to BAM and insertion into the OM, is achieved without an external energy source. Indeed, this process is thought to be driven thermodynamically by favourable free energies of folding of OMPs (Δ*G*°_F_ as high as ~130 kJ/mol^79^) compared with the weak binding of OMPs to SurA (Δ*G*° ~ 35-44 kJ/mol^80^) or their self-association (Δ*G*° ~ 38 kJ/mol^80–82^). The weak binding events between OMPs and SurA are likely key to enable efficient transfer of OMPs between chaperone molecules and for handover to the BAM complex^78^. It has been proposed that unfolded OMPs are recognised by BAM via the so-called C-terminal “β-signal”^83,84^. Our XL-MS experiments suggest that this region of OmpX forms contacts with SurA (Supplementary Fig. 13). Hence, the transient nature of the SurA-OMP interactions may be essential both to facilitate presentation of the β-signal to BAM and, subsequently, to enable rapid handover of OMPs to BAM for folding and insertion into the OM. Recent studies using neutron diffraction and cross-linking support this model^32^.

SurA plays multiple roles in OMP biogenesis, including sequestration of OMPs in the periplasm to prevent their toxic aggregation^85^ and delivery of OMPs to the BAM complex to enable folding into the OM^78^. The results presented here demonstrate a role for SurA inter-domain dynamics in OMP binding, notably the reorganisation of the core and P1 domains, and dynamic localisation of P2 close to these domains. Such structural plasticity may also be important for facilitating binding of SurA:OMP complexes to BAM, assisting BAM catalytic activity, or priming the OMP for membrane insertion by pre-selecting favourable conformations for folding, thereby smoothing the energy landscape of folding^33,44,78^. Understanding the interplay between the conformational dynamics of SurA and those of BAM, in particular the communication and coordination between SurA and different BAM subunits, will be essential in unravelling the molecular mechanism of OMP biogenesis. The model of SurA action presented here, whereby a compact, dynamic and responsive chaperone structure is responsible for client binding, represents a first key step in this endeavour. This adds to the growing body of data suggesting that all components of the OMP assembly line, including SurA, Skp^57,58^ and BAM^86–90^ have intrinsic conformational dynamics which, in combination, may be key to achieving efficient OMP biogenesis in the absence of ATP.

## Methods

### Cloning, expression and purification of SurA

A pET28b plasmid containing the mature SurA sequence preceded by an N-terminal 6x His-tag and thrombin-cleavage site (pSK257) was a kind gift from Daniel Kahne (Harvard University, USA)^91^. The thrombin-cleavage site was mutated to a TEV-cleavage site using Q5 site-directed mutagenesis (NEB), and the resulting plasmid was transformed into BL21(DE3) cells (Stratagene). Cells were grown in LB medium supplemented with 30 µg/mL kanamycin at 37 °C with shaking (200 rpm) until an OD_600_ of ~0.6 was reached. The temperature was subsequently lowered to 20 °C, and expression induced with 0.4 mM IPTG. After ~18 h, cells were harvested by centrifugation, resuspended in 25 mM Tris-HCl, pH 7.2, 150 mM NaCl, 20 mM imidazole, containing EDTA-free protease inhibitor tablets (Roche), and lysed using a cell disrupter (Constant Cell Disruption Systems). The cell debris was removed by centrifugation (20 min, 4 °C, 39,000 × *g*), and the lysate was applied to 5 mL HisTrap columns (GE Healthcare). The columns were washed with 25 mM Tris-HCl, pH 7.2, 150 mM NaCl and 20 mM imidazole, followed by 25 mM Tris-HCl, 6 M Gdn-HCl, pH 7.2 (to denature the SurA on-column). After washing with 25 mM Tris-HCl, 150 mM NaCl, pH 7.2, SurA was eluted with 25 mM Tris-HCl, 150 mM NaCl, 500 mM imidazole, pH 7.2. The eluate was dialysed against 25 mM Tris-HCl, 150 mM NaCl, pH 8.0 overnight, and the following day TEV protease^46^ (ca. 0.5 mg) and 0.1% (v/v) β-mercaptoethanol were added. The cleavage reaction was left to proceed overnight at 4 °C on a tube roller. The cleavage reaction was again applied to the 5-mL HisTrap columns (GE Healthcare) to remove the cleaved His-tag and His-tagged TEV protease. The unbound, cleaved SurA product was dialysed extensively against 25 mM Tris-HCl, 150 mM NaCl, pH 8.0, before being concentrated to ~200 µM with Vivaspin 20 concentrators (Sartorius; 5-kDa MWCO), aliquoted, snap-frozen in liquid nitrogen and stored at −80 °C. Protein concentrations were determined spectrophotometrically using an extinction coefficient at 280 nm of 29450 M^−1^ cm^−1^.

Cys-containing variants (Q85C, N193C, E301C, Q85C-N193C, N193C-E301C and Q85C-E301C) were generated by Q5 site-directed mutagenesis (NEB) and were purified as detailed above, except for the addition of 10 mM DTT to all buffers in the purification procedure, up until the elution step.

### Expression and purification of TEV protease

Vector pMHTDelta238 containing His-tagged TEV fused with MBP which is removed in vivo by autocleavage, was obtained from DNASU (Clone TvCD00084286). The vector was transformed into BL21-CodonPlus[DE3]-RIPL cells (Stratagene, UK). Cells were grown in LB medium containing 50 µg/mL kanamycin at 37 °C with shaking (200 rpm) until the culture reached an OD_600_ of ~0.6. The temperature was then lowered to 30 °C and expression induced with 0.5 mM IPTG. After ~4 h the cells were harvested by centrifugation, resuspended in 25 mM sodium phosphate buffer, pH 8.0, 200 mM NaCl, 10% (v/v) glycerol, 25 mM imidazole, 1 mM PMSF, 2 mM benzamidine, ~0.02 mg/ml DNase (Sigma, UK), and lysed by sonication (6 × 30 s bursts with 1 min cooling on ice between each sonication). The lysate was centrifuged to remove cell debris (20 min, 4 °C, 39,000 × *g*), applied to Ni^2+^ Sepharose beads (GE Healthcare) and washed twice with 25 mM sodium phosphate buffer, pH 8.0, 200 mM NaCl, 10% (v/v) glycerol, 25 mM imidazole. His-tagged TEV was eluted with 25 mM sodium phosphate buffer, pH 8.0, 200 mM NaCl, 10% (v/v) glycerol, 500 mM imidazole. The eluate was filtered (0.2 μM syringe filter, Sartorius, UK) and gel filtered on a HiLoad Superdex 75 26/60 column (GE Healthcare) equilibrated with 25 mM sodium phosphate buffer, pH 8.0, 200 mM NaCl, 25 mM imidazole, 10% (v/v) glycerol, 5 mM β-mercaptoethanol. Peak fractions were concentrated to ~1 mg/mL using Vivaspin 20 (5 kDa MWCO) concentrators (Sartorius), aliquoted, snap-frozen in liquid nitrogen and stored at −80 ^°^C.

### Cloning of OmpX, OmpF and Cys-OmpX

Codon-optimised synthetic genes (Eurofins) of the mature sequences of OmpX (residues 24–171) and OmpF (residues 23–362) were cloned into pET11a (Novagen) between the NdeI (5′) and BamHI (3′) restriction sites. To create the Cys-OmpX construct, the residues Gly-Ser-Cys were added immediately after the N-terminal Met residue using Q5 site-directed mutagenesis (NEB).

### Expression and purification of OMPs

OMPs were purified using a method adapted from ref. ^50^. Briefly, *E. coli* BL21[DE3] cells (Stratagene) were transformed with a pET11a plasmid containing the gene sequence of the mature OMP. Overnight cultures were subcultured and grown in LB medium (500 mL) supplemented with carbenicillin (100 μg/mL), at 37 °C with shaking (200 rpm). Protein expression was induced with IPTG (1 mM) once an OD_600_ of 0.6 was reached. After 4 h the cells were harvested by centrifugation (5000 × *g*, 15 min, 4 °C). The cell pellet was resuspended in 50 mM Tris-HCl pH 8.0, 5 mM EDTA, 1 mM phenylmethylsulfonyl fluoride, 2 mM benzamidine, and the cells were subsequently lysed by sonication. The lysate was centrifuged (25,000 × *g*, 30 min, 4 °C) and the insoluble material was resuspended in 50 mM Tris-HCl pH 8.0, 2% (v/v) Triton X-100, before being incubated for 1 h at room temperature, with gentle agitation. The insoluble material was pelleted (25,000 × *g*, 30 min, 4 °C) and the inclusion bodies washed twice by resuspending in 50 mM Tris-HCl pH 8.0 followed by incubation for 1 h at room temperature with gentle agitation, and then collected by centrifugation (25,000 × *g*, 30 min, 4 °C). For the OmpX and OmpF constructs, the inclusion bodies were solubilised in 25 mM Tris-HCl, 6 M Gdn-HCl, pH 8.0 and centrifuged (20,000 × *g*, 20 min, 4 °C). The supernatant was filtered (0.2 µM syringe filter, Sartorius) and the protein was purified using a Superdex 75 HiLoad 26/60 gel filtration column (GE Healthcare) equilibrated with 25 mM Tris-HCl, 6 M Gdn-HCl, pH 8.0. Peak fractions were concentrated to ∼500 μM using Vivaspin 20 (5 kDa MWCO) concentrators (Sartorius), and the protein solution was snap-frozen in liquid nitrogen and stored at −80 °C.

### Chemical cross-linking-mass spectrometry (XL-MS)

OmpX was buffer exchanged from storage buffer (25 mM Tris-HCl, 6 M Gdn-HCl, pH 8.0) into 10 mM potassium phosphate, pH 8.0, 8 M urea using Zeba spin desalting columns (Thermo Fisher Scientific). For cross-linking, apo-SurA was prepared at a concentration of 5 µM in 10 mM potassium phosphate, pH 8.0, while SurA-OmpX complexes were assembled by mixing SurA and OmpX such that the final concentration of each was 5 µM, in 10 mM potassium phosphate, pH 8.0, 0.24 M urea. DSBU (Thermo Fisher Scientific) was added at 10× (apo-SurA) or 10–400× (SurA:OmpX) molar equivalents relative to the concentration of SurA, and the cross-linking reaction was left to proceed for 45 min at room temperature before quenching by adding 0.2 M Tris-HCl, pH 8.0. The cross-linked material was separated by SDS-PAGE and gel bands corresponding to either SurA alone or the SurA:OmpX complex were excised and the proteins trypsinised in-gel^48^. Briefly, gel bands were cut into ca. 1-mm^3^ pieces, and the pieces were destained in 30% (v/v) ethanol at 60 °C for 30 min, dehydrated with 100% acetonitrile, and dried in a laminar flow hood for 60 min. The gel pieces were rehydrated with 20 µL of 0.02 μg.μL^−1^ trypsin solution (Promega) in 25 mM ammonium bicarbonate pH 8, and incubated at 37 °C for 18 h with shaking (500 rpm). Peptides were recovered by incubating gel pieces with 50 μL of 60% (v/v) acetonitrile/5% (v/v) formic acid (×3) for 10 min. The peptides were then evaporated to dryness and resuspended in 20 μL with 5% (v/v) acetonitrile/0.1% (v/v) formic acid prior to MS analysis. Peptides (5 µL) were injected onto a reverse-phase Acquity M-Class C18, 75 µm × 150 mm column (Waters) and separated by gradient elution of 1–50% (v/v) solvent B (0.1% (v/v) formic acid in acetonitrile) in solvent A (0.1% (v/v) formic acid in water) over 60 min at 300 nL.min^−1^. The eluate was infused into an Orbitrap Q Exactive (Thermo Fisher Scientific) mass spectrometer operating in positive ion mode. Orbitrap calibration was performed using Ultramark solution (Thermo Fisher Scientific). Data acquisition was performed in DDA mode and fragmentation was performed using HCD. Each high-resolution full scan (*m*/*z* range 500–2000, *R* = 120,000) was followed by high-resolution product ion scans (*R* = 15,000), with a normalised collision energy of 30%. The 15 most intense ions in the MS spectrum were selected for MS/MS. Dynamic exclusion of 60 s was used. RAW data files were converted to MGF format using PEAKS Studio 8.5 (Bioinformatics Solutions). Cross-link identification was performed using MeroX v1.6.6^92^. Data were processed using RISE mode. Automated cross-link assignment was performed considering Lys-Lys cross-links and allowing a maximum of 2 of the four 26-u doublets, corresponding to fragmentation of the cross-linker, to be missing. Up to two oxidised Met residues per peptide were considered as variable modifications. Mass deviation tolerances of up to 10 ppm in both MS and MS/MS were used. Only results with scores corresponding to a false discovery rate (FDR) of <5% were taken forward. All spectra were manually verified to ensure they comprised fragment ions with significant coverage of each cross-linked peptide and for the presence of diagnostic fragments from cross-linker fragmentation. A representative mass spectrum for each cross-link can be found in Supplementary Data 1. The Raw DSBU XL-MS data have been deposited to the ProteomeXchange Consortium via the PRIDE^93^ partner repository with the dataset identifier PXD016993. A reporting summary (based on community guidelines^94^) can be found (Supplementary Data 2).

### Preparation of SurA variants for smFRET

For each SurA variant, the protein was diluted to a concentration of 50 μM in 25 mM Tris-HCl, pH 7.2, 150 mM NaCl, 5 mM DTT. The protein solution was incubated for 30 min at room temperature before being buffer exchanged into 25 mM Tris-HCl, pH 7.2, 150 mM NaCl, 1 mM EDTA using 7 kDa MWCO Zeba spin desalting columns (Thermo Fisher Scientific). A ten-fold molar excess of Alexa Fluor 488 C5 maleimide/Alexa Fluor C5 594 maleimide (Thermo Fisher Scientific) was then added and the samples incubated for 2 h at room temperature with gentle rocking. The reaction was quenched with a 10-fold molar excess (over Alexa Fluor 488 C5 maleimide and Alexa Fluor C5 594 maleimide) of β-mercaptoethanol. Protein was separated from unbound dye by size exclusion chromatography on a Superdex 200 10/300 GL column (GE Healthcare, UK) equilibrated with 50 mM Tris-HCl, 150 mM NaCl, pH 8.0. Fractions containing labelled protein were combined, snap-frozen in liquid nitrogen and stored at −80 °C.

### Single-molecule Förster resonance energy transfer (smFRET)

smFRET experiments were performed using a custom-built experimental set-up for μs ALEX^95^. Laser wavelengths and powers used were 488 nm, 140 μW and 594 nm, 120 μW, respectively before the objective and losses from the objective were on the order of 50%. The laser alternation period was set to 40 μs (duty cycle of 40%). Samples of labelled SurA were prepared on the day of use from concentrated stocks that had been stored at −80 °C and were kept on ice and in the dark while in use. A sample (100 µL, 50 mM Tris-HCl pH 8.0, 50 pM of labelled SurA) selectively supplemented with 1.5 μM OmpX/OmpF and/or 0.24 M urea or 1.5 μM peptide was added atop a coverslip set on the objective. A camera was used to monitor the distance of the focal plane from the coverslip and the objective height adjusted using a piezo-controller (piezo system jena) to 20 μm above the surface of the coverslip. Data acquisition was performed in 3 × 10 min runs with fresh sample prepared after every third collection to counteract the issues of protein aggregation and adherence to the coverslip as well as changes in solution osmolarity resulting from evaporation. Evaporation over the course of 30 min was minimised by employing a plastic lid that fitted over the coverslip. Data were collected using Labview graphical environment (LabView 7.1 Professional Development System for Windows, National Instruments)^96^. Separate photon streams were then converted and stored in an open file format for timestamp-based single-molecule fluorescence experiments (Photon-HDF5), which is compatible with many recent data processing environments^97^. Fluorescence bursts were analysed using customised Python 2.7 scripts^98^, and made use of FRETBursts, an open source toolkit for analysis of freely-diffusing single-molecule FRET bursts^99^. Functions from the FRETBursts package were used to recover single-molecule ‘bursts’ of fluorescence containing a minimum of 20 photons and being 1.7 times higher than the background signal of the measured time traces. Artefacts due to photophysical effects such as blinking were also removed. Apparent E and S values were calculated by relating the measured intensities in each of the four photon streams within each burst, i.e.,

1. donor emission during periods of donor excitation $(I_{Dem|Dex})$,
2. donor emission during periods of accpetor excitation $(I_{Dem|Aex})$,
3. acceptor emission during periods of donor excitation $(I_{Aem|Dex})$,
4. acceptor emission during periods of acceptor excitation $(I_{Aem|Aex})$,

according to Eqs. (1) and (2), respectively.

1 $$^iE_{app} = \frac{{I_{Aem|Dex}}}{{I_{Aem|Dex} + I_{Dem|Dex}}}$$

2 $$^iS_{app} = \frac{{I_{Aem|Dex} + I_{Dem|Dex}}}{{I_{Aem|Dex} + I_{Dem|Dex} + I_{Aem|Aex}}}$$

Four correction parameters described by relations between the photon streams were determined and applied to the data during the burst search algorithm of FRETBursts:

1. Donor leakage into the acceptor channel
  3 $$\alpha = \frac{{g_{R|D}}}{{g_{G|D}}} = \frac{{\left( {{\,}^{ii}E_{app}^{({\mathrm{DO}})}} \right)}}{{1 - \left( {{\,}^{ii}E_{app}^{({\mathrm{DO}})}} \right)}}$$
2. Excitation of the acceptor dye by the donor excitation laser
  4 $$\delta = \frac{{\sigma _{A|G}}}{{\sigma _{A|R}}}\frac{{I_{Dex}}}{{I_{Aex}}} = \frac{\left({{\,}^{ii}S_{app}^{({\mathrm{AO}})}}\right)}{{1 - \left( {{\,}^{ii}ES_{app}^{({\mathrm{AO}})}} \right)}}$$
3. Normalization of effective fluorescence quantum yields, ${}^{eff}{\mathrm{\Phi }}_F = a_b.{\mathrm{\Phi }}_F$ and detection efficiencies,$g$, of $A$ and $D.$
  5 $$\gamma = \frac{{g_{R|A}}}{{g_{G|D}}}\frac{{{\,}^{eff}_{\Phi _{F,A}}}}{{{\,}^{eff}_{\Phi _{F,D}}}}$$
  *a*_*b*_ is the fraction of molecules in the bright state and ${\mathrm{\Phi }}_F$ is the fluorescence quantum yield without photophysical (saturation) effects.
4. Normalization of excitation intensities,$I$, and cross-sections,$\sigma$, of *A* and *D*.
  6 $$\beta = \frac{{\sigma _{A|R}}}{{\sigma _{D|G}}}\frac{{I_{Aex}}}{{I_{Dex}}}$$
  according to the standard FRET workflow developed by Hellenkamp et al.^100^, employing the following definitions:

FRET efficiency:

7 $$E\,{\mathrm{or}}\,E_{{\mathrm{FRET}}} = \frac{{F_{A|D}}}{{F_{D|D} + F_{A|D}}}$$

Stoichiometry:

8 $$S\,{\mathrm{or}}\,S_{{\mathrm{FRET}}} = \frac{{F_{D|D} + F_{A|D}}}{{F_{D|D} + F_{A|D} + F_{A|A}}}$$

Subscripts:

$D\,{\mathrm{or}}\,A$ – Concerning donor or acceptor

$A|D$ – Acceptor fluorescence given donor excitation

$A_{em}|D_{ex}$, $D_{em}|D_{ex}$, $A_{em}|A_{ex}$ – Intensity in the acceptor channel given donor excitation, accordingly

Superscripts:

$${\mathrm{BG}}$$ – Background

${\mathrm{DO}}/{\mathrm{AO}}$ – Donor-only/acceptor-only species

$i - iii$ – Indicates (i) the uncorrected intensity; (ii) intensity after BG correction; (iii) intensity after BG, alpha and delta corrections.

The data from each 10-min acquisition was merged prior to subsequent analysis. In order to remove bursts arising from singly labelled proteins, the data were also further filtered using ALEX-2CDE, yielding bursts with a Gaussian distribution of *S* values in a narrow range of dye stoichiometry (*S* within 0.25–0.75)^101^. Typically, ~5000 bursts were collected for each condition examined after all filters had been applied. The result of this procedure of correction and filtering is demonstrated graphically in the form of 2D plots of E versus S for the example of the core-P1 labelled SurA which also served as our etalon (Supplementary Fig. 14). Filtered bursts were then assembled into 1D histograms and kernel density estimation was used to approximate 1D probability density functions of the E_FRET_ values in each condition which were then fitted to up to two Gaussians. Burst variance analysis (BVA)^41^ was performed using FRETBursts^98^, and plots were made using the Seaborn and Matplotlib^102^ packages in the Spyder IDE on python 3.7. Visualisations of the available volumes for FRET dyes attached at different positions in SurA were generated using the FRET Positioning and Screening (FPS) software with dye linker lengths and radii parameters suggested in the FPS manual for the FRET dyes used^39^. Predicted E_FRET_ value distributions from the crystal structure of full-length SurA for each dye pair were calculated from distance distributions generated using the MtsslWizard PyMOL plugin^103^. Raw smFRET data are available at the University of Leeds data repository (10.5518/701). Burst variance analysis (BVA)^41^ was performed using FRETBursts^98^, and plots were made using the Seaborn and Matplotlib^102^ packages. Visualisations of the available volumes for FRET dyes attached at different positions in SurA were generated using the FRET Positioning and Screening (FPS) software with dye linker lengths and radii parameters suggested in the FPS manual for the FRET dyes used^39^. Predicted E_FRET_ value distributions from the crystal structure of full-length SurA for each dye pair were calculated from distance distributions generated using the MtsslWizard PyMOL plugin^103^. Raw smFRET data are available at the University of Leeds data repository (10.5518/701).

### Fluorescence anisotropy

Fluorescence anisotropy decay measurements were performed on single Cys variants of SurA with/without OmpX (600 nM SurA with/without 3 µM OmpX), with SurA labelled with Alexa Fluor 488 C5 maleimide or Alexa Fluor C5 594 using a Quantamaster 8000 (Horiba) equipped with a Whitelase supercontinuum pulsed laser (NKT) for excitation with a repetition rate of 10 MHz and TCSPC detection. Three pairs of scans were taken with VV and VH polarisation for each sample (500 µL, 600 nM in 50 mM Tris-HCl pH 8), and a peak height of 10,000 photons was collected for each scan. Normalisation and global fitting of each pair of polarised decay curves along with the IRF and HV and HH polarised decays that defined the G factor was performed using FelixGX v4.9.0.10243 (Horiba). The steady state and time-resolved anisotropy are related by the following expression (9):

9 $$r = \frac{{r_0}}{{1 + {t /{T_r}}}}$$

where *r* is the steady state anisotropy, *r*_0_ is the initial anisotropy, *τ* is the fluorescence lifetime and *T*_*r*_ is the rotational correlation timecalculated from the measured decay,

### Molecular dynamics simulations

All-atom molecular dynamics simulations of the mature sequence of SurA (residues 21–428) in explicit solvent were performed with GROMACS 5.0.2^104^ using the CHARMM36 force field^105^. For simulations starting from the crystal structure of full-length SurA (PDB 1M5Y^27^), loop residues which are unresolved in the structure were modelled using MODELLER^106^, and the four missing N-terminal residues were added in Chimera^107^. The system was minimised (5000 steps) followed by equilibration for 25 ps, with backbone and sidechain position restraints of 400 and 40 kJ mol^−1^ nm^−2^, respectively, in the *x*, *y* and *z* directions. The temperature reached its target value (300 K) within the first 10 ps and remained stable for the rest of the equilibration. The system contained 202 sodium ions and 198 chloride ions (150 mM NaCl), and 70,091 TIP3P water molecules. The total number of atoms was 217,001 in a periodic box size of 13.2 nm × 13.2 nm × 13.2 nm. For simulations starting from a SurA^core-P1-open^ conformation a model was first built using the crystal structures of full-length SurA (PDB 1M5Y^27^) and SurA-ΔP2 (PDB 2PV3^52^), in which the P1 domain is extended away from the core. The two structures were aligned on the core domain and the P1 and core domains were removed from the full-length SurA structure. Linker residues between domains were added using MODELLER^106^, and the four missing N-terminal residues were added in Chimera. The system was minimised (5000 steps) followed by equilibration for 25 ps with backbone and sidechain position restraints of 400 and 40 kJ mol^−1^ nm^−2^, respectively, in the *x*, *y* and *z* directions. The temperature reached its target value (300 K) within the first 10 ps and remained stable for the rest of the equilibration. The system contained 189 sodium ions and 185 chloride ions (150 mM NaCl), and 64,809 TIP3P water molecules. The total number of atoms was 201,129 in a periodic box size of 12.9 nm × 12.9 nm × 12.9 nm. Simulation systems were built using CHARMM-GUI^108^. In all simulations the pressure was maintained using a Parrinello-Rahman barostat^109^ and the temperature was maintained using a Nose-Hoover thermostat^110^. The temperature of the systems was 300 K and the timestep was 2 fs. Analysis of Cα–Cα distances between residue pairs identified in cross-linking experiments was performed using the ‘*gmx distance*’ GROMACS command. Calculations of solvent accessible surface distances (SASDs) made use of JWalk^35^. MD simulation data, including those starting from the SurA crystal structure, are available at the University of Leeds data repository (10.5518/701). Included are GROMACS input files, starting structures, reduced MD trajectories and the final structures after 1 μs of simulation.

### Simulated annealing

Simulated annealing calculations were carried out in XPLOR-NIH^111^. Cross-links were treated as distance restraints with a flat-well energy potential using noePot. A rigid-body calculation (100 calculations in total) was performed, where each domain was treated as a rigid body and residues in the linker regions were given torsion angle degrees of freedom. To ensure that the initial parameters used do not bias the outcome, the starting structures used in each of the 100 simulations were all different, generated by randomly orienting the domains relative to each other. Pseudo-potential energy terms describing covalent geometry restraints were applied to restrict deviation from bond lengths, angles and improper torsion angles. All cross-links were utilised as distance restraints in the 100 calculations. The first step in the structure calculation consisted of 10,000 steps of energy minimization, followed by simulated annealing dynamics with all the potential terms active, where the temperature is slowly decreased (3000–25 K) over 4 fs and a final energy minimization in torsion angle space. During the hot phase (*T* = 3000 K) the cross-link terms were underweighted to allow the domain to sample a large conformational space and they were geometrically increased during the cooling phase. For each calculation the coordinates of P1 and P2 were randomized by applying a random translation within 20 Å and a random rotation within 90° of their initial positions. The linkers were re-built using torsionDB^112^ to enforce correct geometry before the first step in the structure calculation protocol. The 100 generated structures were ranked based on their energies, taking into account how well the distance restraints are satisfied (this is the main contributor to the final energy) and covalent geometry/VDW terms to ensure that the selected models do not have any geometry violations. The 10 lowest energy structures were visualised and analysed in further detail. Given this simulated annealing approach will drive the structure to compact states, more extended states of SurA that smFRET data show are populated in solution, will not be captured by this method. The structures of the 10 lowest energy conformations of SurA are available at the University of Leeds data repository (10.5518/701).

### Labelling of Cys-OmpX with Alexa Fluor 488

Purified Cys-OmpX was covalently labelled with Alexa Fluor 488 dye via maleimide chemistry. A sample containing 200 μM Cys-OmpX in 25 mM Tris-HCl, 6 M Gdn-HCl, pH 7.2, was incubated with 10 mM DTT for 30 min. This sample was subsequently buffer exchanged into 25 mM Tris-HCl, 6 M Gdn-HCl, pH 7.2 (that had been sparged for 15 min with nitrogen gas) using Zeba spin desalting columns (Thermo Fisher Scientific). Alexa Fluor 488 C5 maleimide (Thermo Fisher Scientific) (10 mg/mL dissolved in DMSO) was immediately added to the OmpX sample at a final concentration of 2 mM. The total sample volume was 480 µL. The labelling reaction was kept at 25 °C for 1 h then left overnight at 4 °C. The reaction was then loaded onto a Superdex Peptide 10/300 column (GE Healthcare) equilibrated with 6 M Gdn-HCl, 25 mM Tris-HCl, pH 7.2 to remove the excess free dye. Samples were collected every 1 mL and peak protein fractions tested for dye labelling using a Nanodrop 2000 (Thermo Fisher Scientific). Samples containing labelled OmpX were snap-frozen using liquid nitrogen and stored at −80 °C until required.

### Microscale thermophoresis (MST)

From a 200 μM SurA stock solution in 50 mM Tris-HCl, pH 8.0, a series of twofold serial dilutions was performed to obtain 16 15-µL samples. Labelled Cys-OmpX was buffer exchanged into 8 M urea, 50 mM Tris-HCl, pH 8.0, to a concentration of 1.7 µM. This stock was diluted 16.6-fold to a concentration of 100 nM with 50 mM Tris-HCl, pH 8.0, then immediately added to the sixteen SurA-containing samples in 15 µL aliquots (30 µL total sample volume). The final sample concentrations were 50 nM Cys-OmpX, 100 µM–3 nM SurA, 0.24 M urea, 50 mM Tris-HCl, pH 8.0. Samples were immediately added to capillaries by capillary action then read using a Monolith NT.115 MST instrument (NanoTemper, Germany). To obtain the dissociation constant, *K*_d_, data were fitted to the Hill Eq. (10):

10 $$S_{{\mathrm{obs}}} = S_{\mathrm{U}} + \left( {S_{\mathrm{B}} - S_{\mathrm{U}}} \right).\left( {\frac{{[{\mathrm{SurA}}]^n}}{{K_{\mathrm{D}} + [{\mathrm{SurA}}]^n}}} \right)$$

where *S*_obs_ is the observed signal, *S*_U_ is the signal from unbound OmpX, *S*_B_ is the signal from bound OmpX, and *n* is the Hill coefficient. Data fitting was carried out using IgorPro 6.3.4.1 (Wavemetrics, Oregon, USA).

### Tag-transfer photo-cross-linking

Single Cys variants of OmpX (M41C, I102C, K122C, V167C) were conjugated with MTS-diazirine^48^. Briefly, Each OmpX variant was buffer exchanged into 6 M guanidine-HCl, 50 mM Tris-HCl, 10 mM DTT, 1 mM EDTA, pH 8.0 that had been sparged with N_2_. After incubation at room temperature for 15 min, the OmpX variant was buffer exchanged (Zeba Spin Desalting Columns, 7 K MWCO, Thermo Scientific) into the same buffer without DTT. MTS-diazirine (from a stock solution in DMSO) was added in 20-times molar excess over the OmpX variant. Final concentrations were 200 µM OmpX, 4 mM MTS-diazirine, 20% (v/v) DMSO, 4.8 M guanidine-HCl, 40 mM Tris-HCl and 0.8 mM EDTA. This solution was incubated at room temperature for 1 h before being buffer exchanged into 6 M guanidine-HCl, 50 mM Tris-HCl, 1 mM EDTA, pH 8.0. The labelled protein was aliquoted, snap-frozen in liquid nitrogen and stored at −80 °C.

SurA-OmpX complexes were assembled by mixing SurA with each OmpX variant such that the final concentrations of each was 10 µM and 5 µM, respectively, in 10 mM potassium phosphate buffer, pH 8.0, 0.24 M urea. Photo-cross-linking was performed for 30 s using a UV LED irradiation platform^48^. The cross-linked material was separated by SDS-PAGE. The gel band corresponding to the cross-linked complex was excised and the proteins were trypsinised in-gel (see XL-MS methods above)^48^. To detect additional modified peptides, reduction of the cross-linker and thiol capture was performed to enrich cross-linked peptides. Peptides (5 µL) were injected onto a reverse-phase Acquity M-Class C18, 75 µm × 150 mm column (Waters) and separated by gradient elution of 1–50% (v/v) solvent B (0.1% (v/v) formic acid in acetonitrile) in solvent A (0.1% (v/v) formic acid in water) over 60 min at 300 nL.min^−1^. The eluate was infused into a Xevo G2-XS (Waters) mass spectrometer operating in positive ion mode. Mass calibration was performed by infusion of aqueous NaI (2 µg/µL). [Glu1]-Fibrinopeptide B (GluFib) was used for the lock mass spray, with a 0.5 s lock spray scan taken every 30 s. The lock mass correction factor was determined by averaging 10 scans. Data acquisition was performed in DDA mode with a 1 s MS scan over *m*/*z* 350–2000. The four most intense ions in the MS spectrum were selected for MS/MS by CID, each with a 0.5 s scan over *m*/*z* 50–2000. The collision energy applied was dependent upon the charge and mass of the selected ion. Dynamic exclusion of 60 s was used. Data processing and modification localization was performed using PEAKS Studio 8.5 (Bioinformatics Solutions). Search parameters were as follows: parent mass error tolerance = 10 ppm; fragment mass error tolerance = 0.05 Da, maximum number of missed cleavages = 3; fixed modification = carbamidomethylation (57.02 Da); variable modifications = deamidation (0.98 Da), oxidation of Met (15.99 Da), MTS tag (145.06 Da). A FDR cut-off of 1% was used. A representative mass spectrum for each cross-link can be found in Supplementary Data 1. The raw tag-transfer XL data have been deposited to the ProteomeXchange Consortium via the PRIDE^93^ partner repository with the dataset identifier PXD016993.

### Hydrogen-deuterium exchange mass spectrometry

An automated HDX robot (LEAP Technologies, Ft Lauderdale, FL, USA) coupled to a Acquity M-Class LC and HDX manager (Waters, UK) was used for all HDX-MS experiments. For differential HDX-MS of SurA in the absence and presence of OmpX/OmpF, samples contained 8 µM of SurA or 8 µM of SurA with 8 µM OmpX/OmpF (in 10 mM potassium phosphate, pH 8.0, 0.24 M urea). For differential experiments with addition of WEYIPNV peptide, the samples contained 8 µM SurA with 110 µM peptide (in 10 mM potassium phosphate, pH 8.0). Note that the addition of 0.24 M urea does not dramatically alter the intrinsic rate of exchange^113^.

Thirty microlitres of protein-containing solution was added to 135 μL deuterated buffer (10 mM potassium phosphate buffer pD 8.0, 0.24 M d_4_-urea or 10 mM potassium phosphate buffer pD 8.0, 82% D_2_O) and incubated at 4 °C for 0.5, 2, 30 or 120 min. Four replicate measurements were performed for each condition and each time point. After labelling, HDX was quenched by adding 100 μL of quench buffer (10 mM potassium phosphate, 2 M Gdn-HCl, pH 2.2) to 50 μL of the labelling reaction. Fifty microlitres of the quenched sample was passed through immobilised pepsin and aspergillopepsin columns (Affipro, Mratín, Czech Republic) connected in series (20 °C) and the peptides were trapped on a VanGuard Pre-column [Acquity UPLC BEH C18 (1.7 μm, 2.1 mm × 5 mm, Waters, UK)] for 3 min. The peptides were separated using a C18 column (75 μm × 150 mm, Waters, UK) by gradient elution of 0–40% (v/v) acetonitrile (0.1% v/v formic acid) in H_2_O (0.3% v/v formic acid) over 7 min at 40 μL min^−1^. Peptides were detected using a Synapt G2Si mass spectrometer (Waters, UK). The mass spectrometer was operated in HDMS^E^ mode, with dynamic range extension enabled (data independent analysis (DIA) coupled with IMS separation) were used to separate peptides prior to CID fragmentation in the transfer cell^114^. CID data were used for peptide identification, and uptake quantification was performed at the peptide level (as CID results in deuterium scrambling). Data were analysed using PLGS (v3.0.2) and DynamX (v3.0.0) software (Waters, UK). Search parameters in DynamX were as follows: peptide and fragment tolerances = automatic, min fragment ion matches = 1, digest reagent = non-specific, false discovery rate = 4. Restrictions for peptides in DynamX were as follows: minimum intensity = 1000, minimum products per amino acid = 0.3, max sequence length = 25, max ppm error = 5, file threshold = 3. The software Deuteros^115^ was used to identify peptides with statistically significant increases/decreases in deuterium uptake (applying a 99% confidence interval) and to prepare Wood’s plots. The raw HDX-MS data, have been deposited to the ProteomeXchange Consortium via the PRIDE^93^ partner repository with the dataset identifier PXD017010. A summary of the HDX-MS data, as recommended by reported guidelines^116^, is shown in Supplementary Table 9.

### Electrostatic surface potential and conservation analyses

Calculation of the surface electrostatic potential of SurA was performed using the APBS plugin for PyMOL^117^. Amino acid conservation analysis was carried out using the ConSurf webserver using default parameters^118^.

### Reporting summary

Further information on research design is available in the Nature Research Reporting Summary linked to this article.

## Supplementary information

Supplementary Information

Description of Additional Supplementary Files

Supplementary Movie 1

Supplementary Movie 2

Supplementary Movie 3

Supplementary Data 1

Supplementary Data 2

Reporting Summary

## Data Availability

MS data, have been deposited to the ProteomeXchange Consortium via the PRIDE^93^ partner repository with the dataset identifiers PXD016993 (XL-MS) and PXD017010(HDX-MS). Source data for Figs. 4a and 5a are provided with the paper. MD trajectories and the final structures after 1 μs of simulation) and the structures of the 10 lowest energy Raw smFRET data, MD simulation data (including GROMACS input files, starting structures, and conformations of SurA from simulated annealing are freely available at the University of Leeds data repository (https://doi.org/10.5518/701). The source data underlying Fig. 4a, Fig. 5a, Supplementary Fig. 8b, Supplementary Fig. 7 and Supplementary Table 2 are provided as a Source Data file. All other data are available from the corresponding author on reasonable request.

## Source data

Source Data
