# Supplementary material for: Inter-domain dynamics in the chaperone SurA and multi-site binding to its outer membrane protein clients
Source: Nat Commun. 2020 May 1;11:2155. doi: 10.1038/s41467-020-15702-1 (PMC7195389; doi:10.1038/s41467-020-15702-1)
Supplement: Supplementary file 1 — Supplementary Information [file 41467_2020_15702_MOESM1_ESM.docx]

**Supplementary Information**

**Inter-domain dynamics in the chaperone SurA and multi-site binding to its outer membrane protein clients**

Antonio N. Calabrese^1,^*, Bob Schiffrin^1,^*, Matthew Watson^1,^*, Theodoros K. Karamanos^1,2^, Martin Walko^1,3^, Julia R. Humes^1^, Jim E. Horne^1^, Paul White ^1^, Andrew J. Wilson^1,3^, Antreas C. Kalli^4^, Roman Tuma^1,5^, Alison E. Ashcroft^1^, David J. Brockwell^1^, Sheena E. Radford^1#^

**^1^**Astbury Centre for Structural Molecular Biology, School of Molecular and Cellular Biology, Faculty of Biological Sciences, University of Leeds, Leeds LS2 9JT, UK

^2^Current address: National Institute of Diabetes and Digestive and Kidney Diseases, National Institutes of Health, Bethesda, MD, 20892, USA

**^3^**Astbury Centre for Structural Molecular Biology, School of Chemistry, University of Leeds, Leeds LS2 9JT, UK

**^4^**Astbury Centre for Structural Molecular Biology and School of Medicine, University of Leeds, Leeds LS2 9JT, UK

^5^Faculty of Science, University of South Bohemia, Ceske Budejovice, Czech Republic

*Equal contribution

^#^To whom correspondence should be addressed: [s.e.radford@leeds.ac.uk](mailto:s.e.radford@leeds.ac.uk), Telephone: +44 113 343 3170


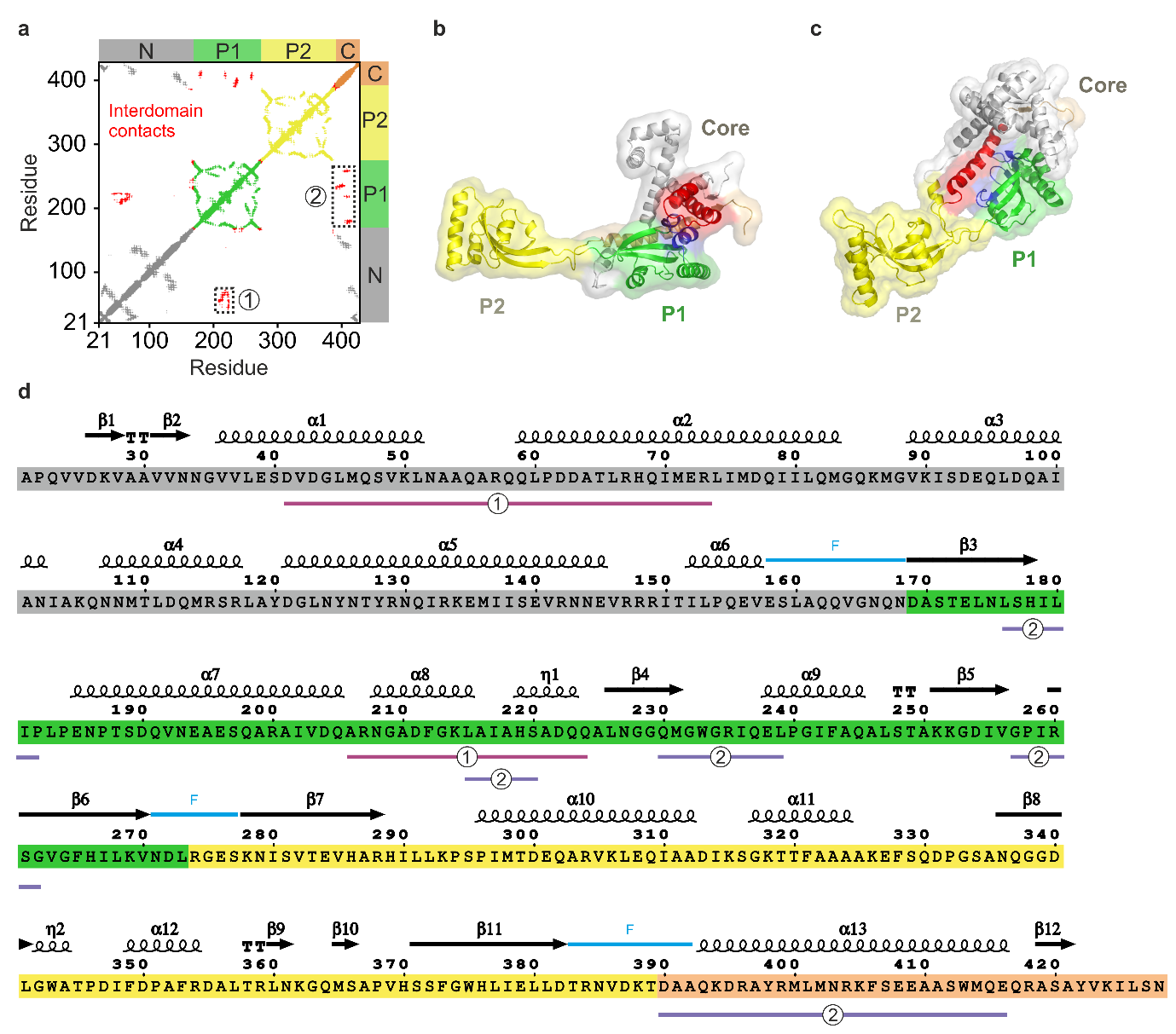


**Supplementary Fig. 1. Inter-domain contacts and secondary structure of *E. coli* SurA.** **(a)** Contact map showing residue pairs with a Cα-Cα distance of less than 12 Å. Contacts within the core (N- and C-terminal regions), P1 and P2 domains are coloured grey, green and yellow, respectively. Inter-domain contacts are shown in red. Contacts between the core and P1 domains cluster in two regions (shown by boxed regions 1 and 2). These clusters correspond to contacts between P1 and the N-terminal region of the core domain (cluster 1), and contacts between P1 and the C-terminal helix (cluster 2). **(b)** Inter-domain contacts from cluster 1 in **(a)**, involving contacts between P1 and the N-terminal region of the core domain. Residues involved in inter-domain contacts in the core and P1 domains are coloured in red and blue, respectively. **(c)** Inter-domain contacts from cluster 2 in **(a)**, summarising contacts between P1 and the C-terminal helix of the core domain. Residues are coloured as in **(b)**. **(d)** SurA sequence coloured by domains (grey: N-terminal region of the core domain, green: P1, yellow: P2, orange: C-terminal region of the core domain). Regions of secondary structure are shown above the sequence: α-helices (α), β-strands (β), 3_10_-helices (η) and turns (T). Flexible regions (F) linking domains are indicated with cyan lines and the two clusters (1 and 2) of contacts from **(a-c)** are indicated. The figure was prepared using the ENDscript server ^1^.

**
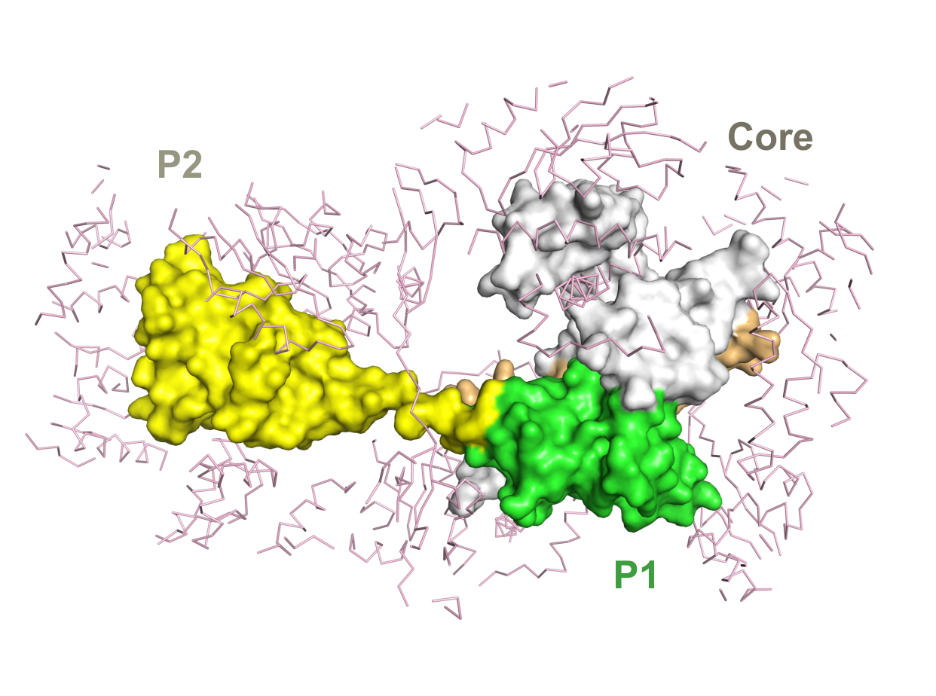
**

**Supplementary Fig. 2. Multiple packing interactions in the crystal structure of full-length SurA (PDB: 1M5Y ^2^)**. One copy of full-length SurA is shown in surface representation with the N-terminal region of the core, P1, P2, and the C-terminal region of the core shown in grey, green, yellow and orange, respectively. Atoms from neighbouring molecules in the crystal within 20 [Å](https://en.wikipedia.org/wiki/%C3%85) are shown in pink in ribbon representation.


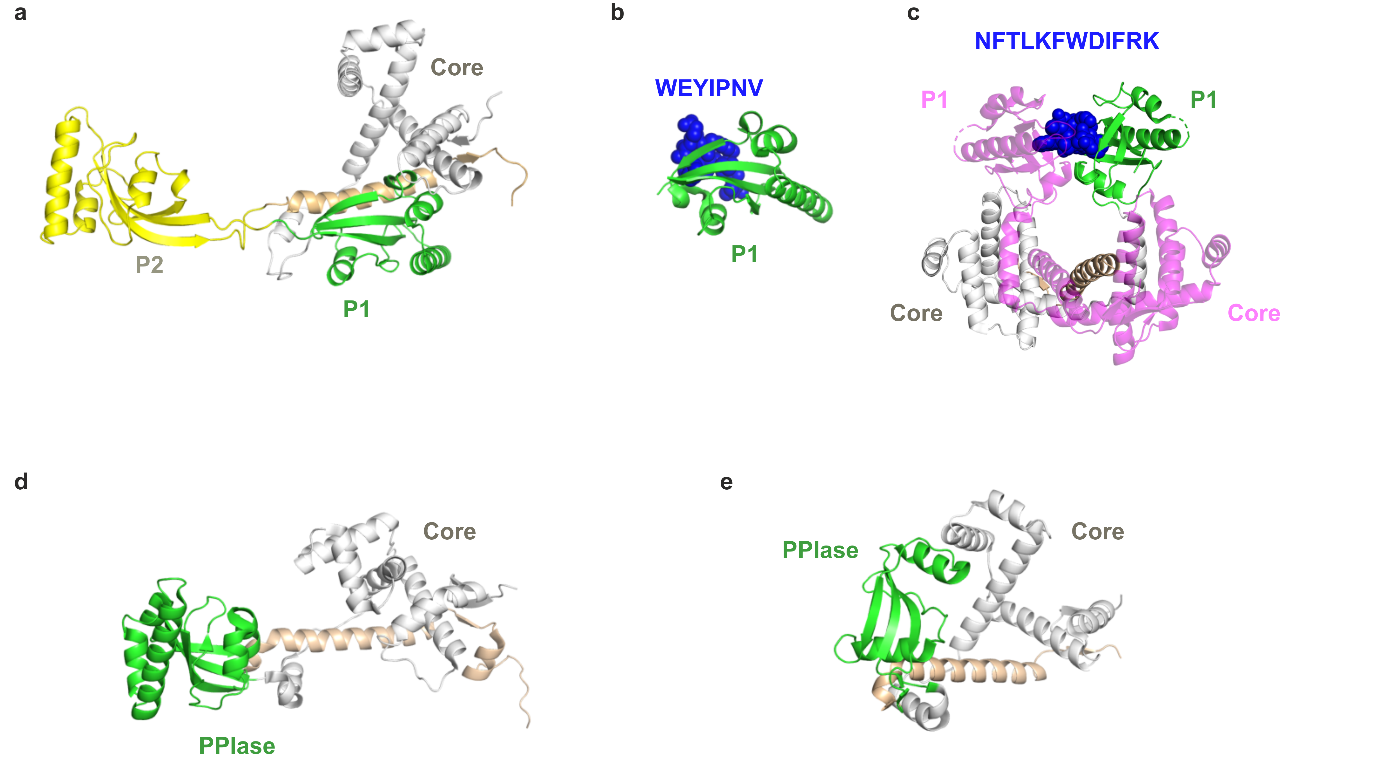


**Supplementary Fig. 3. Crystal structures of *E. coli* SurA, SurA-peptide complexes and SurA homologues.** **(a)** Structure of *E. coli* SurA (PDB: 1M5Y ^2^). **(b)** Structure of the P1 domain of *E. coli* SurA bound to the peptide WEYIPNV (blue spheres) (PDB: 2PV1 ^3^). **(c)** Structure of dimeric *E. coli* SurA-ΔP2 in complex with the peptide NFTLKFWDIFRK (blue spheres) (PDB: 2PV3 ^3^). For clarity, one monomer is coloured as in **(a)** and the other is shown in pink. **(d)** Structure of the SurA homologue LIC12922 from *Leptospira interrogans (*PDB: 3NRK) ^4^. **(e)** Structure of SurA homologue Cj1289 from *Campylobacter jejuni*, (PDB: 3RGC) ^5^. Note that *E. coli* SurA homologues from γ- and β- proteobacteria commonly contain two PPIase domains, whilst those from α- ε- and δ-proteobacteria more commonly contain only one PPIase domain ^6^. *C. jejuni* is a member of the ε-proteobacterial class, and *Leptospira interrogans* is a member of the phylum Spirochaetes.

**
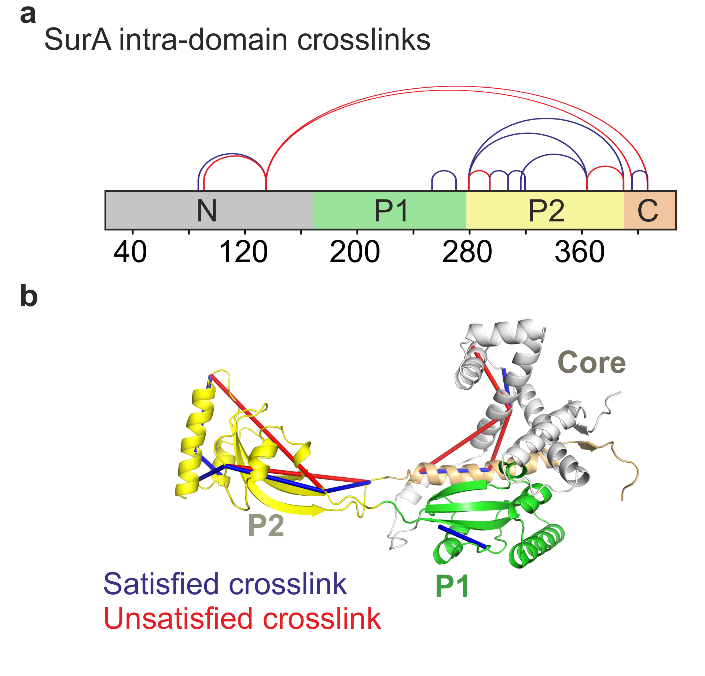
**

**Supplementary Fig. 4. Intra-domain Lys-Lys cross-links in apo-SurA.** **(a)** Locations of the 13 experimentally observed SurA intra-domain crosslinks. Crosslinks between residues with a solvent accessible surface distance (SASD) of less than or greater than 35 Å in the crystal structure of full-length SurA (PDB: 1M5Y ^2^) are defined as satisfied (blue) (8 crosslinks) or violated (red) (5 crosslinks), respectively. **(b)** Locations of all intra-domain cross-links shown on the SurA crystal structure (PDB 1M5Y ^2^). Note that for clarity crosslinks are shown as straight lines between residues, rather than solvent accessible distance paths, coloured as in **(a)**. Details of crosslinked residues are given in **Supplementary Table 1**. A representative mass spectrum for each crosslink can be found in Supplementary Data 1.

***
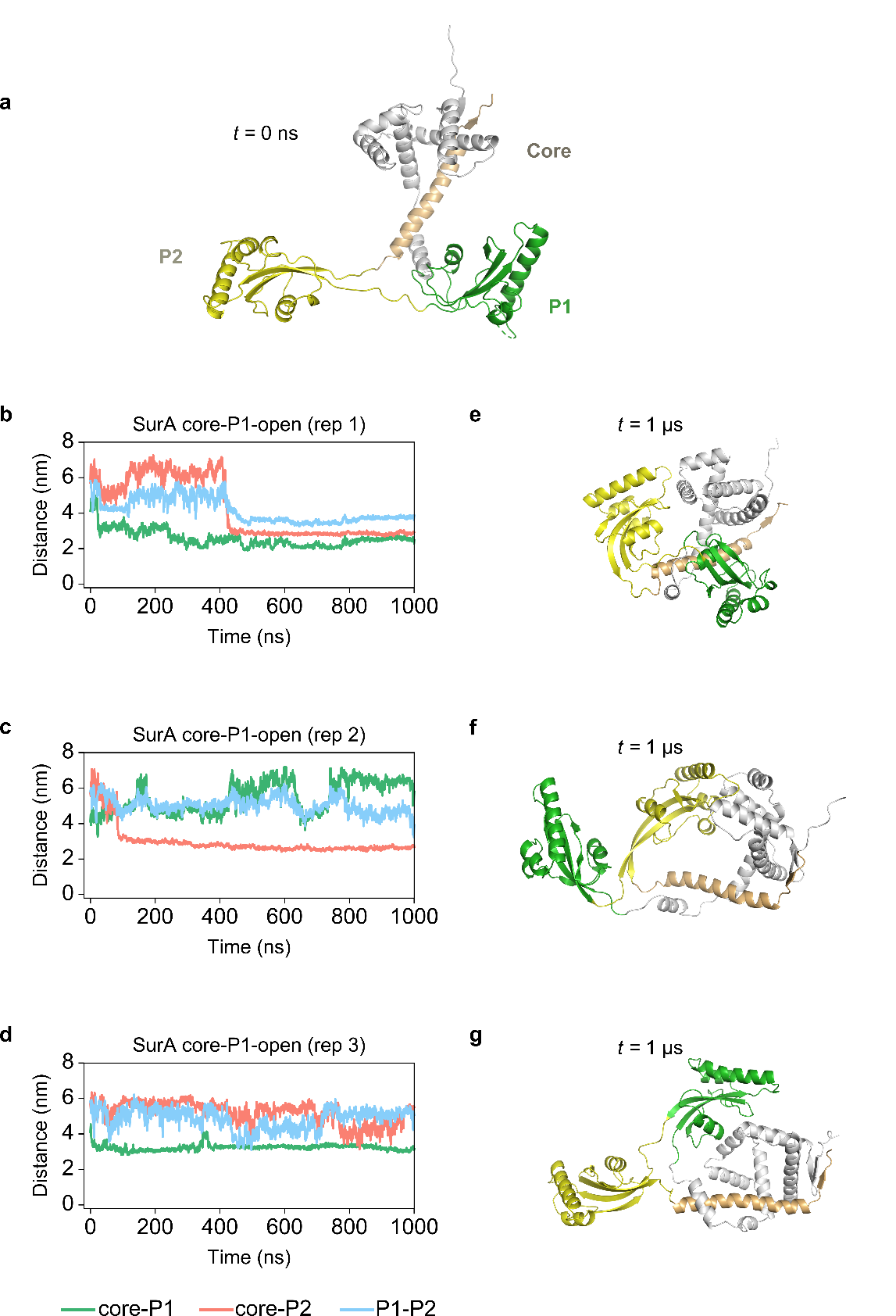
***

**Supplementary Fig. 5. MD simulations of SurA^core-P1-open^ suggest a broad conformational ensemble with a range of inter-domain orientations and distances. (a)** Model of a SurA^core‑P1‑open^ conformation used as a starting structure for simulation. The model was generated using the X-ray structures of full-length SurA (PDB: 1M5Y ^2^) and SurA-ΔP2 (PDB: 2PV3 ^3^) (see Methods). **(b-d)** Inter-domain distances over time for each of 3 x 1 μs simulations starting from the SurA^core-P1-open^ model shown in **(a)**. Inter-domain distances between the centre of mass of each domain for core-P1, core-P2 and P1‑P2, are shown in green, red and blue, respectively. **(e-g)** Structures at the end of 1 μs simulation for each of replicates 1, 2 and 3, respectively. Rep: repeat simulation.


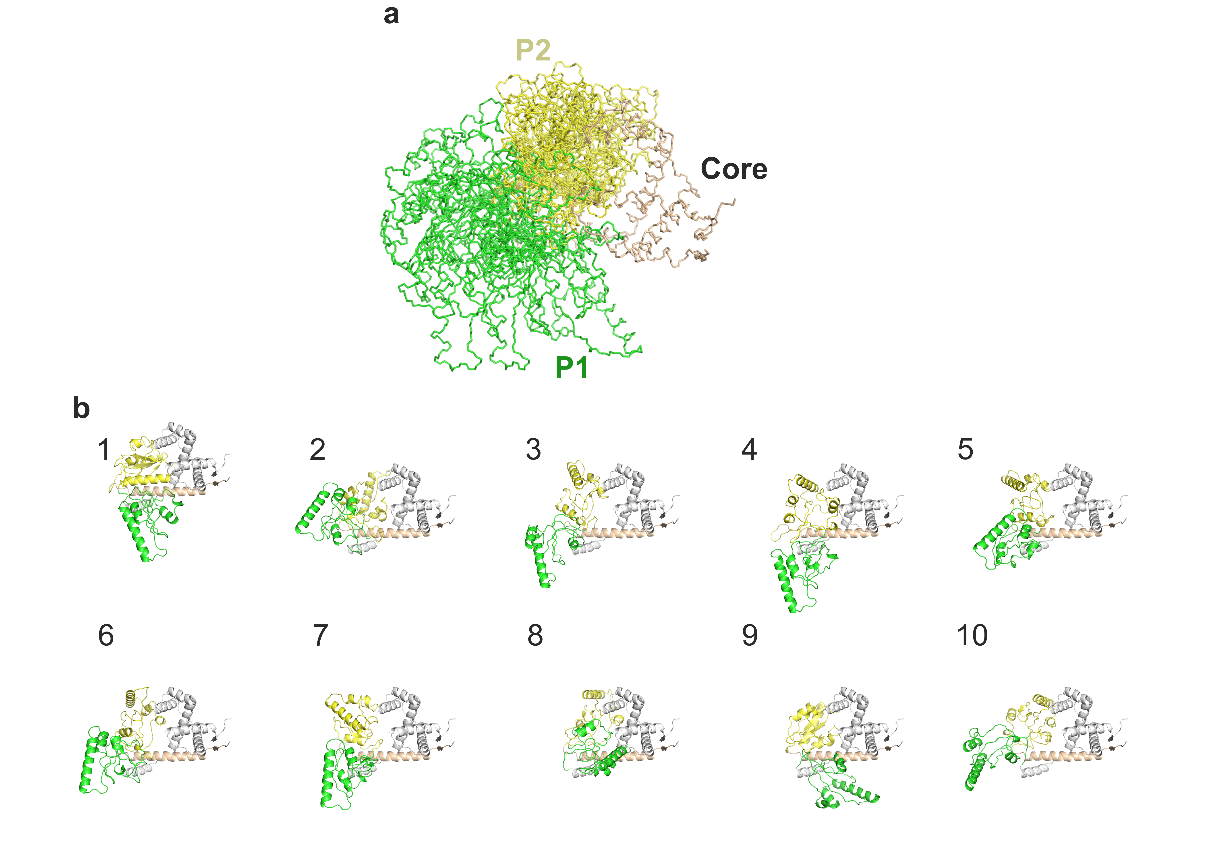


**Supplementary Fig. 6. Simulated annealing MD results in structures consistent with the XL-MS data.** **(a)** Superposition of the 10 lowest energy structures of SurA from simulated annealing MD. The structures are all aligned on the core domain. The 10 structures were derived by ranking the 100 generated structures based on their energies, taking into account how well the distance restraints are satisfied (this is the main contributor to the final energy) and covalent geometry/van der Waals terms to ensure that the selected models do not have any geometry violations. **(b)** The 10 lowest energy structures of SurA from simulated annealing MD.

**
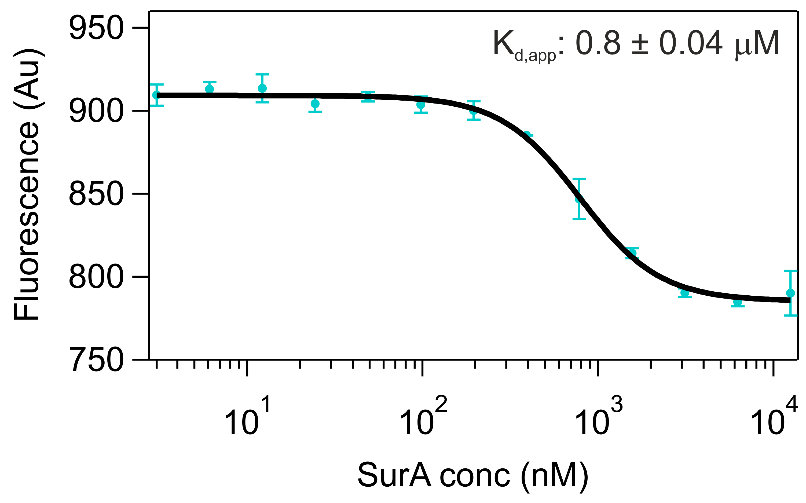
**

**Supplementary Fig. 7. OmpX binds to SurA with μM** **affinity.** Microscale thermophoresis (MST) data for binding of SurA to OmpX. Samples contained 50 nM Alexa Fluor 488-labelled OmpX (see Methods), SurA (0.3 nM-12.5 μM), 0.24 M urea, 50 mM Tris-HCl, pH 8.0, 25 °C. A fit to the Hill equation is indicated by a black solid line. Data are shown as the mean ± standard deviation of three independent technical replicates. The fitted values for K_d,app_ and Hill coefficient were 0.8 ± 0.04 μM and 1.5 ± 0.1, respectively. Source data are provided as a Source Data file.


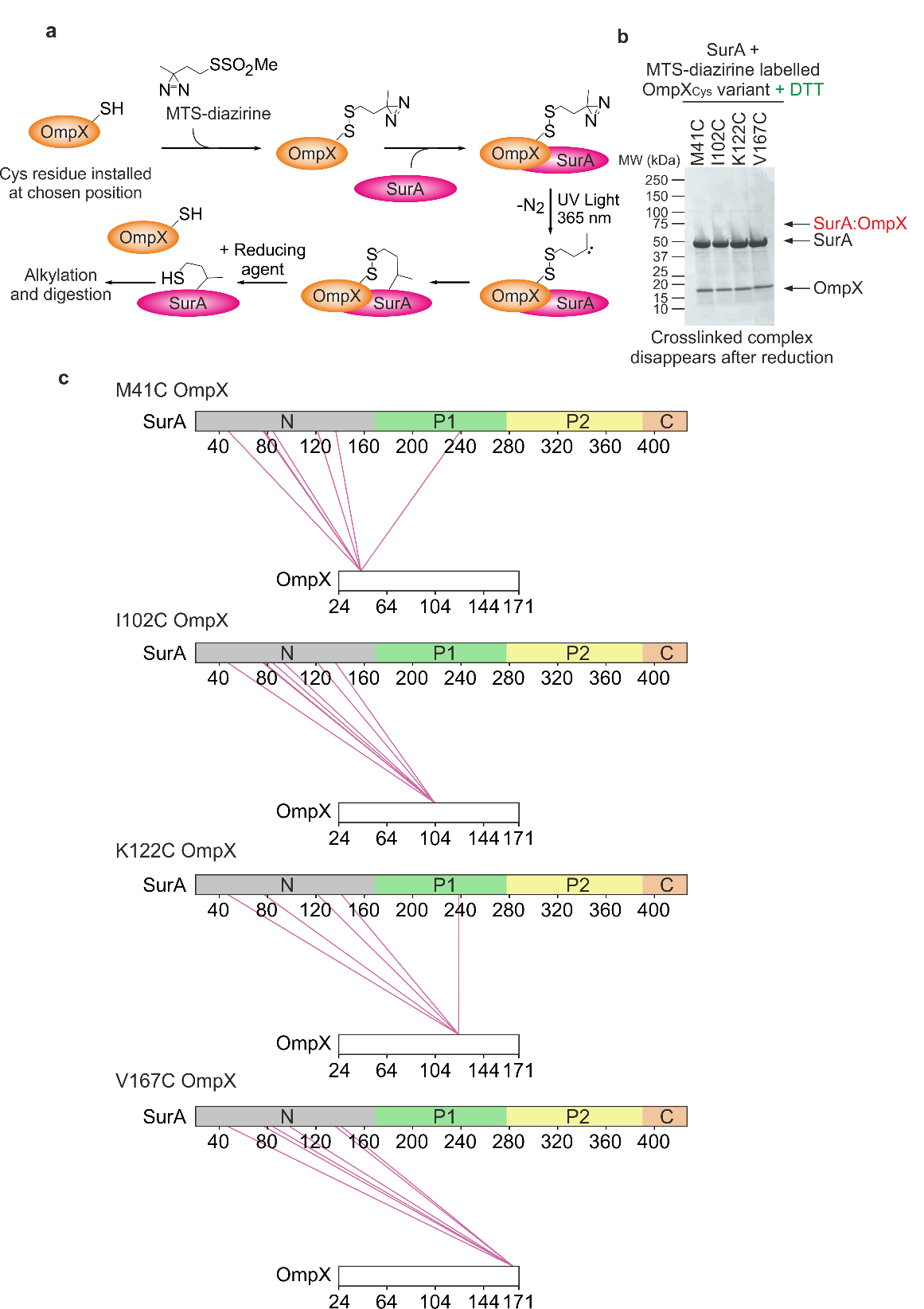


**Supplementary Fig. 8. Multiple locations across the OmpX sequence interact with similar sites on SurA. (a)** Outline of the ‘tag-transfer’ photo-crosslinking workflow (see Methods) ^7^. **(b)** SDS-PAGE analysis of photo-crosslinking reactions between SurA and ‘tagged’ OmpX variants under reducing conditions. Note that the band corresponding to the SurA-OmpX complex is lost in the presence of reducing agent (compare with **Fig. 5a**). Source data are provided as a Source Data file. **(c)** Crosslinks (pink lines) identified for each of the Cys residues introduced into the OmpX sequence. Note that the combined dataset for all four Cys variants are shown in **Fig. 5b and 5c**. A representative mass spectrum for each crosslink can be found in Supplementary Data 1.


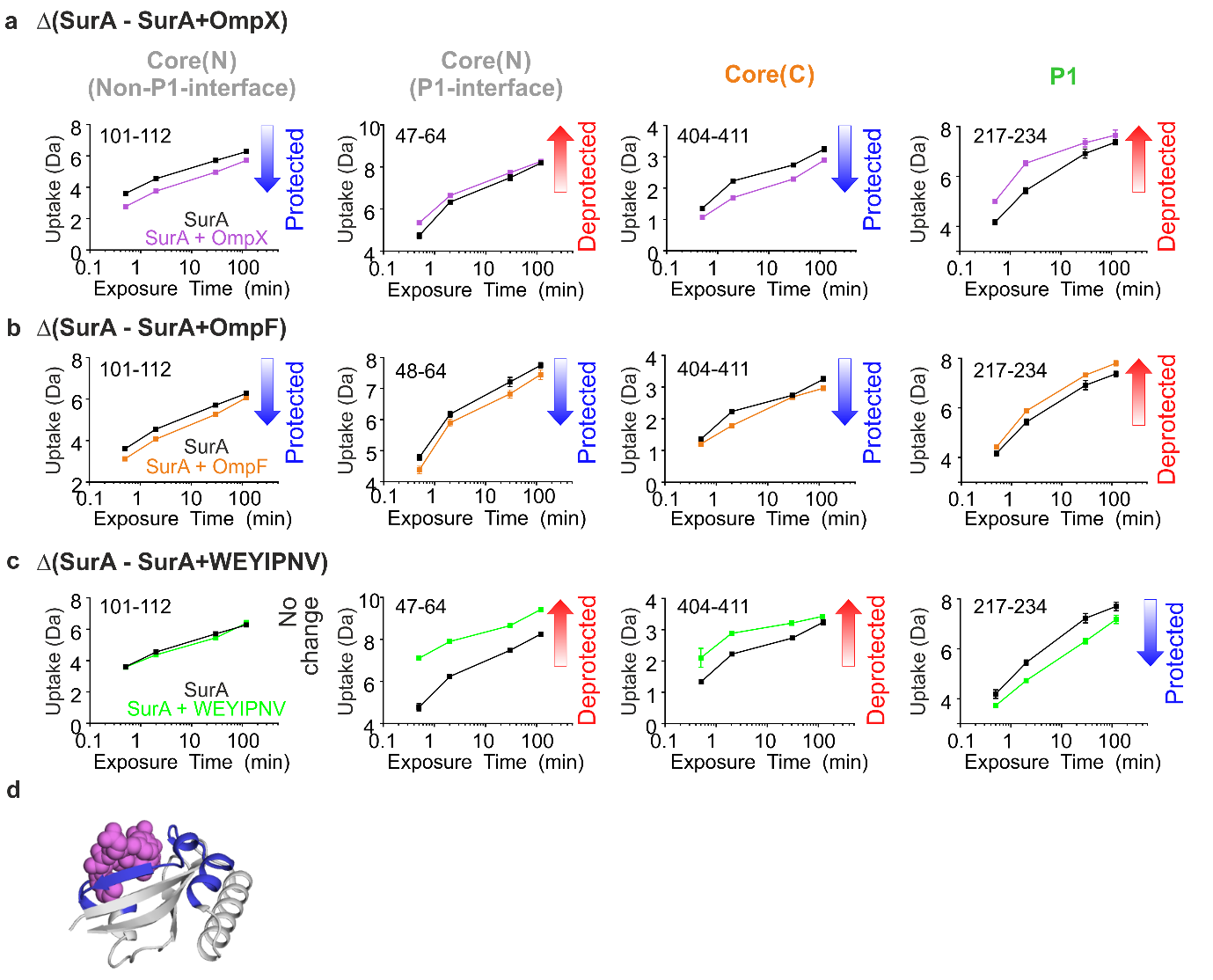


**Supplementary Fig. 9. Different regions of SurA are protected or deprotected from hydrogen exchange in the presence of OmpX, OmpF or WEYIPNV.** Example deuterium uptake curves for SurA (black) or SurA in the presence of **(a)** OmpX, **(b)** OmpF or **(c)** WEYIPNV (coloured as indicated) for four regions of SurA: (left) N-terminal region of the core domain distal to the core-P1 interface, (second from left) N-terminal region of the core domain at the core-P1 interface, (third from left) the C-terminal region of the core domain, and (right), the P1 domain at the interface with the core. The residue numbers in each peptide are indicated in the top left of each plot. **(d)** Crystal structure of WEYIPNV bound to the P1 domain of SurA (PDB 2PV1 ^3^). The peptide is shown as purple spheres, and the region of SurA protected from HDX in the presence of WEYIPNV is shown in blue. See Methods for experimental details. Data are shown as mean ± standard deviation of three technical replicate measurements.

**
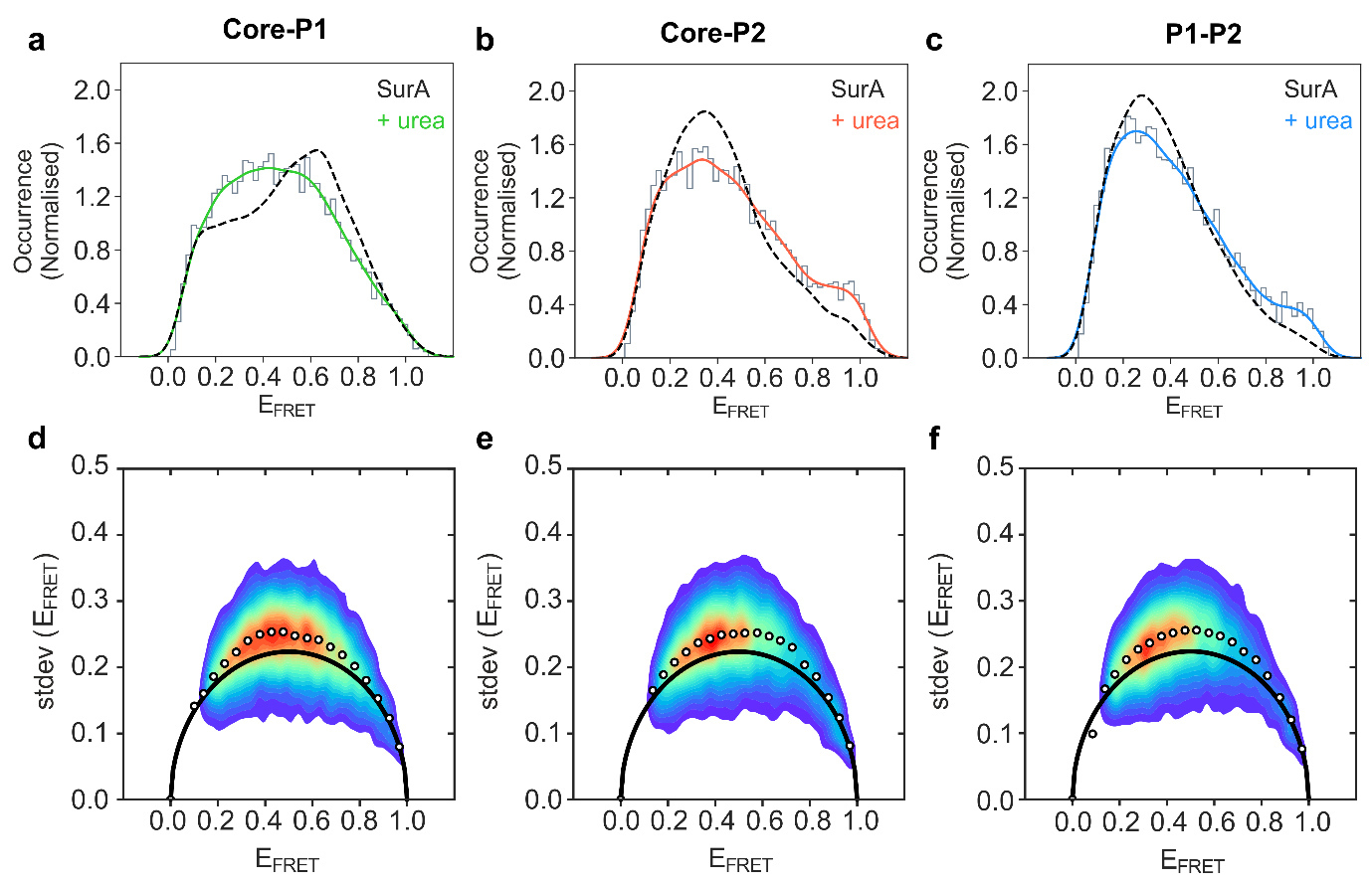
**

**Supplementary Fig. 10. Addition of 0.24 M urea to SurA alters the equilibrium between core-P1_open_ and core‑P1_closed_** conformations. (**a-c**) Experimentally measured E_FRET_ distributions for the three pairwise combinations of fluorescently-labelled SurA double mutants **(a)** core-P1, **(b)** core-P2, and **(c)** P1-P2 in the presence of 0.24 M urea. In each plot, kernel density estimations (KDEs) of the probability density function of the measured E_FRET_ values are shown in green, red or blue for data acquired in the presence of 0.24 M urea, while the KDE in the absence of urea are shown as a dashed black line (taken from data in Fig. 3d-f). (**d-f**) Burst variance analysis showed that the presence of urea increased the proportion of intra burst timescale (sub-ms) motions compared with those observed in the absence of urea (compare with Fig. 3g-i). The black lines indicate the expected shot-noise limited standard deviation as a function of E_FRET_. The average values of the measured variance for the range of E_FRET_ values (white circles) above the theoretical distribution indicate dynamics on a timescale faster than the duration of the bursts (here sub-ms). Samples contained ~50 pM labelled SurA variant in 0.24 M urea, 50 mM Tris‑HCl, pH 8.0, 25 ºC.

**
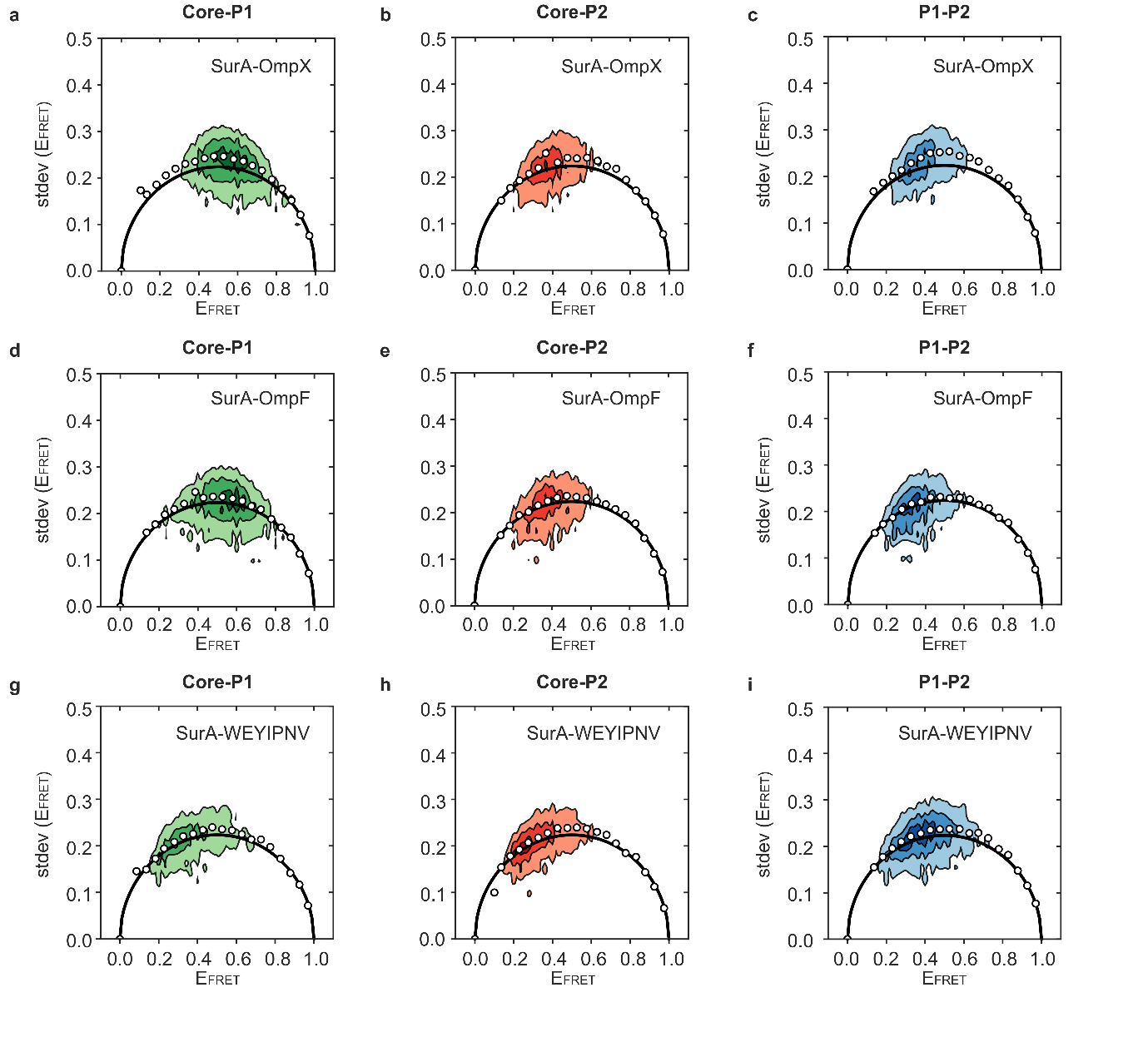
**

**Supplementary Fig. 11. Burst variance analysis of smFRET data for SurA in complex with OmpX, OmpF or the peptide WEYIPNV indicates sub-ms inter-domain dynamics.** BVA analysis for SurA in the presence of **(a-c)** OmpX, **(d-f)** OmpF or **(g-i)** WEYIPNV for all three pairwise combinations of fluorescently-labelled SurA double mutants **(a,d,g)** core-P1, **(b,e,h)** core-P2, or **(c,f,i)** P1-P2. Each detected burst was divided into sub-bursts each containing 5 photons. E_FRET_ values were calculated for each sub-burst and the E_FRET_ value for each burst plotted against the standard deviation of the sub-burst E_FRET_ values within the burst. The black arcs indicate the expected shot-noise limited standard deviation as a function of E_FRET_. The average values of the measured variance (white circles) above the theoretical distribution indicate dynamics on a timescale faster than the duration of the bursts (here sub-ms).


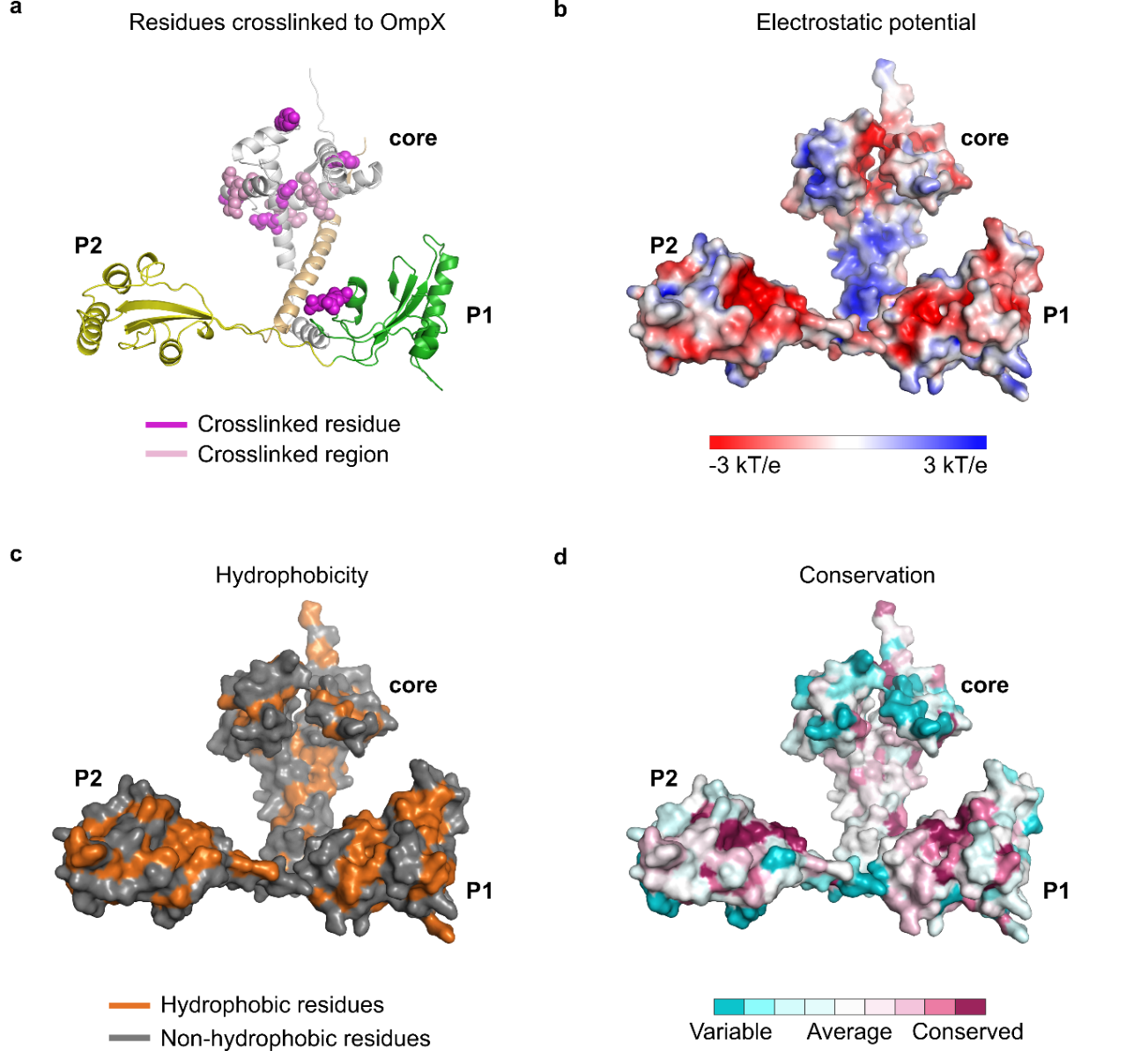


**Supplementary Fig. 12. Regions of SurA crosslinked to OmpX in tag-transfer crosslinking experiments show no obvious correlation with locations of a particular electrostatic surface potential, hydrophobicity or with areas of high conservation on SurA. (a)** Crosslinks identified in tag-transfer experiments between OmpX and SurA (**Fig. 5, Supplementary Fig. 8)** mapped to a single residue (dark purple) or short peptide (light purple). **(b)** Electrostatic surface representation of SurA (-3 kT/e to +3 kT/e) generated using the APBS plugin for PyMOL ^8^. **(c)** Surface hydrophobicity of SurA. Hydrophobic residues (Gly, Ala, Val, Leu, Ile, Pro, Phe, Met, and Trp) are shown in orange, all other residues are highlighted in grey. **(d)** Residue conservation of SurA. Amino acid conservation is indicated as a colour gradient between cyan and red for variable and conserved residues, respectively. Conservation scores were generated using the ConSurf webserver (see Methods) ^9^. Data are illustrated using the SurA^core‑P1‑open^ model used as a starting structure for simulation (**Supplementary** **Fig.6a**).

**
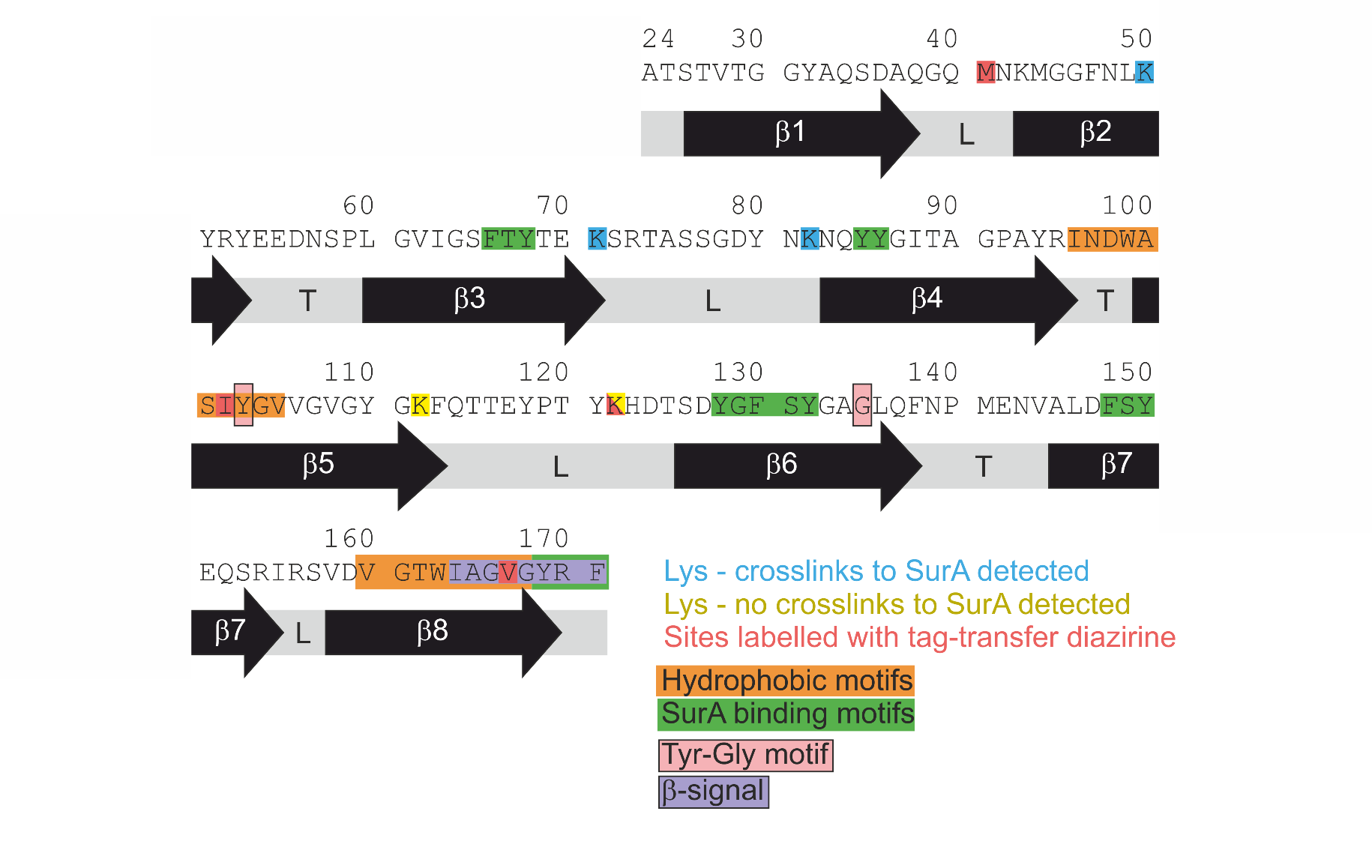
**

**Supplementary Fig. 13. OmpX sequence motifs and the location of crosslinks detected to SurA.** OmpX sequence with regions that form β-strands, loops (L) and turns (T) in the folded state are indicated. Residues that crosslink to SurA are shown in blue and red. A number of sequence motifs in OMPs have been implicated in facilitating folding. The central hairpin (β4-β5) has been proposed to nucleate assembly ^10^, hydrophobic stretches (orange) mediate membrane interactions and hydrophobic collapse ^11^, a tyrosine-glycine motif in OmpX (pink) is proposed to lock β5 and β6 together, stabilising the folded state ^12^, and the β-signal^13^ (purple blue) and aromatic motifs^14-17^ (green) (Ar-X-Ar or Ar-Ar, where Ar is an aromatic residue and X is any residue) are thought to mediate BAM and SurA recognition, respectively. Many of the detected crosslinks are close to these motifs. Note that the residues are numbered including the signal sequence (not shown here).

**
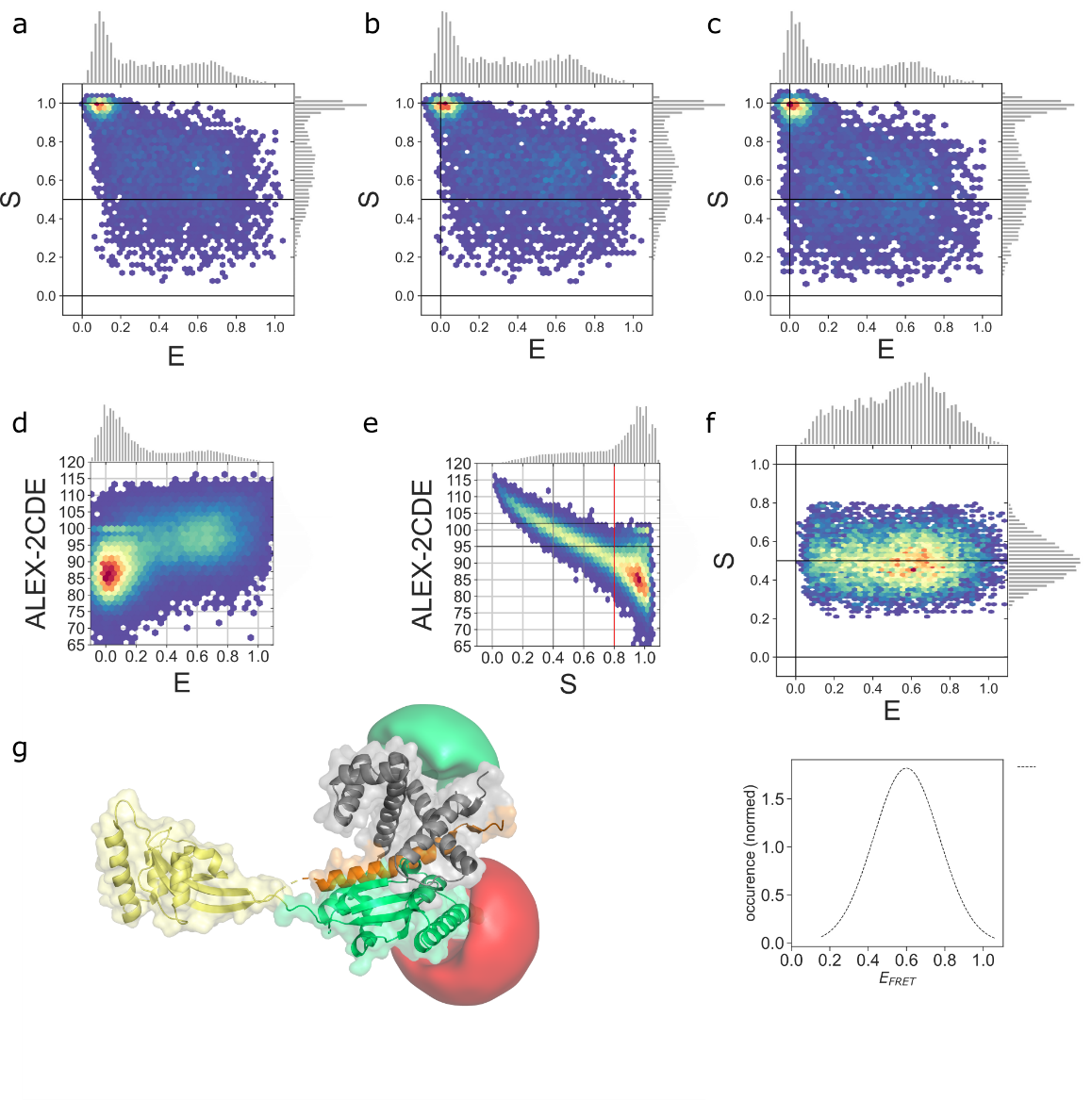
**

**Supplementary Fig. 14. Example stepwise data correction using the FRETBursts package for apo SurA labelled on the core and P1 domains**. The uncorrected fluorescence intensities within bursts returned by an initial burst search algorithm with a stringent threshold of 100 photons per bin are converted into uncorrected apparent FRET efficiency (^i^E_app_, eq. 1 in Methods) and dye stoichiometry (^i^S_app_, eq. 2 in Methods), respectively. The burst search algorithm also estimates a background signal value for each channel which is subtracted from the signals to yield background corrected ^ii^E_app_ and ^ii^S_app_ which are shown in panel (a). These data are further corrected by applying several numerical correction factors to account for leakage of the donor emission into the acceptor channel (α, eq. 3 in Methods) and direct excitation of the acceptor dye by the donor laser (δ, eq. 4 in Methods). These two parameters are estimated by constraining the corrected donor only peak to E = 0, S = 1 (intersection of vertical black line with top horizontal line, panel b). Next, excitation and detection efficiency correction factors are applied (γ defined by eq. 5 and β by eq. 6 in Methods) in order that the maximum density of bursts (corresponding to molecules containing both dyes) map around line S=0.5 (middle horizontal black line, panel c)^18^ for the corrected ^iii^S_app_ and ^iii^E_app_. In order to remove the donor only population, the ALEX 2CDE filter was applied^19^ (panels d and e), and those bursts with S between 0.4 and 0.6 (grey lines) selected *by their ALEX 2CDE values* (black lines here 95 -102) and cropped to remove bursts with S > 0.8 (e). This returns the fully corrected distribution of bursts and allows the iterative use of the β and γ factors to maximise the number of bursts with S around 0.5. In this case, the sample exhibited multiple populations which facilitated the empirical determination of β and γ by systematically varying them to optimize the graphical result of the remaining bursts and allowed the threshold for burst selection to be lowered to 20 photons once the correction parameters were established using the more stringent threshold (100 photons) to maximise the total number of bursts identified (f). The projected distribution of E_FRET_ values returned from this process for each investigated condition are presented in the main text and fitted to one or two Gaussians. The most abundant peak was found to agree very well with a single Gaussian fit of the distribution predicted for the crystal structure using the core-P1 mutant labelling positions (g). As the sample also exhibited another population with a very different E_FRET_ it was suitable for use as a single species etalon^18^. The γ and β factors were optimised as described for each sample, the γ factor used was always 1 while the β factor was 0.7- 0.8. We also further validated our use of a γ-factor of 1 using a polyproline 20 mer which exhibited good agreement between the experimental distribution of E_FRET_ and that predicted using the MtsslWizard PyMOL plugin^20^.

**Supplementary Tables**

**Supplementary Table 1: Inter-domain and intra-domain crosslinks identified for apo-SurA and their SLDs and SASDs calculated from the crystal structure of *E. coli* SurA (PDB 1M5Y ^2^).** DSBU has been shown to crosslink residues within ~27-30 Å (Cα-Cα SLDs) ^21^. To account for uncertainty due to the resolution in the crystal structure, crosslinks are defined as satisfied (blue) or violated (red) if they are separated by SLD of less than or greater than 28 Å, respectively. Crosslinks between residues with a Cα‑Cα SASDs of less than or greater than 35 Å are defined as satisfied (blue) or violated (red), respectively. An asterisk (*) indicates that the distance indicated is between a residue pair in which one partner is not present in the crystal structure and the distance is therefore an approximation based on filled missing loop residues using MODELLER ^22^ (see Methods). A representative mass spectrum for each crosslink can be found in Supplementary Data 1.

|  | **Cross-links**  **identified** | **Cα-Cα SLD (Å)** | **SASD (Å)** |
| --- | --- | --- | --- |
| **Inter-domain crosslinks** | | | |
| Core-P1 | K251-K405 | 27 | 36 |
|  | K252-K394 | ~26* | ~44 * |
|  | K269-K394 | ~25* | ~46* |
| Core-P2 | K105-K278 | 28 | 32 |
|  | K105-K293 | 51 | 55 |
|  | K134-K278 | 41 | 53 |
|  | K134-K293 | 67 | 80 |
|  | K278-K394 | ~27* | ~32* |
|  | K278-K405 | 29 | 37 |
|  | K293-K394 | 54* | 71^*^ |
|  | K293-K405 | 60 | 79 |
|  | K362-K405 | 50 | 58 |
| P1-P2 | K251-K278 | 21 | 27 |
|  | K251-K293 | 63 | 73 |
|  | K252-K278 | 21 | 26 |
|  | K252-K293 | 63 | 76 |
|  | K252-K362 | 47 | 53 |
|  | K269-K278 | 26 | 36 |
|  | K269-K293 | 66 | 84 |
| **Intra-domain cross-links** | | |  |
| Core-Core | K86 K134 | 14 | 21 |
|  | K90 K134 | 10 | 39 |
|  | K134 K394 | ~26^*^ | ~36* |
|  | K134 K405 | 26 | 44 |
|  | K394 K405 | ~16^*^ | ~34* |
| P1-P1 | K252 K269 | 9 | 16 |
| P2-P2 | K278-K293 | 42 | 52 |
|  | K278-K362 | 27 | 30 |
|  | K278-K388 | ~16* | ~17* |
|  | K293-K306 | 11 | 26 |
|  | K306-K318 | 15 | 22 |
|  | K315-K362 | 15 | 20 |
|  | K362-K388 | ~34* | ~52* |

**Supplementary Table 2. Steady state anisotropy measurements.** Single cysteine variants of SurA were labelled with either Alexa Fluor 488 or Alexa Fluor 594 dyes and the anisotropy measured as described in the Methods. The measured steady-state anisotropies of all the samples are similar and low, indicating good dye mobility, allowing changes in E_FRET_ to be ascribed to changes in inter-domain distances. The steady anisotropy remained low for all dyes at all positions in the presence of 0.24 M urea or 0.24 M urea with added 3 μM OmpX. Data are shown as mean ± SEM of three replicate measurements. Source data are provided as a Source Data file.

| SurA sample | Anisotropy |
| --- | --- |
| 488-Q85C | 0.14 ± 0.01 |
| 488-N193C | 0.16 ± 0.01 |
| 488-E301C | 0.16 ± 0.01 |
| 594-Q85C | 0.11 ± 0.04 |
| 594-N193C | 0.11 ± 0.08 |
| 594-E301C | 0.16 ± 0.06 |
| 488-Q85C + OmpX + urea | 0.12 ± 0.02 |
| 488-N193C + OmpX + urea | 0.15 ± 0.01 |
| 488-E301C + OmpX + urea | 0.10 ± 0.01 |
| 594-Q85C + OmpX + urea | 0.15 ± 0.08 |
| 594-N193C + OmpX + urea | 0.17 ± 0.03 |
| 594-E301C + OmpX + urea | 0.17 ± 0.03 |
| 488-Q85C + urea | 0.11 ± 0.01 |
| 488-N193C + urea | 0.11 ± 0.00 |
| 488-E301C + urea | 0.14 ± 0.02 |
| 594-Q85C + urea | 0.18 ± 0.05 |
| 594-N193C + urea | 0.21 ± 0.02 |
| 594-E301C + urea | 0.15 ± 0.04 |

**Supplementary Table 3. Inter-domain Lys-Lys crosslinks identified for apo-SurA and their minimum SLDs in the structures at the end of the three 1 µs simulations.** DSBU has been shown to crosslink residues within ~27 - 30 Å (Cα-Cα SLDs) ^21^. To account for uncertainty due to the resolution of the crystal structure, crosslinks are defined satisfied (blue) if the Cα-Cα SLDs of the residues involved are less than/equal to 28 Å, or violated (red) if they are greater than 28 Å. An asterisk (*) indicates that the measured distance is between a residue pair in which one partner is not present in the crystal structure and this distance is therefore an approximation based on filled missing loop residues using MODELLER ^22^. Rep: repeat simulation. Note that SLDs were used here to assess compatibility with the crosslinks observed as calculating SASDs across the entire MD trajectories is not feasible computationally.

| **Inter-domain crosslinks** | **Cross-links identified** | **1M5Y distances (Å)** | **Distance (SLD) in the final structure of the simulation (Å)** | | |  |
| --- | --- | --- | --- | --- | --- | --- |
|  |  |  | **Rep 1** | **Rep 2** | **Rep 3** | |
| Core-P1 | K251-K405 | 27 | 21 | 39 | 38 | |
|  | K252-K394 | ~26* | 25 | 33 | 34 | |
|  | K269-K394 | ~25* | 26 | 37 | 39 | |
| Core-P2 | K105-K278 | 28 | 23 | 48 | 27 | |
|  | K105-K293 | 51 | 11 | 14 | 49 | |
|  | K134-K278 | 41 | 36 | 48 | 46 | |
|  | K134-K293 | 67 | 22 | 22 | 69 | |
|  | K278-K394 | ~27* | 18 | 18 | 28 | |
|  | K278-K405 | 29 | 23 | 29 | 39 | |
|  | K293-K394 | 54* | 27 | 33 | 45 | |
|  | K293-K405 | 60 | 28 | 29 | 59 | |
|  | K362-K405 | 49 | 36 | 37 | 41 | |
| P1-P2 | K251-K278 | 21 | 13 | 14 | 22 | |
|  | K251-K293 | 63 | 36 | 43 | 52 | |
|  | K252-K278 | 21 | 16 | 16 | 19 | |
|  | K252-K293 | 63 | 39 | 47 | 49 | |
|  | K252-K362 | 47 | 38 | 39 | 32 | |
|  | K269-K278 | 26 | 21 | 20 | 25 | |
|  | K269-K293 | 66 | 40 | 49 | 55 | |

**Supplementary Table 4: Inter-domain Lys-Lys crosslinks identified for apo-SurA and their minimum SLDs during the 1 μsec MD simulations starting from a SurA^core-P1-open^ model.** DSBU has been shown to crosslink residues within ~27 - 30 Å (Cα-Cα SLDs) ^21^. To account for uncertainty due to the resolution in the crystal structure, crosslinks are defined satisfied (blue) or violated (red) if they are less than or greater than 28 Å, respectively. An asterisk (*) indicates that the measured distance is between a residue pair in which one partner is not present in the crystal structure and this distance is therefore an approximation based on filled missing loop residues using MODELLER ^22^. Rep: repeat simulation.

| **Inter-domain crosslinks** | **Cross-links identified** | **1M5Y distances (Å)** | **Minimum distance (SLD) in the MD simulations (Å)** | | |  |
| --- | --- | --- | --- | --- | --- | --- |
|  |  |  | **Rep 1** | **Rep 2** | **Rep 3** | |
| Core-P1 | K251-K405 | 27 | 18 | 15 | 19 | |
|  | K252-K394 | ~26* | 20 | 13 | 21 | |
|  | K269-K394 | ~25* | 21 | 18 | 29 | |
| Core-P2 | K105-K278 | 28 | 21 | 31 | 12 | |
|  | K105-K293 | 51 | 9 | 8 | 17 | |
|  | K134-K278 | 41 | 30 | 45 | 32 | |
|  | K134-K293 | 67 | 18 | 16 | 24 | |
|  | K278-K394 | ~27* | 15 | 12 | 12 | |
|  | K278-K405 | 29 | 21 | 25 | 20 | |
|  | K293-K394 | 54* | 26 | 23 | 32 | |
|  | K293-K405 | 60 | 24 | 20 | 33 | |
|  | K362-K405 | 49 | 27 | 32 | 22 | |
| P1-P2 | K251-K278 | 21 | 6 | 8 | 10 | |
|  | K251-K293 | 63 | 29 | 33 | 19 | |
|  | K252-K278 | 21 | 6 | 9 | 8 | |
|  | K252-K293 | 63 | 30 | 35 | 17 | |
|  | K252-K362 | 47 | 14 | 25 | 21 | |
|  | K269-K278 | 26 | 14 | 17 | 16 | |
|  | K269-K293 | 66 | 30 | 37 | 22 | |

**Supplementary Table 5. Inter-domain Lys-Lys crosslinks identified for apo-SurA and their SLDs in the 10 lowest energy structures from simulated annealing MD.** Inter-domain crosslinks identified for apo-SurA and their SLDs in the crystal structure of *E. coli* SurA (PDB 1M5Y ^2^) and in the 10 lowest energy structures from simulated annealing MD (**Supplementary Fig. 6**). Crosslinks are defined satisfied (blue) or violated (red) if they are less than or greater than 28 Å, respectively. Where the crosslink is not satisfied in the structure the Cα-Cα SLD is shown. Note that all 19 inter-domain crosslinks were used as restraints in all simulations.

| **Inter-domain crosslinks** | **Cross-links identified** | **SLD between residues if crosslink is not satisfied** | | | | | | | | | |
| --- | --- | --- | --- | --- | --- | --- | --- | --- | --- | --- | --- |
|  |  | **1** | **2** | **3** | **4** | **5** | **6** | **7** | **8** | **9** | **10** |
| Core-P1 | K251-K405 |  |  |  |  |  |  |  |  |  |  |
|  | K252-K394 |  |  |  |  |  |  |  |  |  |  |
|  | K269-K394 |  |  |  |  |  |  |  |  |  |  |
| Core-P2 | K105-K278 |  |  |  |  |  |  |  |  |  |  |
|  | K105-K293 |  |  |  |  |  |  |  |  |  |  |
|  | K134-K278 |  |  |  | 29 |  |  |  | 31 |  |  |
|  | K134-K293 |  |  |  |  |  |  |  |  |  |  |
|  | K278-K394 |  |  |  |  |  |  |  |  |  |  |
|  | K278-K405 |  |  |  |  |  |  |  |  |  |  |
|  | K293-K394 |  | 30 |  |  | 31 | 33 |  | 29 |  |  |
|  | K293-K405 |  |  |  |  | 30 | 32 |  |  |  |  |
|  | K362-K405 |  |  | 34 | 35 | 30 |  | 35 | 30 |  | 36 |
| P1-P2 | K251-K278 |  |  |  |  |  |  |  |  |  |  |
|  | K251-K293 |  |  |  |  |  |  |  |  |  |  |
|  | K252-K278 |  |  |  |  |  |  |  |  |  |  |
|  | K252-K293 |  |  |  |  |  |  |  |  |  |  |
|  | K252-K362 |  |  |  |  |  |  |  | 30 |  |  |
|  | K269-K278 |  |  |  |  |  |  |  |  |  |  |
|  | K269-K293 |  |  |  |  |  |  |  |  | 32 |  |

**Supplementary Table 6. Comparison of smFRET data and the 10 lowest energy structures from simulated annealing MD.** Table of the mean E_FRET_ values for the three FRET pairs predicted from the 10 lowest energy structures from simulated annealing MD (**Supplementary Fig. 6**) and those from the smFRET experimental data (**Fig. 3**). Note that the smFRET distributions are broad and only the peak top values are shown.

|  | **Predicted E_FRET_** | | | | | | | | | |  |
| --- | --- | --- | --- | --- | --- | --- | --- | --- | --- | --- | --- |
| **Model** | **1** | **2** | **3** | **4** | **5** | **6** | **7** | **8** | **9** | **10** | **Experimental Data** |
| **Core-P1** | 0.23 | 0.12 | 0.12 | 0.1 | 0.1 | 0.09 | 0.1 | 0.5 | 0.85 | 0.1 | 0.6, 0.2 |
| **Core-P2** | 0.9 | 0.85 | 0.41 | 0.91 | 0.37 | 0.51 | 0.42 | 0.65 | 0.82 | 0.75 | 0.3, 0.6 |
| **P1-P2** | 0.9 | 0.34 | 0.45 | 0.17 | 0.97 | 0.55 | 0.89 | 0.44 | 0.25 | 0.3 | 0.2, 0.5 |

**Supplementary Table 7: Crosslinks detected between SurA and OmpX.** List of detected residues that were crosslinked with DSBU and the corresponding domains in SurA. A representative mass spectrum for each crosslink can be found in Supplementary Data 1.

| Domain | SurA residue | OmpX residue |
| --- | --- | --- |
| N | 87 | 50 |
|  | 87 | 82 |
|  | 91 | 82 |
|  | 106 | 82 |
|  | 135 | 50 |
|  | 135 | 71 |
|  | 135 | 82 |
| P1 | 253 | 71 |
|  | 253 | 82 |
| P2 | 279 | 50 |
|  | 279 | 82 |
|  | 294 | 50 |
|  | 294 | 71 |
|  | 294 | 82 |
|  | 307 | 50 |
|  | 307 | 82 |
|  | 316 | 82 |
| C | 389 | 50 |
|  | 389 | 71 |
|  | 389 | 82 |
|  | 395 | 50 |
|  | 395 | 71 |
|  | 395 | 82 |
|  | 406 | 71 |
|  | 406 | 82 |
|  | 425 | 82 |

**Supplementary Table 8:** **Modified SurA peptides identified from photocrosslinking experiments between SurA and MTS-diazirine-conjugated single Cys OmpX variants.** SurA residues found to be modified are shown in red. Where the spectral quality of the MS/MS spectra was insufficient to conclusively assign the modified residue, the residues to which the modification could be localised are shown in red. A representative mass spectrum for each crosslink can be found in Supplementary Data 1.

| Sequence | Start Residue | End Residue | Modified Residue | OmpX Cys-variant |
| --- | --- | --- | --- | --- |
| VAAVVNNGVVLESDVDGL**M**QSV | 28 | 49 | M46 | M41C, I102C, K122C, V167C |
| **I**MDQIILQMGQK | 75 | 86 | I75 | M41C, I102C |
| I**M**DQIILQMGQK | 75 | 86 | M76 | M41C, I102C, V167C |
| LIMDQIILQ**MG**Q | 74 | 85 | M83-G84 | M41C, I102C, V167C |
| **IMDQ**IILQMGQ | 75 | 85 | I75-Q78 | K122C, V167C |
| **S**DEQLDQAIANIAK | 92 | 105 | S92 | I102C |
| SDEQ**LDQ**AIANIAK | 92 | 105 | L96-Q98 | V167C |
| LA**Y**DGLNYNTYR | 118 | 129 | Y120 | M41C, M41C*, I102C, K122C* |
| **E**MIISEVR | 135 | 142 | E135 | M41C*, I102C*, V167C* |
| EMIIS**E**VR | 135 | 142 | E140 | K122C*, V167C* |
| IQ**E**LPGIFAQALSTAK | 236 | 251 | E238 | M41C |
| **Q**ELPGIFAQALSTAK | 237 | 251 | Q237 | K122C |

* denotes identification in a sample enriched using thiopropyl Sepharose 6B resin

**Supplementary Table 9: HDX Data Summary Table.** SD = standard deviation, CI = confidence interval.

| **Data Set** | **SurA** | **SurA OmpX** | **SurA OmpF** | **SurA (no Urea)** | **SurA + WEYIPNV** |
| --- | --- | --- | --- | --- | --- |
| **HDX reaction details** | 10 mM potassium phosphate pD 8.0, 0.24 M d_4_-urea, 82 % D_2_O, 4 ºC | 10 mM potassium phosphate pD 8.0, 0.24 M d_4_-urea, 82 % D_2_O, 4 ºC | 10 mM potassium phosphate pD 8.0, 0.24 M d_4_-urea, 82 % D_2_O, 4 ºC | 10 mM potassium phosphate pD 8.0, 82 % D_2_O, 4 ºC | 10 mM potassium phosphate pD 8.0, 82 % D_2_O, 4 ºC |
| **HDX time course (min)** | 0.5, 2, 30, 120 | | | | |
| **HDX control samples** | Maximally-labeled controls were not performed. | | | | |
| **Back-exchange** | ~ 30 % | | | | |
| **# of Peptides** | 250 | 250 | 250 | 244 | 244 |
| **Sequence coverage** | 100% | 100% | 100% | 100% | 100% |
| **Average peptide length / Redundancy** | 10.9 / 6.95 | 10.9 / 6.96 | 10.9 / 6.97 | 11.2 / 6.98 | 11.2 / 6.99 |
| **Replicates (biological or technical)** | 4 (technical) | 4 (technical) | 4 (technical) | 4 (technical) | 4 (technical) |
| **Repeatability** | 0.101 (average SD) | 0.089 (average SD) | 0.097 (average SD) | 0.102 (average SD) | 0.105 (average SD) |
| **Significant differences in HDX**  **(delta HDX > X D)** | Reference | 0.65 D (99% CI), 1.01 D (99 % CI) in summed data | 0.62 D (99% CI), 0.7 D (99 % CI) in summed data | Reference | 0.51 D (99 % CI), 1.03 D (99 % CI) in summed data |

**Supplementary References**

1. Robert X, Gouet P. Deciphering key features in protein structures with the new ENDscript server. *Nucleic Acids Res* **42**, W320-324 (2014).

2. Bitto E, McKay DB. Crystallographic structure of SurA, a molecular chaperone that facilitates folding of outer membrane porins. *Structure* **10**, 1489-1498 (2002).

3. Xu X, Wang S, Hu YX, McKay DB. The periplasmic bacterial molecular chaperone SurA adapts its structure to bind peptides in different conformations to assert a sequence preference for aromatic residues. *J Mol Biol* **373**, 367-381 (2007).

4. Giuseppe PO, Atzingen MV, Nascimento ALTO, Zanchin NIT, Guimarães BG. The crystal structure of the leptospiral hypothetical protein LIC12922 reveals homology with the periplasmic chaperone SurA. *J Struct Biol* **173**, 312-322 (2011).

5. Kale A, Phansopa C, Suwannachart C, Craven CJ, Rafferty JB, Kelly DJ. The Virulence Factor PEB4 (Cj0596) and the Periplasmic Protein Cj1289 Are Two Structurally Related SurA-like Chaperones in the Human Pathogen *Campylobacter jejuni*. *J Biol Chem* **286**, 21254-21265 (2011).

6. Humes JR, Bob Schiffrin, Calabrese AN, Higgins AJ, Westhead DR, Brockwell DJ, Radford SE. The Role of SurA PPIase Domains in Preventing Aggregation of the Outer Membrane Proteins tOmpA and OmpT. *J Mol Biol* **431**, 1267-1283 (2019).

7. Horne JE, Walko M, Calabrese AN, Levenstein MA, Brockwell DJ, Kapur N, Wilson AJ, Radford SE. Rapid Mapping of Protein Interactions Using Tag-Transfer Photocrosslinkers. *Angew Chem Int Ed Engl* **57**, 16688-16692 (2018).

8. Baker NA, Sept D, Joseph S, Holst MJ, McCammon JA. Electrostatics of nanosystems: Application to microtubules and the ribosome. *Proc Natl Acad Sci* **98**, 10037-10041 (2001).

9. Ashkenazy H, Abadi S, Martz E, Chay O, Mayrose I, Pupko T, Ben-Tal N. ConSurf 2016: an improved methodology to estimate and visualize evolutionary conservation in macromolecules. *Nucleic Acids Res* **44**, W344-W350 (2016).

10. Chaturvedi D, Mahalakshmi R. Folding Determinants of Transmembrane β-Barrels Using Engineered OMP Chimeras. *Biochemistry* **57**, 1987-1996 (2018).

11. Hiller S, Wider G, Imbach LL, Wuthrich K. Interactions with hydrophobic clusters in the urea-unfolded membrane protein OmpX. *Angew Chem Int Ed Engl* **47**, 977-981 (2008).

12. Michalik M, Orwick-Rydmark M, Habeck M, Alva V, Arnold T, Linke D. An evolutionarily conserved glycine-tyrosine motif forms a folding core in outer membrane proteins. *PLOS ONE* **12**, e0182016 (2017).

13. Kutik S, Stojanovski D, Becker L, Becker T, Meinecke M, Kruger V, Prinz C, Meisinger C, Guiard B, Wagner R, Pfanner N, Wiedemann N. Dissecting membrane insertion of mitochondrial beta-barrel proteins. *Cell* **132**, 1011-1024 (2008).

14. Bitto E, McKay DB. The periplasmic molecular chaperone protein SurA binds a peptide motif that is characteristic of integral outer membrane proteins. *J Biol Chem* **278**, 49316-49322 (2003).

15. Bitto E, McKay DB. Binding of phage-display-selected peptides to the periplasmic chaperone protein SurA mimics binding of unfolded outer membrane proteins. *FEBS Lett* **568**, 94-98 (2004).

16. Hennecke G, Nolte J, Volkmer-Engert R, Schneider-Mergener J, Behrens S. The periplasmic chaperone SurA exploits two features characteristic of integral outer membrane proteins for selective substrate recognition. *J Biol Chem* **280**, 23540-23548 (2005).

17. Marx DC, Plummer AM, Faustino AM, Roskopf MA, Leblanc MJ, Devlin T, Lessen HJ, Majumdar A, Amann BT, Fleming PJ, Krueger S, Fried SD, Fleming KG. SurA is a “Groove-y” Chaperone That Expands Unfolded Outer Membrane Proteins. Preprint at <https://www.biorxiv.org/content/10.1101/2019.12.17.878660v1> (2019).

18. Hellenkamp B, Schmid S, Doroshenko O, Opanasyuk O, Kuhnemuth R, Rezaei Adariani S, Ambrose B, Aznauryan M, Barth A, Birkedal V, Bowen ME, Chen H, Cordes T, Eilert T, Fijen C, Gebhardt C, Gotz M, Gouridis G, Gratton E, Ha T, Hao P, Hanke CA, Hartmann A, Hendrix J, Hildebrandt LL, Hirschfeld V, Hohlbein J, Hua B, Hubner CG, Kallis E, Kapanidis AN, Kim JY, Krainer G, Lamb DC, Lee NK, Lemke EA, Levesque B, Levitus M, McCann JJ, Naredi-Rainer N, Nettels D, Ngo T, Qiu R, Robb NC, Rocker C, Sanabria H, Schlierf M, Schroder T, Schuler B, Seidel H, Streit L, Thurn J, Tinnefeld P, Tyagi S, Vandenberk N, Vera AM, Weninger KR, Wunsch B, Yanez-Orozco IS, Michaelis J, Seidel CAM, Craggs TD, Hugel T. Precision and accuracy of single-molecule FRET measurements-a multi-laboratory benchmark study. *Nat Methods* **15**, 669-676 (2018).

19. Tomov TE, Tsukanov R, Masoud R, Liber M, Plavner N, Nir E. Disentangling subpopulations in single-molecule FRET and ALEX experiments with photon distribution analysis. *Biophys J* **102**, 1163-1173 (2012).

20. Hagelueken G, Ward R, Naismith JH, Schiemann O. MtsslWizard: In Silico Spin-Labeling and Generation of Distance Distributions in PyMOL. *Appl Magn Reson* **42**, 377-391 (2012).

21. Sinz A. Cross-Linking/Mass Spectrometry for Studying Protein Structures and Protein–Protein Interactions: Where Are We Now and Where Should We Go from Here? *Angew Chem Int Ed* **57**, 6390-6396 (2018).

22. Eswar N, Webb B, Marti-Renom MA, Madhusudhan MS, Eramian D, Shen MY, Pieper U, Sali A. Comparative protein structure modeling using Modeller. *Curr Protoc Bioinformatics* **Chapter 5, Unit 5.6**, 5.6.1-5.6.30, <https://doi.org/10.1002/0471250953.bi0471250506s0471250915> (2006).
