# Supplementary material for: Inter-domain dynamics in the chaperone SurA and multi-site binding to its outer membrane protein clients
Source: Nat Commun. 2020 May 1;11:2155. doi: 10.1038/s41467-020-15702-1 (PMC7195389; doi:10.1038/s41467-020-15702-1)
Supplement: Supplementary file 2 — Description of Additional Supplementary Files [file 41467_2020_15702_MOESM2_ESM.docx]

**Supplementary Movie 1:**

**First repeat of a MD simulation of full-length SurA using a SurA^core-P1-open^ model as the starting structure.** 1 μs all-atom simulations of the mature sequence of SurA (residues 21-428) in explicit solvent were performed with GROMACS 5.0.2 using the CHARMM36 force field. The SurA^core-P1-open^ model was built using the crystal structures of full-length SurA (PDB: 1M5Y) and SurA-ΔP2 (PDB: 2PV3) in which the P1 domain is extended away from the core (see Methods). The N-terminal region of the core domain, P1, P2 and the C-terminal region of the core domain are shown in grey, green, yellow, and orange, respectively.

**Supplementary Movie 2:**

**Second repeat of a MD simulation of full-length SurA using a SurA^core-P1-open^ model as the starting structure.** 1 μs all-atom simulations of the mature sequence of SurA (residues 21-428) in explicit solvent were performed with GROMACS 5.0.2 using the CHARMM36 force field. The SurA^core-P1-open^ model was built using the crystal structures of full-length SurA (PDB: 1M5Y) and SurA-ΔP2 (PDB: 2PV3) in which the P1 domain is extended away from the core (see Methods). The N-terminal region of the core domain, P1, P2 and the C-terminal region of the core domain are shown in grey, green, yellow, and orange, respectively.

**Supplementary Movie 3:**

**Third repeat of a MD simulation of full-length SurA using a SurA^core-P1-open^ model as the starting structure.** 1 μs all-atom simulations of the mature sequence of SurA (residues 21-428) in explicit solvent were performed with GROMACS 5.0.2 using the CHARMM36 force field. The SurA^core-P1-open^ model was built using the crystal structures of full-length SurA (PDB: 1M5Y) and SurA-ΔP2 (PDB: 2PV3) in which the P1 domain is extended away from the core (see Methods). The N-terminal region of the core domain, P1, P2 and the C-terminal region of the core domain are shown in grey, green, yellow, and orange, respectively.

**Supplementary Data 1:**

**Representative spectra of all detected crosslinked peptides.** Spectra of intra-domain/inter-domain crosslinks detected in DSBU crosslinked SurA, Spectra of all DSBU crosslinks detected between SurA and OmpX. XL-MS spectra of tag-transfer crosslinks detected between SurA and OmpX.

**Supplementary Data 2:**

**XL-MS experimental design spreadsheet.**
