# Supplementary material for: Inter-domain dynamics in the chaperone SurA and multi-site binding to its outer membrane protein clients
Source: Nat Commun. 2020 May 1;11:2155. doi: 10.1038/s41467-020-15702-1 (PMC7195389; doi:10.1038/s41467-020-15702-1)
Supplement: Supplementary file 6 — Supplementary Data 1 [file 41467_2020_15702_MOESM6_ESM.pdf]

XL-MS spectra of intra-domain crosslinks detected in DSBU crosslinked SurA.

K86-K134

theor.Mass(M+H+):3245.749
PrecursorMass(M+H+):3245.74
Deviation-2.79ppm
m/z:812.1905
Charge:+4

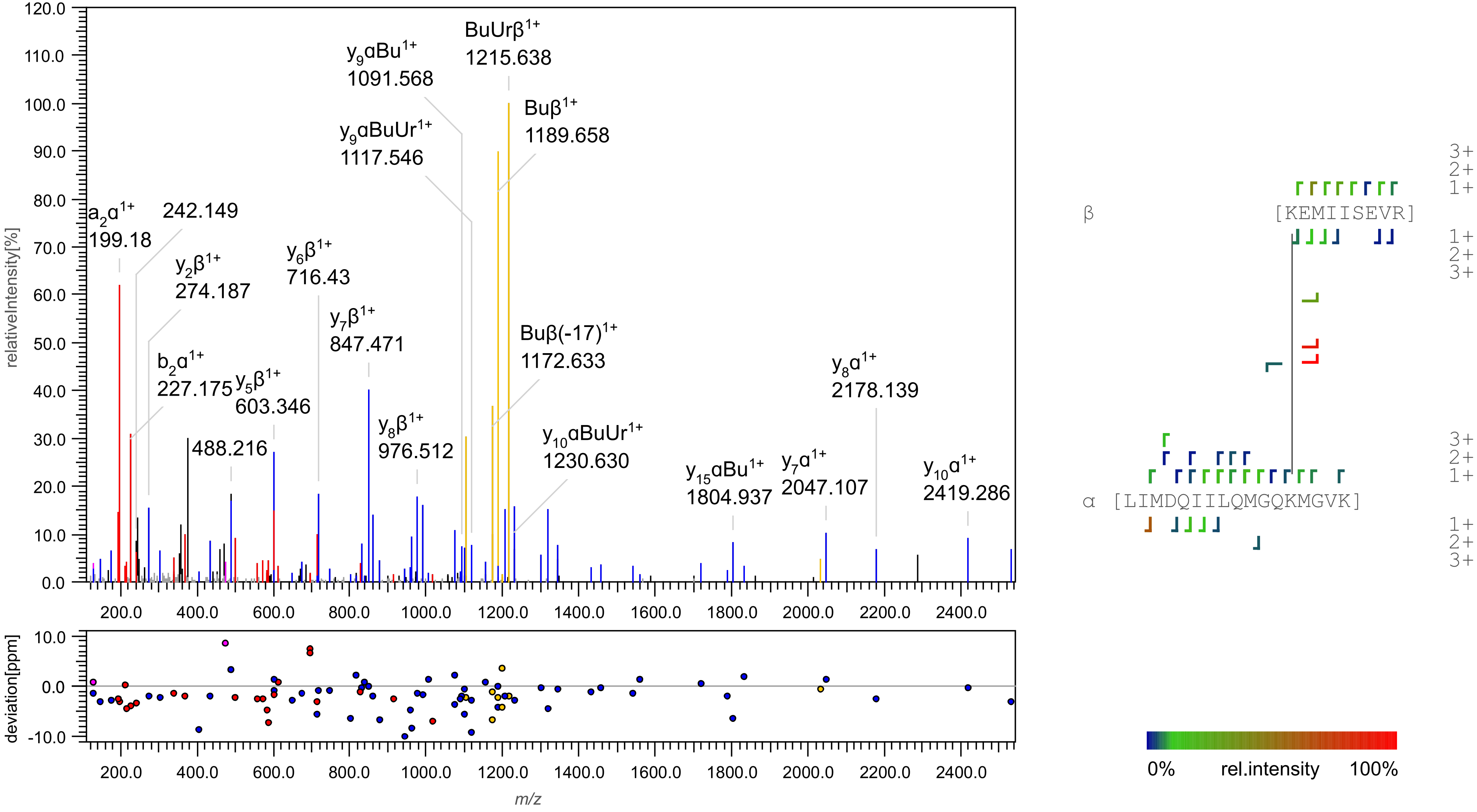

K90-K134

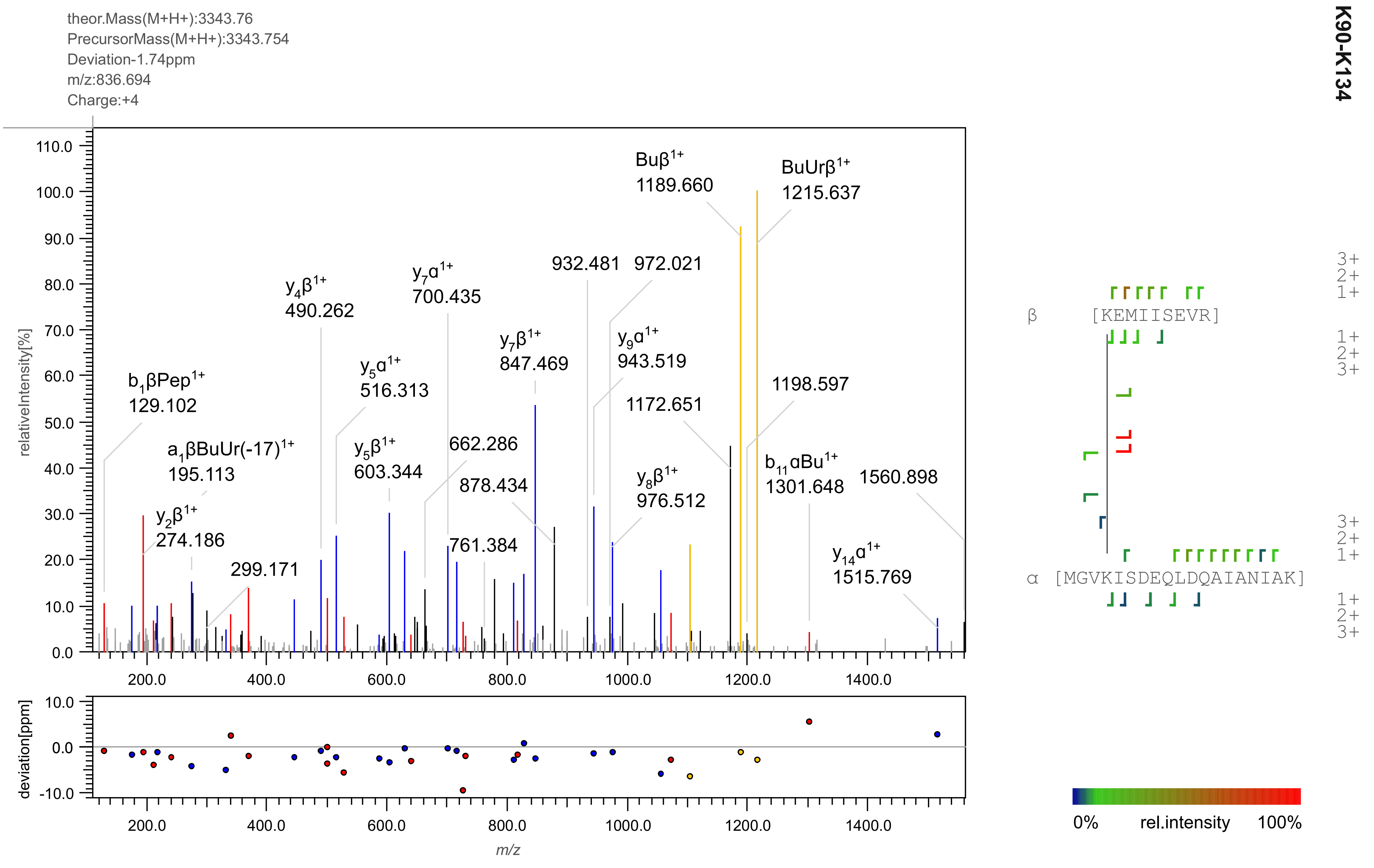

theor.Mass(M+H+):3343.76
PrecursorMass(M+H+):3343.754
Deviation-1.74ppm
m/z:836.694
Charge:+4
relativeIntensity[%]
deviation[ppm]
m/z
b1βPep1+ 129.102
a1βBuUr(-17)1+ 195.113
y2β1+ 274.186
299.171
y4β1+ 490.262
y5α1+ 516.313
y5β1+ 603.344
662.286
y7α1+ 700.435
761.384
y7β1+ 847.469
878.434
932.481
y9α1+ 943.519
972.021
y8β1+ 976.512
1172.651
Buβ1+ 1189.660
1198.597
BuUrβ1+ 1215.637
b11αBu1+ 1301.648
y14α1+ 1515.769
1560.898
β [KEMIISEVR]
α [MGVKISDEQLDQAIANIAK]
3+ 2+ 1+
0% rel.intensity 100%

K134-K394

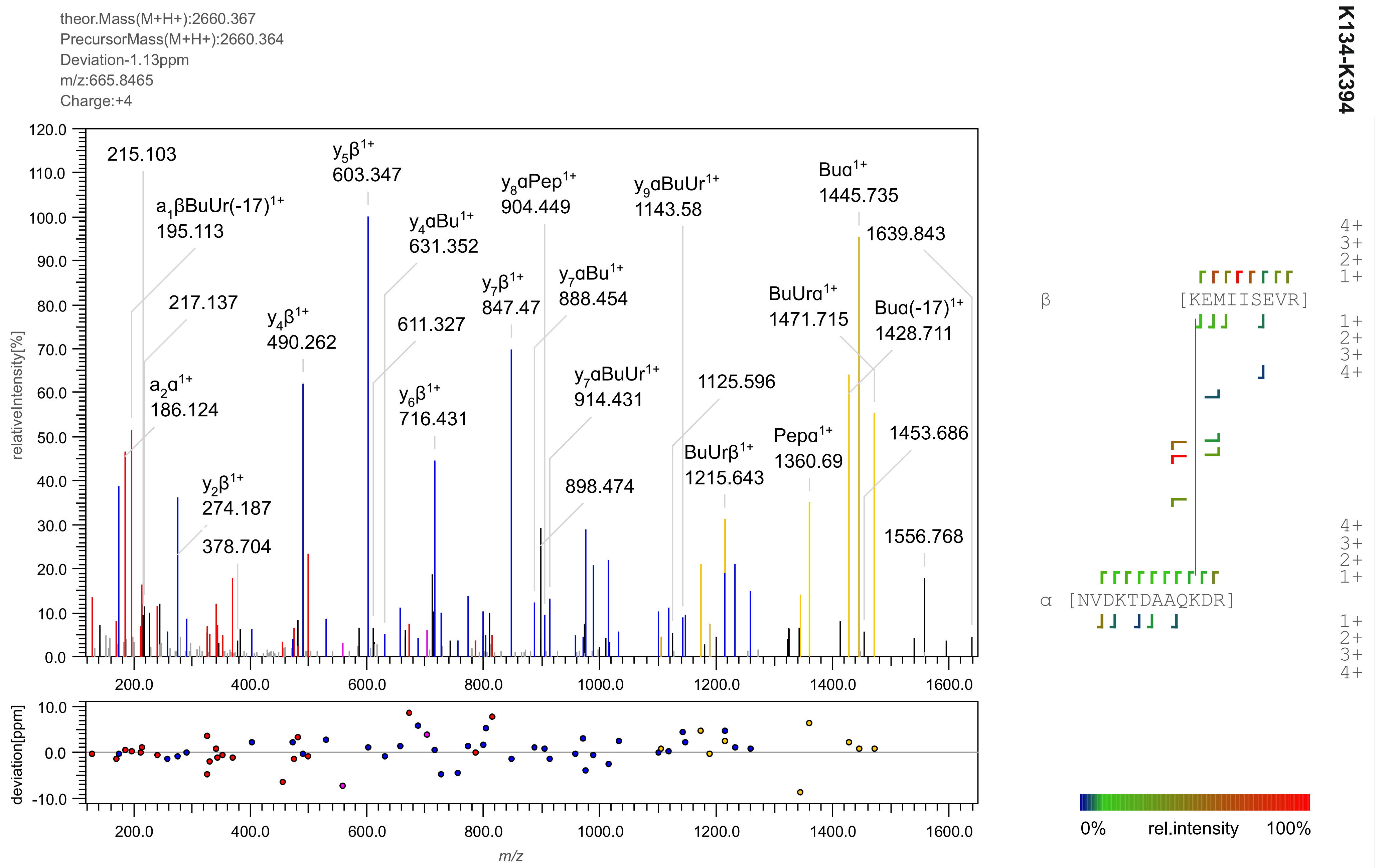

theor.Mass(M+H+):2660.367
PrecursorMass(M+H+):2660.364
Deviation-1.13ppm
m/z:665.8465
Charge:+4
215.103
a1βBuUr(-17)1+ 195.113
217.137
a2α1+ 186.124
y2β1+ 274.187
378.704
y4β1+ 490.262
y5β1+ 603.347
y4αBu1+ 631.352
611.327
y6β1+ 716.431
y7β1+ 847.47
y8αPep1+ 904.449
y7αBu1+ 888.454
y7αBuUr1+ 914.431
898.474
y9αBuUr1+ 1143.58
1125.596
BuUrβ1+ 1215.643
Pepα1+ 1360.69
BuUrα1+ 1471.715
Buα1+ 1445.735
1639.843
Buα(-17)1+ 1428.711
1453.686
1556.768
relativeIntensity[%]
deviation[ppm]
m/z
β [KEMIISEVR]
α [NVDKTDAAQKDR]
1+ 2+ 3+ 4+
0% rel.intensity 100%

K134-K405

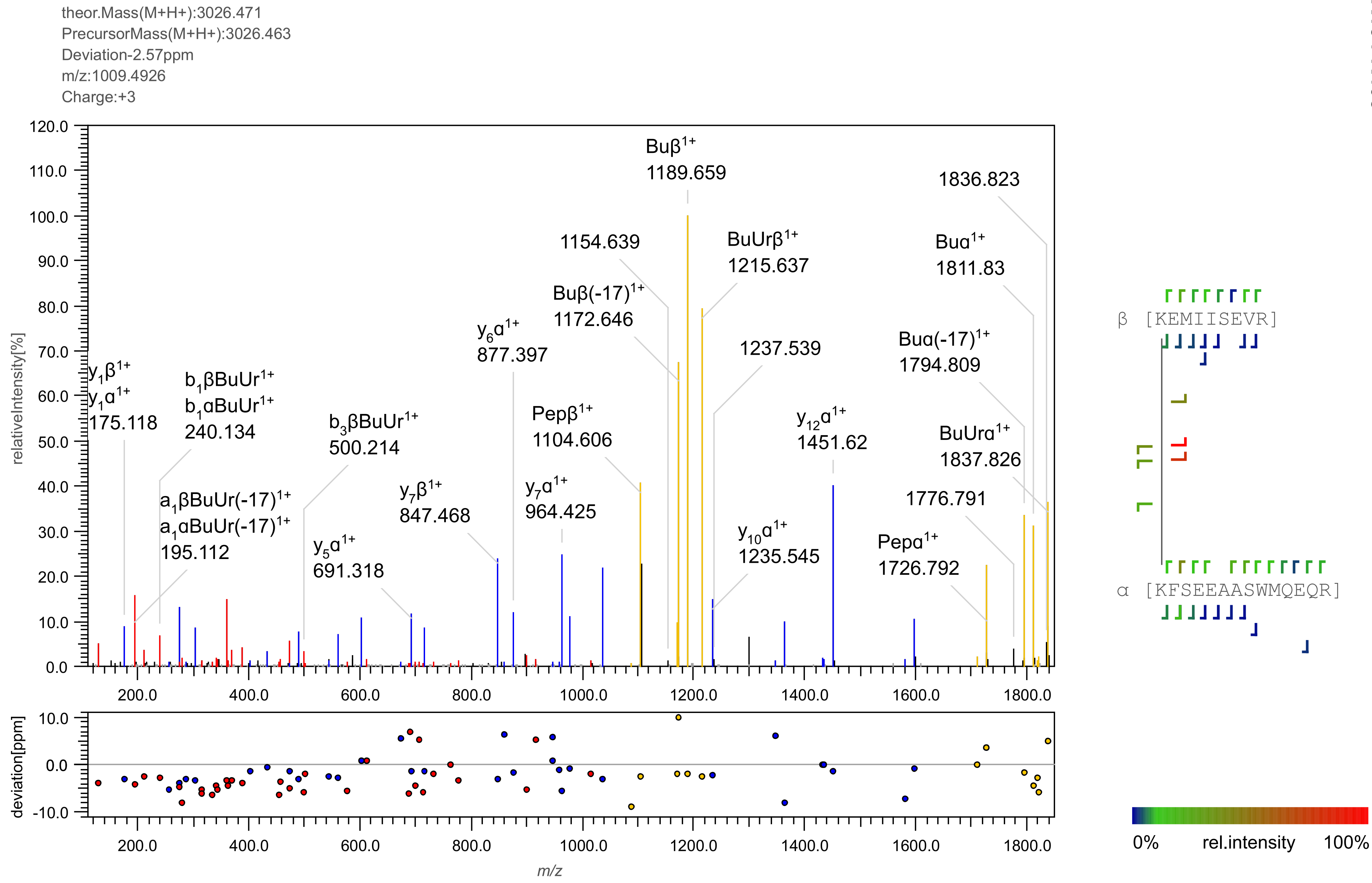
theor.Mass(M+H+):3026.471
PrecursorMass(M+H+):3026.463
Deviation-2.57ppm
m/z:1009.4926
Charge:+3
relativeIntensity[%]
deviation[ppm]
m/z
y1β1+
y1α1+
175.118
b1βBuUr1+
b1αBuUr1+
240.134
a1βBuUr(-17)1+
a1αBuUr(-17)1+
195.112
b3βBuUr1+
500.214
y5α1+
691.318
y7β1+
847.468
y6α1+
877.397
y7α1+
964.425
Pepβ1+
1104.606
Buβ(-17)1+
1172.646
1154.639
Buβ1+
1189.659
BuUrβ1+
1215.637
1237.539
y10α1+
1235.545
y12α1+
1451.62
Pepα1+
1726.792
1776.791
BuUrα1+
1837.826
Buα(-17)1+
1794.809
Buα1+
1811.83
1836.823
β [KEMIISEVR]
α [KFSEEAASWMQEQR]
1+
2+
3+
0%
rel.intensity
100%

K252-K269

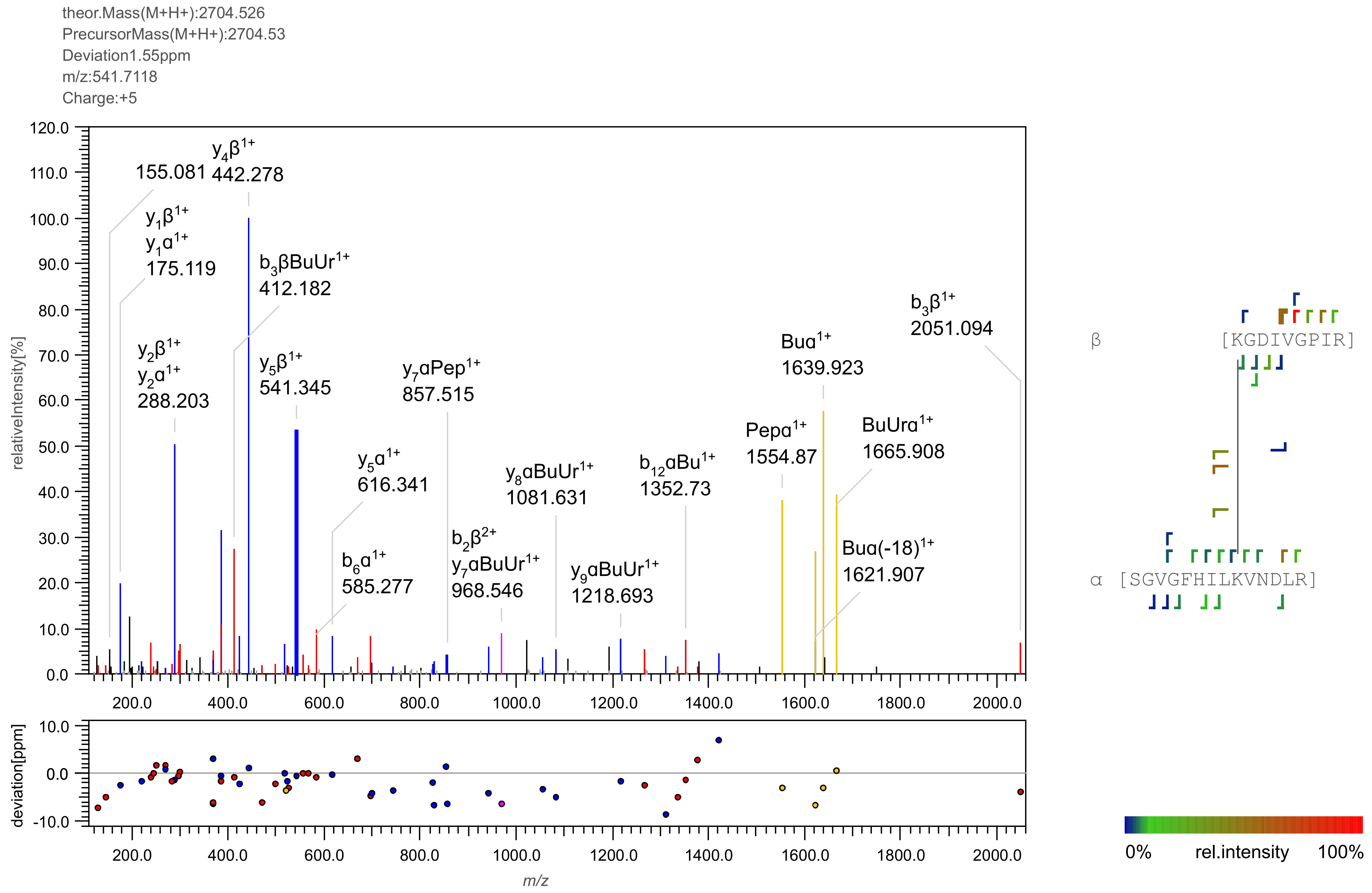
theor.Mass(M+H+):2704.526
PrecursorMass(M+H+):2704.53
Deviation1.55ppm
m/z:541.7118
Charge:+5
relativeIntensity[%]
deviation[ppm]
m/z
155.081
y4β1+ 442.278
y1β1+
y1α1+
175.119
b3βBuUr1+
412.182
y2β1+
y2α1+
288.203
y5β1+
541.345
y7αPep1+
857.515
y5α1+
616.341
b6α1+
585.277
b2β2+
y7αBuUr1+
968.546
y8αBuUr1+
1081.631
y9αBuUr1+
1218.693
b12αBu1+
1352.73
Pepα1+
1554.87
Buα1+
1639.923
BuUrα1+
1665.908
Buα(-18)1+
1621.907
b3β1+
2051.094
β [KGDIVGPIR]
α [SGVGFHILKVNDLR]
2+
1+
0%
rel.intensity
100%

K278-K293

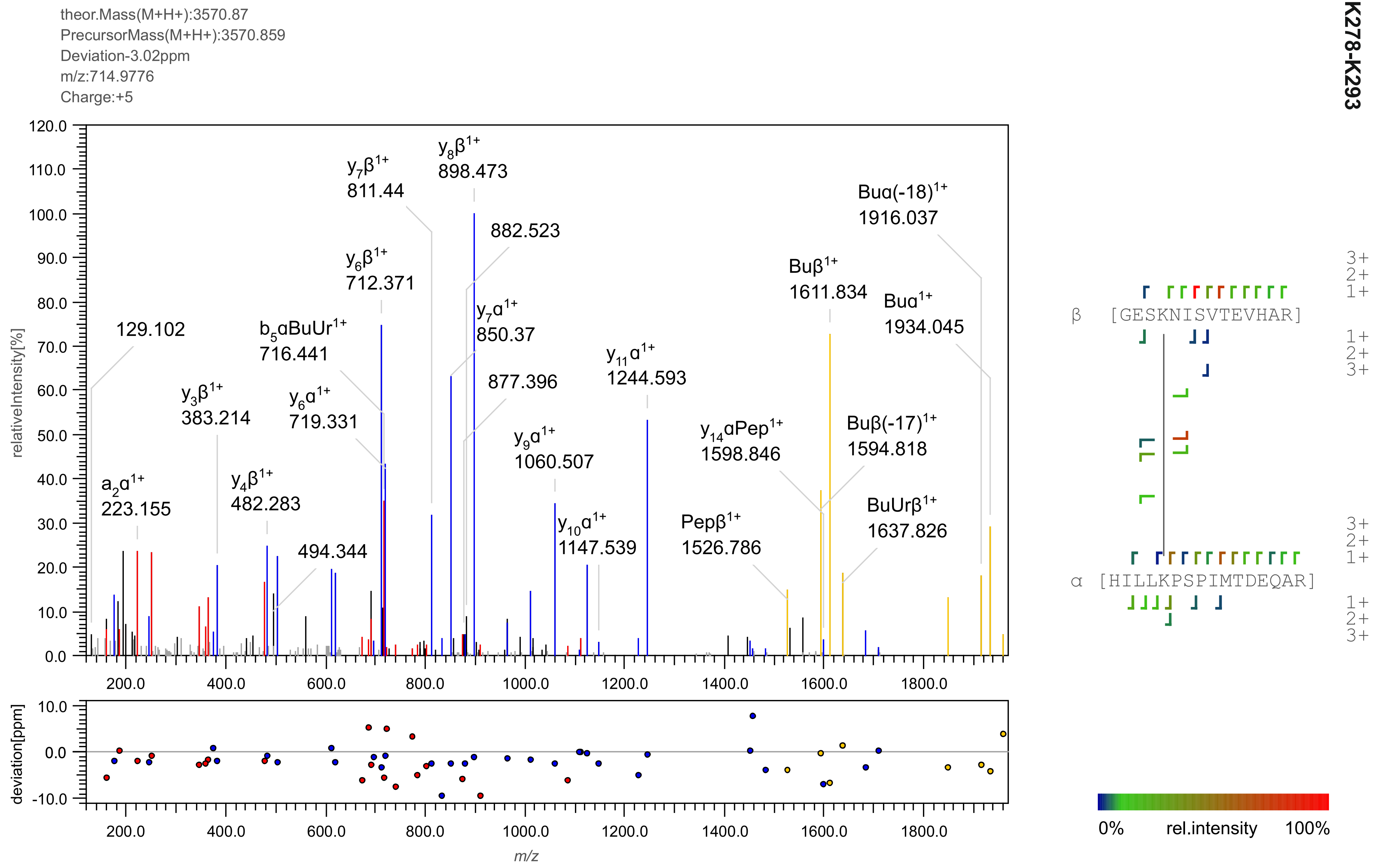
theor.Mass(M+H+):3570.87
PrecursorMass(M+H+):3570.859
Deviation-3.02ppm
m/z:714.9776
Charge:+5
relativeIntensity[%]
deviation[ppm]
m/z
129.102
a2α1+ 223.155
y3β1+ 383.214
y4β1+ 482.283
494.344
b5αBuUr1+ 716.441
y6α1+ 719.331
y6β1+ 712.371
y7β1+ 811.44
y8β1+ 898.473
882.523
y7α1+ 850.37
877.396
y9α1+ 1060.507
y10α1+ 1147.539
y11α1+ 1244.593
y14αPep1+ 1598.846
Pepβ1+ 1526.786
Buβ1+ 1611.834
Buβ(-17)1+ 1594.818
BuUrβ1+ 1637.826
Buα(-18)1+ 1916.037
Buα1+ 1934.045
β [GESKNISVTEVHAR]
α [HILLKPSPIMTDEQAR]
3+
2+
1+
0%
rel.intensity
100%

K278-K362

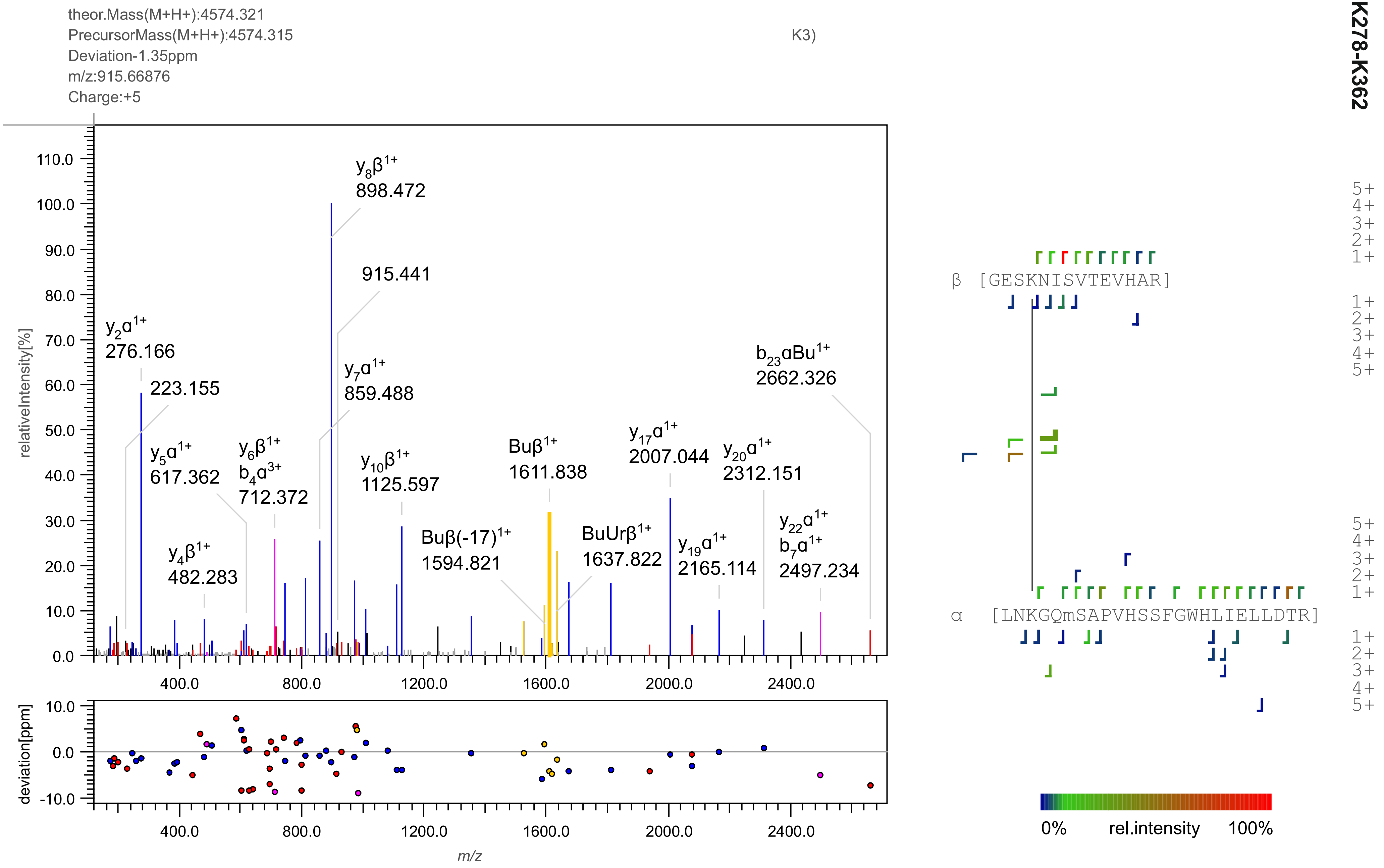
theor.Mass(M+H+):4574.321
PrecursorMass(M+H+):4574.315
Deviation-1.35ppm
m/z:915.66876
Charge:+5
K3)
relativeIntensity[%]
deviation[ppm]
m/z
y2α1+ 276.166
223.155
y5α1+ 617.362
y4β1+ 482.283
y6β1+ b4α3+ 712.372
y8β1+ 898.472
915.441
y7α1+ 859.488
y10β1+ 1125.597
Buβ(-17)1+ 1594.821
Buβ1+ 1611.838
BuUrβ1+ 1637.822
y17α1+ 2007.044
y19α1+ 2165.114
y20α1+ 2312.151
y22α1+ b7α1+ 2497.234
b23αBu1+ 2662.326
β [GESKNISVTEVHAR]
α [LNKGQmSAPVHSSFGWHLIELLDTR]
5+ 4+ 3+ 2+ 1+
1+ 2+ 3+ 4+ 5+
0% rel.intensity 100%

K278-K388

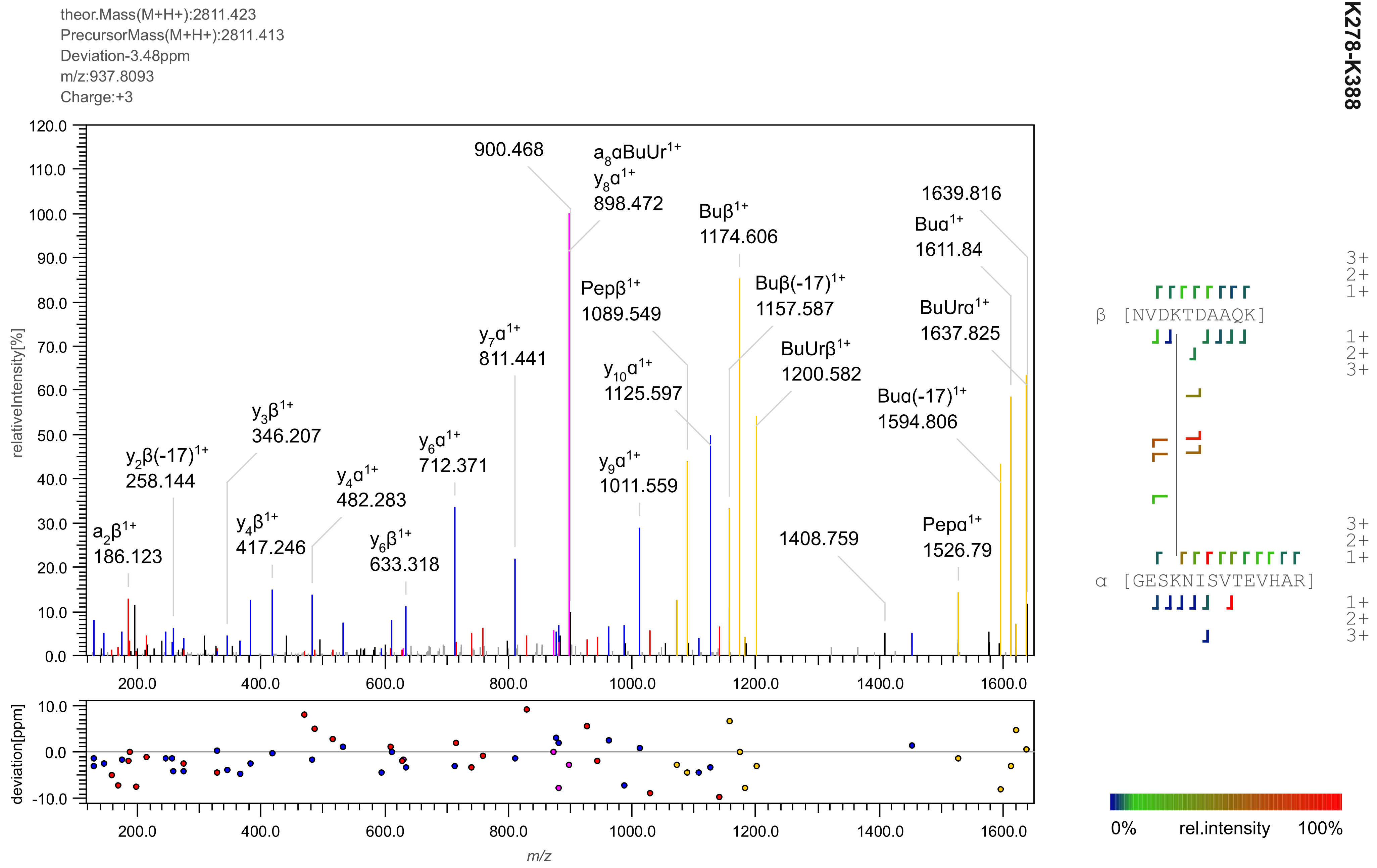

theor.Mass(M+H+):2811.423
PrecursorMass(M+H+):2811.413
Deviation-3.48ppm
m/z:937.8093
Charge:+3
relativeIntensity[%]
deviation[ppm]
m/z
900.468
a8αBuUr1+ y8α1+ 898.472
Buβ1+ 1174.606
1639.816
Buα1+ 1611.84
Pepβ1+ 1089.549
Buβ(-17)1+ 1157.587
BuUrα1+ 1637.825
y7α1+ 811.441
y10α1+ 1125.597
BuUrβ1+ 1200.582
Buα(-17)1+ 1594.806
y3β1+ 346.207
y6α1+ 712.371
y2β(-17)1+ 258.144
y4α1+ 482.283
y9α1+ 1011.559
a2β1+ 186.123
y4β1+ 417.246
y6β1+ 633.318
1408.759
Pepα1+ 1526.79
β [NVDKTDAAQK]
α [GESKNISVTEVHAR]
3+ 2+ 1+
1+ 2+ 3+
0% rel.intensity 100%

K293-K306

theor.Mass(M+H+):3271.808
PrecursorMass(M+H+):3271.804
Deviation-1.2ppm
m/z:818.70654
Charge:+4

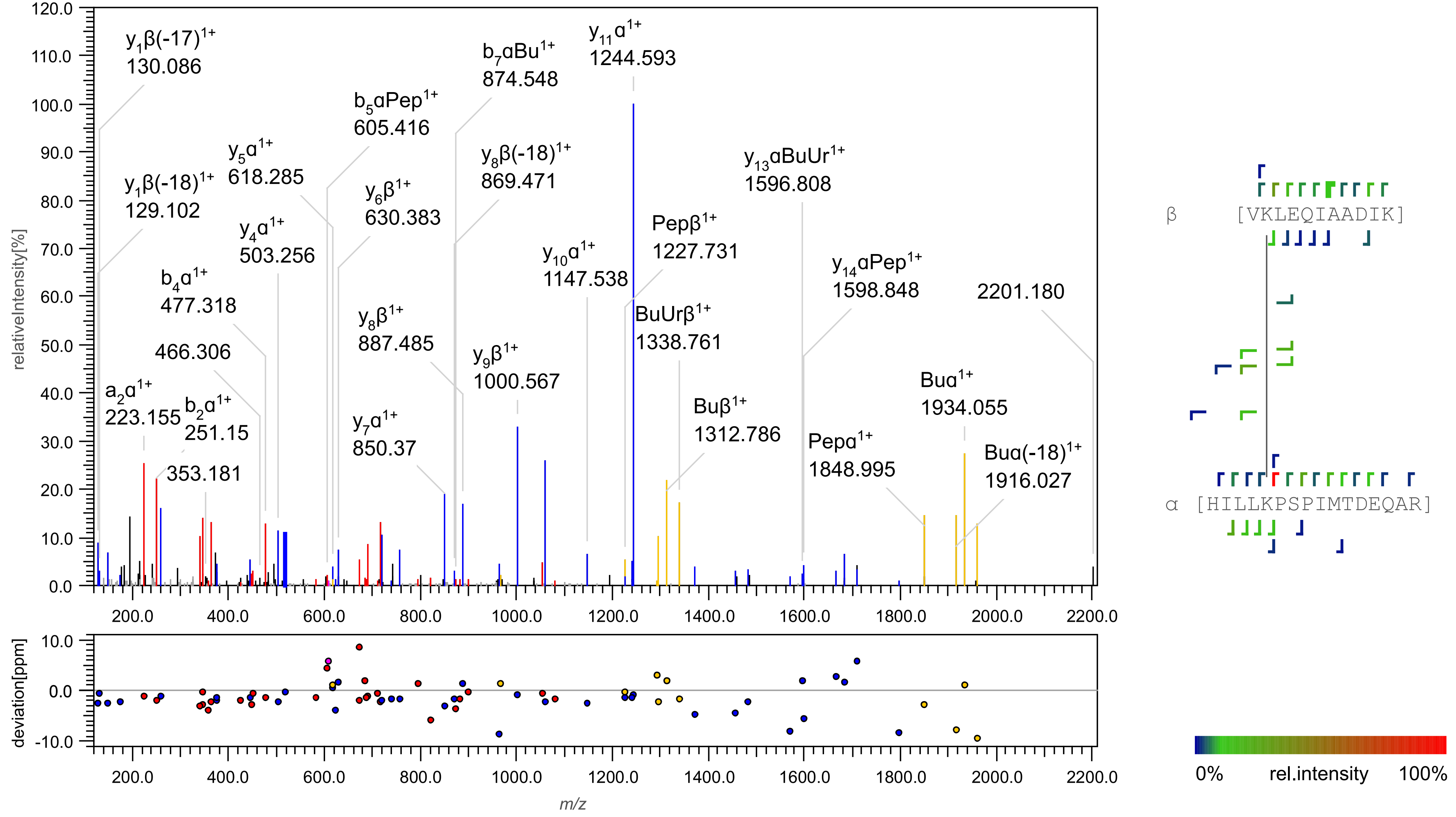

K306-K318

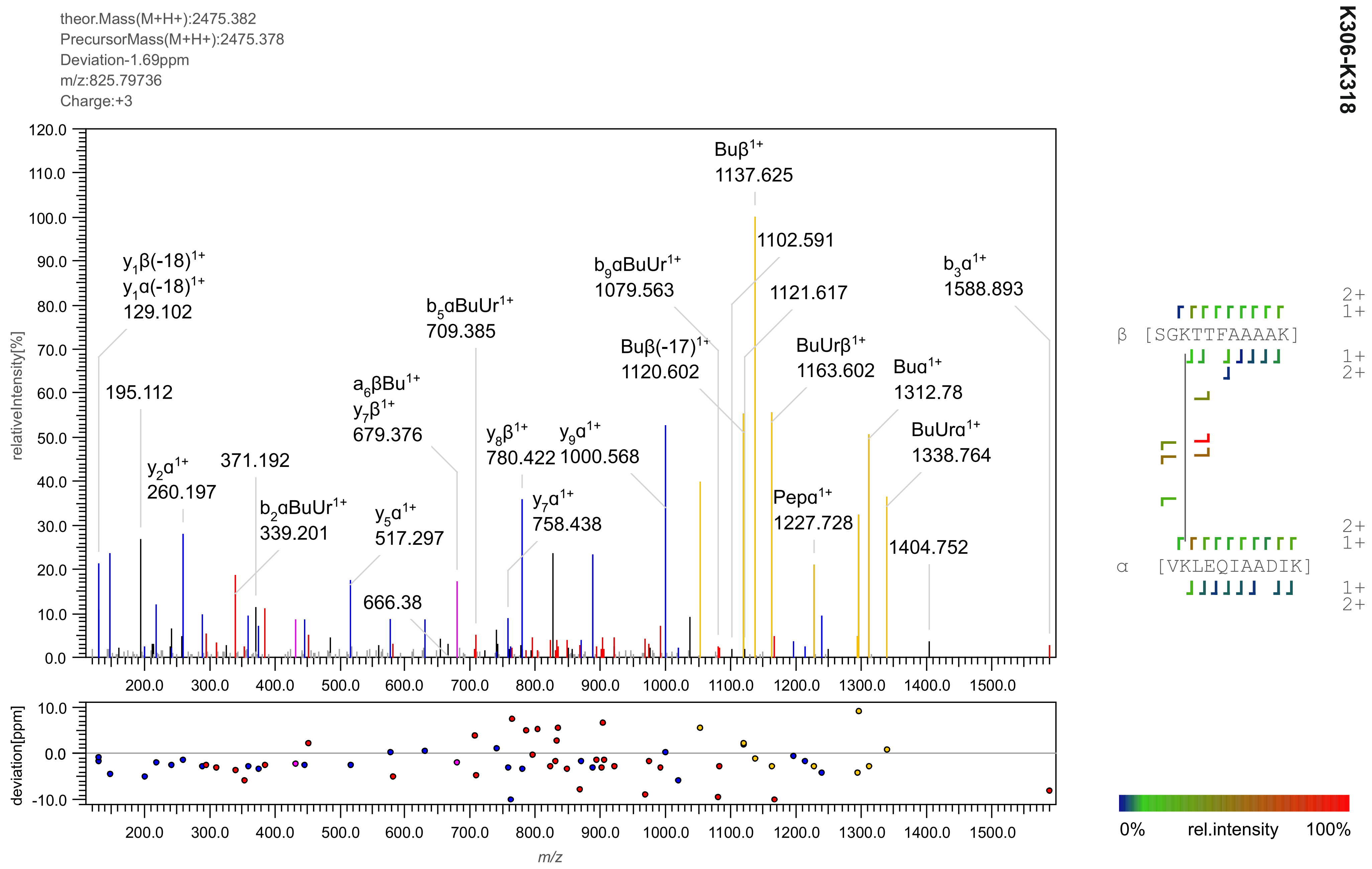

theor.Mass(M+H+):2475.382
PrecursorMass(M+H+):2475.378
Deviation-1.69ppm
m/z:825.79736
Charge:+3
relativeIntensity[%]
deviation[ppm]
m/z
y1β(-18)1+
y1α(-18)1+
129.102
195.112
y2α1+
260.197
371.192
b2αBuUr1+
339.201
y5α1+
517.297
666.38
a6βBu1+
y7β1+
679.376
b5αBuUr1+
709.385
y8β1+
780.422
y7α1+
758.438
y9α1+
1000.568
b9αBuUr1+
1079.563
Buβ(-17)1+
1120.602
Buβ1+
1137.625
1102.591
1121.617
BuUrβ1+
1163.602
Pepα1+
1227.728
Buα1+
1312.78
BuUrα1+
1338.764
1404.752
b3α1+
1588.893
β [SGKTTFAAAAK]
α [VKLEQIAADIK]
2+
1+
0%
rel.intensity
100%

K315-K362

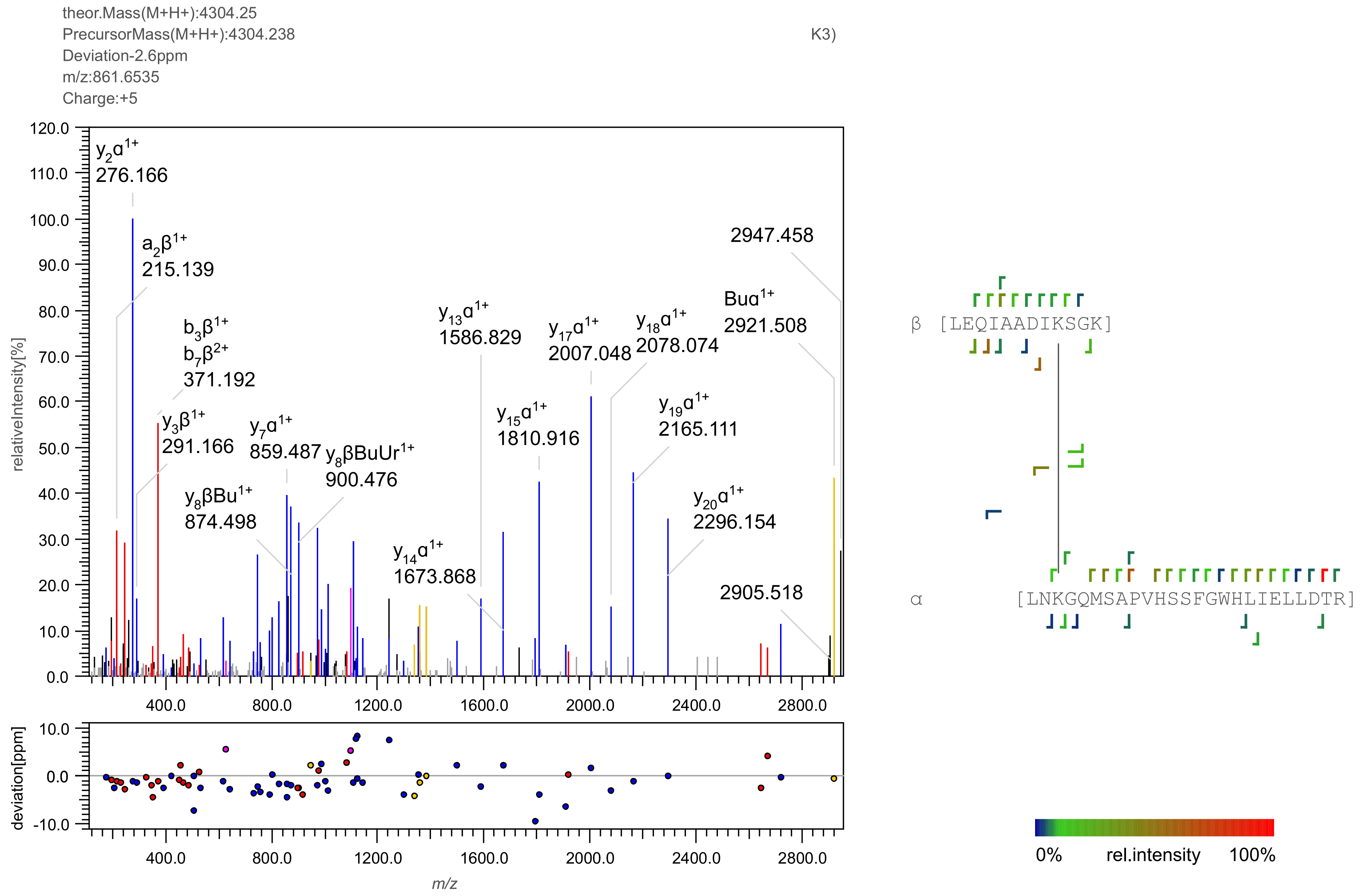
theor.Mass(M+H+):4304.25
PrecursorMass(M+H+):4304.238
Deviation-2.6ppm
m/z:861.6535
Charge:+5
K3)
relativeIntensity[%]
deviation[ppm]
m/z
$y_2α^{1+}$ 276.166
$a_2β^{1+}$ 215.139
$b_3β^{1+}$ $b_7β^{2+}$ 371.192
$y_3β^{1+}$ 291.166
$y_7α^{1+}$ 859.487
$y_8βBuUr^{1+}$ 900.476
$y_8βBu^{1+}$ 874.498
$y_{13}α^{1+}$ 1586.829
$y_{14}α^{1+}$ 1673.868
$y_{15}α^{1+}$ 1810.916
$y_{17}α^{1+}$ 2007.048
$y_{18}α^{1+}$ 2078.074
$y_{19}α^{1+}$ 2165.111
$y_{20}α^{1+}$ 2296.154
$Buα^{1+}$ 2921.508
2947.458
2905.518
β [LEQIAADIKSGK]
α [LNKGQMSAPVHSSFGWHLIELLDTR]
3+ 2+ 1+
1+ 2+ 3+
0% rel.intensity 100%

K362-K388

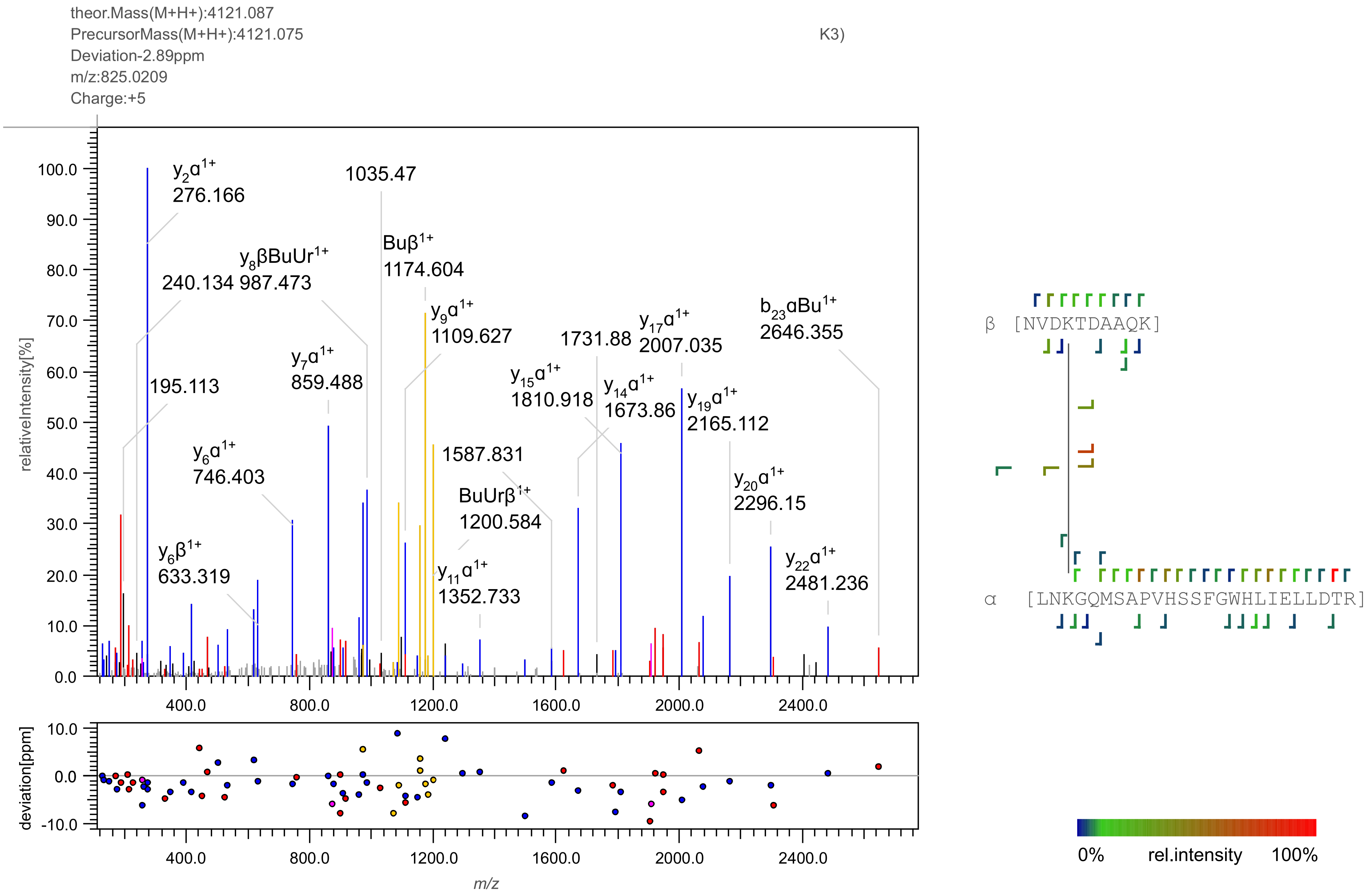
theor.Mass(M+H+):4121.087
PrecursorMass(M+H+):4121.075
Deviation-2.89ppm
m/z:825.0209
Charge:+5
K3)
relativeIntensity[%]
deviation[ppm]
m/z
$y_2α^{1+}$ 276.166
1035.47
$Buβ^{1+}$ 1174.604
$y_8βBuUr^{1+}$
240.134 987.473
$y_9α^{1+}$ 1109.627
$y_7α^{1+}$ 859.488
195.113
1731.88
$y_{17}α^{1+}$ 2007.035
$b_{23}αBu^{1+}$ 2646.355
$y_{15}α^{1+}$ 1810.918
$y_{14}α^{1+}$ 1673.86
$y_{19}α^{1+}$ 2165.112
$y_6α^{1+}$ 746.403
1587.831
$BuUrβ^{1+}$ 1200.584
$y_{20}α^{1+}$ 2296.15
$y_6β^{1+}$ 633.319
$y_{11}α^{1+}$ 1352.733
$y_{22}α^{1+}$ 2481.236
β [NVDKTDAAQK]
α [LNKGQMSAPVHSSFGWHLIELLDTR]
3+ 2+ 1+
1+ 2+ 3+
0% rel.intensity 100%

K394-K405

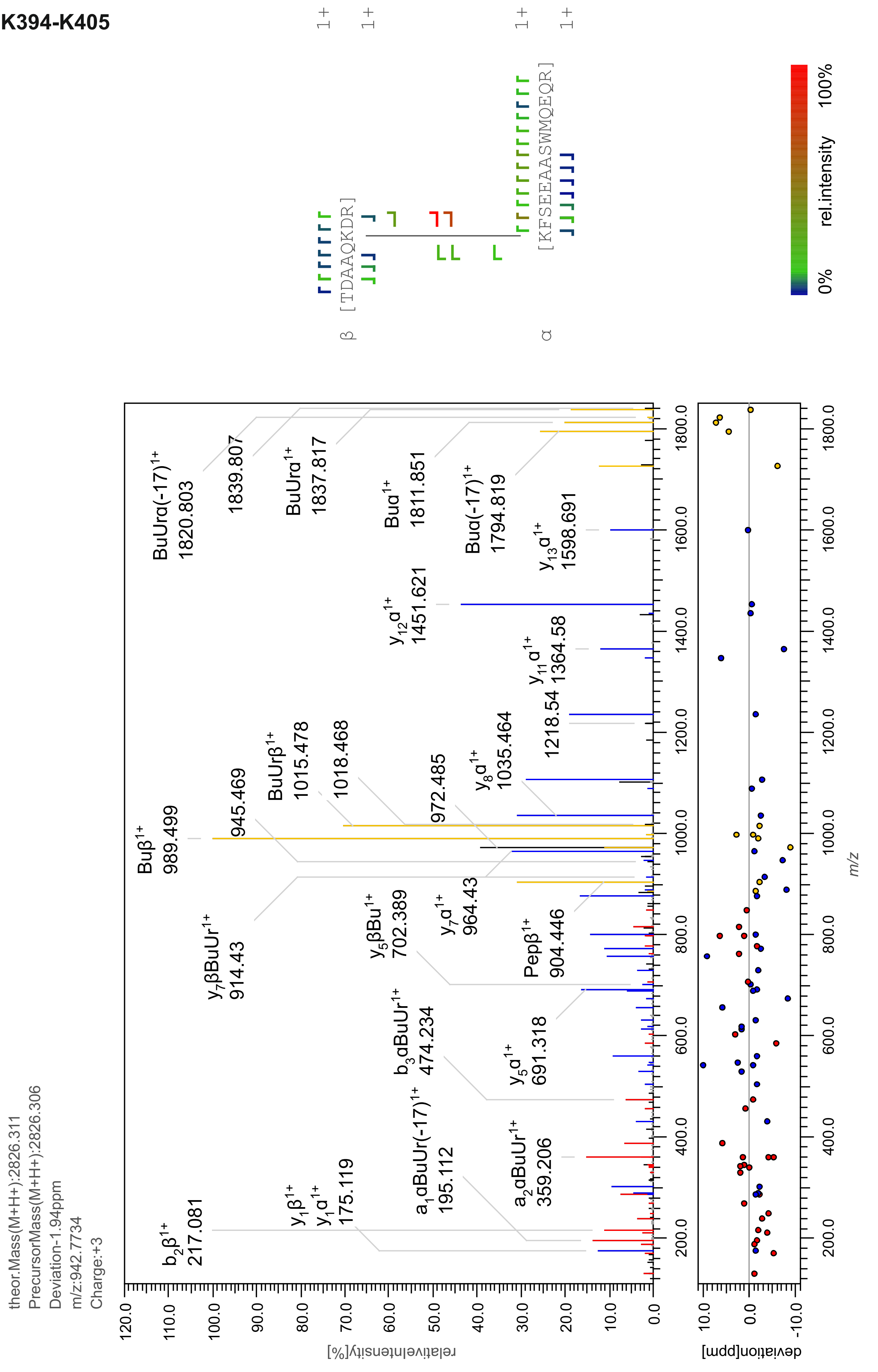

theor.Mass(M+H+):2826.311
PrecursorMass(M+H+):2826.306
Deviation-1.94ppm
m/z:942.7734
Charge:+3
relativeIntensity[%]
deviation[ppm]
m/z
b2β1+ 217.081
y1β1+ y1α1+ 175.119
a1αBuUr(-17)1+ 195.112
a2αBuUr1+ 359.206
b3αBuUr1+ 474.234
y5α1+ 691.318
y7βBuUr1+ 914.43
y5βBu1+ 702.389
y7α1+ 964.43
Pepβ1+ 904.446
Buβ1+ 989.499
945.469
BuUrβ1+ 1015.478
1018.468
972.485
y8α1+ 1035.464
1218.54
y11α1+ 1364.58
y12α1+ 1451.621
y13α1+ 1598.691
BuUrα(-17)1+ 1820.803
1839.807
BuUrα1+ 1837.817
Buα1+ 1811.851
Buα(-17)1+ 1794.819
β [TDAAQKDR] 1+
α [KFSEEAASWMQEQR] 1+
0% rel.intensity 100%

XL-MS spectra of inter-domain crosslinks detected in DSBU crosslinked SurA.

K105-K278

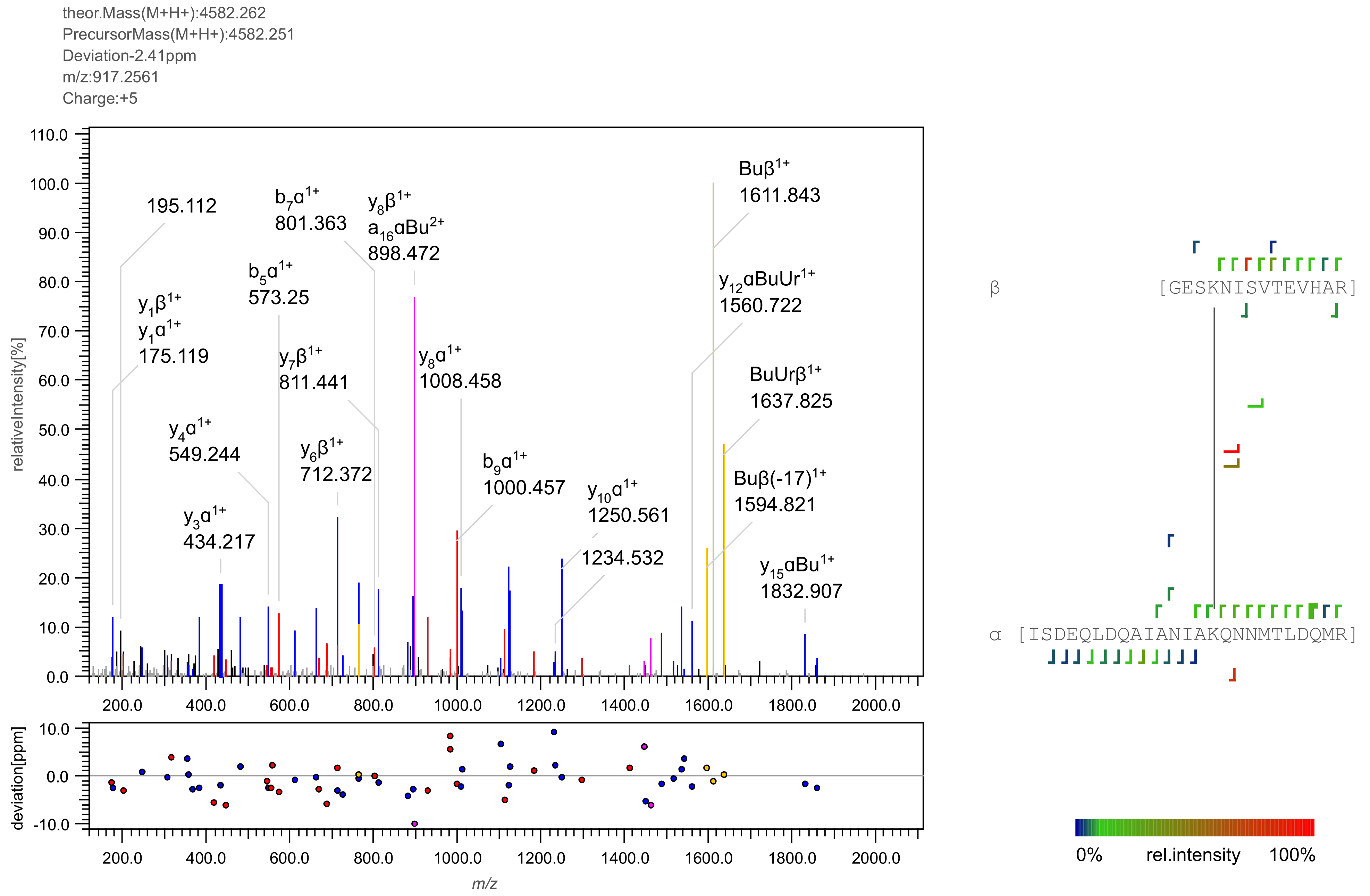

theor.Mass(M+H+):4582.262
PrecursorMass(M+H+):4582.251
Deviation-2.41ppm
m/z:917.2561
Charge:+5
relativeIntensity[%]
deviation[ppm]
m/z
195.112
y1β1+ y1α1+ 175.119
y3α1+ 434.217
y4α1+ 549.244
b5α1+ 573.25
b7α1+ 801.363
y6β1+ 712.372
y7β1+ 811.441
y8β1+ a16αBu2+ 898.472
y8α1+ 1008.458
b9α1+ 1000.457
y10α1+ 1250.561
1234.532
Buβ1+ 1611.843
y12αBuUr1+ 1560.722
BuUrβ1+ 1637.825
Buβ(-17)1+ 1594.821
y15αBu1+ 1832.907
β [GESKNISVTEVHAR]
α [ISDEQLDQAIANIAKQNNMTLDQMR]
1+ 2+ 3+ 4+ 5+
0% rel.intensity 100%

K105-K293

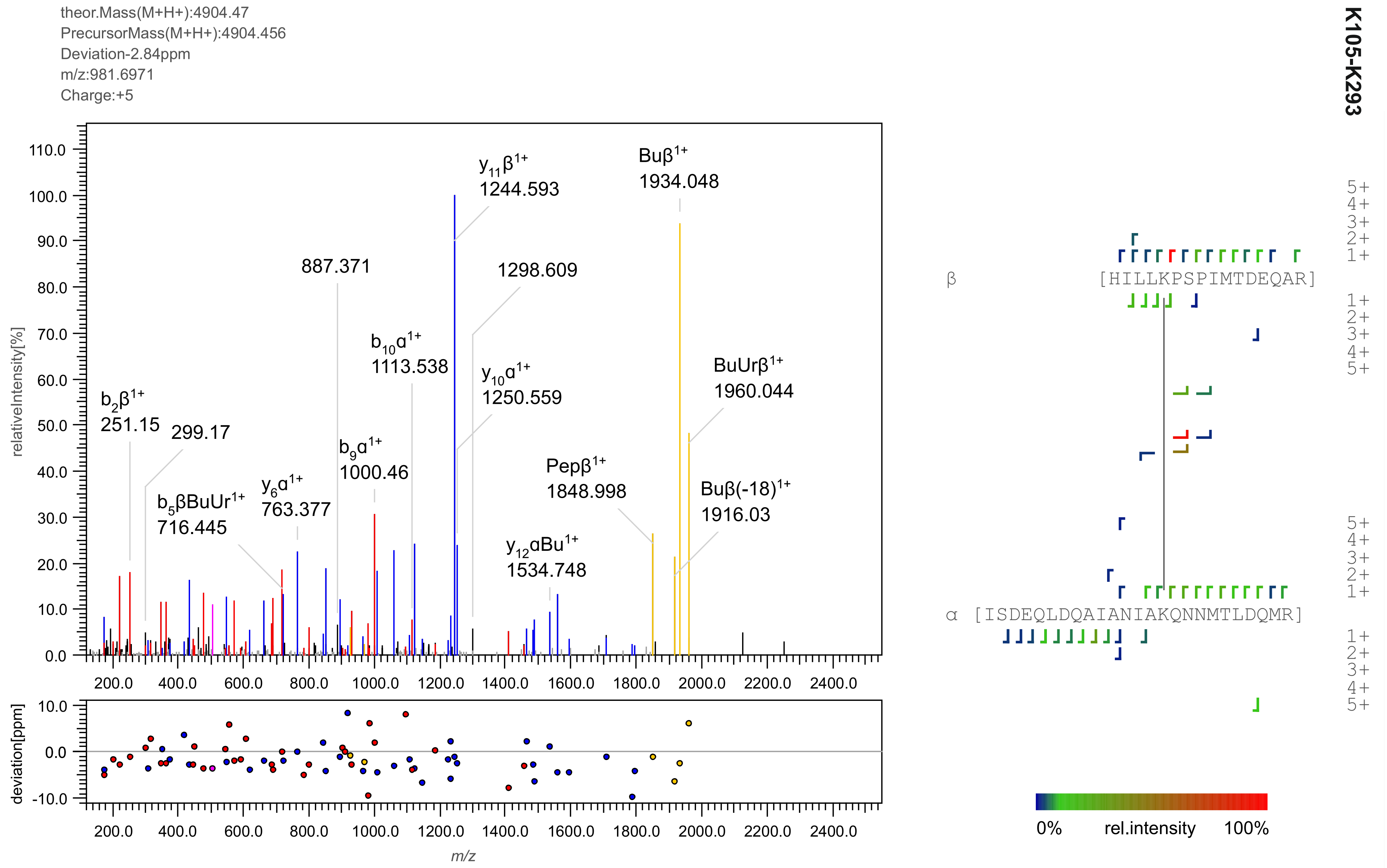

theor.Mass(M+H+):4904.47
PrecursorMass(M+H+):4904.456
Deviation-2.84ppm
m/z:981.6971
Charge:+5
relativeIntensity[%]
$b_2\beta^{1+}$ 251.15
299.17
$b_5\beta BuUr^{1+}$ 716.445
$y_6\alpha^{1+}$ 763.377
887.371
$b_9\alpha^{1+}$ 1000.46
$b_{10}\alpha^{1+}$ 1113.538
$y_{11}\beta^{1+}$ 1244.593
$y_{10}\alpha^{1+}$ 1250.559
1298.609
$y_{12}\alpha Bu^{1+}$ 1534.748
$Pep\beta^{1+}$ 1848.998
$Bu\beta^{1+}$ 1934.048
$BuUr\beta^{1+}$ 1960.044
$Bu\beta(-18)^{1+}$ 1916.03
deviation[ppm]
m/z
β [HILLKPSPIMTDEQAR]
α [ISDEQLDQAIANIAKQNNMTLDQMR]
1+ 2+ 3+ 4+ 5+
0% rel.intensity 100%

K134-K278

theor.Mass(M+H+):2826.478
PrecursorMass(M+H+):2826.473
Deviation-1.85ppm
m/z:566.10034
Charge:+5

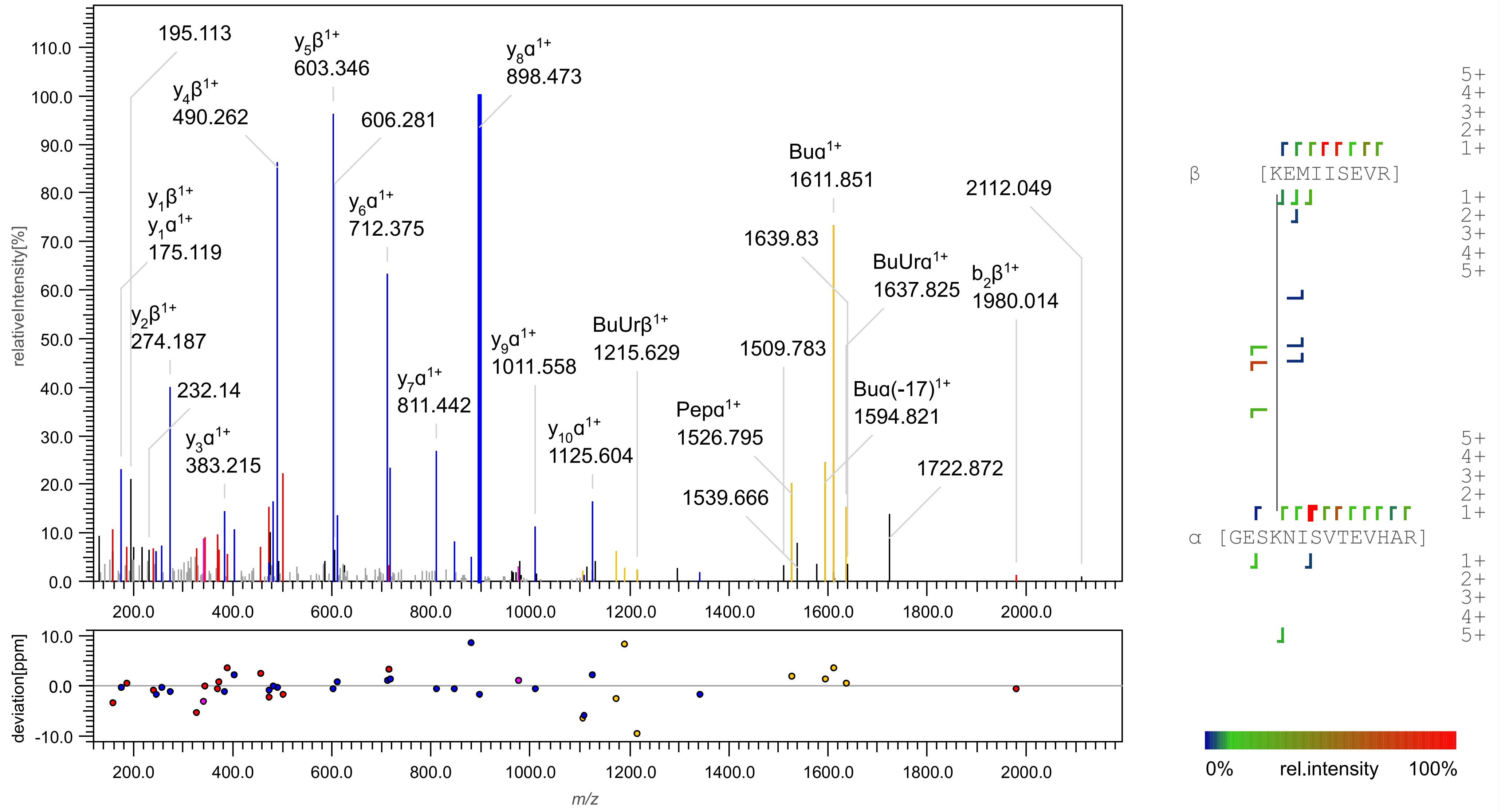

K134-K293

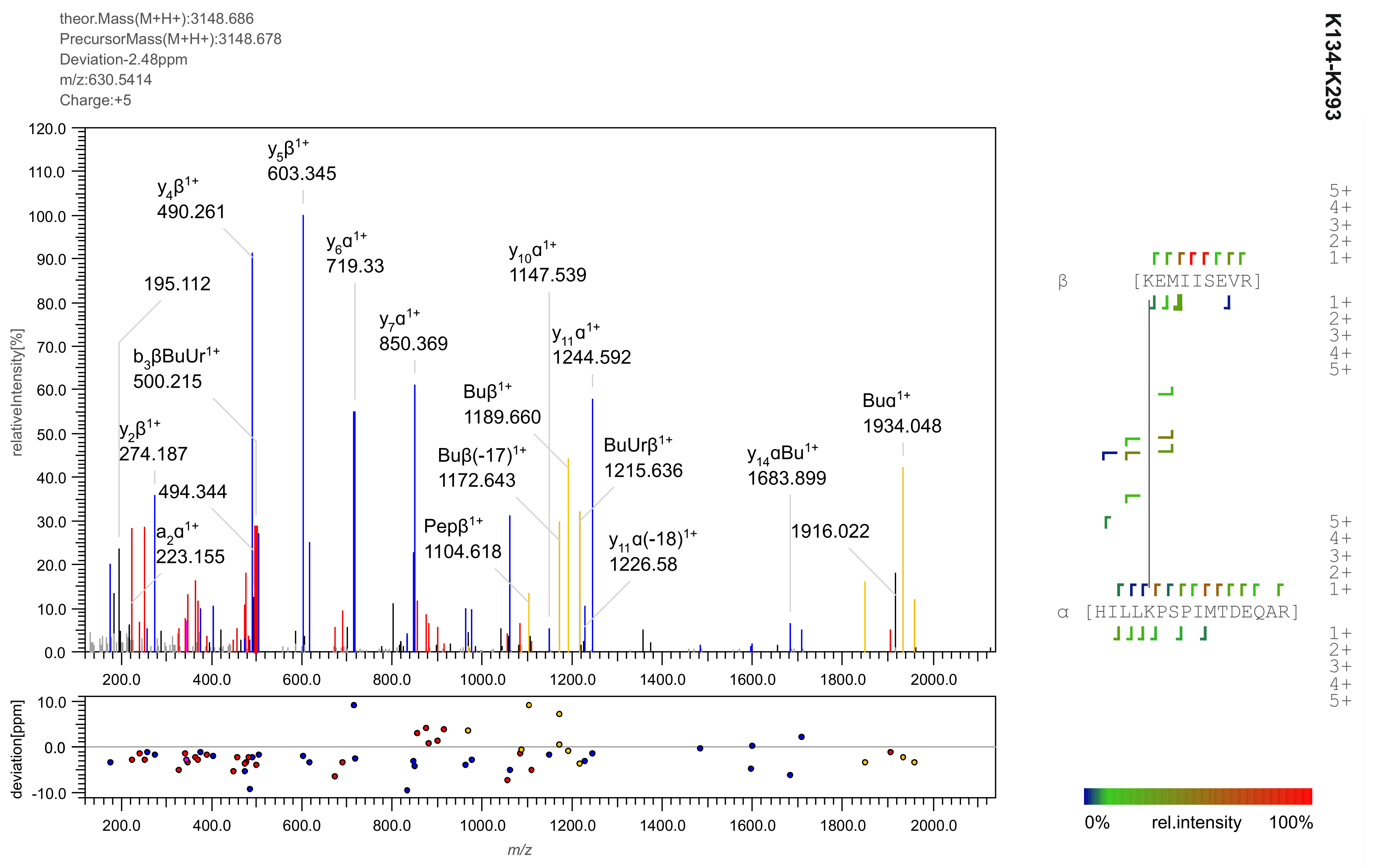

theor.Mass(M+H+):3148.686
PrecursorMass(M+H+):3148.678
Deviation-2.48ppm
m/z:630.5414
Charge:+5
relativeIntensity[%]
deviation[ppm]
m/z
y5β1+ 603.345
y4β1+ 490.261
y6α1+ 719.33
y10α1+ 1147.539
y7α1+ 850.369
y11α1+ 1244.592
195.112
b3βBuUr1+ 500.215
Buβ1+ 1189.660
Buβ(-17)1+ 1172.643
BuUrβ1+ 1215.636
y14αBu1+ 1683.899
Buα1+ 1934.048
y2β1+ 274.187
494.344
a2α1+ 223.155
Pepβ1+ 1104.618
y11α(-18)1+ 1226.58
1916.022
β [KEMIISEVR]
α [HILLKPSPIMTDEQAR]
0% rel.intensity 100%

K251-K278

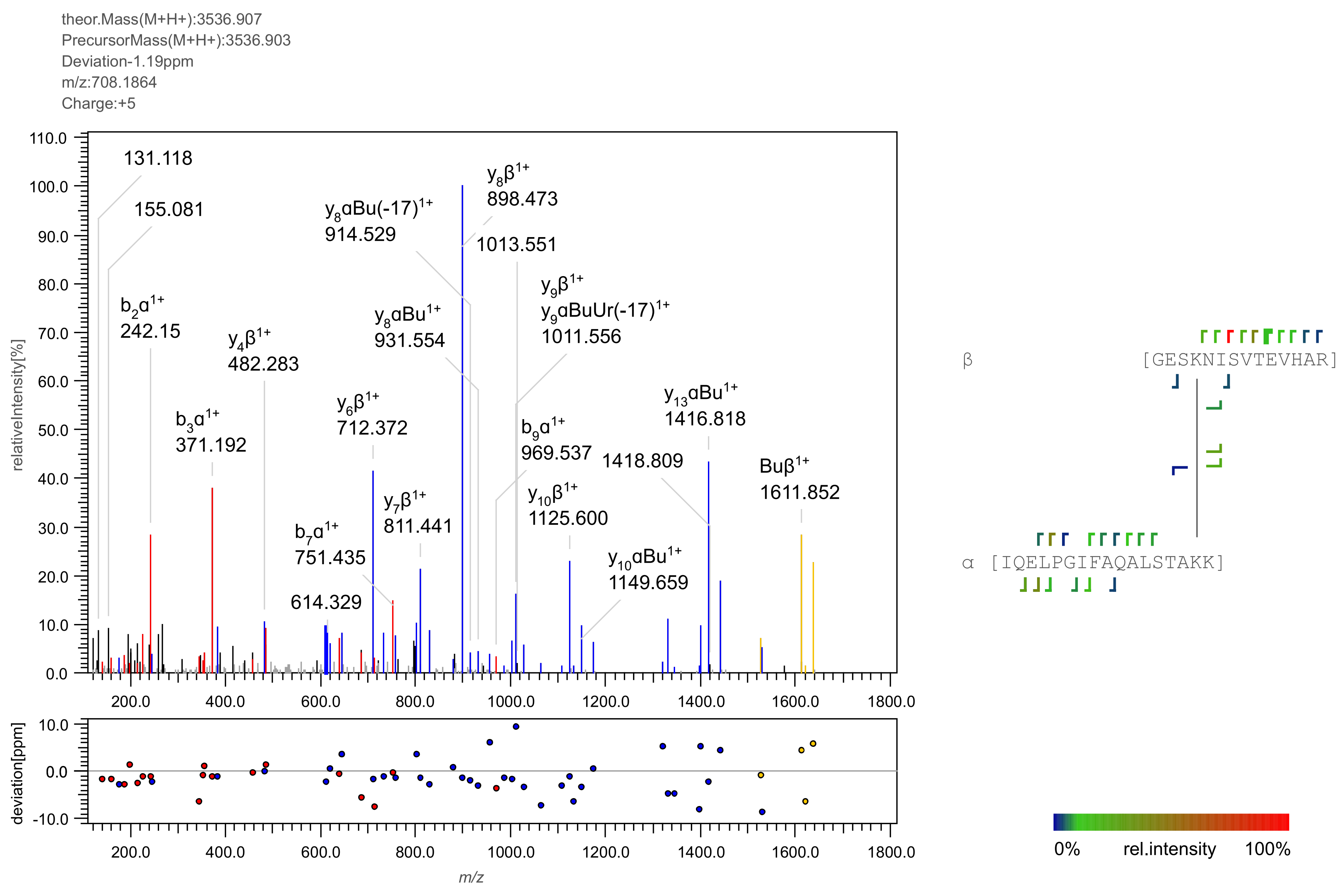

theor.Mass(M+H+):3536.907
PrecursorMass(M+H+):3536.903
Deviation-1.19ppm
m/z:708.1864
Charge:+5
relativeIntensity[%]
131.118
155.081
$b_2α^{1+}$ 242.15
$b_3α^{1+}$ 371.192
$y_4β^{1+}$ 482.283
614.329
$b_7α^{1+}$ 751.435
$y_6β^{1+}$ 712.372
$y_7β^{1+}$ 811.441
$y_8αBu(-17)^{1+}$ 914.529
$y_8αBu^{1+}$ 931.554
$y_8β^{1+}$ 898.473
1013.551
$y_9β^{1+}$
$y_9αBuUr(-17)^{1+}$ 1011.556
$b_9α^{1+}$ 969.537
$y_{10}β^{1+}$ 1125.600
$y_{10}αBu^{1+}$ 1149.659
1418.809
$y_{13}αBu^{1+}$ 1416.818
$Buβ^{1+}$ 1611.852
deviation[ppm]
m/z
β [GESKNISVTEVHAR] 1+ 1+
α [IQELPGIFAQALSTAKK] 1+ 1+
0% rel.intensity 100%

K251-K293

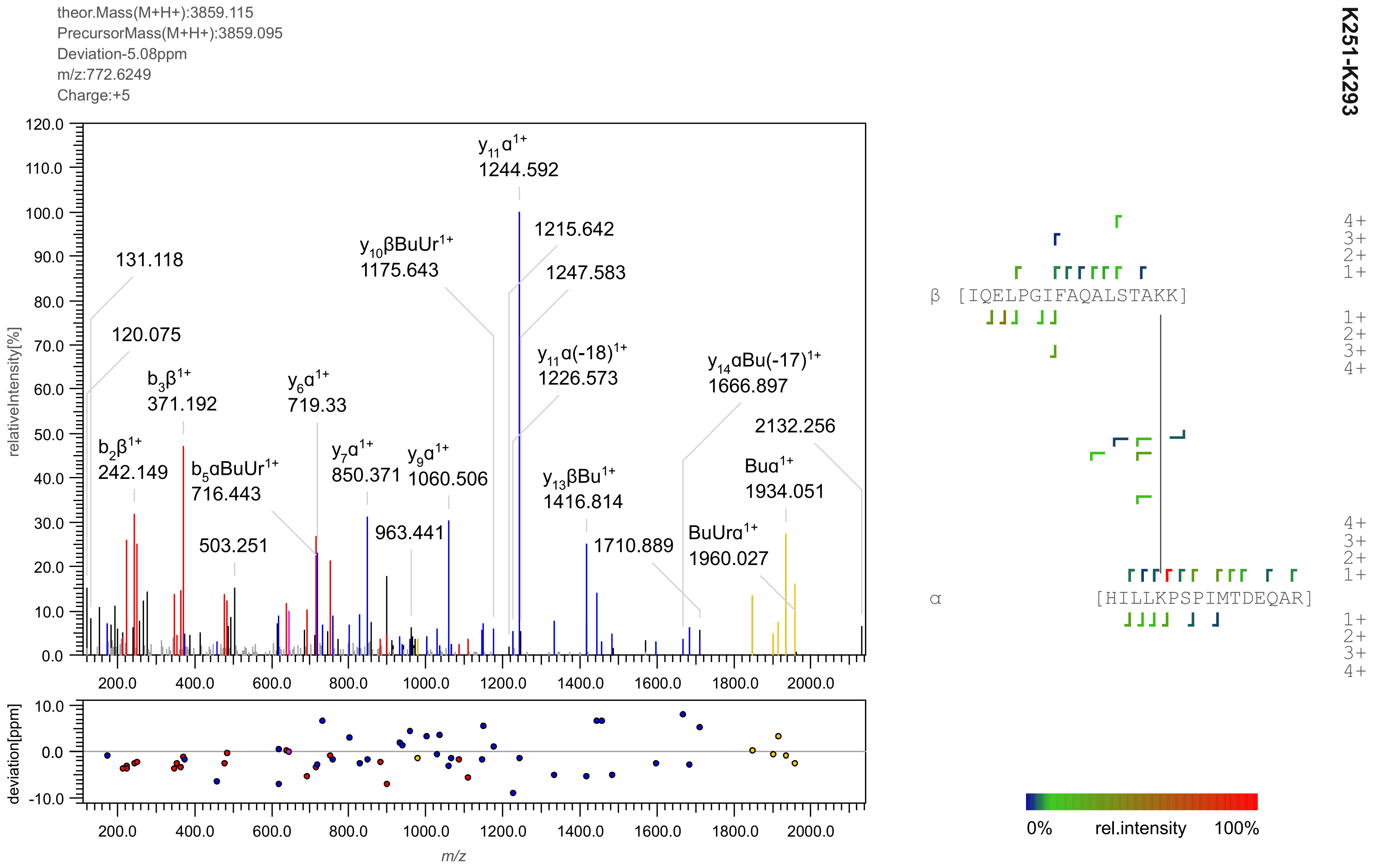

theor.Mass(M+H+):3859.115
PrecursorMass(M+H+):3859.095
Deviation-5.08ppm
m/z:772.6249
Charge:+5
relativeIntensity[%]
deviation[ppm]
m/z
$y_{11}α^{1+}$ 1244.592
1215.642
1247.583
$y_{10}βBuUr^{1+}$ 1175.643
131.118
120.075
$b_3β^{1+}$ 371.192
$y_6α^{1+}$ 719.33
$y_{11}α(-18)^{1+}$ 1226.573
$y_{14}αBu(-17)^{1+}$ 1666.897
2132.256
$b_2β^{1+}$ 242.149
$b_5αBuUr^{1+}$ 716.443
$y_7α^{1+}$ 850.371
$y_9α^{1+}$ 1060.506
$y_{13}βBu^{1+}$ 1416.814
$Buα^{1+}$ 1934.051
963.441
503.251
1710.889
$BuUrα^{1+}$ 1960.027
β [IQELPGIFAQALSTAKK]
α [HILLKPSPIMTDEQAR]
0% rel.intensity 100%

K251-K405

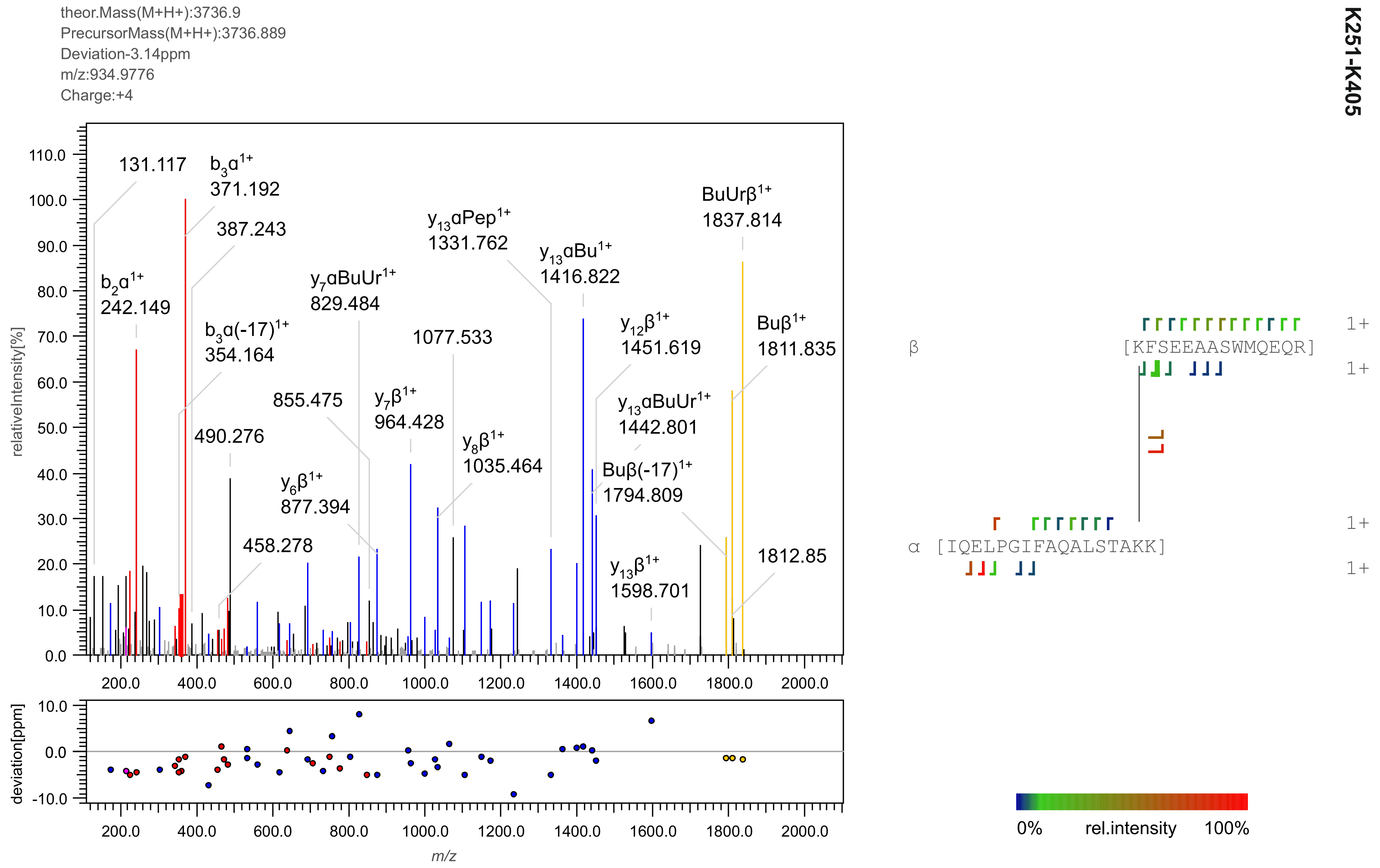

theor.Mass(M+H+):3736.9
PrecursorMass(M+H+):3736.889
Deviation-3.14ppm
m/z:934.9776
Charge:+4
relativeIntensity[%]
131.117
b3α1+ 371.192
387.243
b2α1+ 242.149
b3α(-17)1+ 354.164
490.276
458.278
855.475
y7αBuUr1+ 829.484
y6β1+ 877.394
y7β1+ 964.428
1077.533
y8β1+ 1035.464
y13αPep1+ 1331.762
y13αBu1+ 1416.822
y12β1+ 1451.619
y13αBuUr1+ 1442.801
Buβ(-17)1+ 1794.809
y13β1+ 1598.701
BuUrβ1+ 1837.814
Buβ1+ 1811.835
1812.85
deviation[ppm]
m/z
β [KFSEEAASWMQEQR] 1+ 1+
α [IQELPGIFAQALSTAKK] 1+ 1+
0% rel.intensity 100%

K252-K278

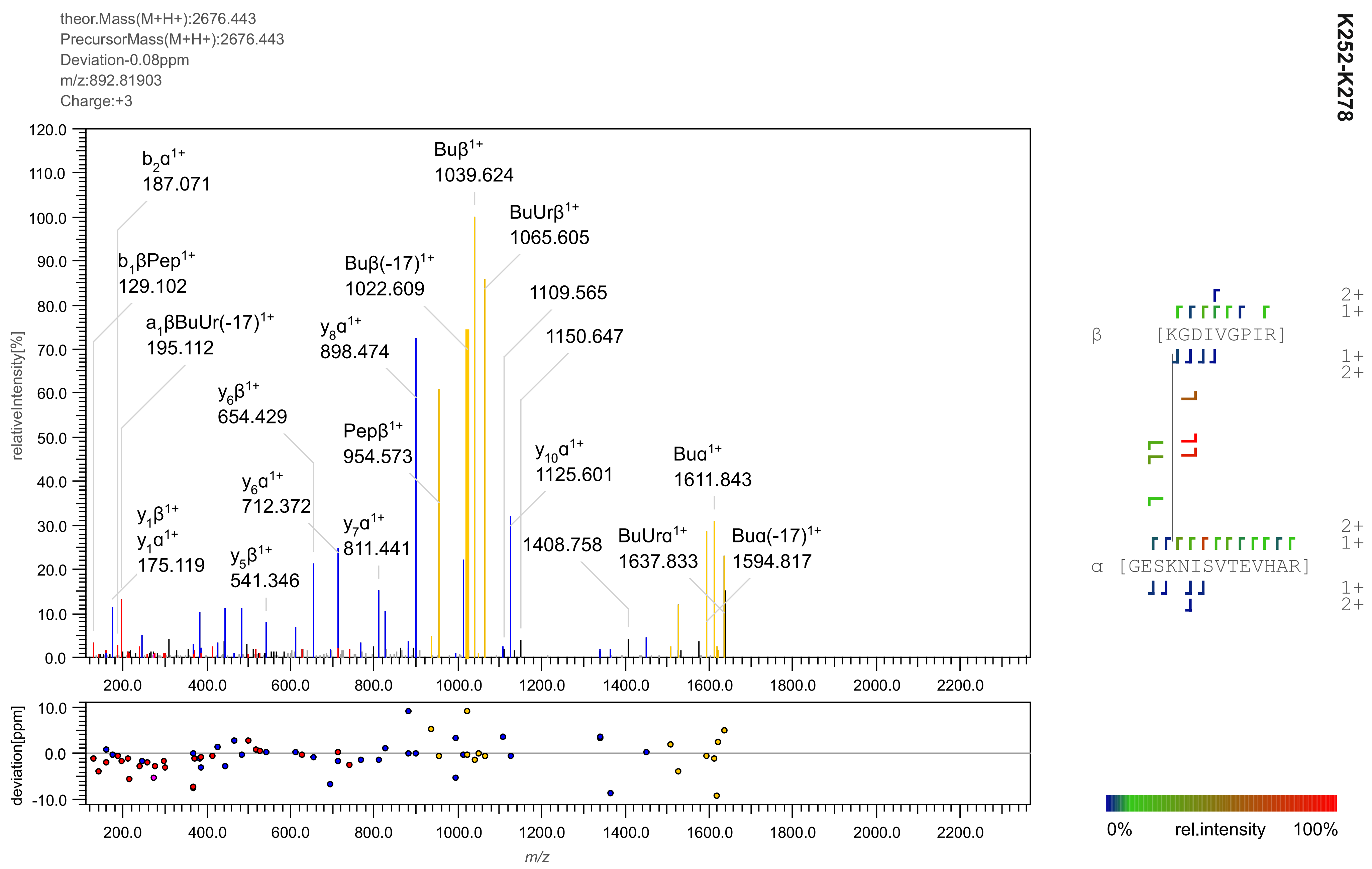

theor.Mass(M+H+):2676.443
PrecursorMass(M+H+):2676.443
Deviation-0.08ppm
m/z:892.81903
Charge:+3
relativeIntensity[%]
deviation[ppm]
m/z
b2α1+ 187.071
b1βPep1+ 129.102
a1βBuUr(-17)1+ 195.112
y1β1+ y1α1+ 175.119
y6β1+ 654.429
y6α1+ 712.372
y5β1+ 541.346
y8α1+ 898.474
y7α1+ 811.441
Pepβ1+ 954.573
Buβ(-17)1+ 1022.609
Buβ1+ 1039.624
BuUrβ1+ 1065.605
1109.565
1150.647
y10α1+ 1125.601
1408.758
BuUrα1+ 1637.833
Buα1+ 1611.843
Buα(-17)1+ 1594.817
β [KGDIVGPIR]
α [GESKNISVTEVHAR]
2+
1+
0%
rel.intensity
100%

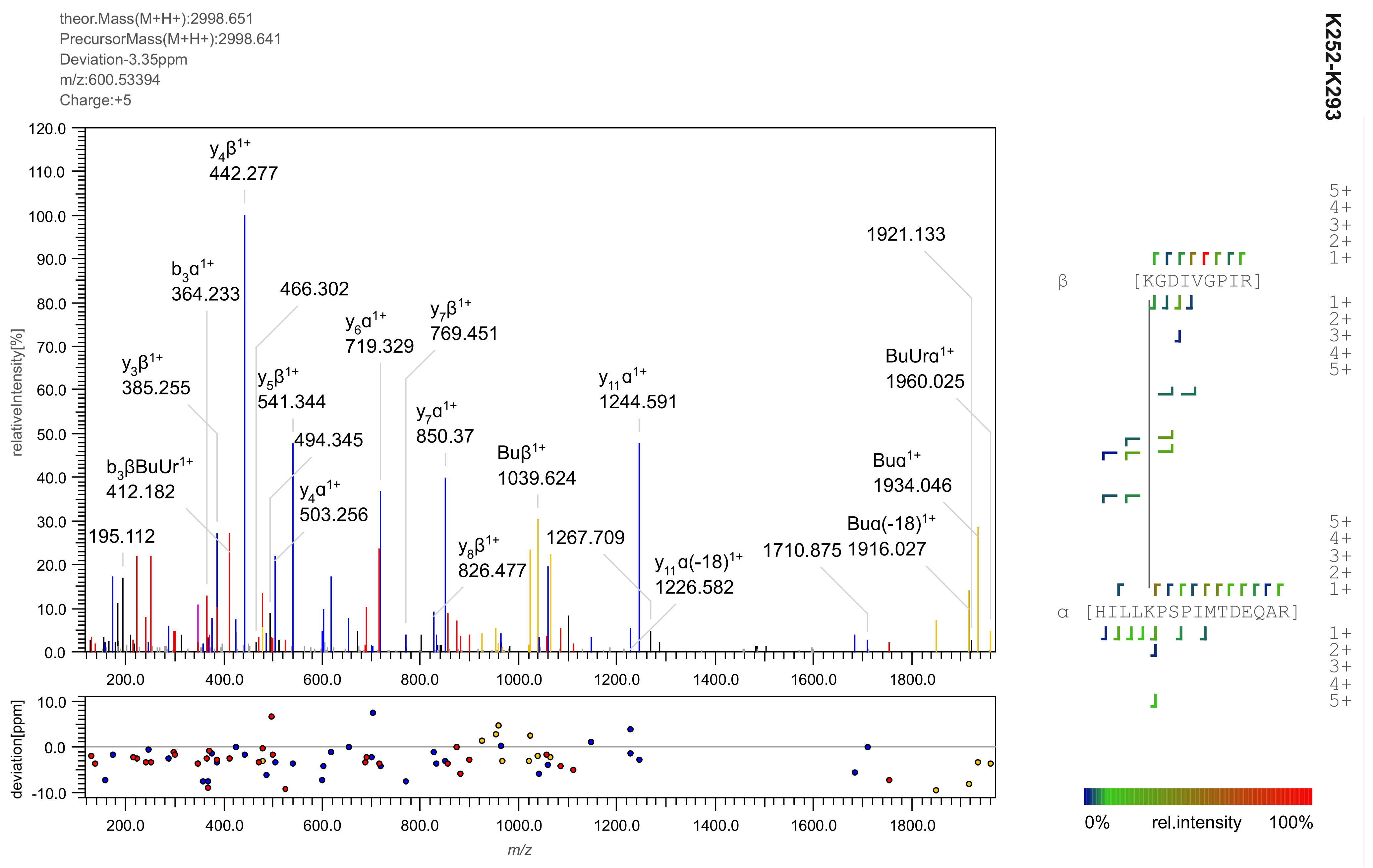
K252-K293
theor.Mass(M+H+):2998.651
PrecursorMass(M+H+):2998.641
Deviation-3.35ppm
m/z:600.53394
Charge:+5
relativeIntensity[%]
y4β1+ 442.277
b3α1+ 364.233
466.302
y6α1+ 719.329
y7β1+ 769.451
y3β1+ 385.255
y5β1+ 541.344
494.345
y7α1+ 850.37
y11α1+ 1244.591
b3βBuUr1+ 412.182
y4α1+ 503.256
Buβ1+ 1039.624
195.112
y8β1+ 826.477
1267.709
y11α(-18)1+ 1226.582
1921.133
BuUrα1+ 1960.025
Buα1+ 1934.046
Buα(-18)1+
1710.875 1916.027
deviation[ppm]
m/z
β [KGDIVGPIR]
α [HILLKPSPIMTDEQAR]
0% rel.intensity 100%

K252-K362

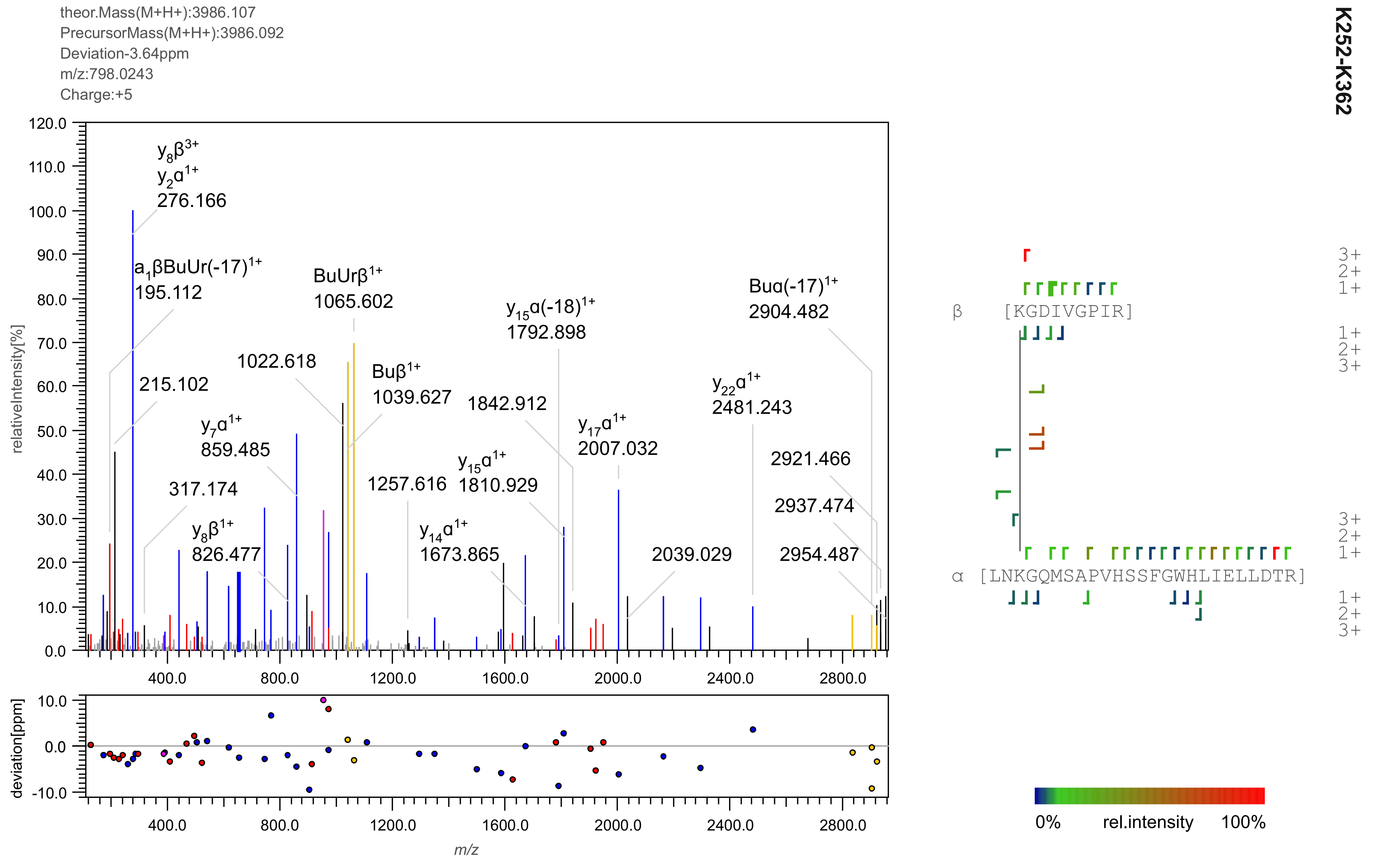
theor.Mass(M+H+):3986.107
PrecursorMass(M+H+):3986.092
Deviation-3.64ppm
m/z:798.0243
Charge:+5
relativeIntensity[%]
deviation[ppm]
m/z
y8β3+
y2α1+
276.166
a1βBuUr(-17)1+
195.112
215.102
317.174
y7α1+
859.485
y8β1+
826.477
1022.618
BuUrβ1+
1065.602
Buβ1+
1039.627
1257.616
1842.912
y15α(-18)1+
1792.898
y15α1+
1810.929
y14α1+
1673.865
y17α1+
2007.032
2039.029
y22α1+
2481.243
Buα(-17)1+
2904.482
2921.466
2937.474
2954.487
β [KGDIVGPIR]
α [LNKGQMSAPVHSSFGWHLIELLDTR]
1+
2+
3+
0%
rel.intensity
100%

K252-K394

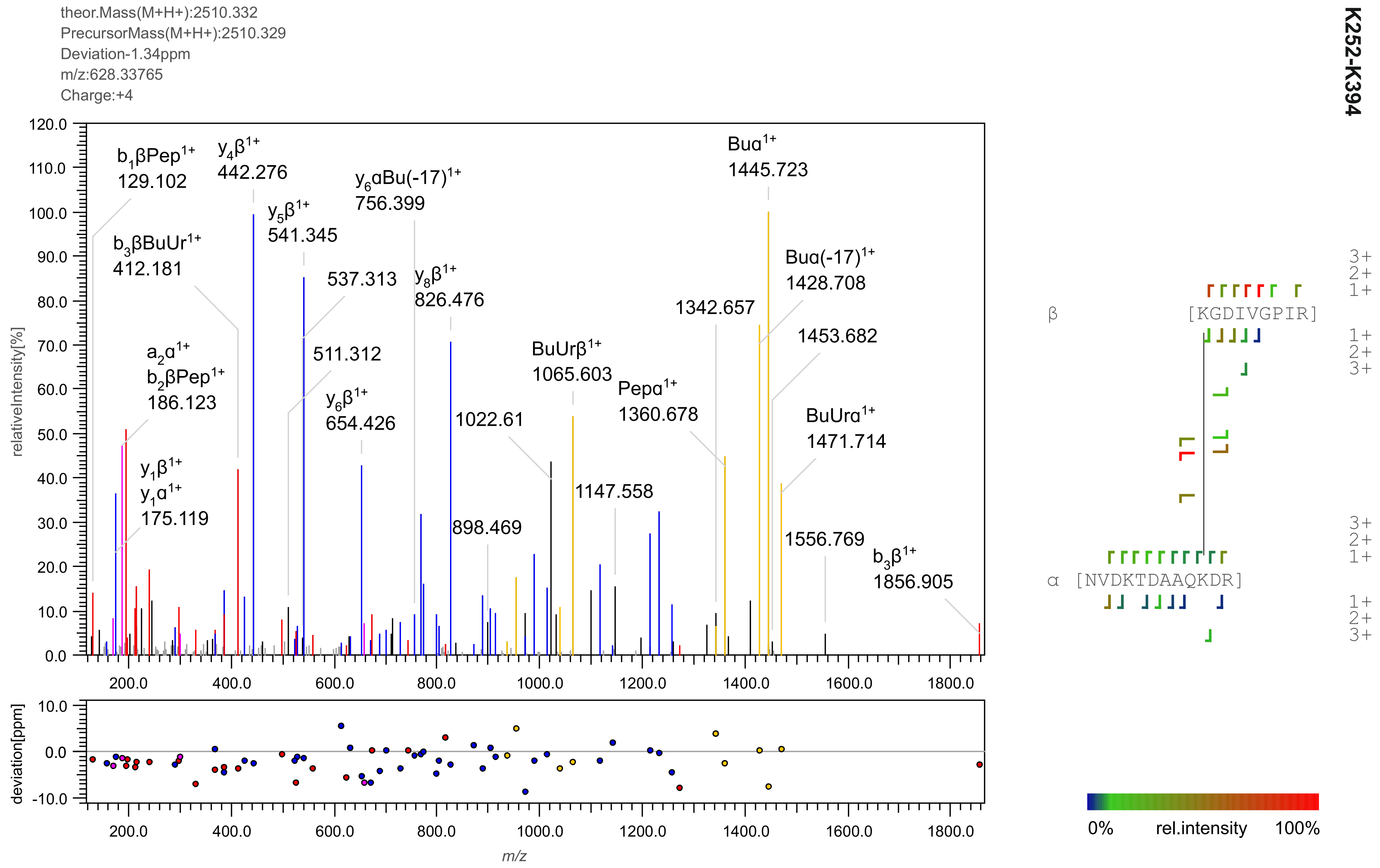
theor.Mass(M+H+):2510.332
PrecursorMass(M+H+):2510.329
Deviation-1.34ppm
m/z:628.33765
Charge:+4
relativeIntensity[%]
deviation[ppm]
m/z
b1βPep1+ 129.102
y4β1+ 442.276
b3βBuUr1+ 412.181
y5β1+ 541.345
y6αBu(-17)1+ 756.399
537.313
y8β1+ 826.476
a2α1+
b2βPep1+ 186.123
511.312
y6β1+ 654.426
y1β1+
y1α1+ 175.119
1022.61
898.469
BuUrβ1+ 1065.603
Pepα1+ 1360.678
1147.558
1342.657
Buα1+ 1445.723
Buα(-17)1+ 1428.708
1453.682
BuUrα1+ 1471.714
1556.769
b3β1+ 1856.905
β [KGDIVGPIR]
α [NVDKTDAAQKDR]
3+ 2+ 1+
1+ 2+ 3+
0% rel.intensity 100%

K269-K278

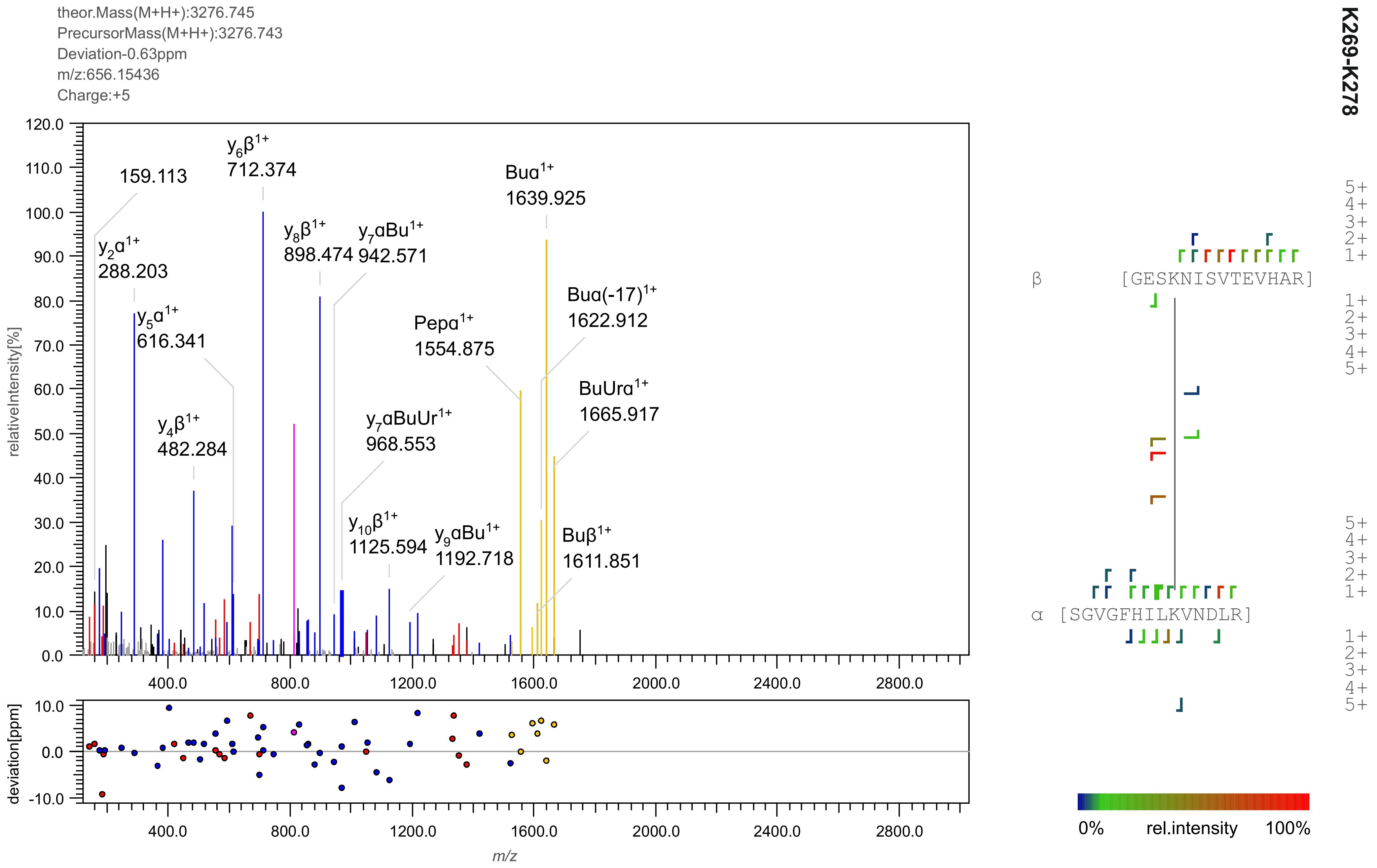
theor.Mass(M+H+):3276.745
PrecursorMass(M+H+):3276.743
Deviation-0.63ppm
m/z:656.15436
Charge:+5
relativeIntensity[%]
deviation[ppm]
m/z
159.113
y2α1+ 288.203
y5α1+ 616.341
y4β1+ 482.284
y6β1+ 712.374
y8β1+ 898.474
y7αBu1+ 942.571
y7αBuUr1+ 968.553
y10β1+ 1125.594
y9αBu1+ 1192.718
Pepα1+ 1554.875
Buα1+ 1639.925
Buα(-17)1+ 1622.912
BuUrα1+ 1665.917
Buβ1+ 1611.851
β [GESKNISVTEVHAR]
α [SGVGFHILKVNDLR]
0% rel.intensity 100%

K269-K293

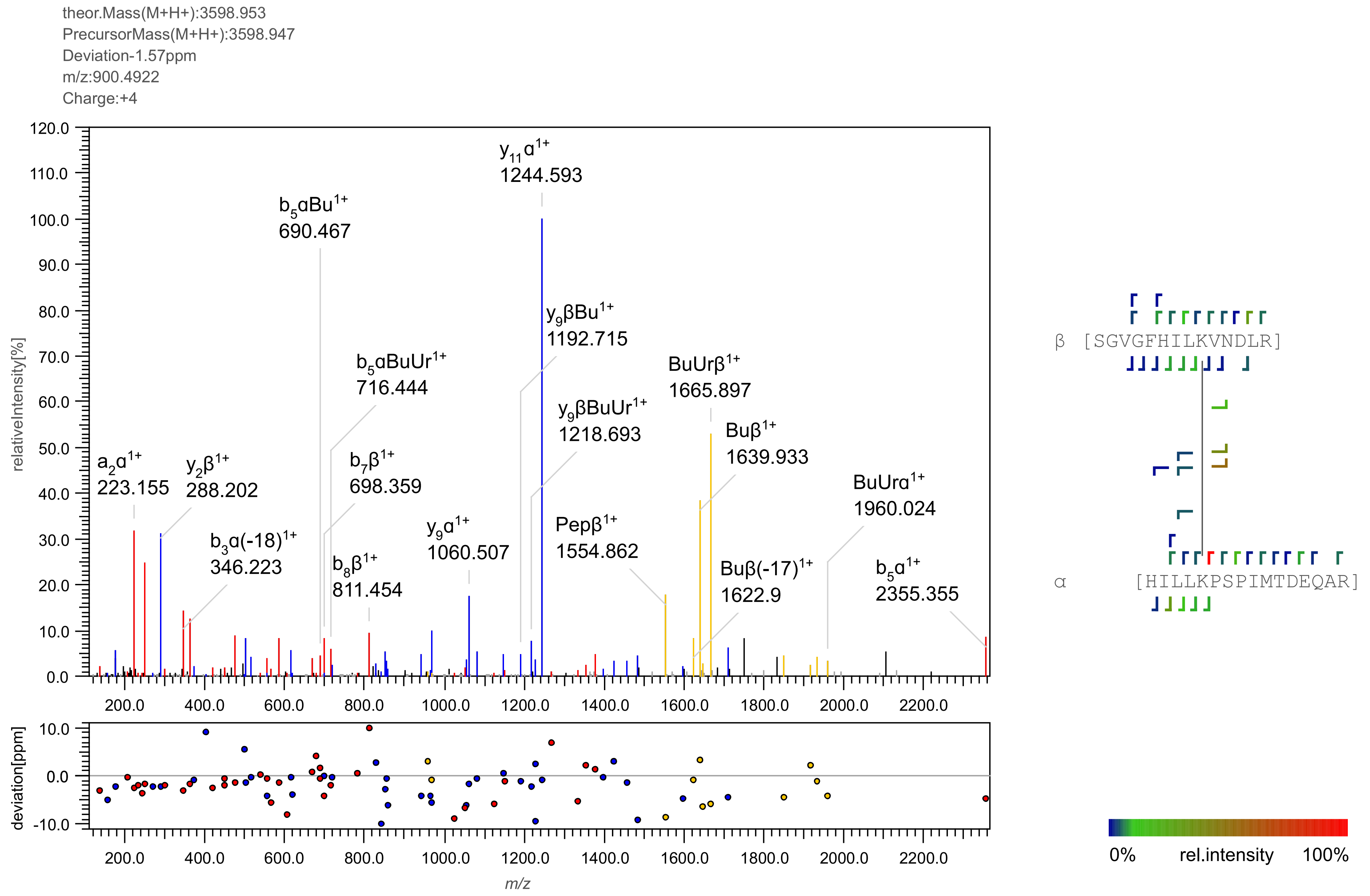

theor.Mass(M+H+):3598.953
PrecursorMass(M+H+):3598.947
Deviation-1.57ppm
m/z:900.4922
Charge:+4
relativeIntensity[%]
deviation[ppm]
m/z
a2α1+ 223.155
y2β1+ 288.202
b3α(-18)1+ 346.223
b5αBu1+ 690.467
b5αBuUr1+ 716.444
b7β1+ 698.359
b8β1+ 811.454
y9α1+ 1060.507
y11α1+ 1244.593
y9βBu1+ 1192.715
y9βBuUr1+ 1218.693
Pepβ1+ 1554.862
BuUrβ1+ 1665.897
Buβ1+ 1639.933
Buβ(-17)1+ 1622.9
BuUrα1+ 1960.024
b5α1+ 2355.355
β [SGVGFHILKVNDLR]
α [HILLKPSPIMTDEQAR]
2+
1+
0%
rel.intensity
100%

K269-K394

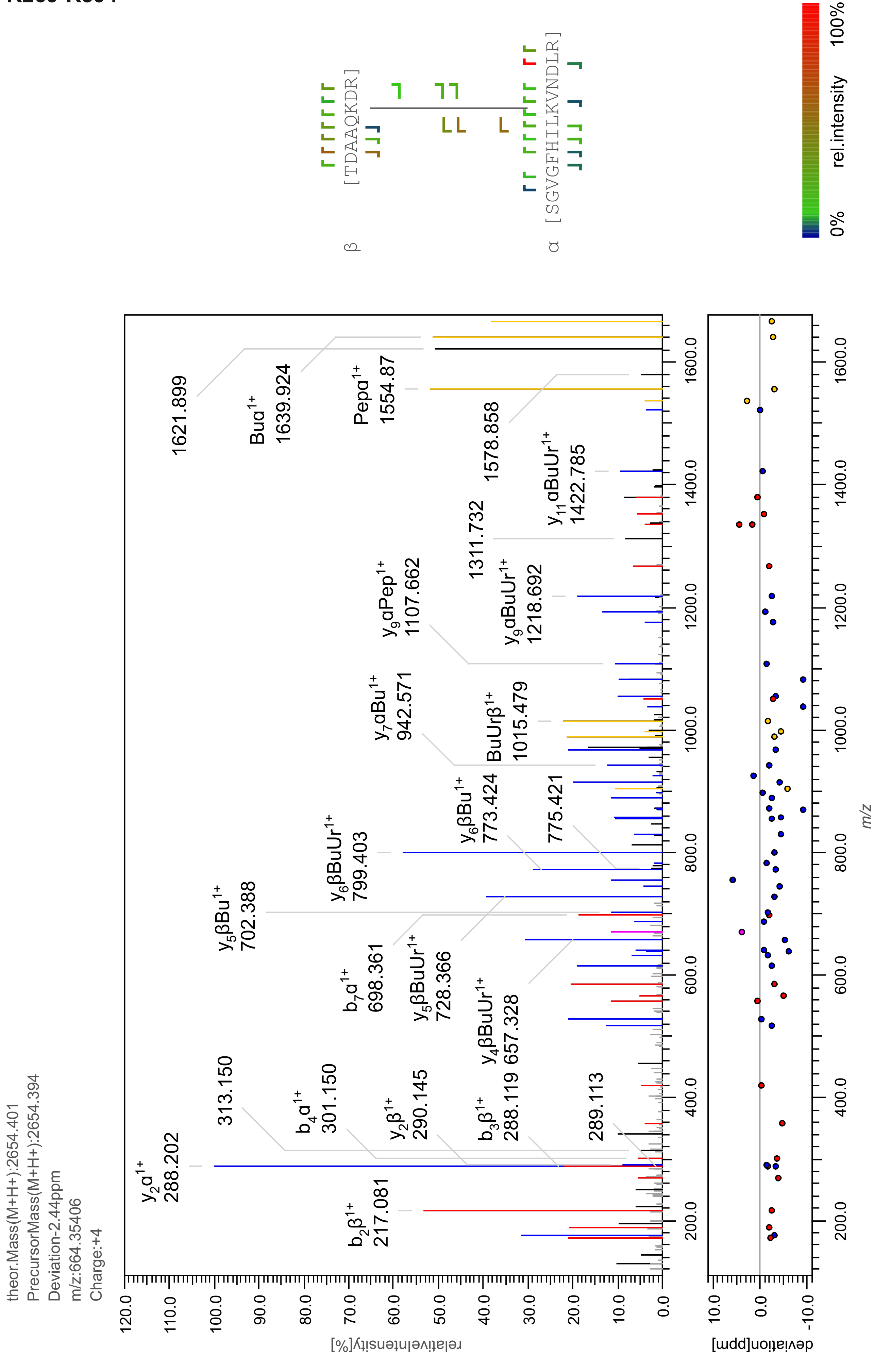

theor.Mass(M+H+):2654.401
PrecursorMass(M+H+):2654.394
Deviation-2.44ppm
m/z:664.35406
Charge:+4
β [TDAAQKDR] 1+
α [SGVGFHILKVNDLR] 1+
0%
rel.intensity
100%
relativeIntensity[%]
deviation[ppm]
m/z
b2β1+ 217.081
y2α1+ 288.202
313.150
b4α1+ 301.150
y2β1+ 290.145
b3β1+ 288.119
289.113
y4βBuUr1+ 657.328
y5βBuUr1+ 728.366
b7α1+ 698.361
y5βBu1+ 702.388
y6βBuUr1+ 799.403
y6βBu1+ 773.424
775.421
y7αBu1+ 942.571
BuUrβ1+ 1015.479
y9αPep1+ 1107.662
y9αBuUr1+ 1218.692
1311.732
y11αBuUr1+ 1422.785
1578.858
Pepα1+ 1554.87
Buα1+ 1639.924
1621.899

K278-K394

theor.Mass(M+H+):2626.318
PrecursorMass(M+H+):2626.312
Deviation-2.44ppm
m/z:876.1087
Charge:+3

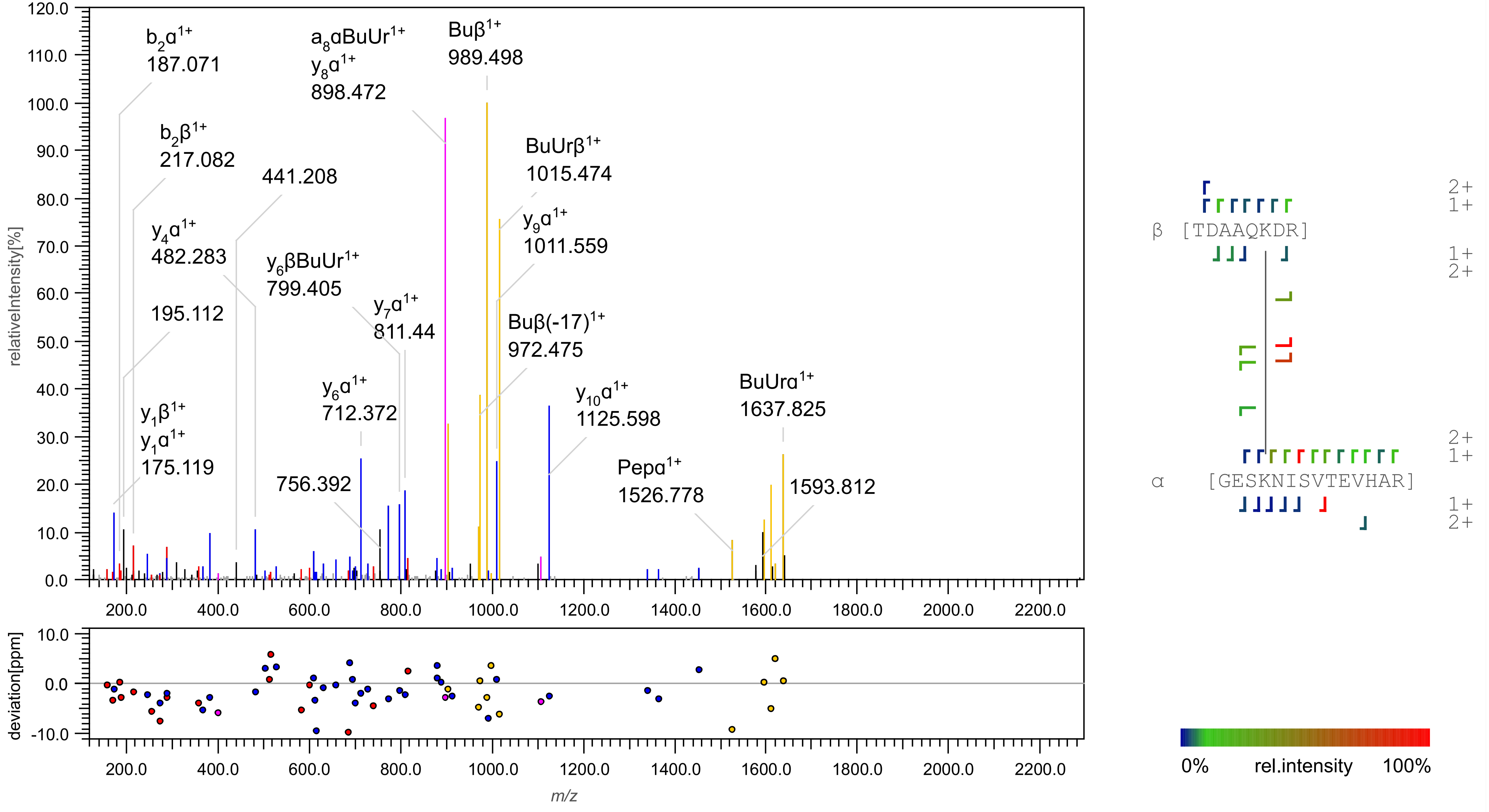

K278-K405

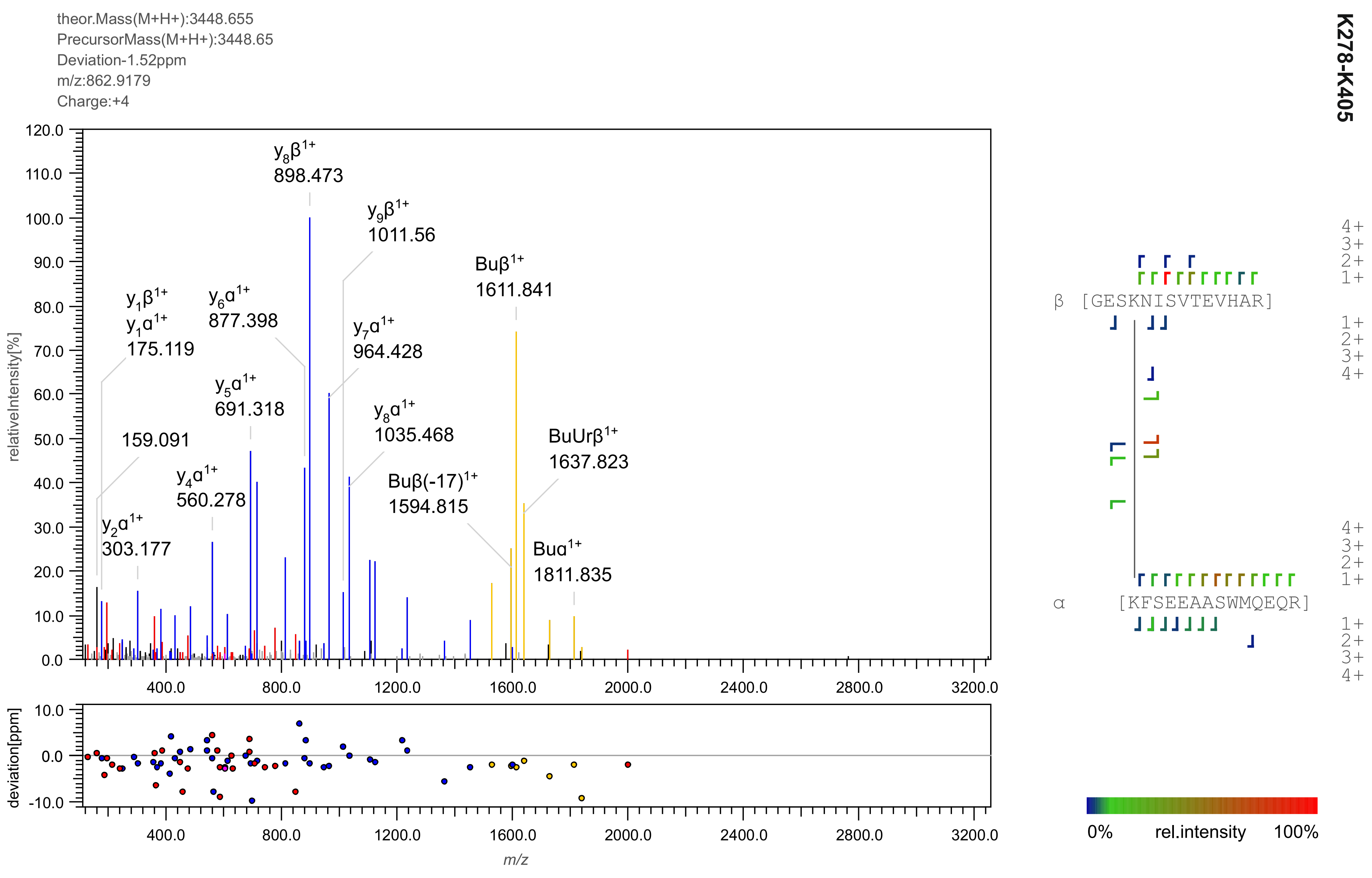

theor.Mass(M+H+):3448.655
PrecursorMass(M+H+):3448.65
Deviation-1.52ppm
m/z:862.9179
Charge:+4
relativeIntensity[%]
deviation[ppm]
m/z
y8β1+ 898.473
y9β1+ 1011.56
Buβ1+ 1611.841
y1β1+ y1α1+ 175.119
y6α1+ 877.398
y7α1+ 964.428
y5α1+ 691.318
y8α1+ 1035.468
159.091
y4α1+ 560.278
BuUrβ1+ 1637.823
Buβ(-17)1+ 1594.815
y2α1+ 303.177
Buα1+ 1811.835
β [GESKNISVTEVHAR]
α [KFSEEAASWMQEQR]
4+ 3+ 2+ 1+
0% rel.intensity 100%

K293-K394

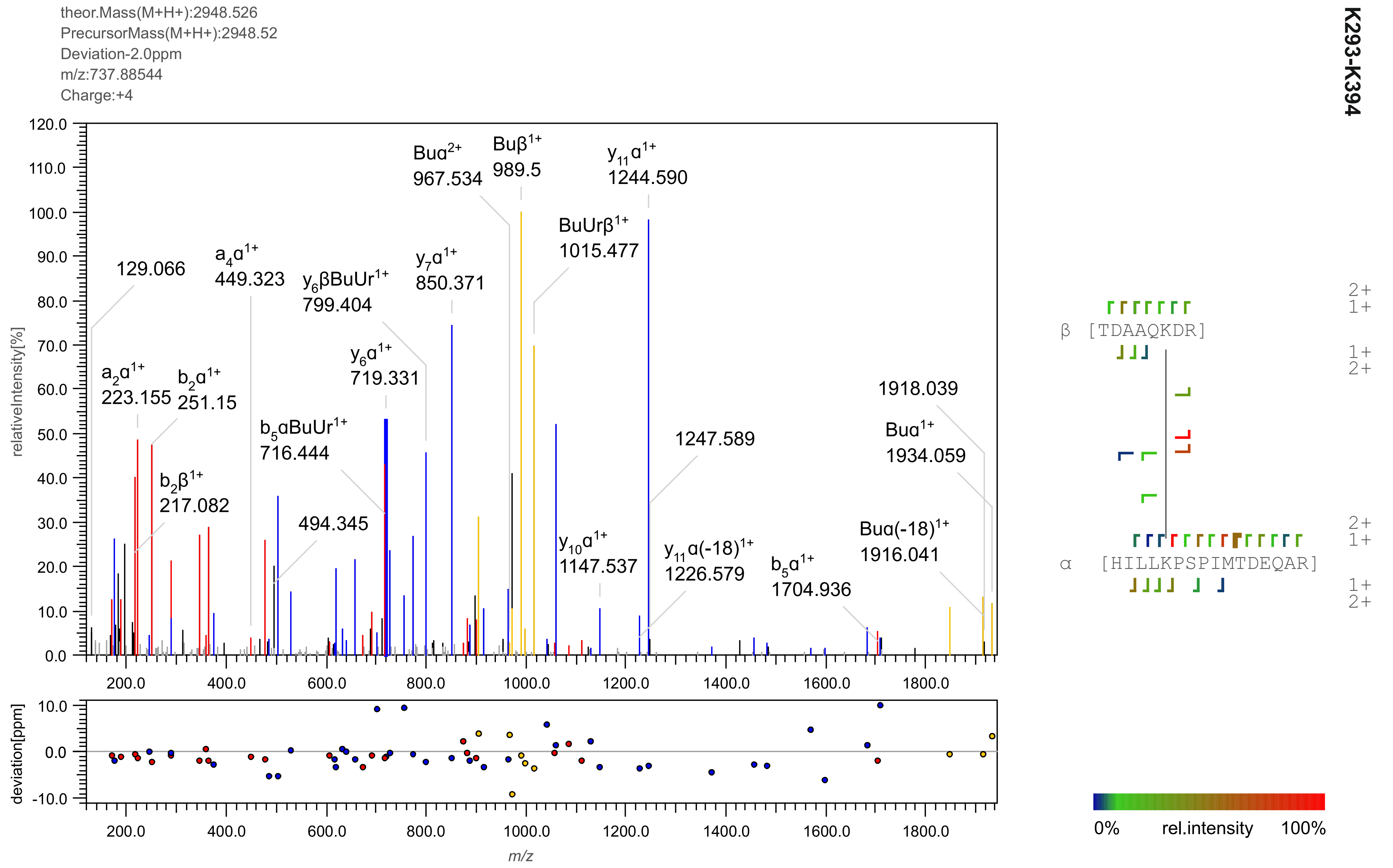
theor.Mass(M+H+):2948.526
PrecursorMass(M+H+):2948.52
Deviation-2.0ppm
m/z:737.88544
Charge:+4
relativeIntensity[%]
deviation[ppm]
m/z
129.066
$a_2α^{1+}$ 223.155
$b_2α^{1+}$ 251.15
$b_2β^{1+}$ 217.082
$a_4α^{1+}$ 449.323
494.345
$b_5αBuUr^{1+}$ 716.444
$y_6βBuUr^{1+}$ 799.404
$y_6α^{1+}$ 719.331
$y_7α^{1+}$ 850.371
$Buα^{2+}$ 967.534
$Buβ^{1+}$ 989.5
$BuUrβ^{1+}$ 1015.477
$y_{10}α^{1+}$ 1147.537
$y_{11}α^{1+}$ 1244.590
1247.589
$y_{11}α(-18)^{1+}$ 1226.579
$b_5α^{1+}$ 1704.936
$Buα(-18)^{1+}$ 1916.041
1918.039
$Buα^{1+}$ 1934.059
β [TDAAQKDR]
α [HILLKPSPIMTDEQAR]
2+
1+
0%
rel.intensity
100%

K293-K405

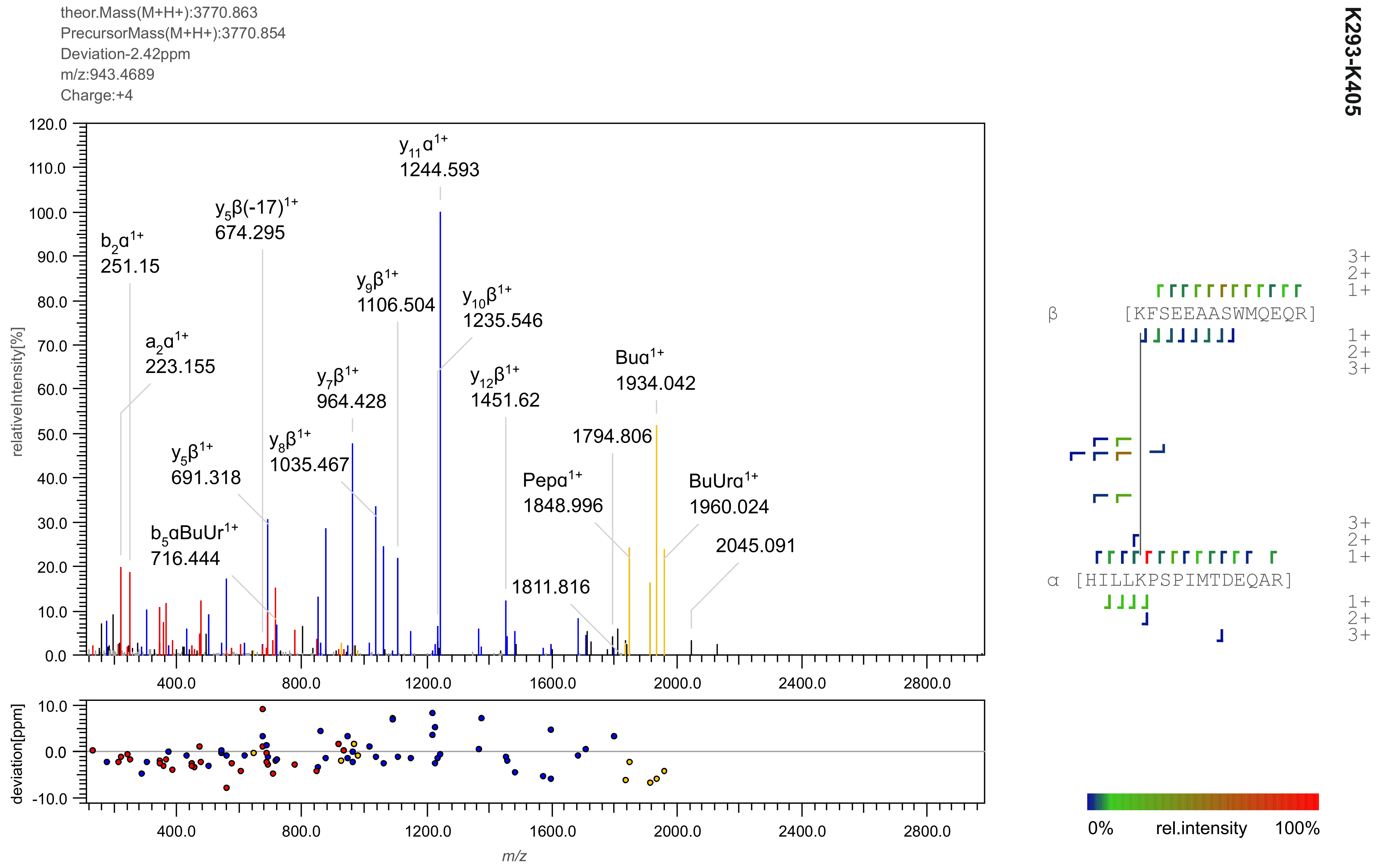

theor.Mass(M+H+):3770.863
PrecursorMass(M+H+):3770.854
Deviation-2.42ppm
m/z:943.4689
Charge:+4
relativeIntensity[%]
$b_2α^{1+}$ 251.15
$a_2α^{1+}$ 223.155
$y_5β(-17)^{1+}$ 674.295
$y_5β^{1+}$ 691.318
$b_5αBuUr^{1+}$ 716.444
$y_9β^{1+}$ 1106.504
$y_7β^{1+}$ 964.428
$y_8β^{1+}$ 1035.467
$y_{11}α^{1+}$ 1244.593
$y_{10}β^{1+}$ 1235.546
$y_{12}β^{1+}$ 1451.62
$Pepα^{1+}$ 1848.996
1794.806
1811.816
$Buα^{1+}$ 1934.042
$BuUrα^{1+}$ 1960.024
2045.091
deviation[ppm]
m/z
β [KFSEEAASWMQEQR]
α [HILLKPSPIMTDEQAR]
3+ 2+ 1+
1+ 2+ 3+
0% rel.intensity 100%

K362-K405

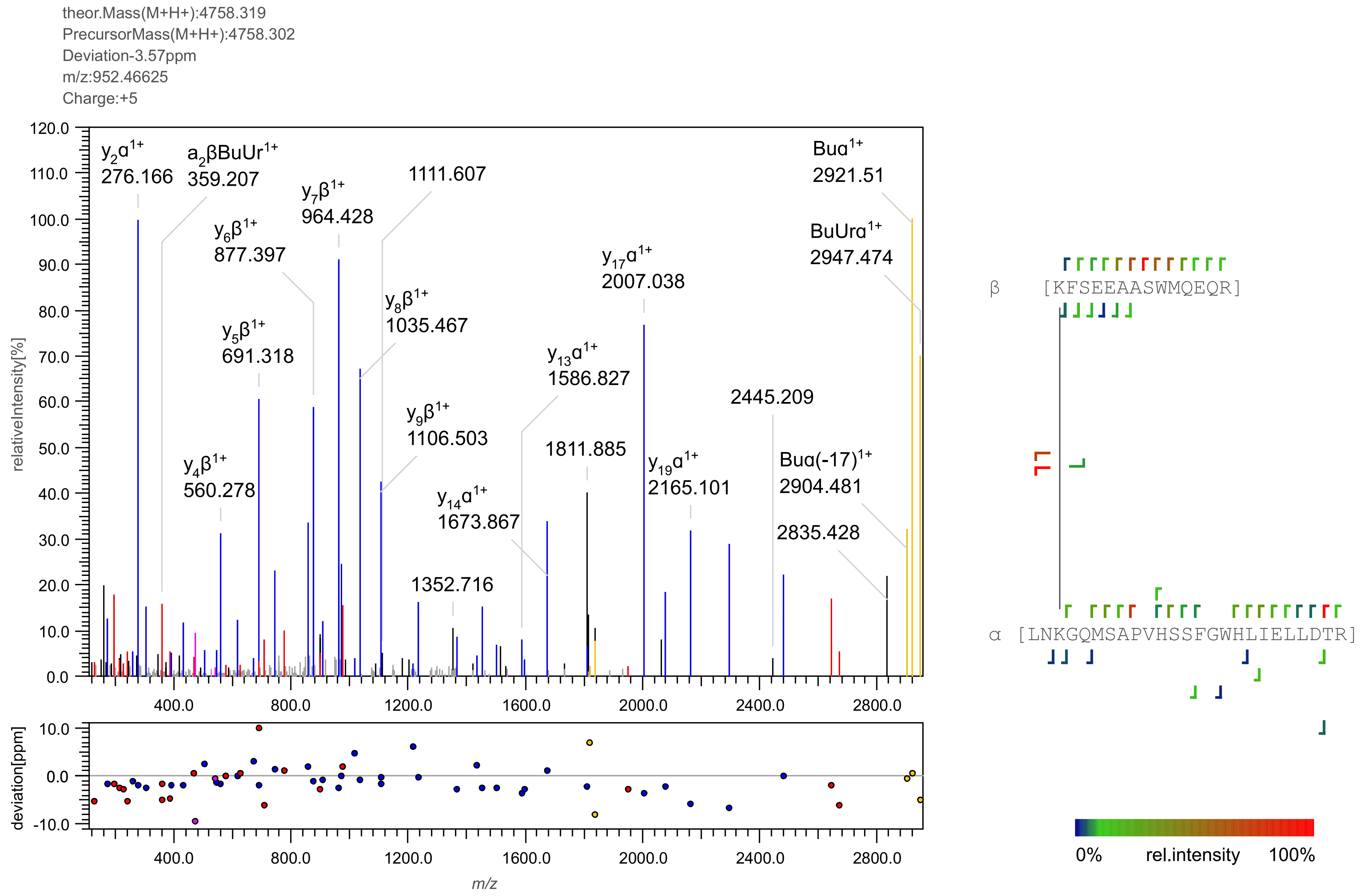
theor.Mass(M+H+):4758.319
PrecursorMass(M+H+):4758.302
Deviation-3.57ppm
m/z:952.46625
Charge:+5
relativeIntensity[%]
deviation[ppm]
m/z
$y_2\alpha^{1+}$ 276.166
$a_2\beta BuUr^{1+}$ 359.207
$y_4\beta^{1+}$ 560.278
$y_5\beta^{1+}$ 691.318
$y_6\beta^{1+}$ 877.397
$y_7\beta^{1+}$ 964.428
$y_8\beta^{1+}$ 1035.467
$y_9\beta^{1+}$ 1106.503
1111.607
1352.716
$y_{13}\alpha^{1+}$ 1586.827
$y_{14}\alpha^{1+}$ 1673.867
1811.885
$y_{17}\alpha^{1+}$ 2007.038
$y_{19}\alpha^{1+}$ 2165.101
2445.209
2835.428
$Bu\alpha(-17)^{1+}$ 2904.481
$Bu\alpha^{1+}$ 2921.51
$BuUr\alpha^{1+}$ 2947.474
β [KFSEEAASWMQEQR]
α [LNKGQMSAPVHSSFGWHLIELLDTR]
5+ 4+ 3+ 2+ 1+
0% rel.intensity 100%

XL-MS spectra of DSBU crosslinks detected between SurA and OmpX.

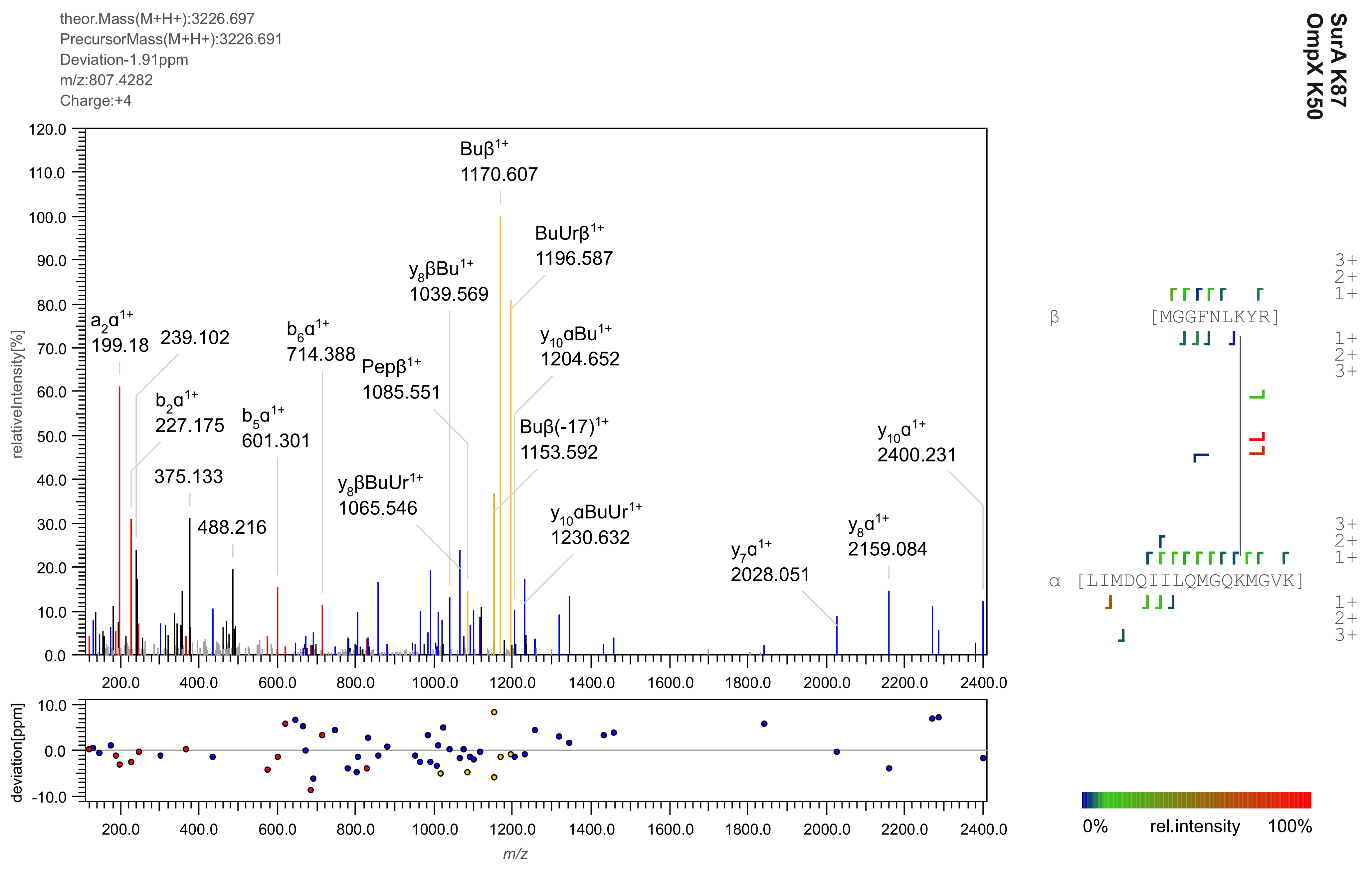
SurA K87
OmpX K50
theor.Mass(M+H+):3226.697
PrecursorMass(M+H+):3226.691
Deviation-1.91ppm
m/z:807.4282
Charge:+4
relativeIntensity[%]
deviation[ppm]
m/z
a2α1+ 199.18
239.102
b2α1+ 227.175
375.133
488.216
b5α1+ 601.301
b6α1+ 714.388
Pepβ1+ 1085.551
y8βBu1+ 1039.569
y8βBuUr1+ 1065.546
Buβ1+ 1170.607
BuUrβ1+ 1196.587
y10αBu1+ 1204.652
Buβ(-17)1+ 1153.592
y10αBuUr1+ 1230.632
y7α1+ 2028.051
y8α1+ 2159.084
y10α1+ 2400.231
β [MGGFNLKYR]
α [LIMDQIILQMGQKMGVK]
1+ 2+ 3+
0% rel.intensity 100%

SurA K87
OmpX K82

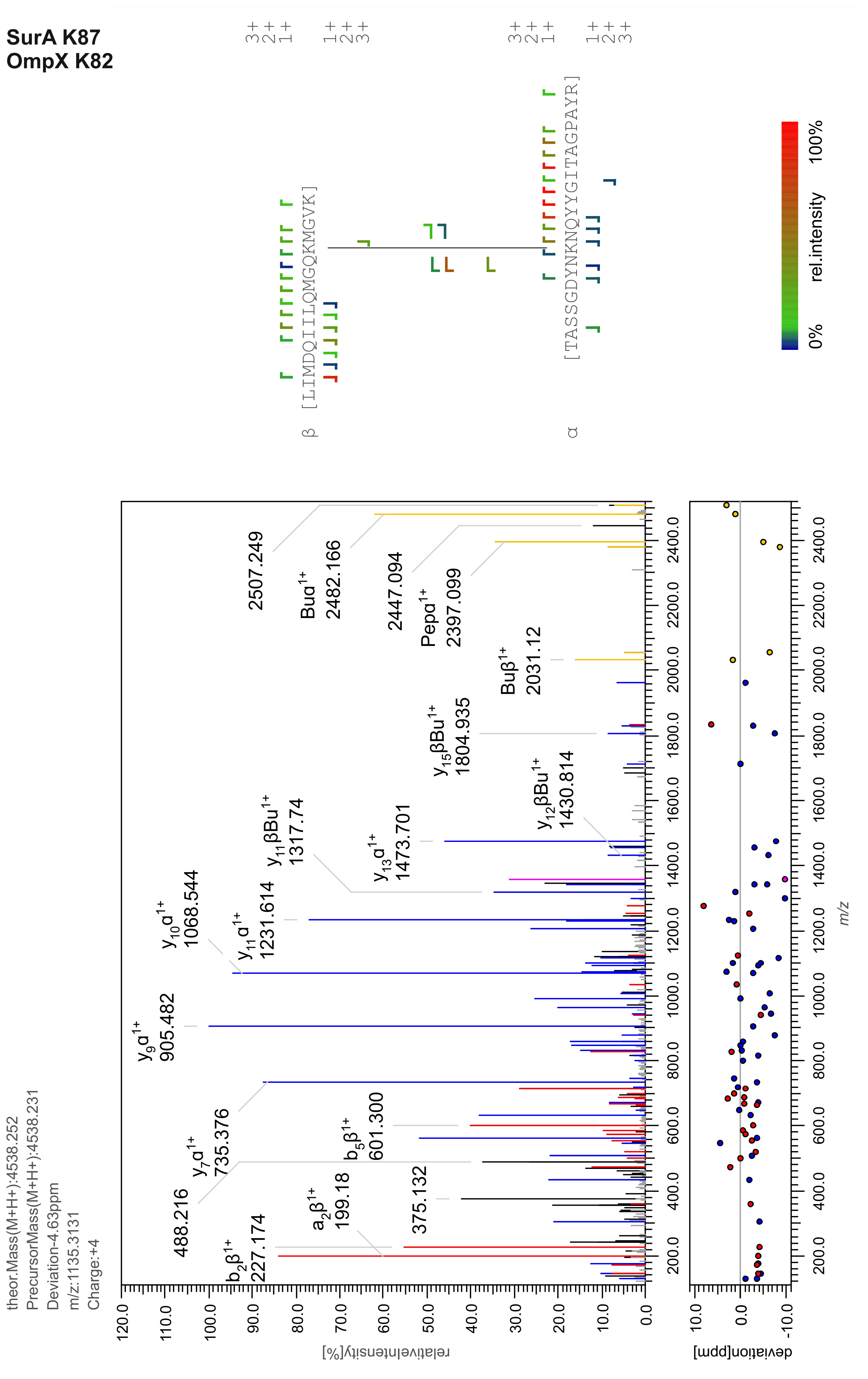

SurA K91
OmpX K82

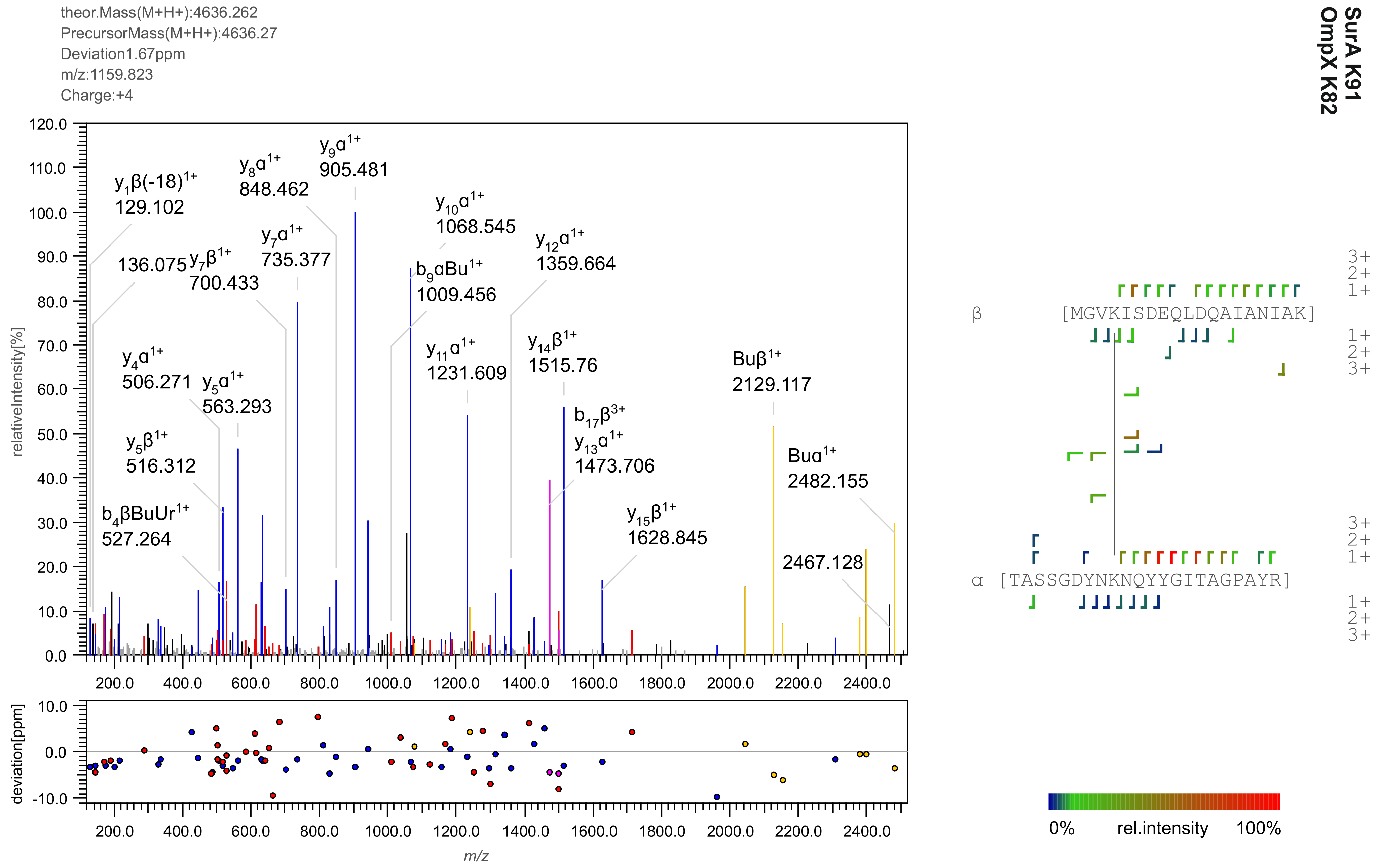

SurA K106
OmpX K82

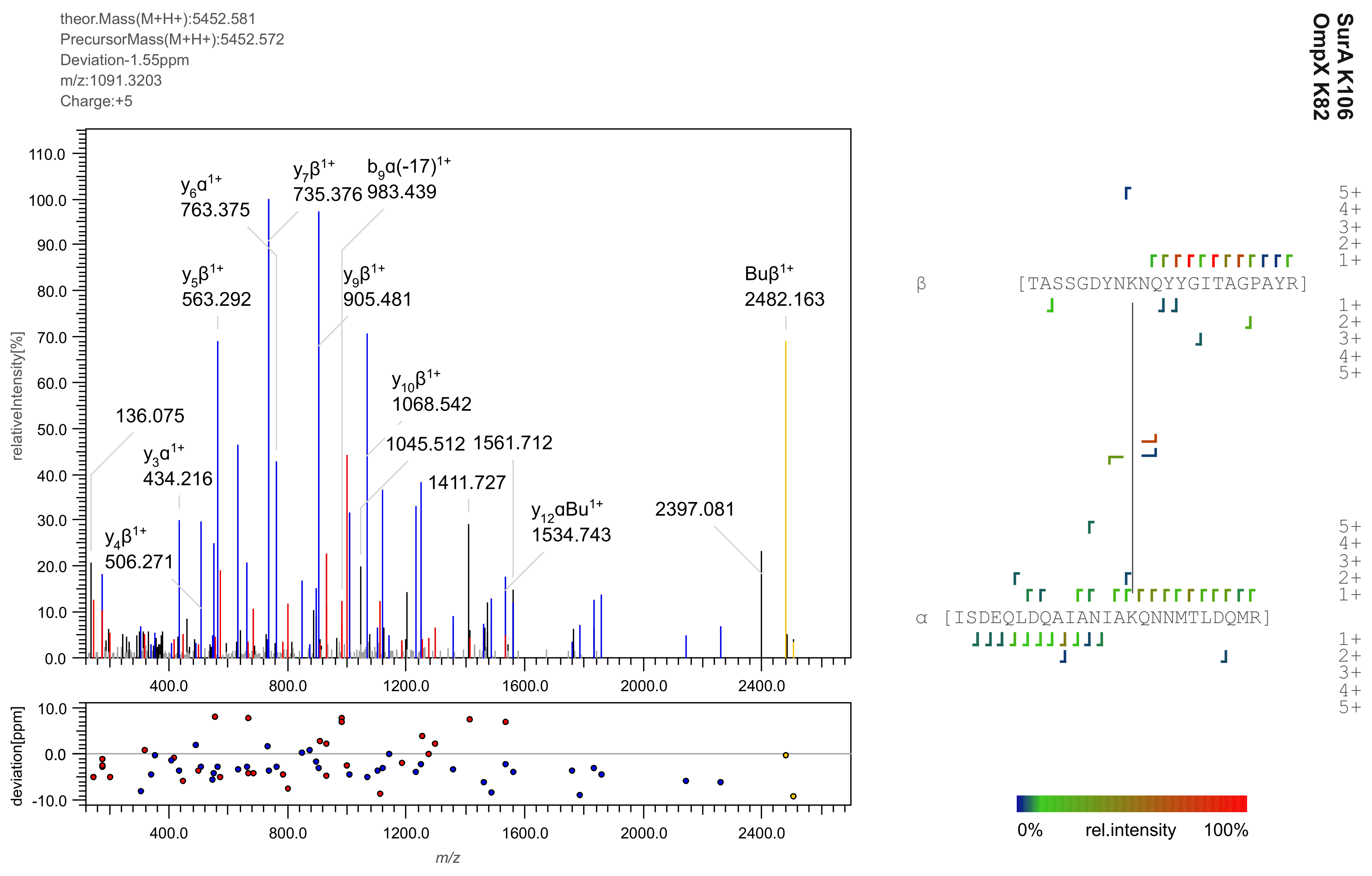

SurA K135
OmpX K50

theor.Mass(M+H+):2385.242
PrecursorMass(M+H+):2385.228
Deviation-5.85ppm
m/z:597.0624
Charge:+4

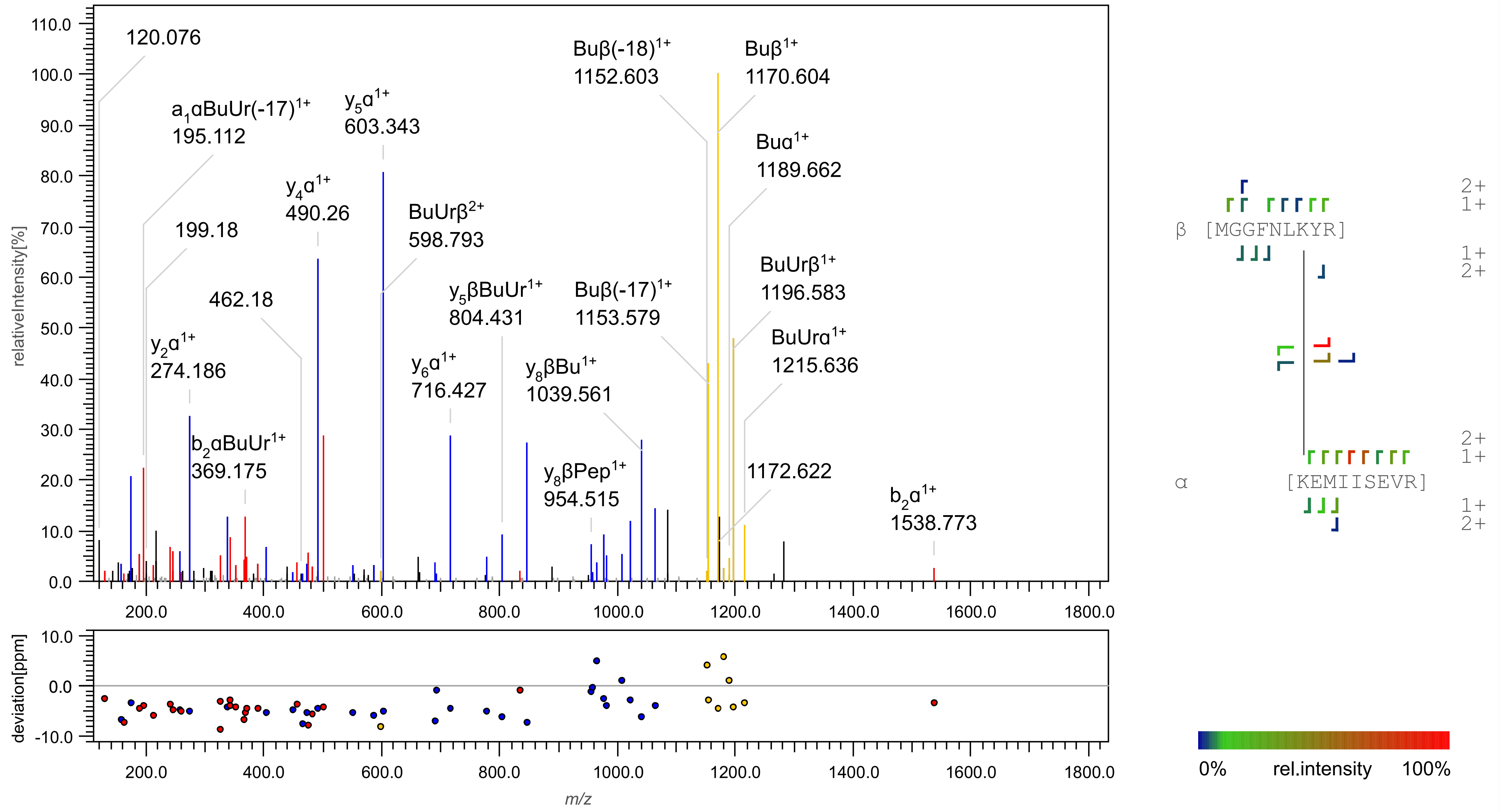

SurA K135
OmpX K71

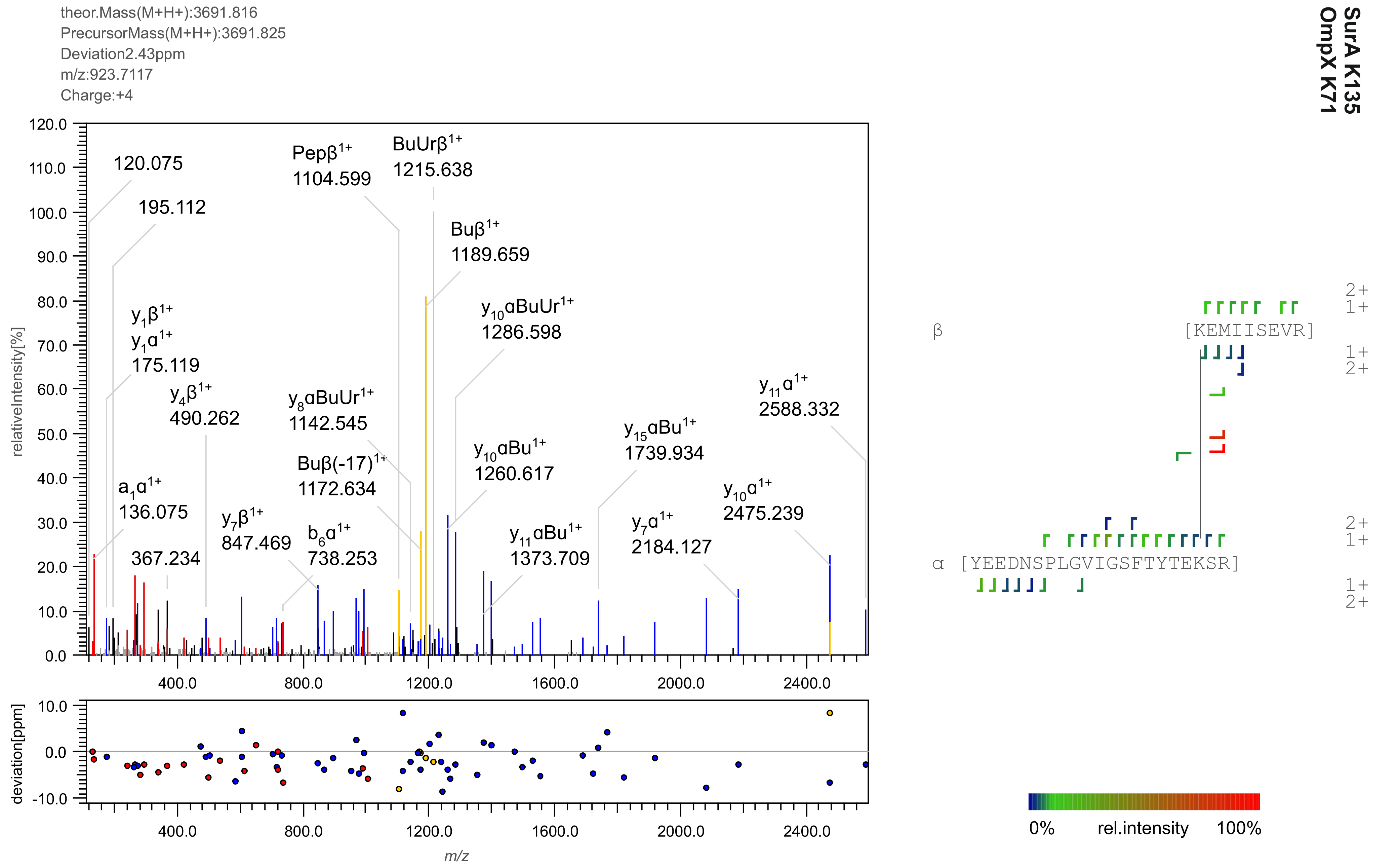

SurA K135
OmpX K82

theor.Mass(M+H+):3696.796
PrecursorMass(M+H+):3696.787
Deviation-2.37ppm
m/z:924.95233
Charge:+4

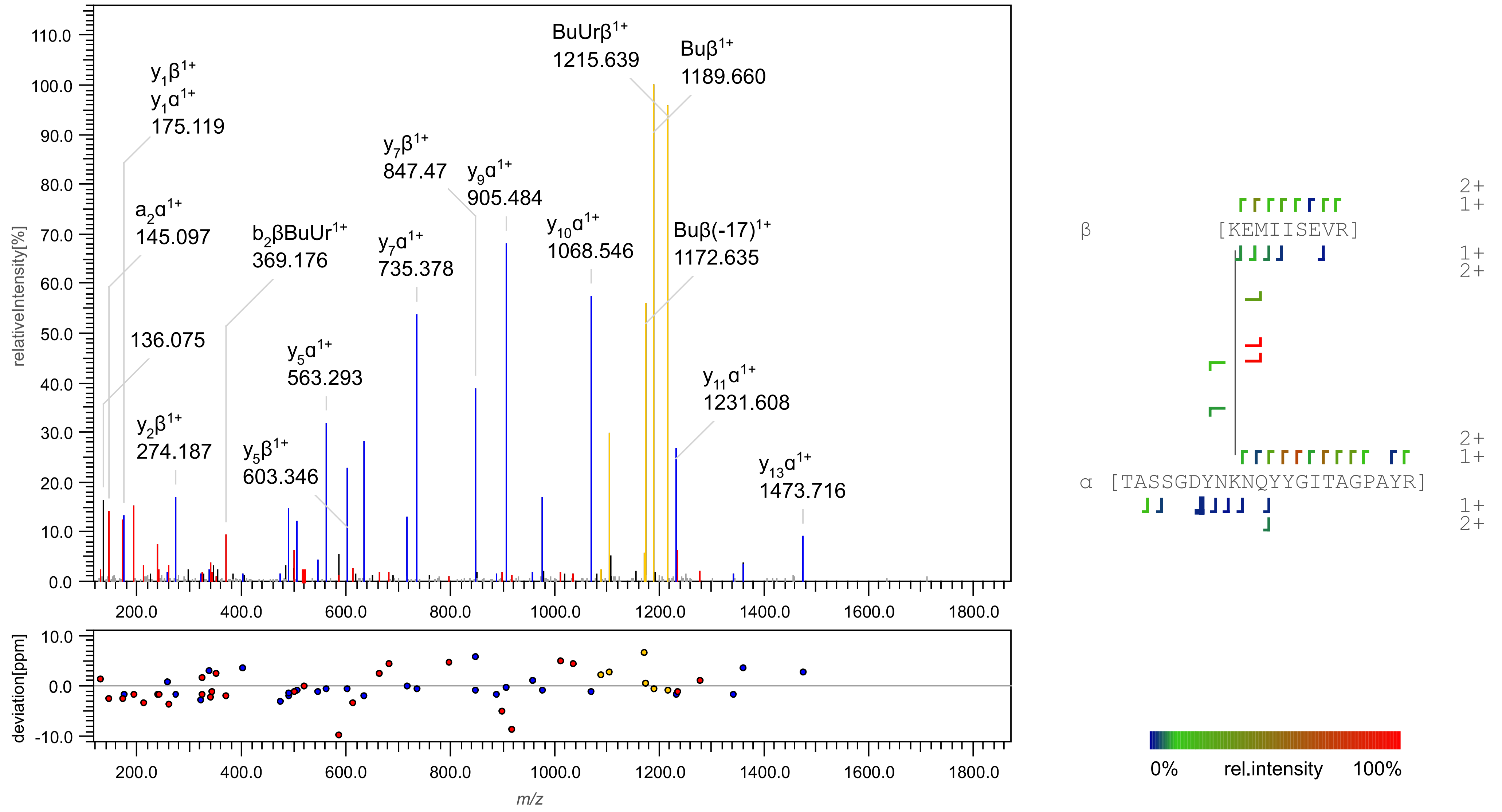

SurA K253
OmpX K71

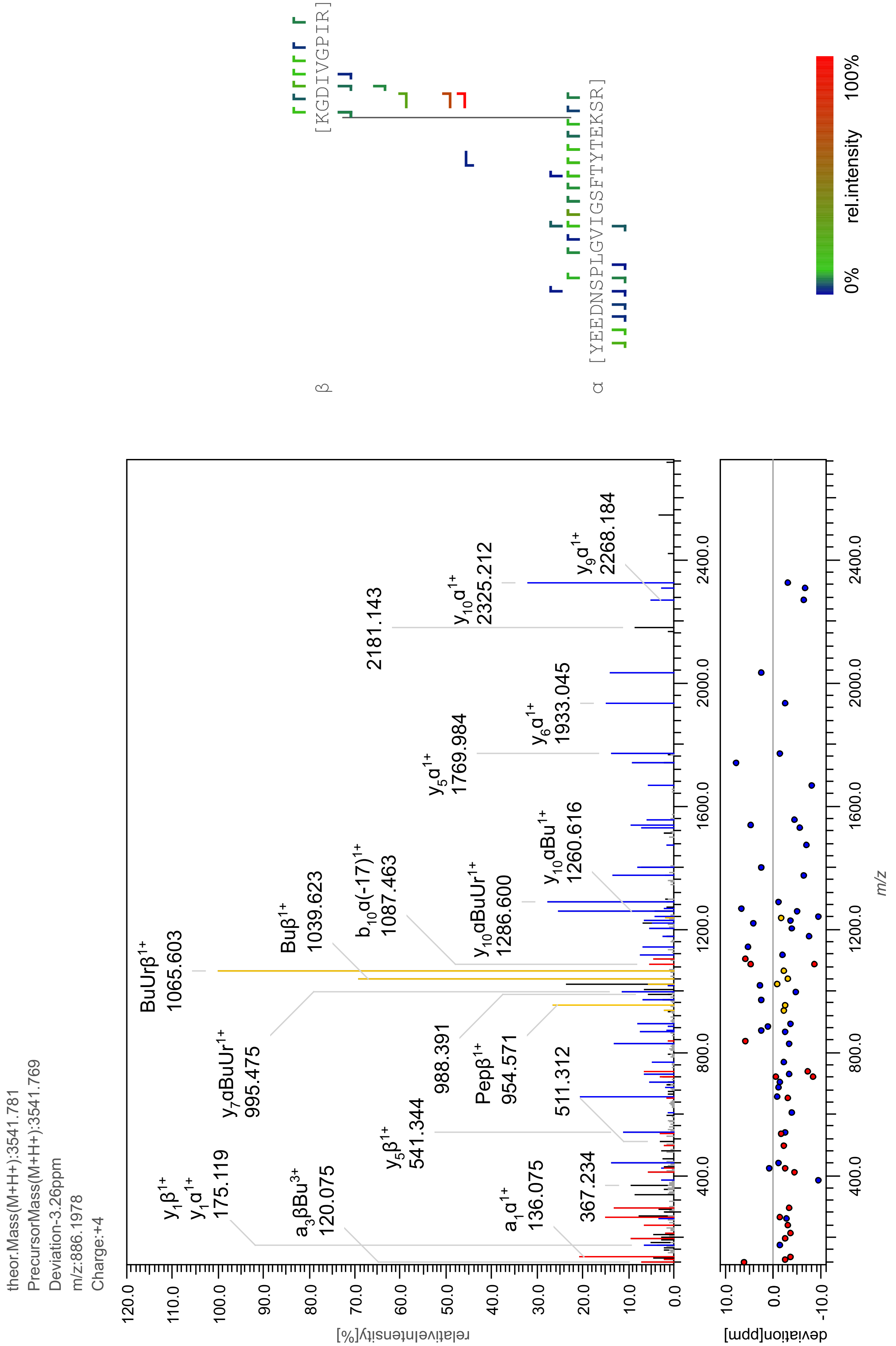

SurA K253
OmpX K82

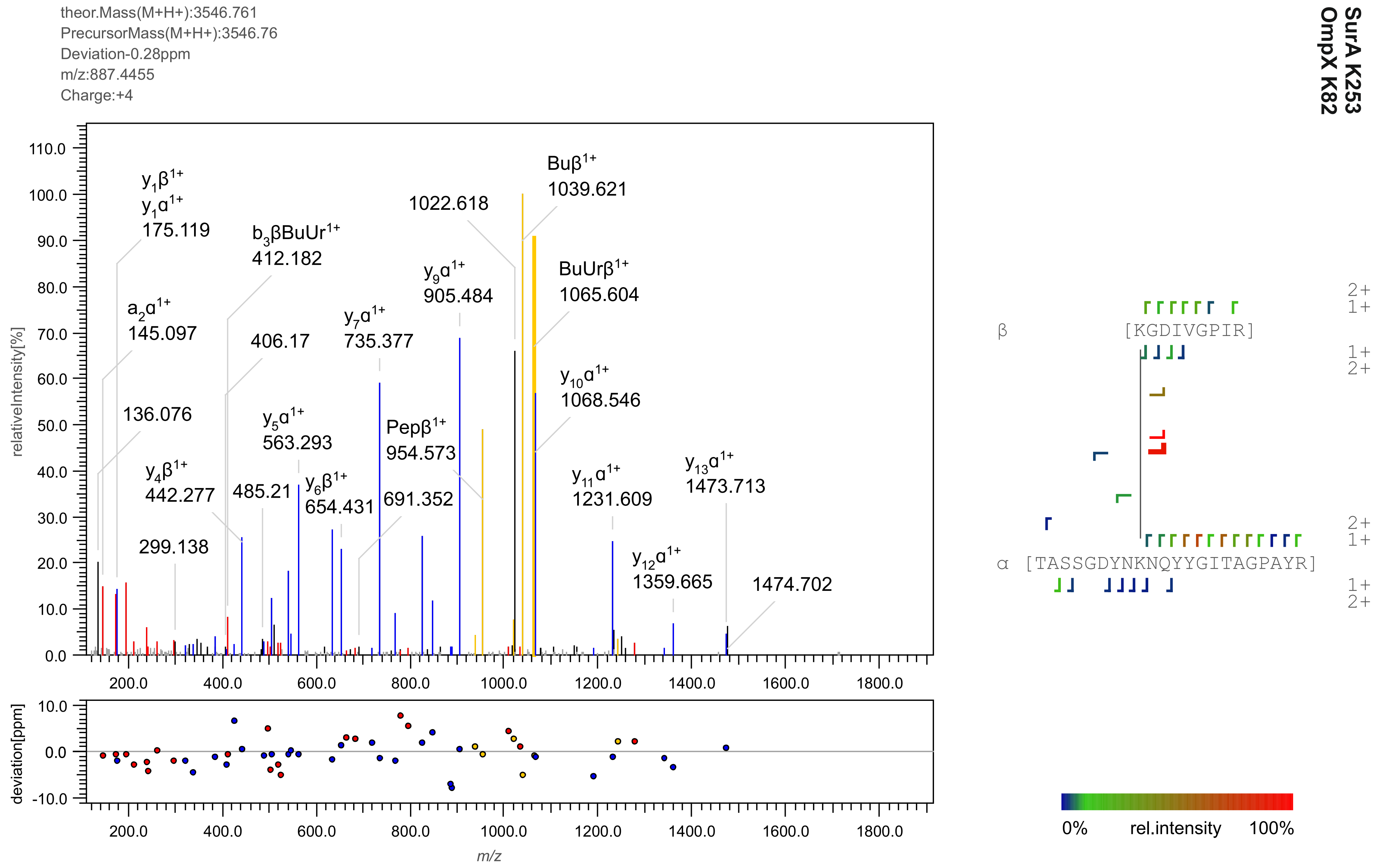

SurA K279
OmpX K50

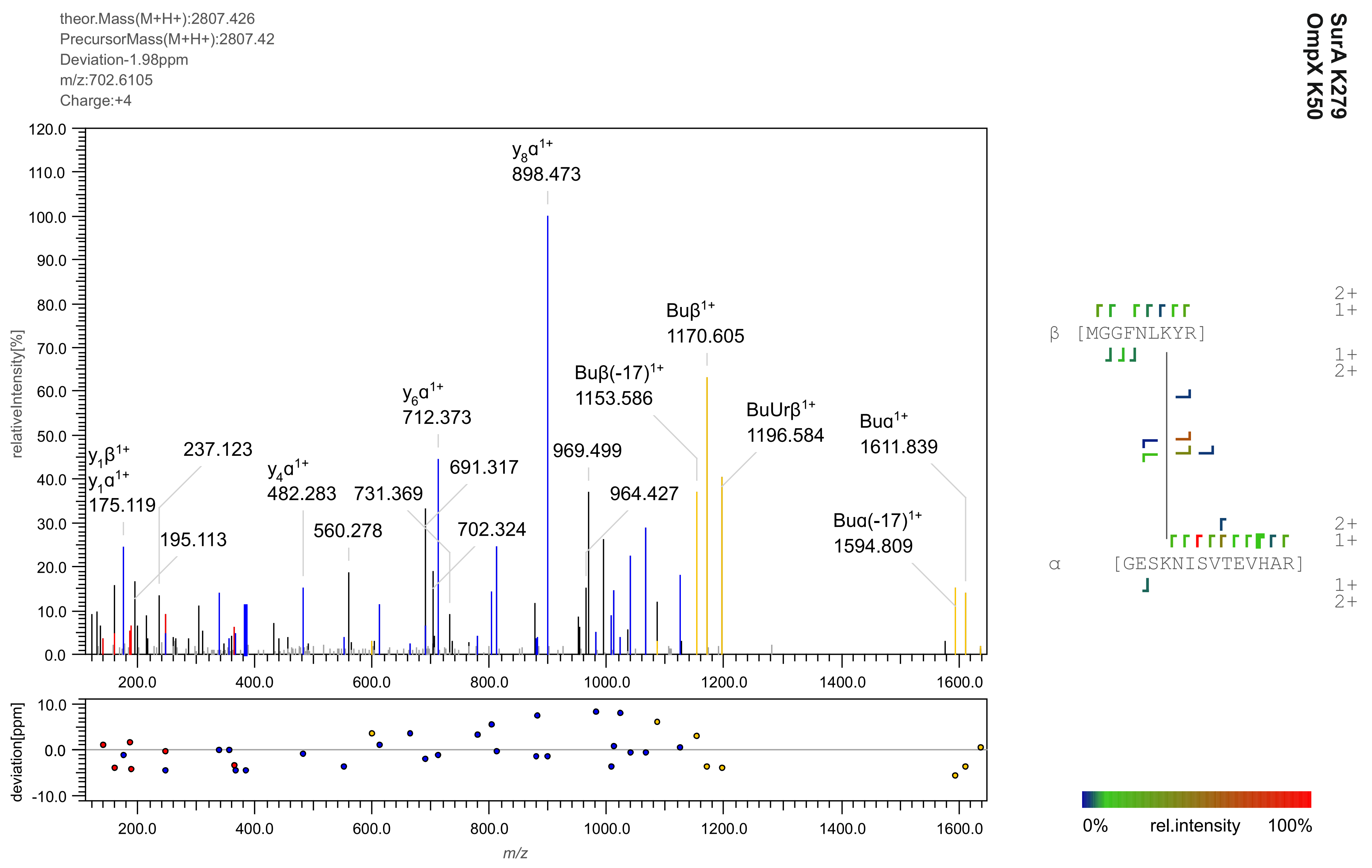

SurA K279
OmpX K82

theor.Mass(M+H+):4118.979
PrecursorMass(M+H+):4118.957
Deviation-5.49ppm
m/z:824.59735
Charge:+5

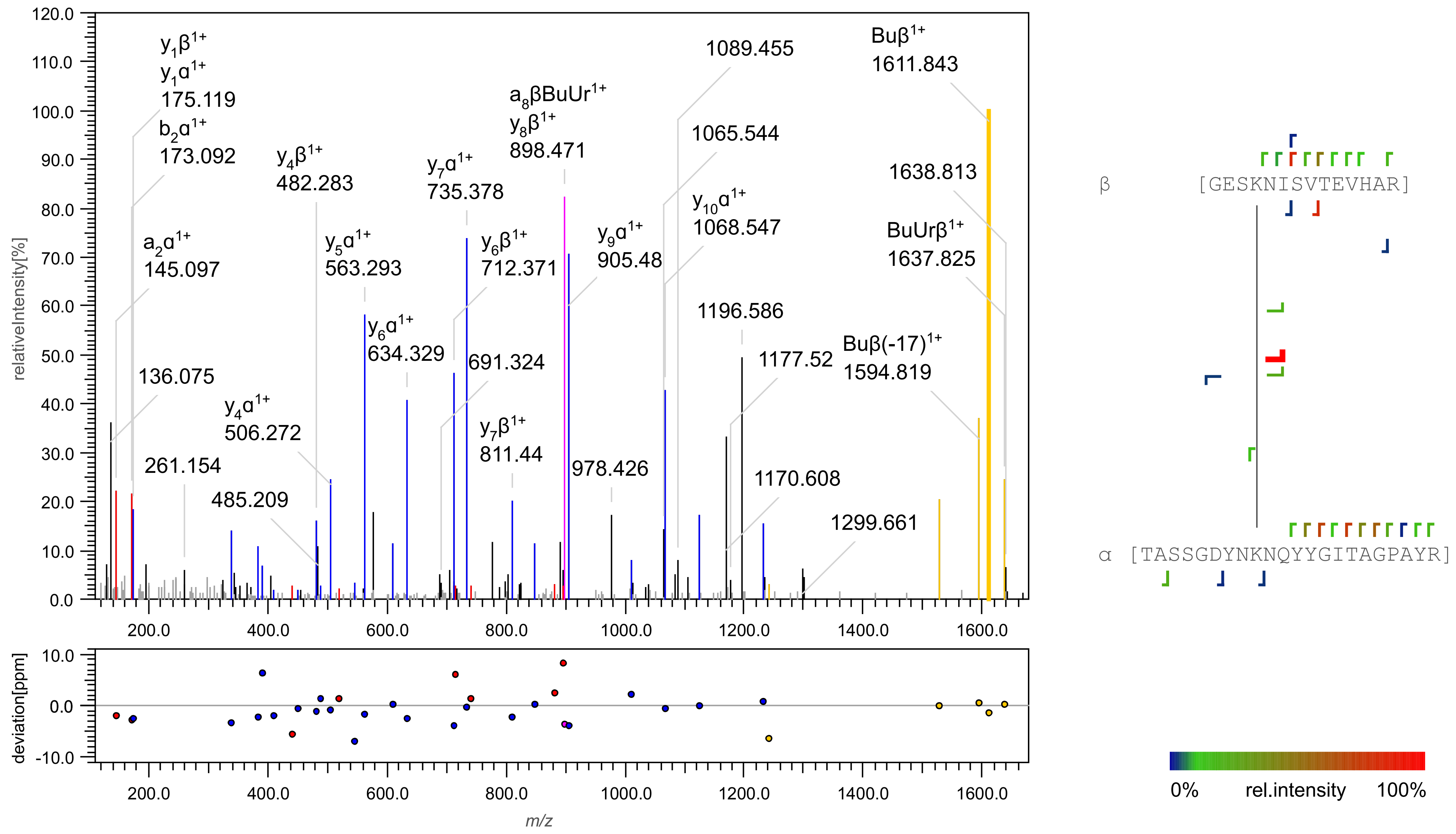

SurA K294
OmpX K50

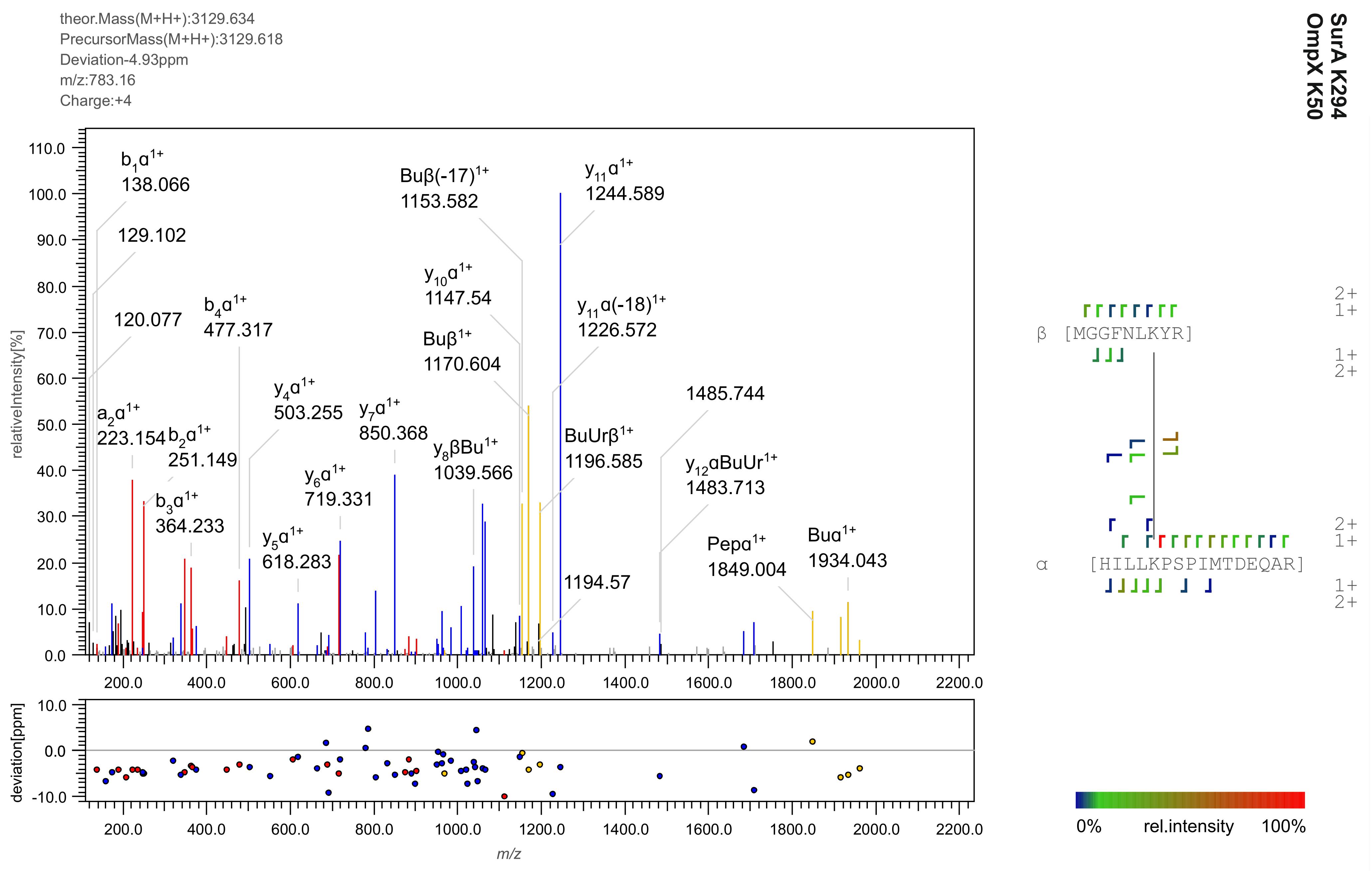

SurA K294
OmpX K71

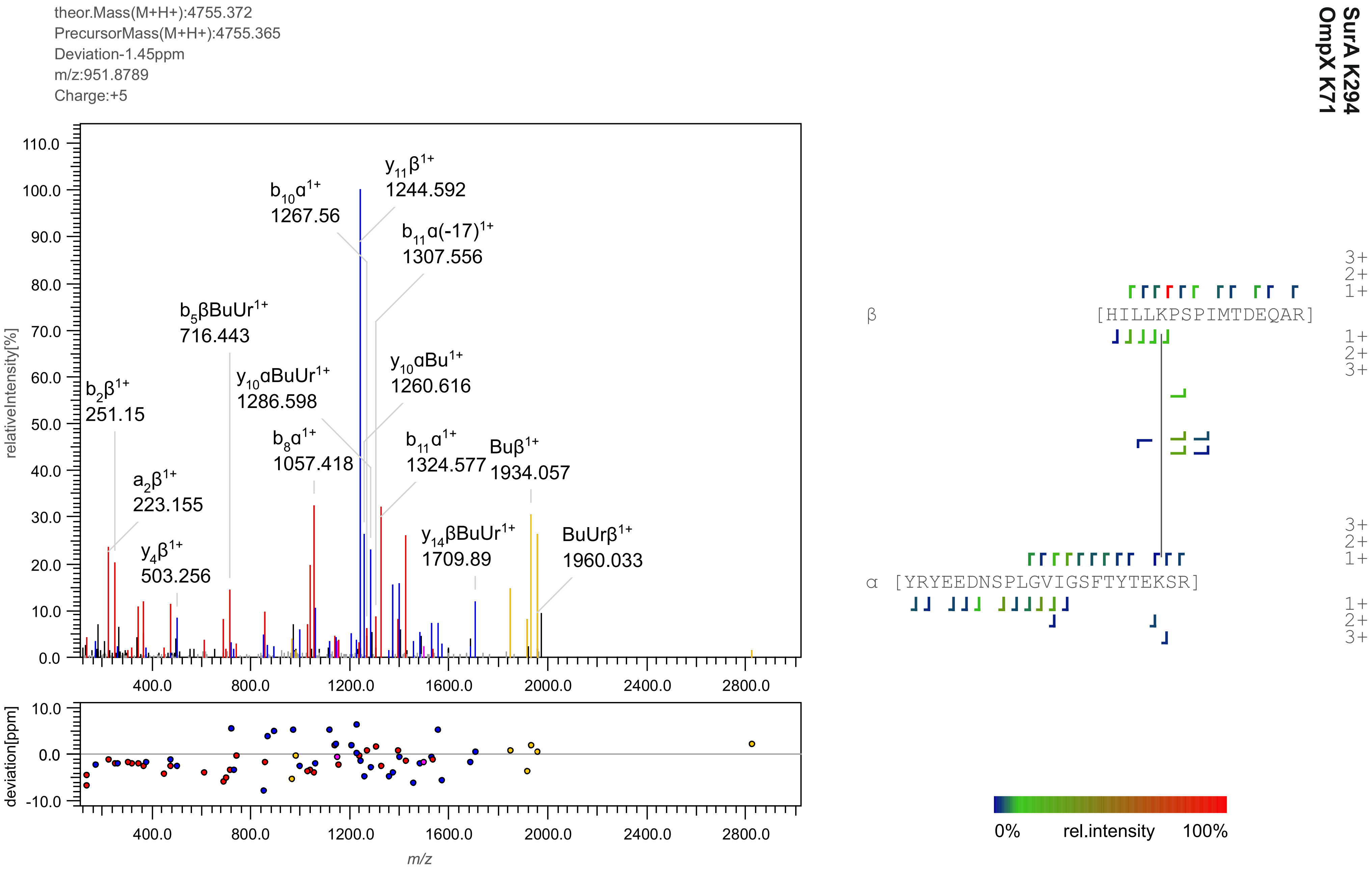

SurA K294
OmpX K82

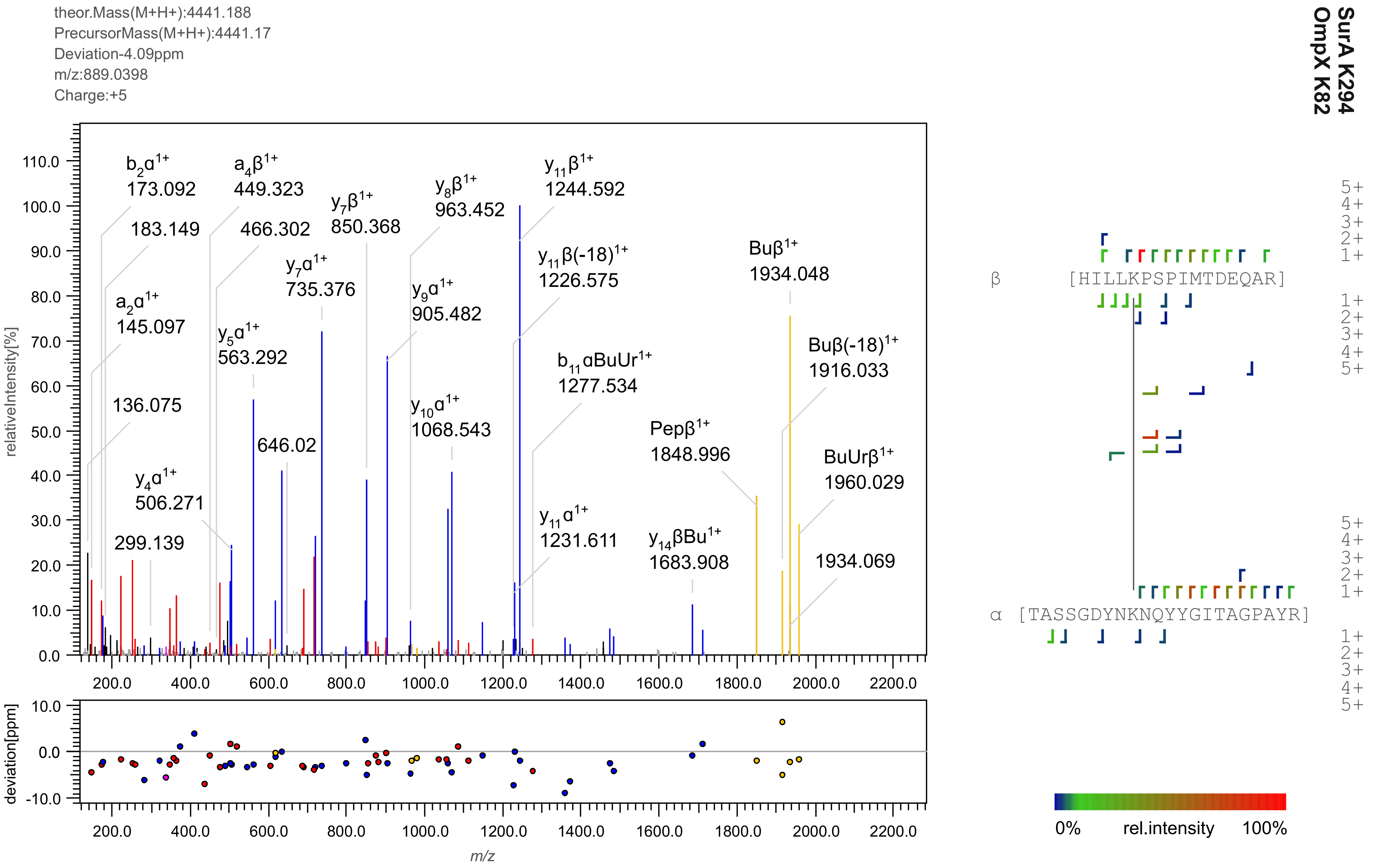

SurA K307
OmpX K50

theor.Mass(M+H+):2508.364
PrecursorMass(M+H+):2508.352
Deviation-4.93ppm
m/z:627.84344
Charge:+4

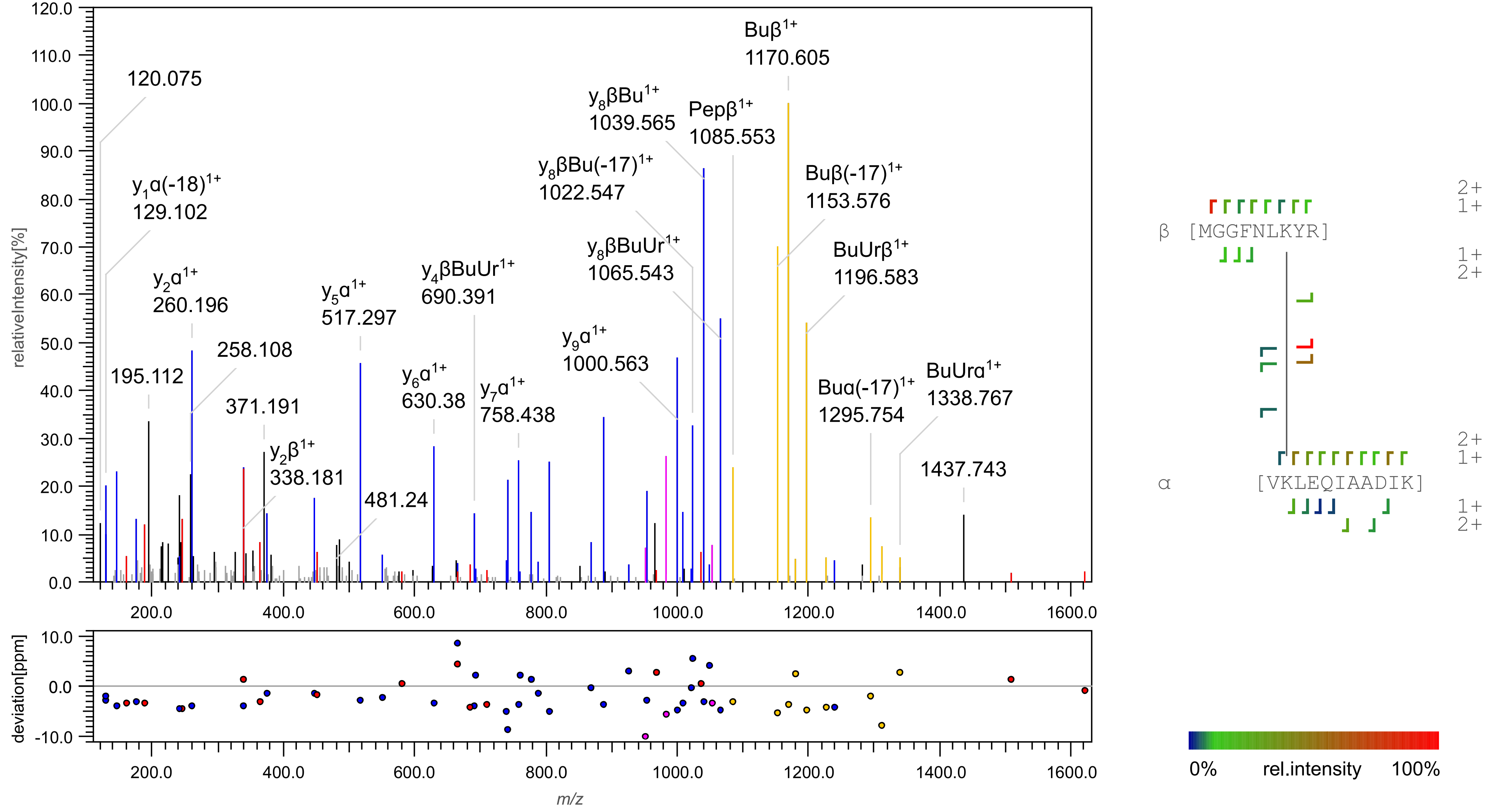

SurA K307
OmpX K82

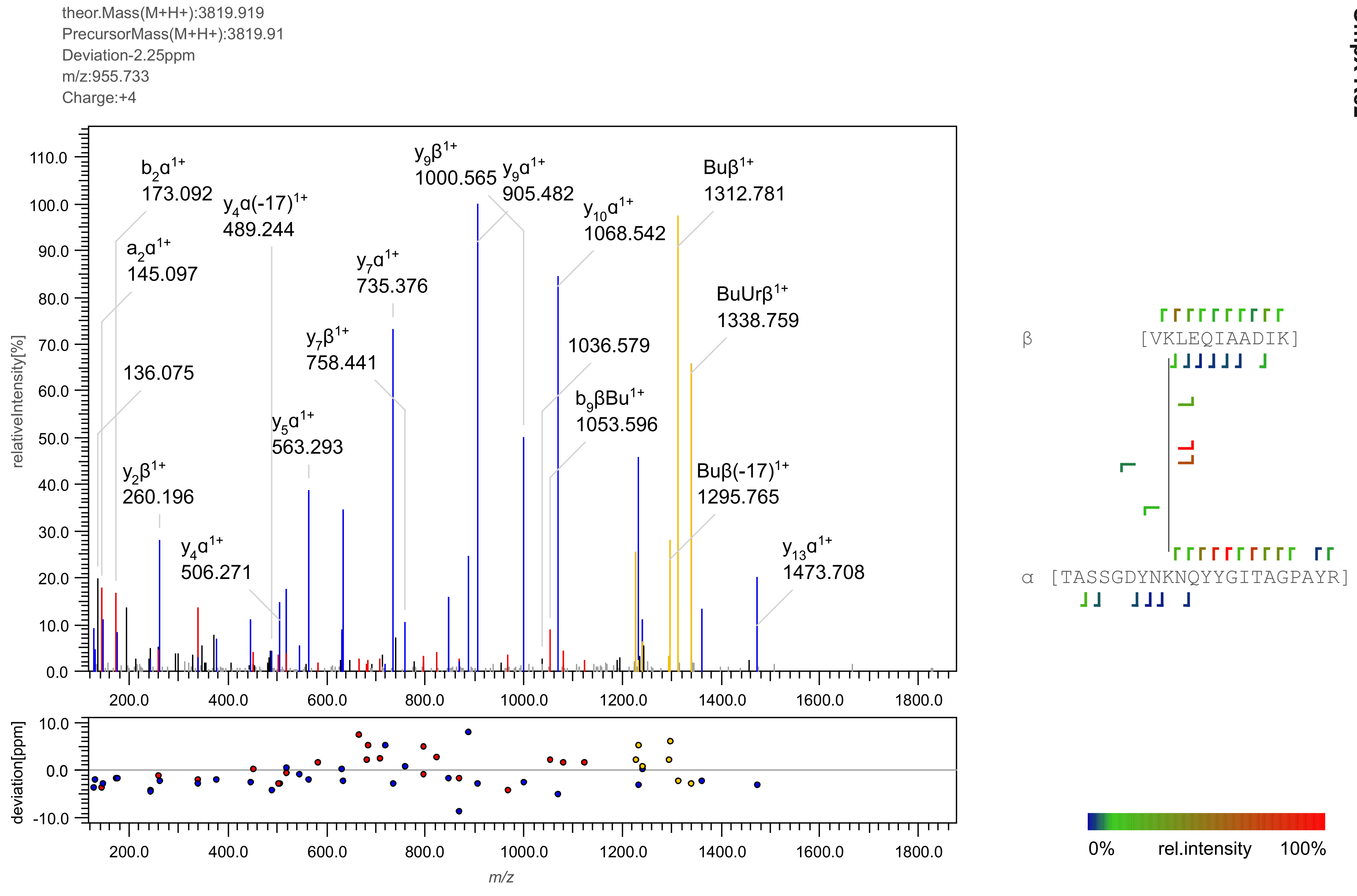

SurA K316
OmpX K82

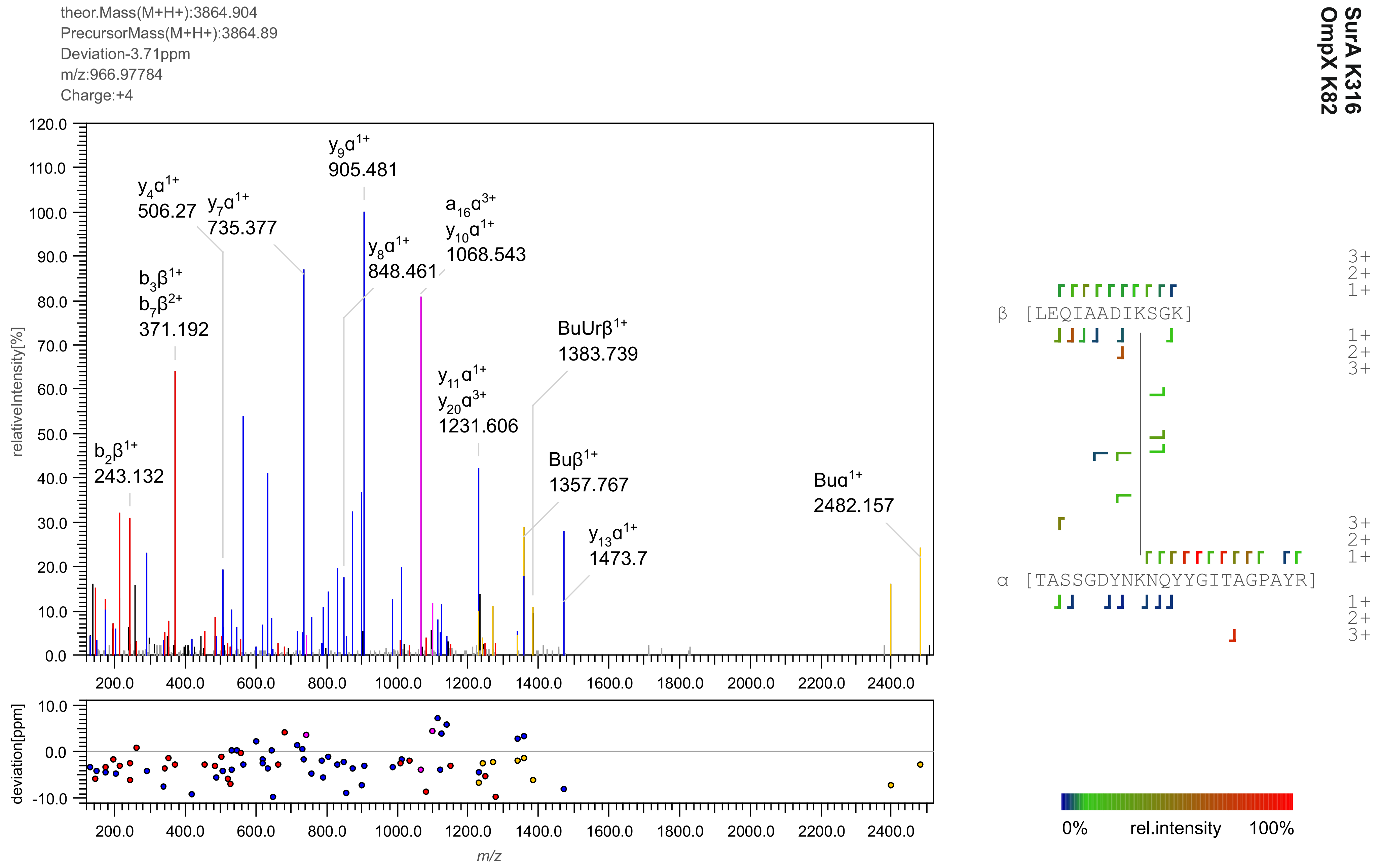

SurA K389
OmpX K50

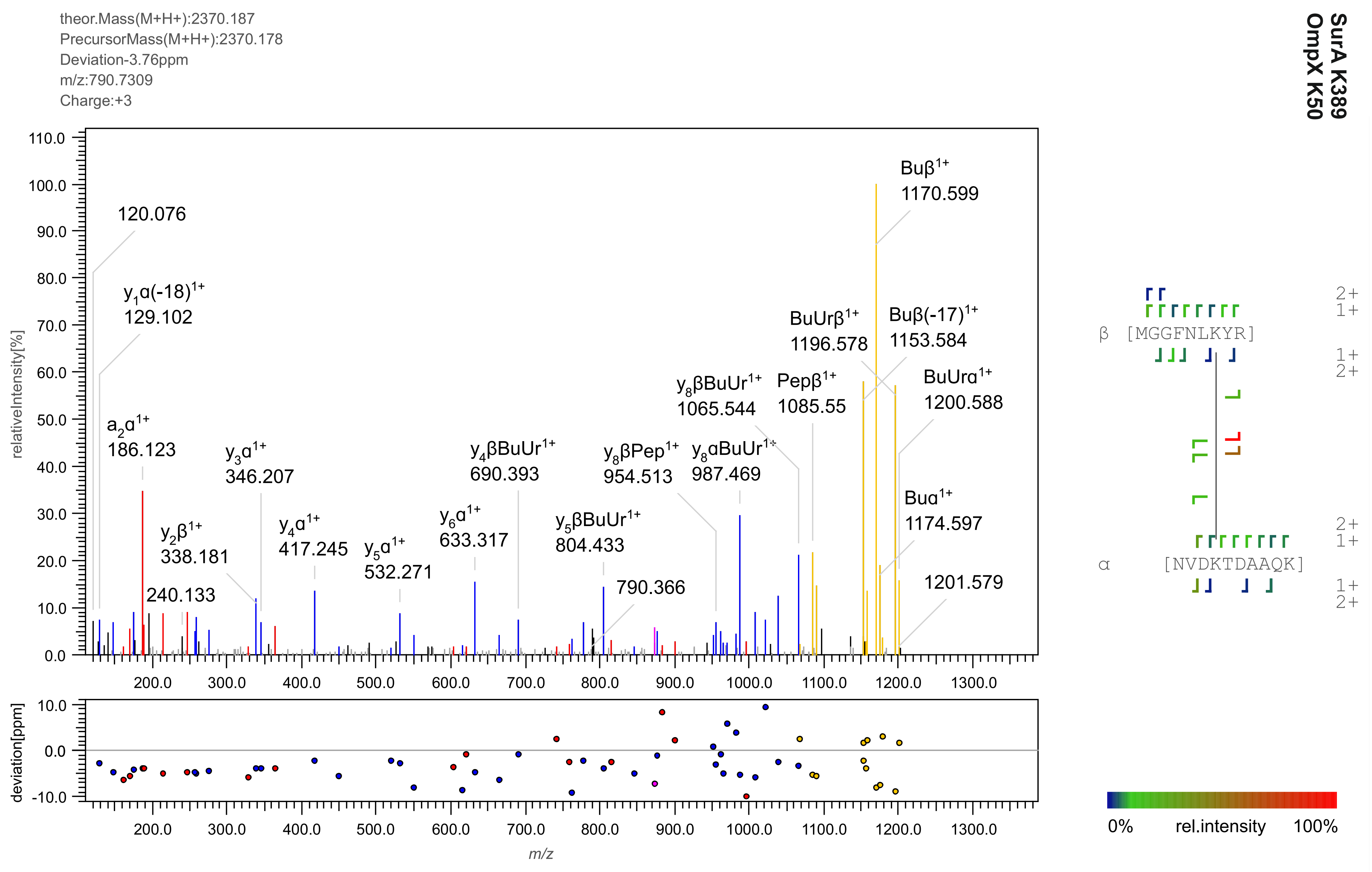

SurA K389
OmpX K71

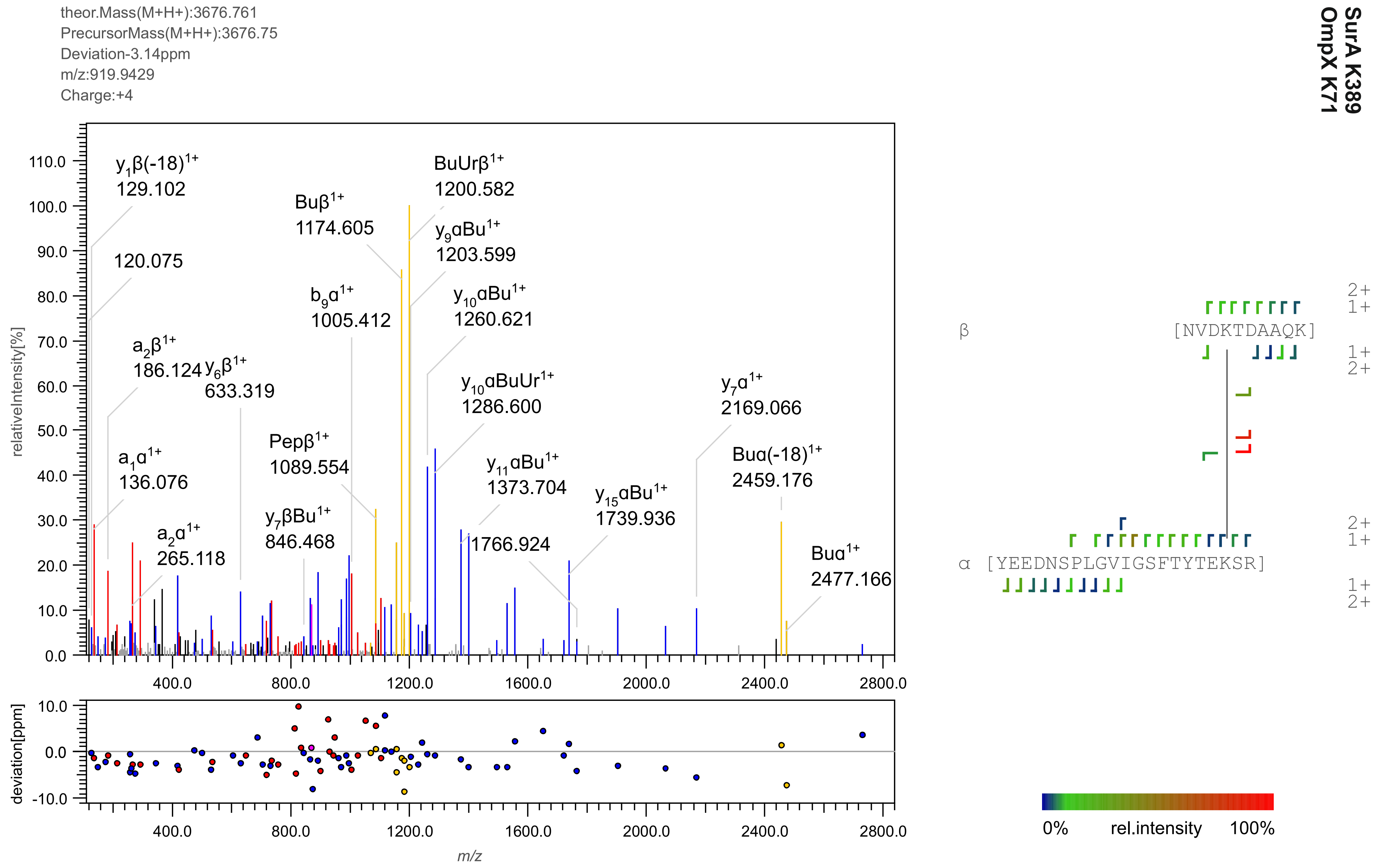

SurA K389
OmpX K82

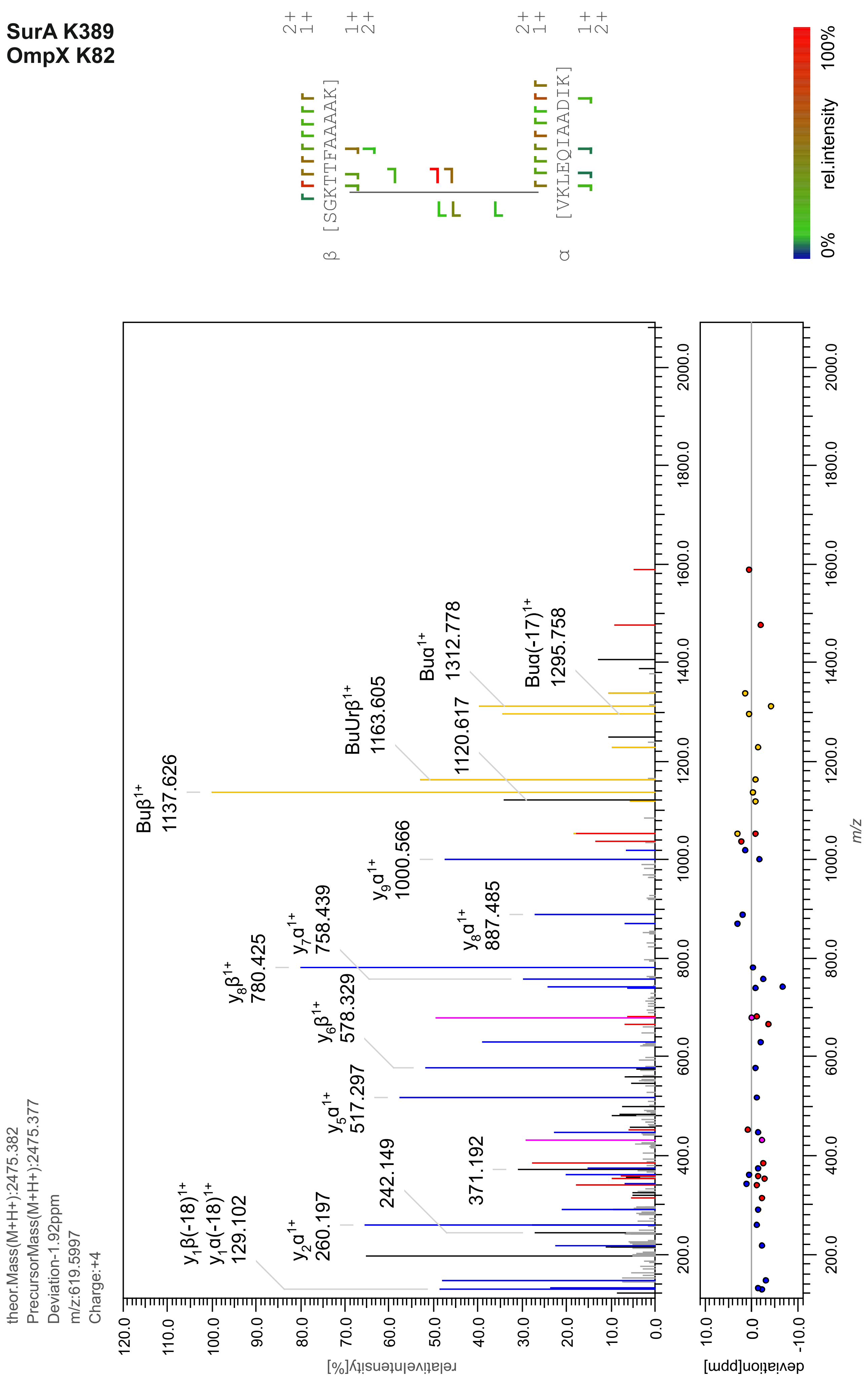

SurA K395
OmpX K50

theor.Mass(M+H+):2185.082
PrecursorMass(M+H+):2185.075
Deviation-3.07ppm
m/z:729.0299
Charge:+3

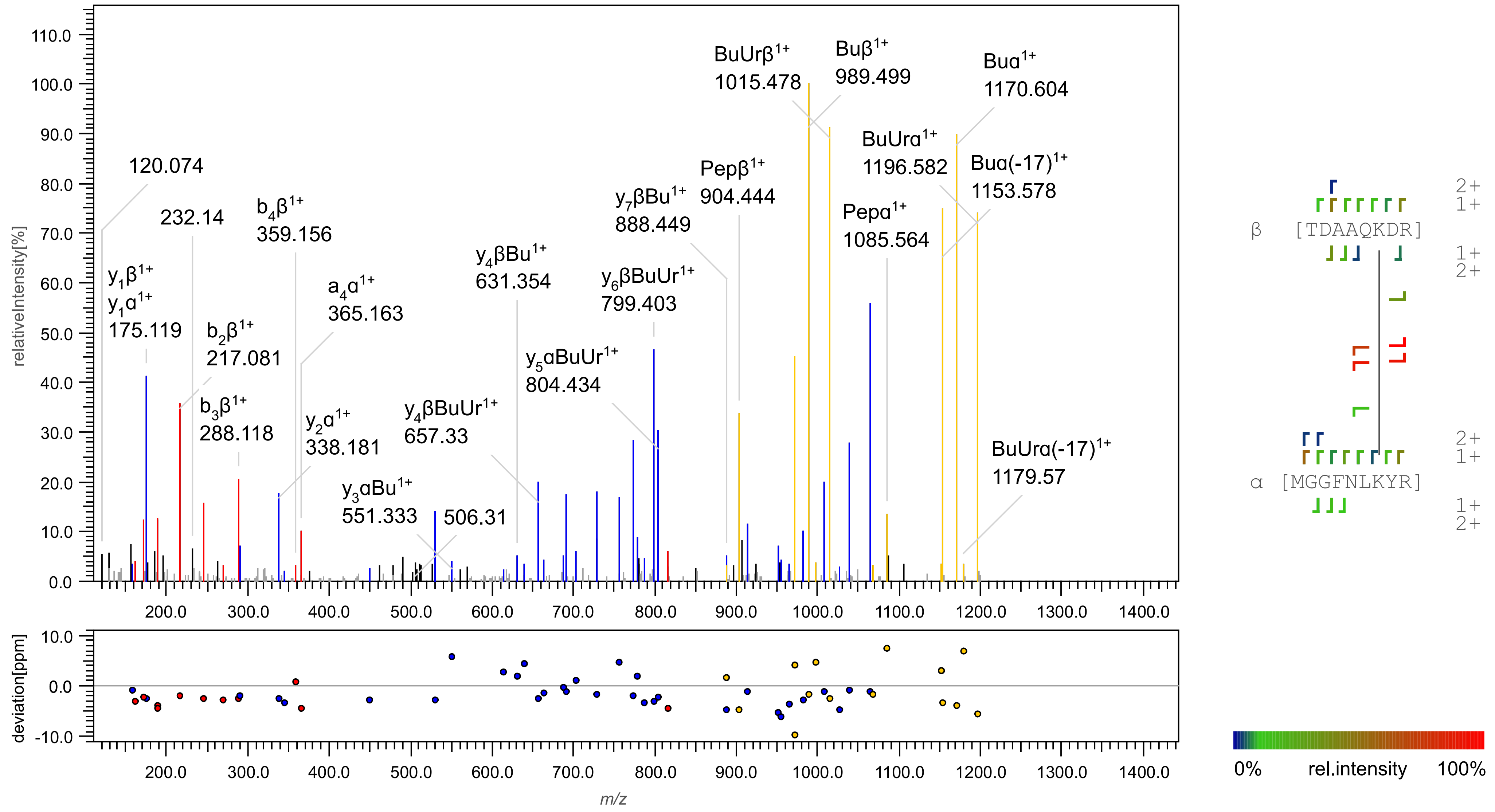

SurA K395
OmpX K71

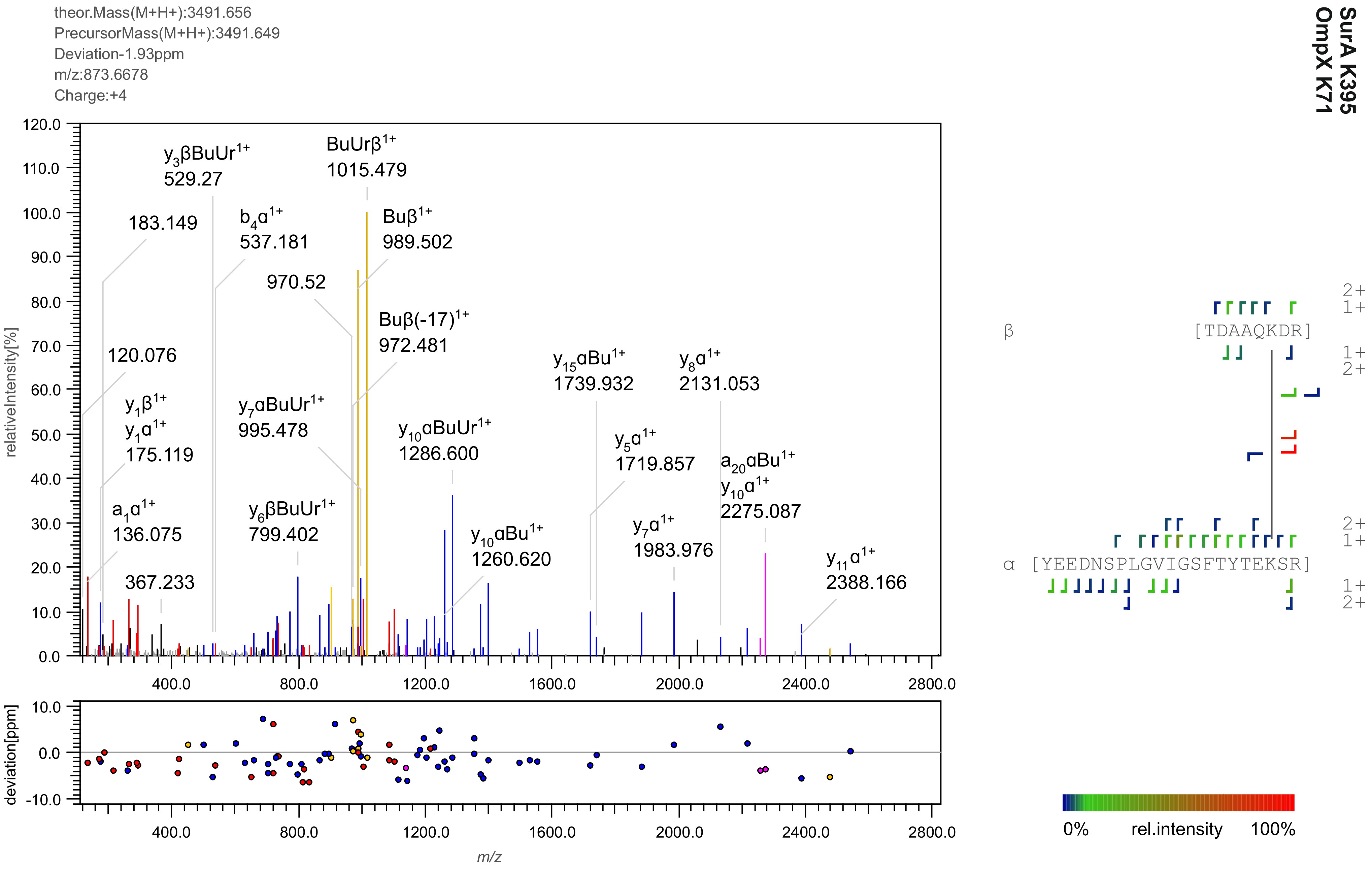

SurA K395
OmpX K82

theor.Mass(M+H+):3496.636
PrecursorMass(M+H+):3496.618
Deviation-5.17ppm
m/z:874.91003
Charge:+4

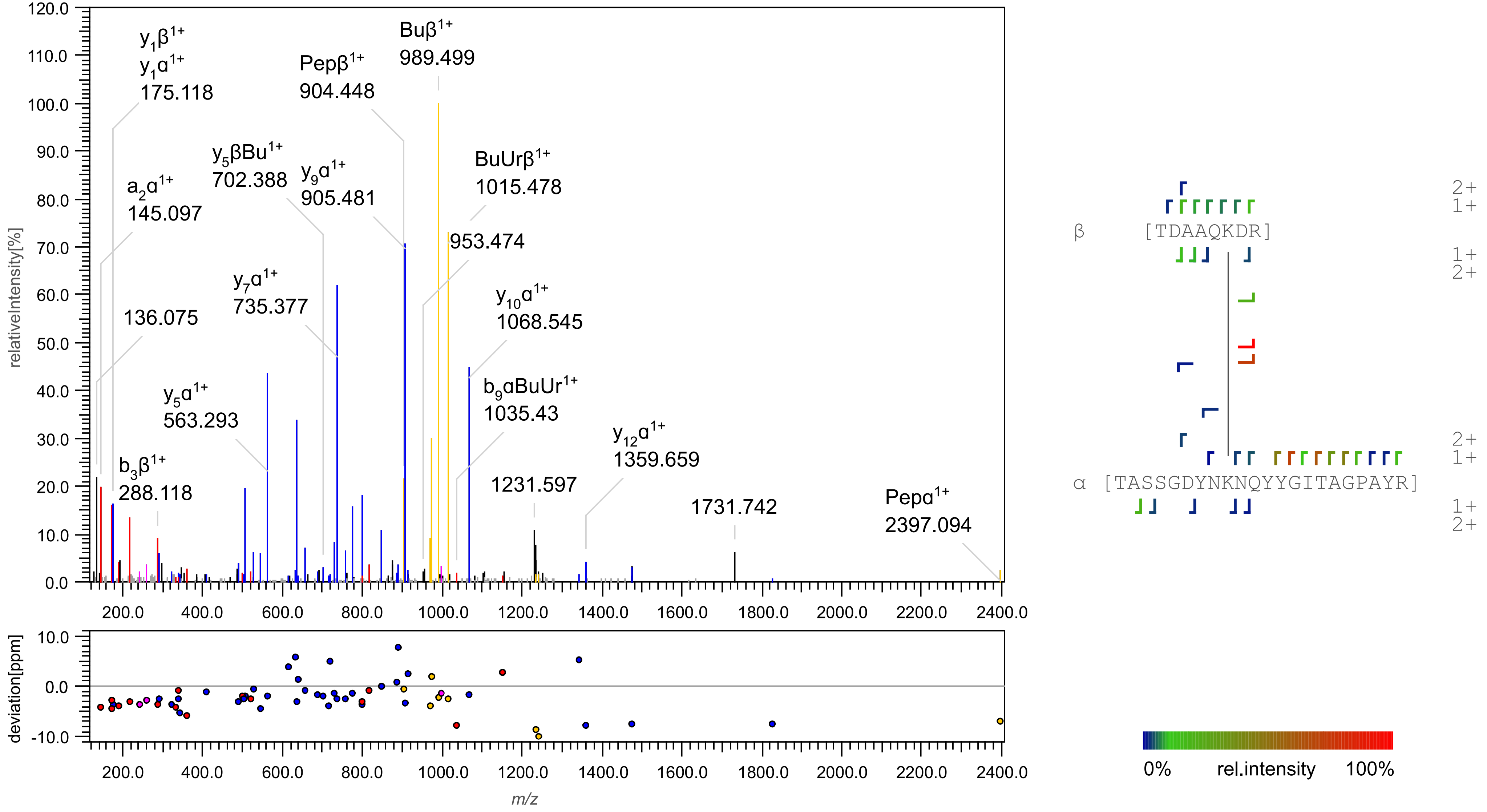

SurA K406
OmpX K71

theor.Mass(M+H+):4313.993
PrecursorMass(M+H+):4313.985
Deviation-1.99ppm
m/z:1079.2516
Charge:+4

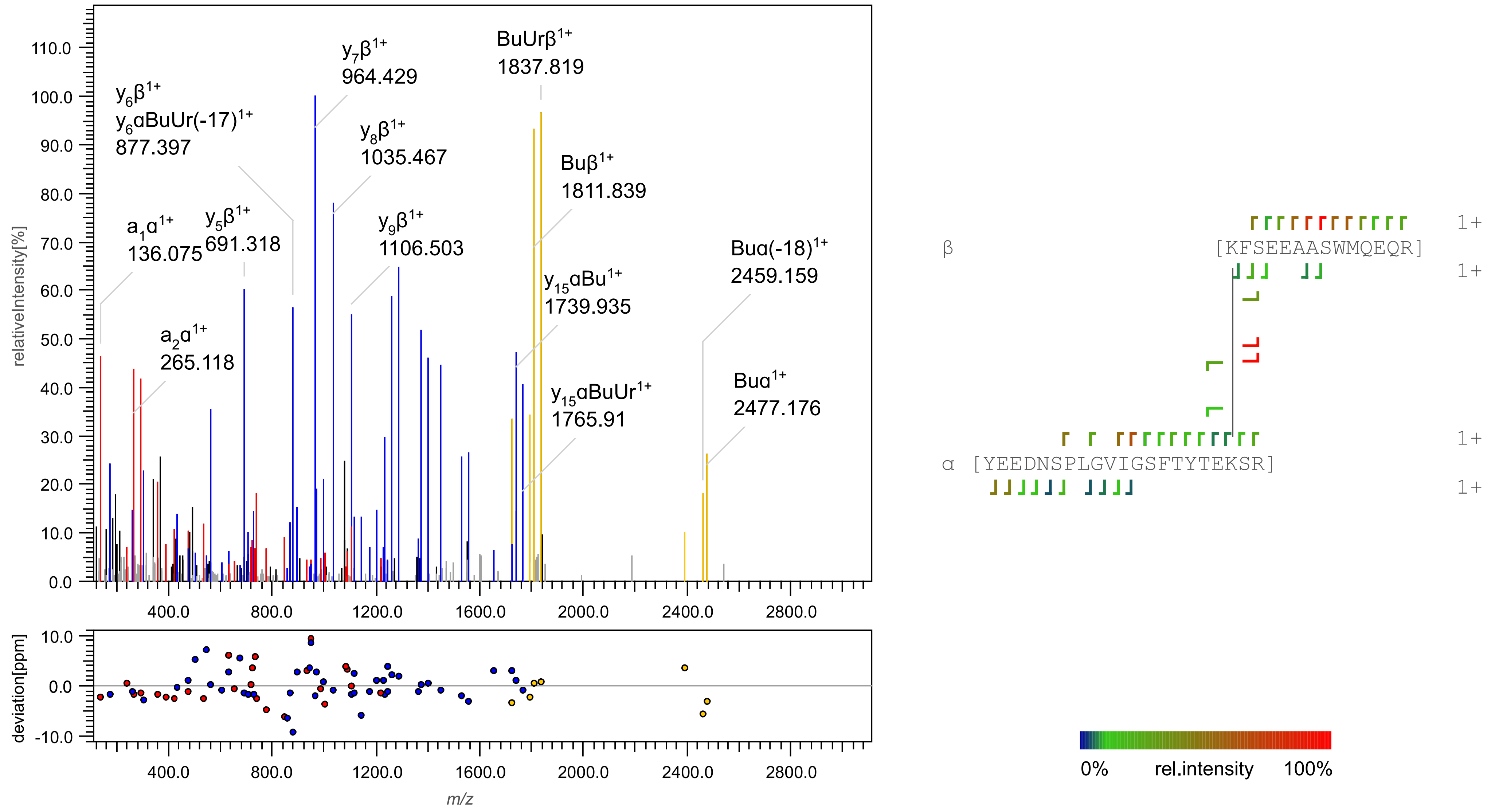

SurA K406
OmpX K82

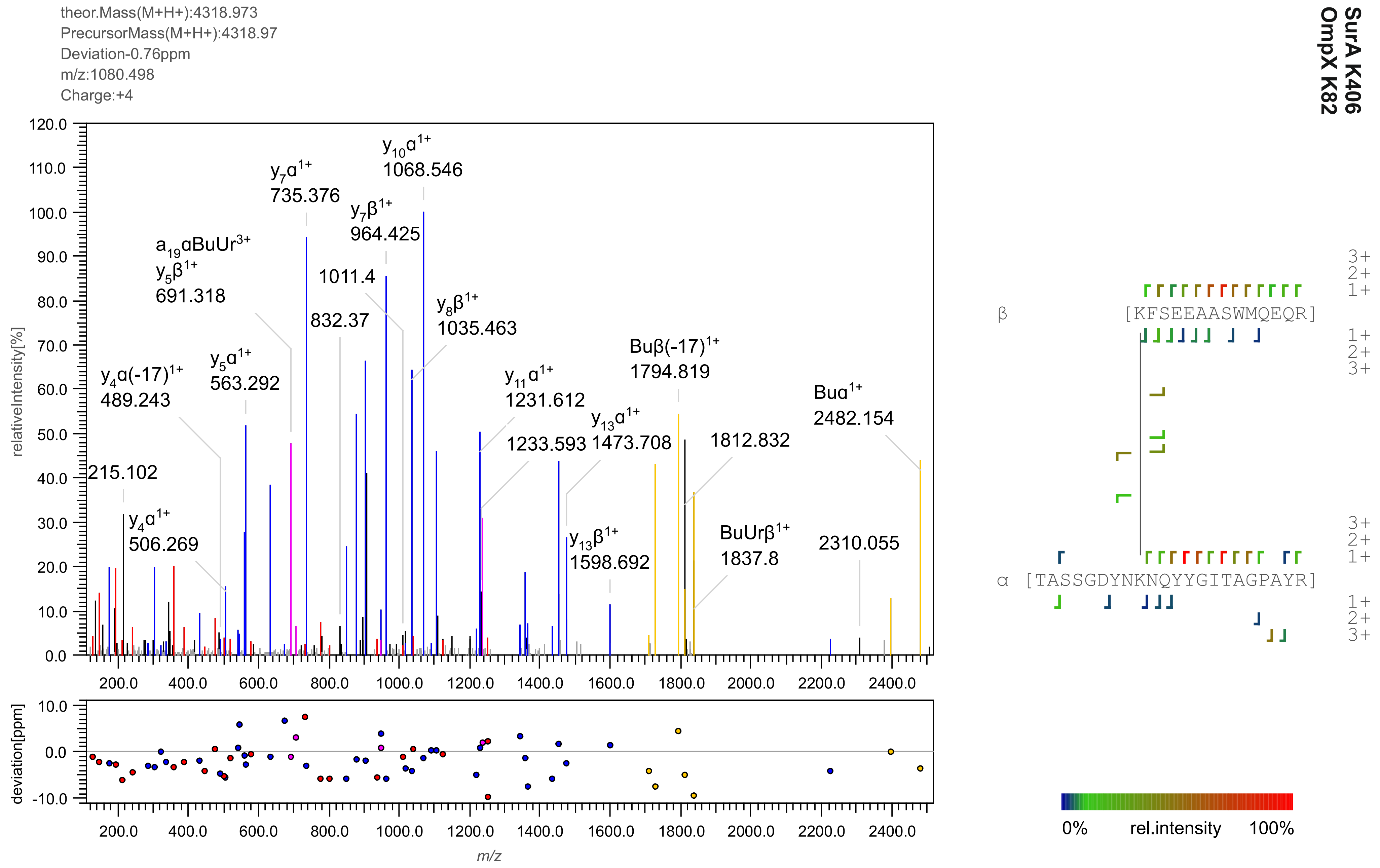

SurA K425
OmpX K82

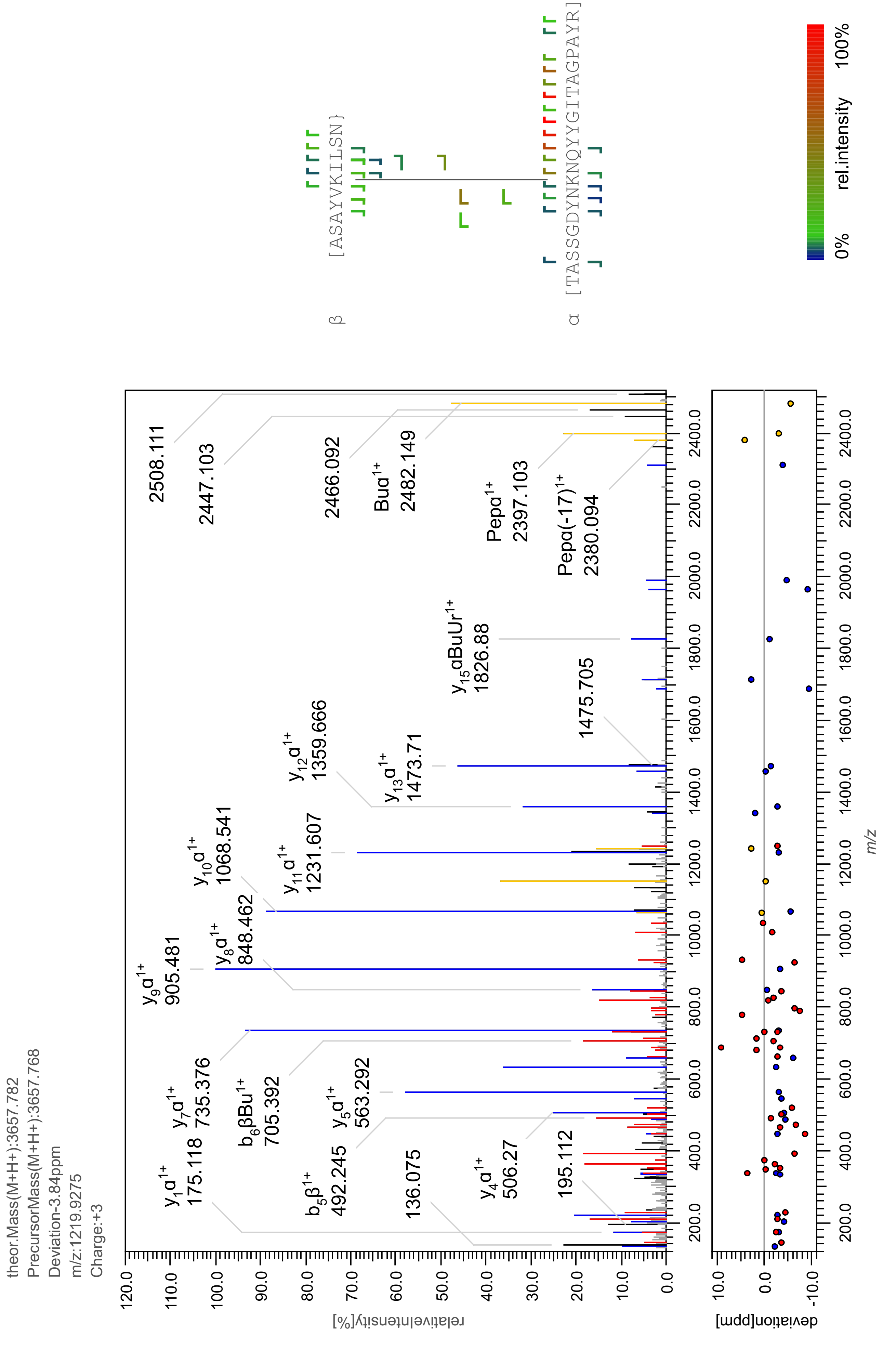

XL-MS spectra of tag-transfer crosslinks detected between SurA and OmpX.

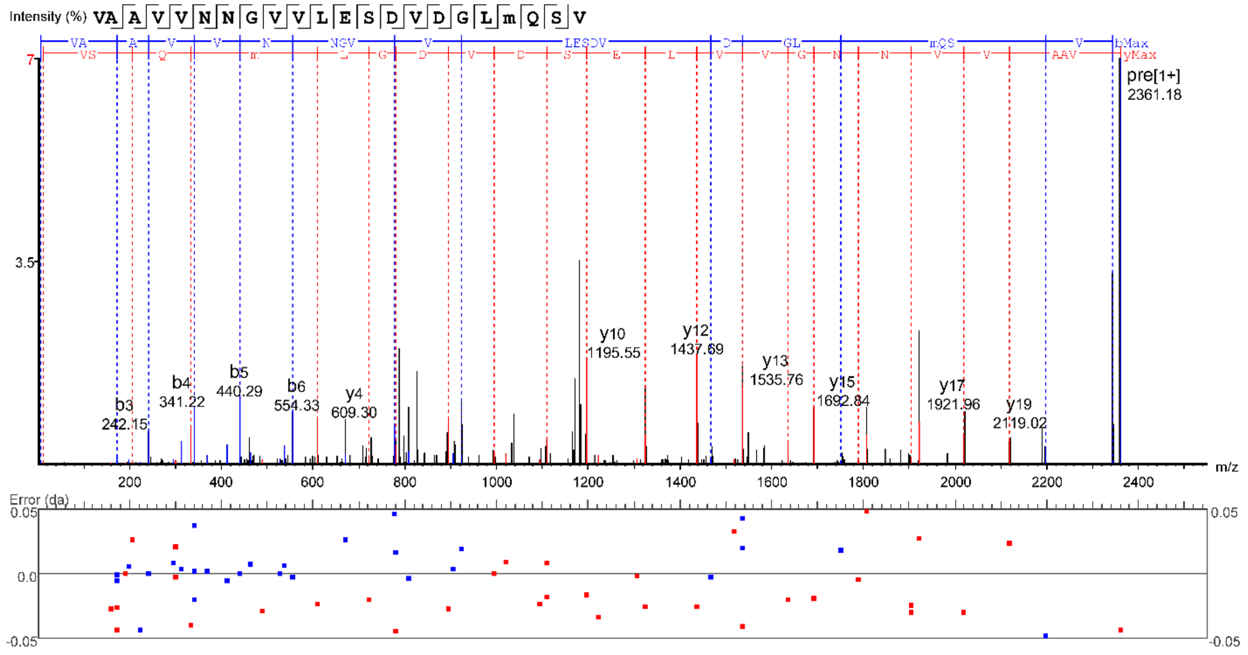
Intensity (%) VAAVVNNGVVLESDVDGLmQSV
pre[1+]
2361.18
b3
242.15
b4
341.22
b5
440.29
b6
554.33
y4
609.30
y10
1195.55
y12
1437.69
y13
1535.76
y15
1692.84
y17
1921.96
y19
2119.02
m/z
Error (da)

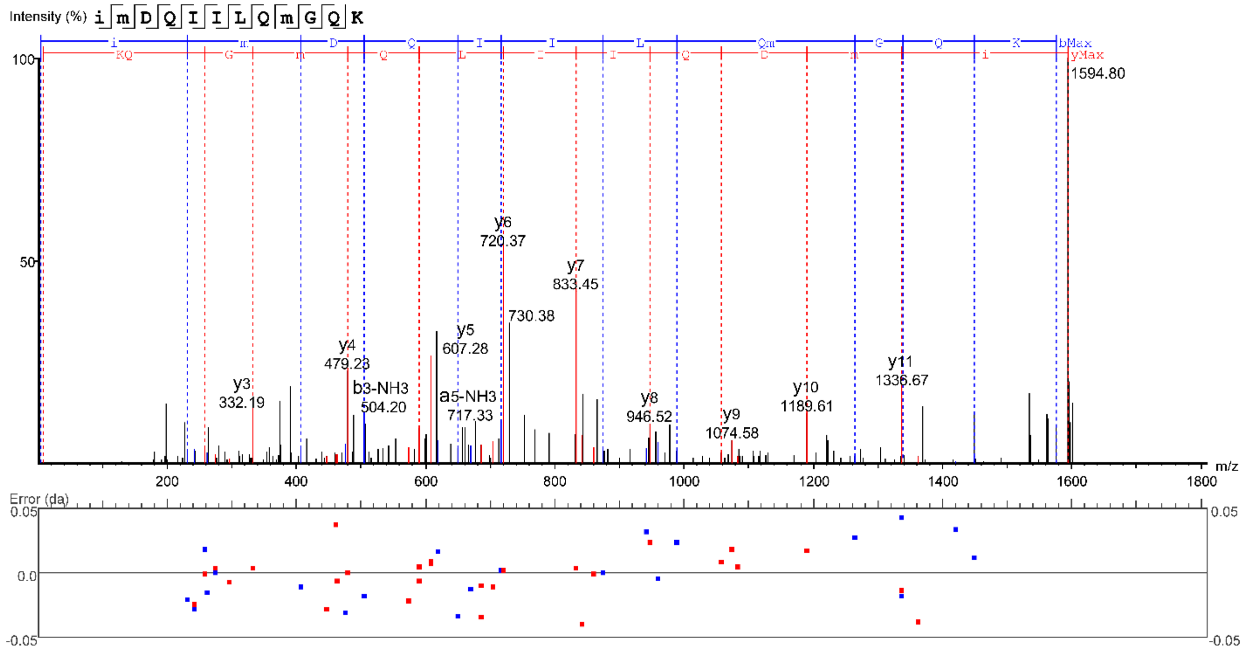
Intensity (%) imDQIILQmGQK
1594.80
y3
332.19
y4
479.23
b3-NH3
504.20
y5
607.28
a5-NH3
717.33
y6
720.37
730.38
y7
833.45
y8
946.52
y9
1074.58
y10
1189.61
y11
1336.67
m/z
Error (da)

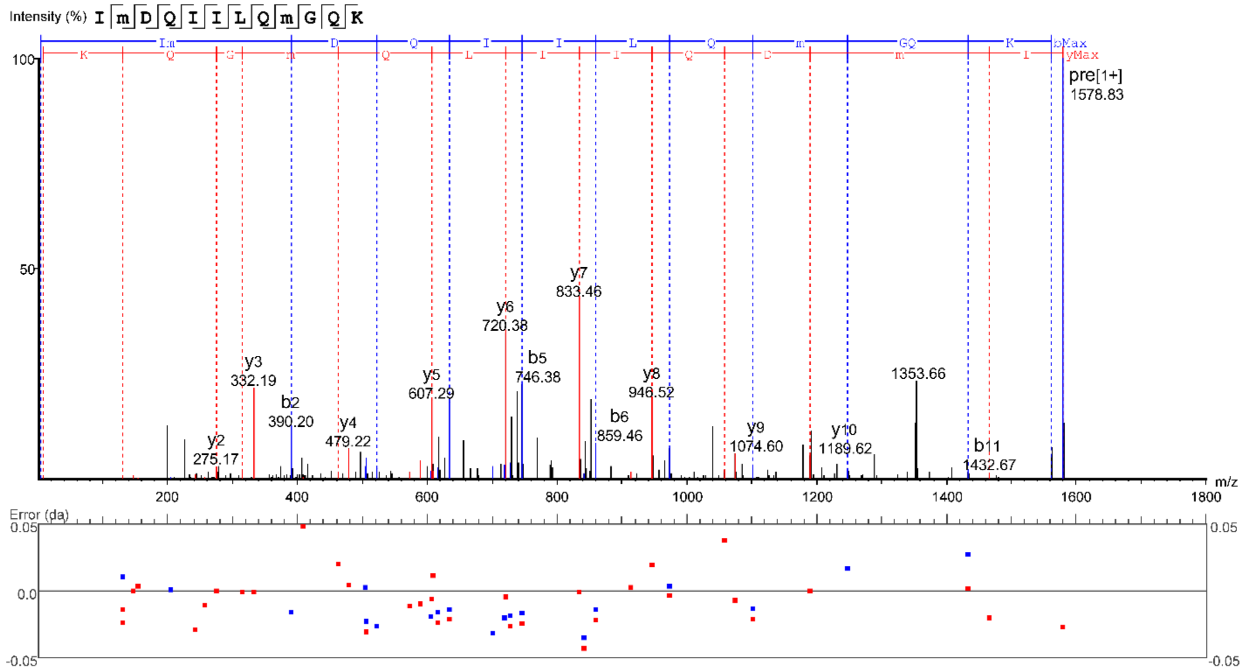
Intensity (%) I m D Q I I L Q m G Q K
pre[1+]
1578.83
y7
833.46
y6
720.38
b5
746.38
y3
332.19
y5
607.29
y8
946.52
1353.66
b2
390.20
y4
479.22
b6
859.46
y2
275.17
y9
1074.60
y10
1189.62
b11
1432.67
bMax
yMax
m/z
Error (da)
0.05
0.0
-0.05

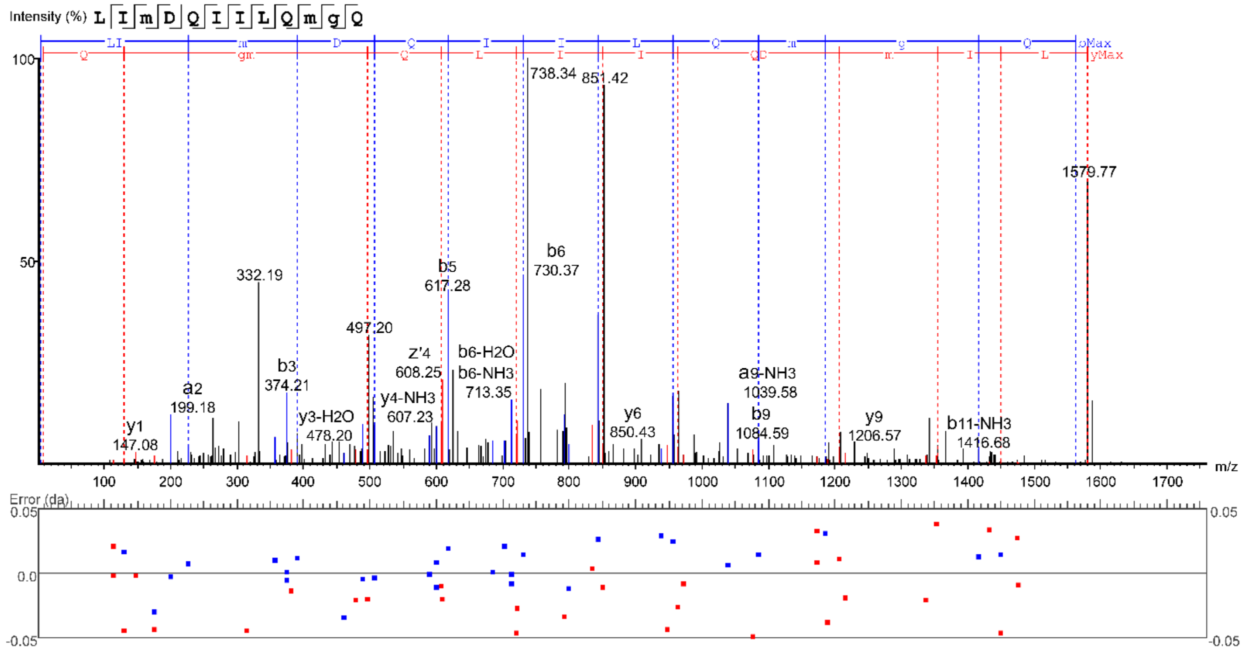
Intensity (%) L I m D Q I I L Q m g Q
738.34
851.42
1579.77
332.19
b5
617.28
b6
730.37
497.20
z'4
608.25
b6-H2O
b6-NH3
713.35
b3
374.21
a2
199.18
y4-NH3
607.23
y3-H2O
478.20
a9-NH3
1039.58
y1
147.08
y6
850.43
b9
1084.59
y9
1206.57
b11-NH3
1416.68
bMax
yMax
m/z
Error (da)
0.05
0.0
-0.05

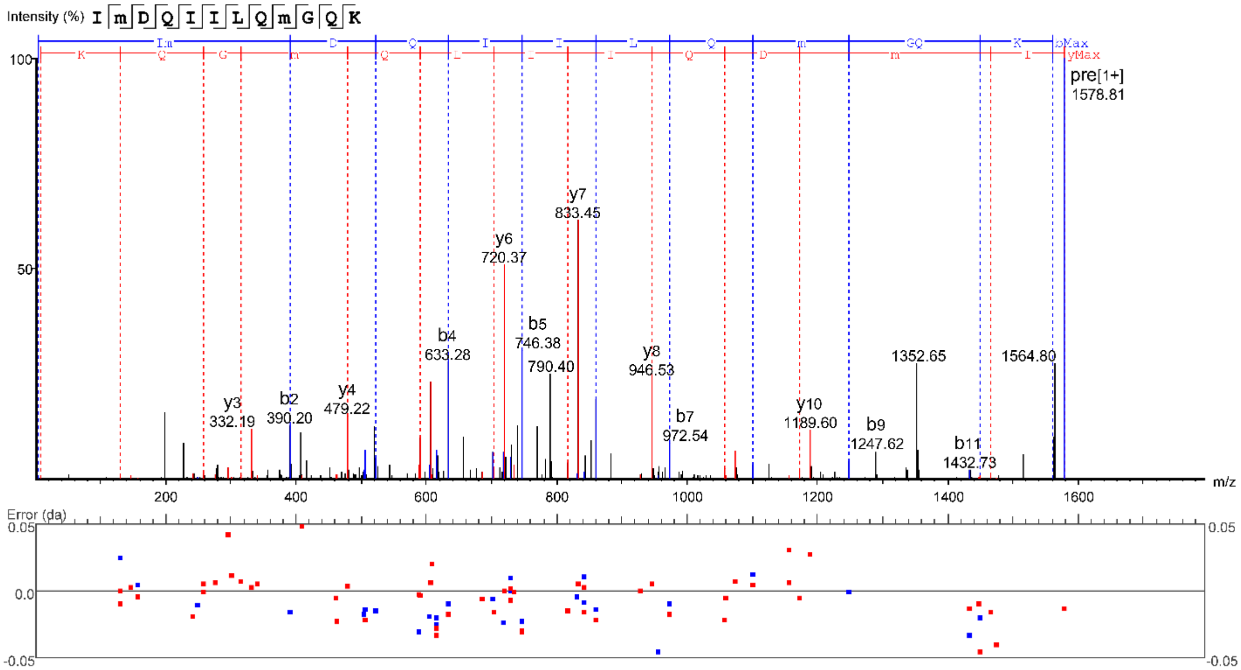
Intensity (%) I m D Q I I L Q m G Q K
100
50
pre[1+]
1578.81
y7
833.45
y6
720.37
b5
746.38
790.40
b4
633.28
y8
946.53
1352.65
1564.80
y3
332.19
b2
390.20
y4
479.22
b7
972.54
y10
1189.60
b9
1247.62
b11
1432.73
m/z
200
400
600
800
1000
1200
1400
1600
Error (da)
0.05
0.0
-0.05

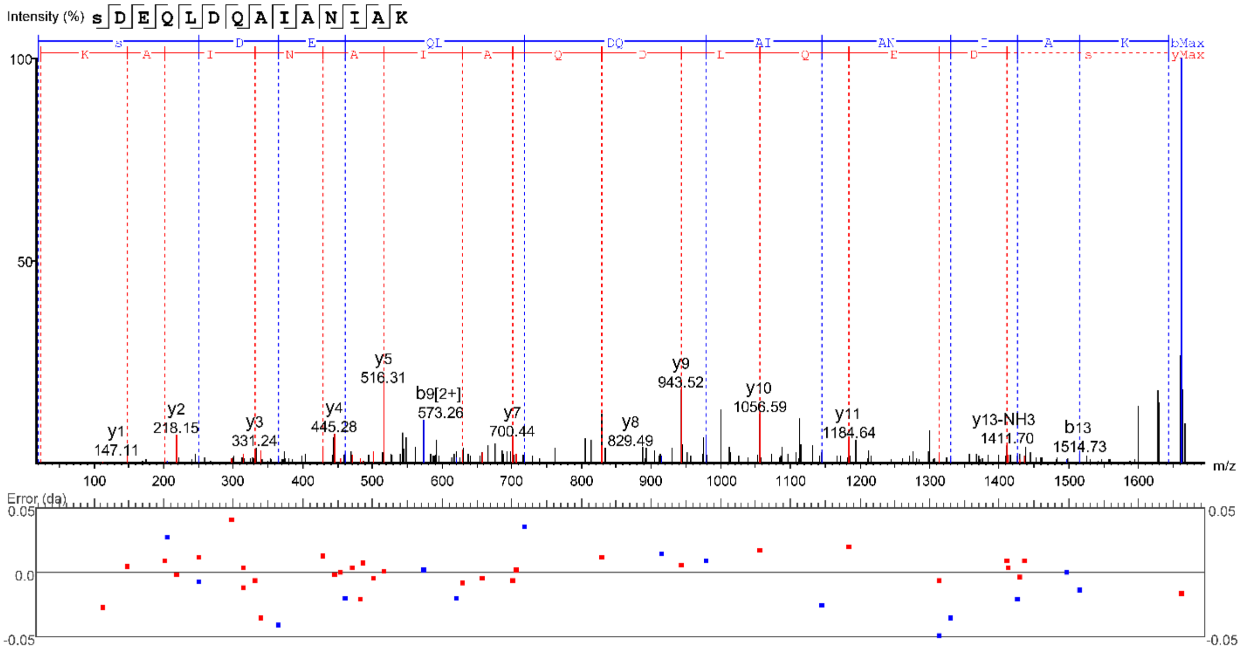
Intensity (%) s D E Q L D Q A I A N I A K
100
50
y5
516.31
y9
943.52
b9[2+]
573.26
y10
1056.59
y2
218.15
y4
445.28
y7
700.44
y11
1184.64
y13-NH3
1411.70
y1
147.11
y3
331.24
y8
829.49
b13
1514.73
m/z
100
200
300
400
500
600
700
800
900
1000
1100
1200
1300
1400
1500
1600
Error (da)
0.05
0.0
-0.05

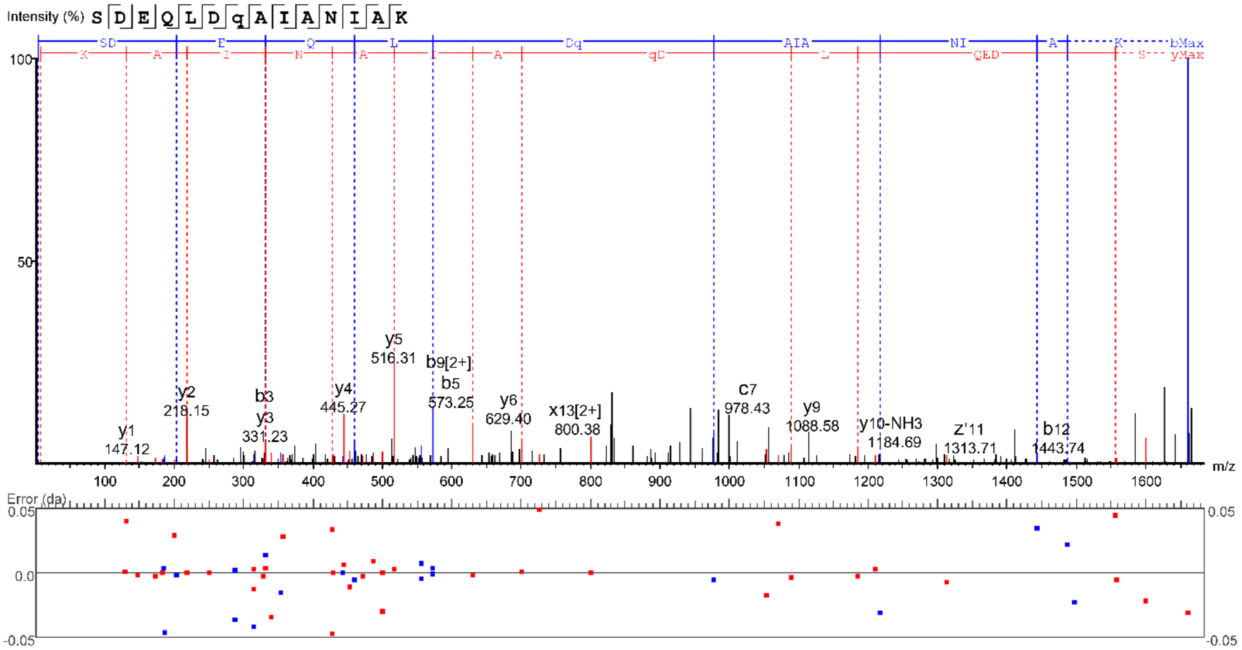

Intensity (%) S D E Q L D q A I A N I A K
y1 147.12
y2 218.15
b3 y3 331.23
y4 445.27
y5 516.31
b9[2+] b5 573.25
y6 629.40
x13[2+] 800.38
c7 978.43
y9 1088.58
y10-NH3 1184.69
z'11 1313.71
b12 1443.74
m/z
Error (da)
bMax
yMax

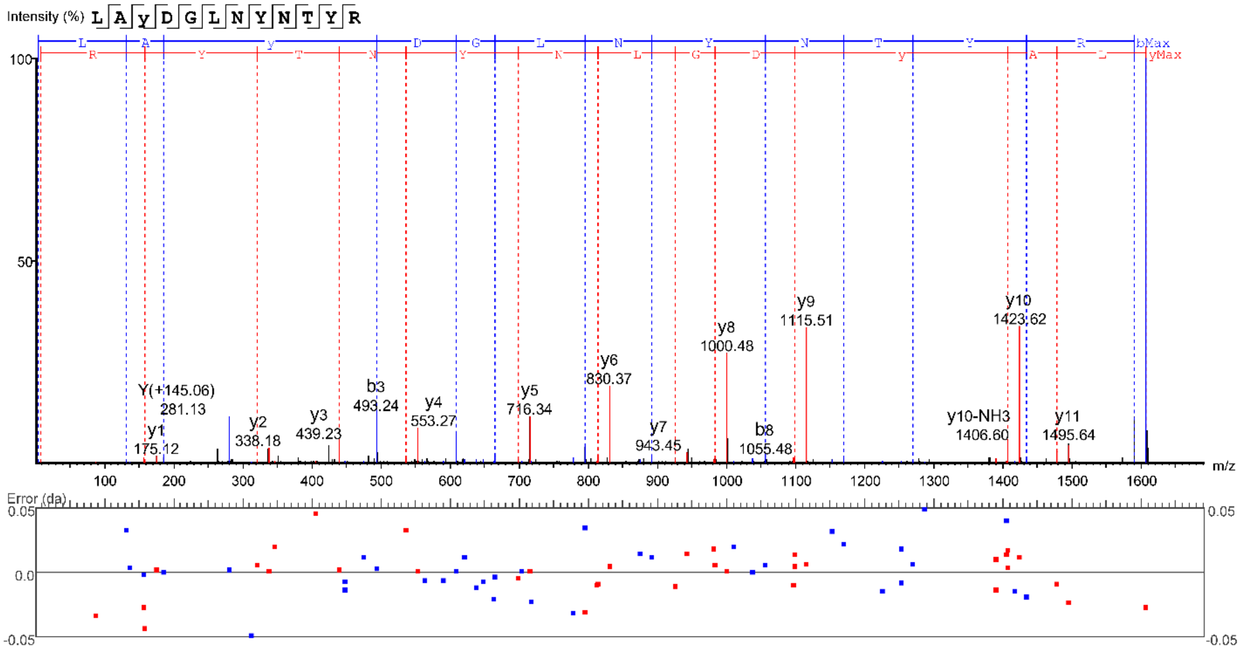

Intensity (%) L A y D G L N Y N T Y R
Y(+145.06) 281.13
y1 175.12
y2 338.18
y3 439.23
b3 493.24
y4 553.27
y5 716.34
y6 830.37
y7 943.45
y8 1000.48
b8 1055.48
y9 1115.51
y10-NH3 1406.60
y10 1423.62
y11 1495.64
m/z
Error (da)
bMax
yMax

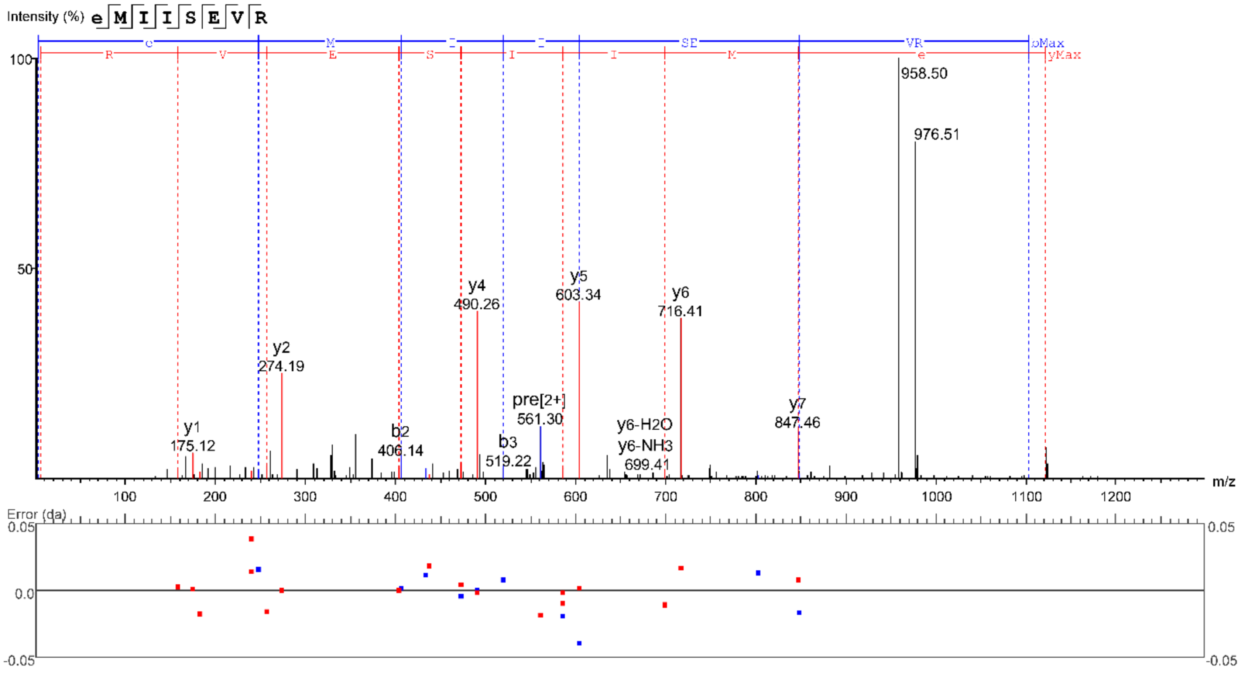
Intensity (%) e M I I S E V R
100
50
958.50
976.51
y4
490.26
y5
603.34
y6
716.41
y2
274.19
pre[2+]
561.30
y7
847.46
y1
175.12
b2
406.14
b3
519.22
y6-H2O
y6-NH3
699.41
100
200
300
400
500
600
700
800
900
1000
1100
1200
m/z
Error (da)
0.05
0.0
-0.05

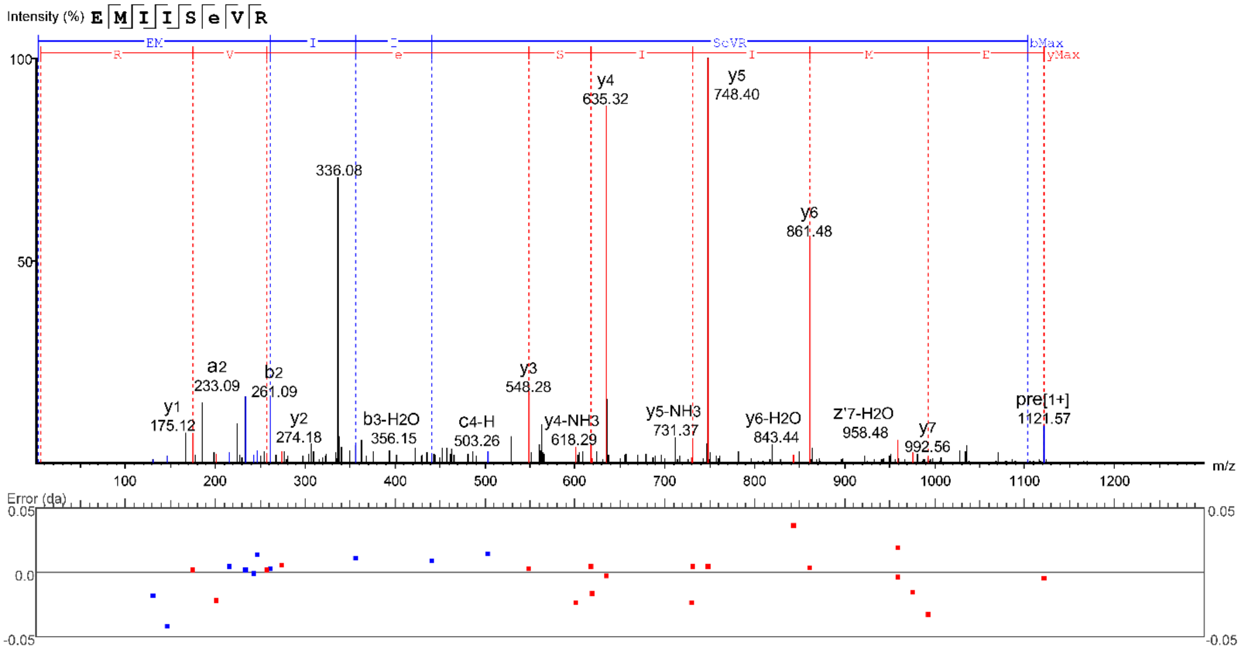
Intensity (%) E M I I S e V R
100
50
y4
635.32
y5
748.40
336.08
y6
861.48
a2
233.09
b2
261.09
y1
175.12
y2
274.18
b3-H2O
356.15
c4-H
503.26
y3
548.28
y4-NH3
618.29
y5-NH3
731.37
y6-H2O
843.44
z'7-H2O
958.48
y7
992.56
pre[1+]
1121.57
100
200
300
400
500
600
700
800
900
1000
1100
1200
m/z
Error (da)
0.05
0.0
-0.05

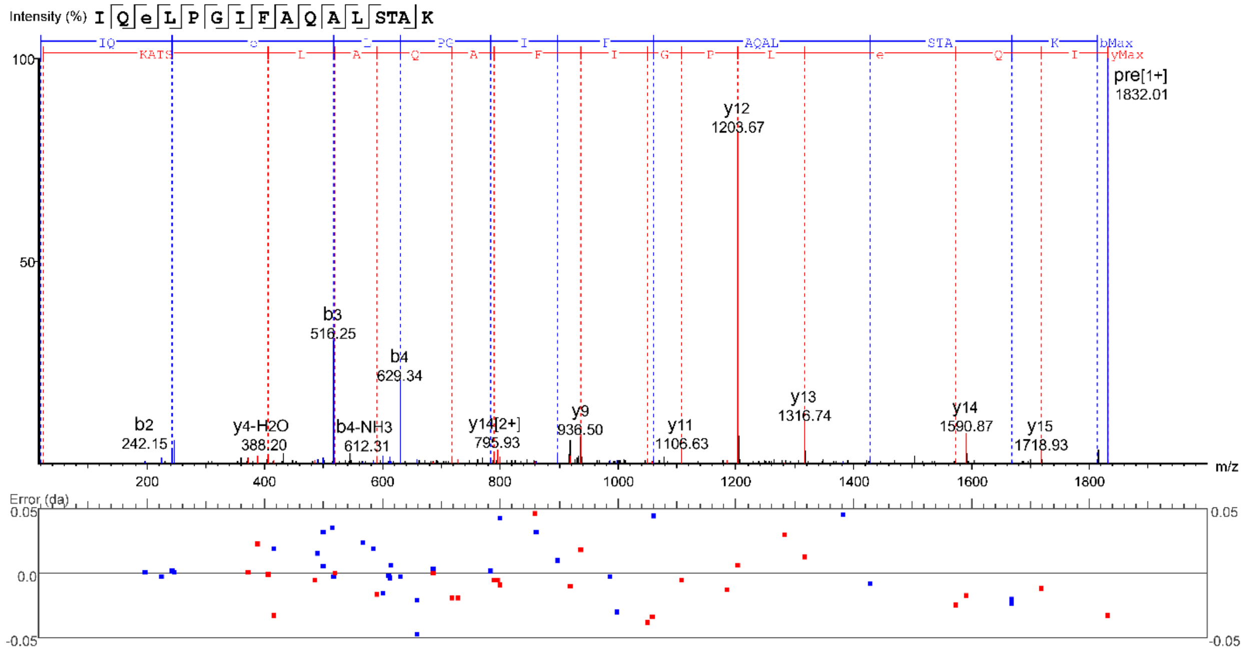
Intensity (%) I Q e L P G I F A Q A L STA K
100
50
b2
242.15
y4-H2O
388.20
b3
516.25
b4-NH3
612.31
b4
629.34
y14[2+]
795.93
y9
936.50
y11
1106.63
y12
1203.67
y13
1316.74
y14
1590.87
y15
1718.93
pre[1+]
1832.01
m/z
200
400
600
800
1000
1200
1400
1600
1800
Error (da)
0.05
0.0
-0.05

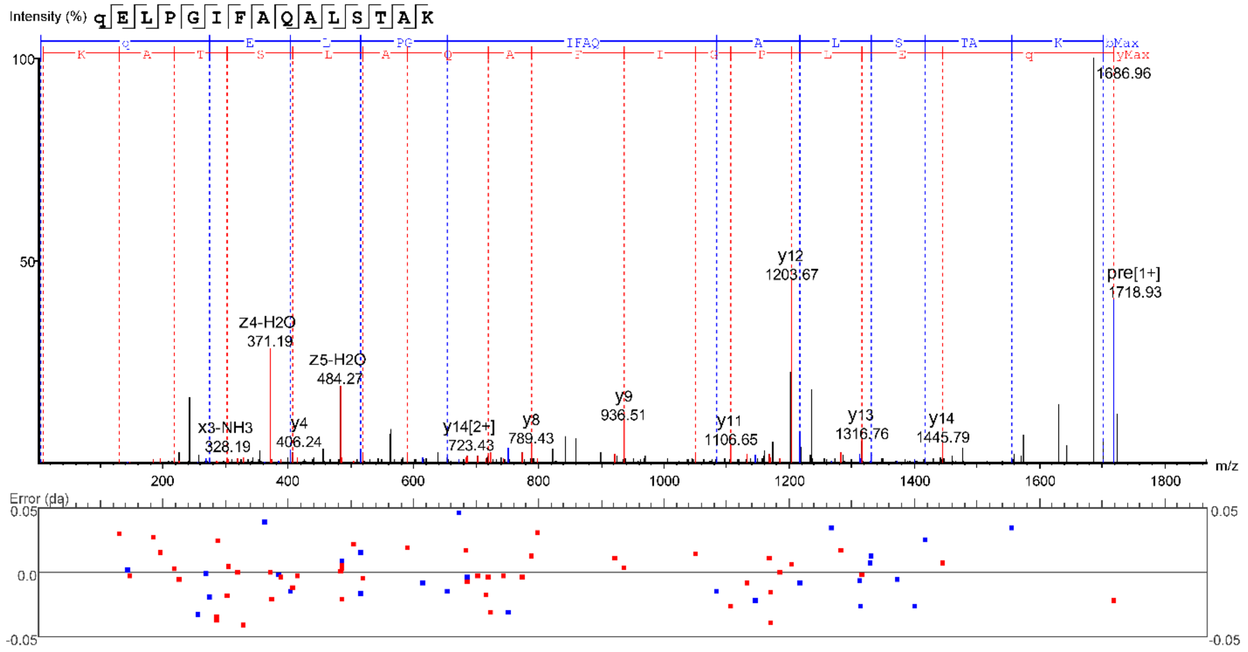
Intensity (%) q E L P G I F A Q A L S T A K
100
50
x3-NH3
328.19
z4-H2O
371.19
y4
406.24
z5-H2O
484.27
y14[2+]
723.43
y8
789.43
y9
936.51
y11
1106.65
y12
1203.67
y13
1316.76
y14
1445.79
pre[1+]
1718.93
1686.96
m/z
200
400
600
800
1000
1200
1400
1600
1800
Error (da)
0.05
0.0
-0.05
