# Supplementary material for: Inter-domain dynamics in the chaperone SurA and multi-site binding to its outer membrane protein clients
Source: Nat Commun. 2020 May 1;11:2155. doi: 10.1038/s41467-020-15702-1 (PMC7195389; doi:10.1038/s41467-020-15702-1)
Supplement: Supplementary file 8 — Reporting Summary [file 41467_2020_15702_MOESM8_ESM.pdf]

nature research

Corresponding author(s): Sheena Radford

Last updated by author(s): Mar 9, 2020

Reporting Summary

Nature Research wishes to improve the reproducibility of the work that we publish. This form provides structure for consistency and transparency in reporting. For further information on Nature Research policies, see Authors & Referees and the Editorial Policy Checklist.

Statistics

For all statistical analyses, confirm that the following items are present in the figure legend, table legend, main text, or Methods section.

n/a	Confirmed	
☐	☒	The exact sample size (n) for each experimental group/condition, given as a discrete number and unit of measurement
☐	☒	A statement on whether measurements were taken from distinct samples or whether the same sample was measured repeatedly
☒	☐	The statistical test(s) used AND whether they are one- or two-sided<br>*Only common tests should be described solely by name; describe more complex techniques in the Methods section.*
☒	☐	A description of all covariates tested
☒	☐	A description of any assumptions or corrections, such as tests of normality and adjustment for multiple comparisons
☒	☐	A full description of the statistical parameters including central tendency (e.g. means) or other basic estimates (e.g. regression coefficient) AND variation (e.g. standard deviation) or associated estimates of uncertainty (e.g. confidence intervals)
☒	☐	For null hypothesis testing, the test statistic (e.g. F, t, r) with confidence intervals, effect sizes, degrees of freedom and P value noted<br>*Give P values as exact values whenever suitable.*
☒	☐	For Bayesian analysis, information on the choice of priors and Markov chain Monte Carlo settings
☒	☐	For hierarchical and complex designs, identification of the appropriate level for tests and full reporting of outcomes
☒	☐	Estimates of effect sizes (e.g. Cohen's d, Pearson's r), indicating how they were calculated

Our web collection on statistics for biologists contains articles on many of the points above.

Software and code

Policy information about availability of computer code

Data collection	Crosslinking data were acquired using XCalibur v4.0.27.19 (Thermo Scientific) or MassLynx v4.2 (Waters). HDX-MS data were acquired using MassLynx v4.2 (Waters).<br>FRET data were collected using a custom build experimental setup for ALEX controlled in the Labview environment (LabView 7.1 Professional Development System for Windows, National Instruments).<br>Unrestrained all-atom MD simulations were performed with GROMACS 5.0.2 using the CHARMM36 force field. Simulated annealing calculations were conducted using XPLOR-NIH 2.50.<br>Fluorescence anisotropy data were collected using FelixGX v4.9.0.10243 (Horiba).<br>MST data were collected using the NT Control Software implemented on the Monolith NT.115 MST instrument (NanoTemper).
Data analysis	DSBU crosslinking data were analysed using MeroX 2.0 (http://www.stavrox.com/) and tag-transfer crosslinking data were analysed with PEAKS Studio 8.5 (Bioinformatics Solutions).<br>HDX-MS data were analysed using PLGS (v3.0.2), DynamX (v3.0.0) (Waters, UK) and Deuteros (v1.07) (Lau et al., Bioinformatics, 35, 3171–3173 (2019)).<br>MST Data fitting was carried out using IgorPro 6.3.4.1 (Wavemetrics, Oregon, USA).<br>FRET data were analysed using FRETBursts (Ingargiola et al., PLoS One 11, e0160716 (2016), Fessl et al., Elife 7, e35112 (2018)).<br>Fluorescence anisotropy data were analysed using FelixGX v4.9.0.10243 (Horiba).<br>Inter-residue Cα-Cα distances in the unrestrained MD simulations were determined using the 'gmx distance' GROMACS 5.0.2 command and calculations of solvent accessible surface distances (SASDs) made use of JWalk.

For manuscripts utilizing custom algorithms or software that are central to the research but not yet described in published literature, software must be made available to editors/reviewers. We strongly encourage code deposition in a community repository (e.g. GitHub). See the Nature Research guidelines for submitting code & software for further information.

Data

Policy information about availability of data

All manuscripts must include a data availability statement. This statement should provide the following information, where applicable:

- Accession codes, unique identifiers, or web links for publicly available datasets
- A list of figures that have associated raw data
- A description of any restrictions on data availability

MS data, have been deposited to the ProteomeXchange Consortium via the PRIDE partner repository with the dataset identifiers PXD016993 (XL-MS) and PXD017010 (HDX-MS). Source data for Figs. 4a and 5a, Supplementary Fig 7 and Supplementary Table 2 are provided with the paper. Raw smFRET data, MD simulation data and the structures of the 10 lowest energy conformations of SurA from simulated annealing are freely available at the University of Leeds data repository (https://doi.org/10.5518/701). Already deposited Protein Data Bank (PDB: 1M5Y, 2PV1, 2PV3, 3NRK, 3RGC) files were used.

Field-specific reporting

Please select the one below that is the best fit for your research. If you are not sure, read the appropriate sections before making your selection.

☒ Life sciences ☐ Behavioural & social sciences ☐ Ecological, evolutionary & environmental sciences

For a reference copy of the document with all sections, see nature.com/documents/nr-reporting-summary-flat.pdf

Life sciences study design

All studies must disclose on these points even when the disclosure is negative.

Sample size	No sample size calculations were performed. All HDX experiments and unrestrained all-atom molecular dynamics simulations were performed in triplicate to ensure they were reproducible.
Data exclusions	No data were excluded from the analysis.
Replication	All experiments were replicated at least three times and all attempts at replication were successful.
Randomization	Randomisation was not necessary in this study as it did not involve subjective trials.
Blinding	Blinding was not necessary in this study as our experiments did not involve subjective trials.

Reporting for specific materials, systems and methods

We require information from authors about some types of materials, experimental systems and methods used in many studies. Here, indicate whether each material, system or method listed is relevant to your study. If you are not sure if a list item applies to your research, read the appropriate section before selecting a response.

Materials & experimental systems

n/a	Involved in the study	
☒	☐	Antibodies
☒	☐	Eukaryotic cell lines
☒	☐	Palaeontology
☒	☐	Animals and other organisms
☒	☐	Human research participants
☒	☐	Clinical data

Methods

n/a	Involved in the study	
☒	☐	ChIP-seq
☒	☐	Flow cytometry
☒	☐	MRI-based neuroimaging
